# Supplementary material for: Adolescent awareness and experience of the pubertal changes: A qualitative study from Rwanda
Source: PLoS One. 2025 Jun 24;20(6):e0325502. doi: 10.1371/journal.pone.0325502 (PMC12186878; doi:10.1371/journal.pone.0325502)
Supplement: S1 File — (DOC) [file pone.0325502.s001.doc]

Title: Mulinga Girls transcribed.docx

Doc Creator: tcuhawenimana11

Doc Date: 3/25/2023

Codes Applied: Physiological changes occuring during puberty-Boys

Linked Memos: 0

Excerpt Creator: tcuhawenimana11

Excerpt Created On: 4/28/2023

Excerpt Range: 1108-1307

Nimero yange ni 01 imyaka yange mfite 17 niga mu wa 3. Rero ku mihindagurikire y’umwana w’umuhungu mugihe cy’ubugimbi mba numva wenda yamera nkincakwaha, akamera insya ndumva ari ibyongibyo naba nzi.

Title: Mulinga Girls transcribed.docx

Doc Creator: tcuhawenimana11

Doc Date: 3/25/2023

Codes Applied: Physiological changes occuring during puberty-Boys

Linked Memos: 0

Excerpt Creator: tcuhawenimana11

Excerpt Created On: 4/28/2023

Excerpt Range: 1350-1531

Nimero yange ni 03 mfite imyaka 16 niga muwa 3. Ngewe ibindi bimenyetso nzi bigaragaza umwana w’umuhungu ugeze mu gihe cy’ubugimbi n’ukuniga ijwi no kumera ndetse ubwanwa, murakoze.

Title: Mulinga Girls transcribed.docx

Doc Creator: tcuhawenimana11

Doc Date: 3/25/2023

Codes Applied: Boys and girls know about the causes leading to the physiological changes during puberty

Linked Memos: 0

Excerpt Creator: tcuhawenimana11

Excerpt Created On: 4/28/2023

Excerpt Range: 1599-1681

Izo mpinduka nkeka ko zaba ziterwa nuko yaba avuye mu cyiciro kimwe agiye mu kindi.

Title: Mulinga Girls transcribed.docx

Doc Creator: tcuhawenimana11

Doc Date: 3/25/2023

Codes Applied: Physiological changes occuring during puberty-Boys

Linked Memos: 0

Excerpt Creator: tcuhawenimana11

Excerpt Created On: 4/28/2023

Excerpt Range: 1770-1896

Nomero yange ni 05 imyaka yange ni 15 niga mw’ishuri rya senior 2. Nange ukuntu mbyumva umuhungu agera mu gihe cyo kwiroteraho

Title: Mulinga Girls transcribed.docx

Doc Creator: tcuhawenimana11

Doc Date: 3/25/2023

Codes Applied: Psychological changes occuring during puberty-Boys

Linked Memos: 0

Excerpt Creator: tcuhawenimana11

Excerpt Created On: 4/28/2023

Excerpt Range: 1898-2091

akaba yakwifuza no kugira igihe kihariye we n’umukobwa bakajya mu rukundo akifuza kugirana igihe Kihariye bakaganira ibyo ngibyo biterwa nuko aba yavuye mu cyiciro kimwe ajya mu kindi murakoze.

Title: Mulinga Girls transcribed.docx

Doc Creator: tcuhawenimana11

Doc Date: 3/25/2023

Codes Applied: Psychological changes occuring during puberty-Boys

Linked Memos: 0

Excerpt Creator: tcuhawenimana11

Excerpt Created On: 4/28/2023

Excerpt Range: 2140-2428

Nimero yange ni 02 imyaka14 niga snieor 2. Nge uko mbyumva numva yuko umuhungu ugeze mu gihe cy’ubugimbi yatangira kwaguka umubiri cyangwa agatangira kwiyumvamo ibindi bihe bitari bimeze nkibyambere agatangira kugera mu gihe yakumva muriwe ashaka umwanya wo kwitekerezaho kuruta na mbere

Title: Mulinga Girls transcribed.docx

Doc Creator: tcuhawenimana11

Doc Date: 3/25/2023

Codes Applied: Physiological changes occuring during puberty-Boys

Linked Memos: 0

Excerpt Creator: tcuhawenimana11

Excerpt Created On: 4/28/2023

Excerpt Range: 2428-2506

cyangwa ibyo bita ngo umuhungu ugeze mu gihe cy’ubugimbi atangira kwiroteraho.

Title: Mulinga Girls transcribed.docx

Doc Creator: tcuhawenimana11

Doc Date: 3/25/2023

Codes Applied: Boys and girls know about the causes leading to the physiological changes during puberty

Linked Memos: 0

Excerpt Creator: tcuhawenimana11

Excerpt Created On: 4/28/2023

Excerpt Range: 2554-2773

Izo mpinduka numva yuko zaba ziterwa nuko mu gihe umwana w’umuhungu yaba ageze mu cyiciro yakumva yuko atangiye kugera igihe hari impinduka zitangiye kumugeraho we yiyumvamo ibindi bihe bitandukanye nuko yarameze mbere.

Title: Mulinga Girls transcribed.docx

Doc Creator: tcuhawenimana11

Doc Date: 3/25/2023

Codes Applied: Boys and girls know about the causes leading to the physiological changes during puberty

Linked Memos: 0

Excerpt Creator: tcuhawenimana11

Excerpt Created On: 4/28/2023

Excerpt Range: 2813-3151

Nimero yange ni 01 mfite imyaka 17 niga muwa 3. Impamvu yenda icyaba kibitera kubu umuntu yagera muricyo kiciro cy’ubugimbi nkuko Imana yaturemye hari igihe umuntu ageramo akaba yakwemererwa nko kubyara, kugira abe yakwemererwa kubyara nkuko process ishobora gusaba kuba yakwiroteraho kuba izo ntanga zakwirema ibintu nkibyo ngibyo nyine.

Title: Mulinga Girls transcribed.docx

Doc Creator: tcuhawenimana11

Doc Date: 3/25/2023

Codes Applied: Physiological changes occuring during puberty-Boys

Linked Memos: 0

Excerpt Creator: tcuhawenimana11

Excerpt Created On: 4/28/2023

Excerpt Range: 3204-3339

Nimero yange ni 4 imyaka ni 16 niga senior 3. Ngewe uko mbyumva umuhungu uri mu gihe cy’ubugimbi aniga ijwi akamera ubwanwa n’incakwaha

Title: Mulinga Girls transcribed.docx

Doc Creator: tcuhawenimana11

Doc Date: 3/25/2023

Codes Applied: Boys and girls know about the causes leading to the physiological changes during puberty

Linked Memos: 0

Excerpt Creator: tcuhawenimana11

Excerpt Created On: 4/28/2023

Excerpt Range: 3340-3487

ibyo bintu rero mba numva biterwa n’imihindagurikire yo mu mubiri we bitewe n’imyaka runaka aba agezemo isaba ko process yo mu mubiri we yahinduka.

Title: Mulinga Girls transcribed.docx

Doc Creator: tcuhawenimana11

Doc Date: 3/25/2023

Codes Applied: Physiological changes occuring during puberty-Boys

Linked Memos: 0

Excerpt Creator: tcuhawenimana11

Excerpt Created On: 4/28/2023

Excerpt Range: 3501-3810

Murakoze, nimero yange ni 03 mfite imyaka 16 niga muwa 3. Rero ikintu numva nakongera mubyo nari navuze mbere nuko umuhungu ugeze mu gihe cy’ubugimbi agomba kumera insya mu myanya we my’ibarukiro ndetse akaniga ijwi kuburyo umuntu azaba umugabo mu gihe yaba afite imyaka 12 azumva ko ijwi rye ryahindutse rero

Title: Mulinga Girls transcribed.docx

Doc Creator: tcuhawenimana11

Doc Date: 3/25/2023

Codes Applied: Boys and girls know about the causes leading to the physiological changes during puberty

Linked Memos: 0

Excerpt Creator: tcuhawenimana11

Excerpt Created On: 4/28/2023

Excerpt Range: 3811-3879

nkeka ko byaba biterwa nimihindagurikire yumuntu uko ateye murakoze.

Title: Mulinga Girls transcribed.docx

Doc Creator: tcuhawenimana11

Doc Date: 3/25/2023

Codes Applied: Physiological changes occuring during puberty-Boys

Linked Memos: 0

Excerpt Creator: tcuhawenimana11

Excerpt Created On: 4/28/2023

Excerpt Range: 3947-4132

Nimero yange ni 06 mfite imyaka 14 niga muwa 1. Ibimenyetso biranga umuhungu ugeze mu gihe cy’ubugimbi aniga ijwi, agatuza ke karaguka, amera incakwaha, azana ubwanwa, yiroteraho nkaba

Title: Mulinga Girls transcribed.docx

Doc Creator: tcuhawenimana11

Doc Date: 3/25/2023

Codes Applied: Boys and girls know about the causes leading to the physiological changes during puberty

Linked Memos: 0

Excerpt Creator: tcuhawenimana11

Excerpt Created On: 4/28/2023

Excerpt Range: 4131-4299

nkeka ko ibyo bintu byose abiterwa nicyiciro agezemo aba yaravuye mu gihe cy’ubwana agatangira nyine gukura nkeka ko ibyo biterwa nimihindagurikire y’umubiri murakoze.

Title: Mulinga Girls transcribed.docx

Doc Creator: tcuhawenimana11

Doc Date: 3/25/2023

Codes Applied: Physiological changes occuring during puberty-Girls

Linked Memos: 0

Excerpt Creator: tcuhawenimana11

Excerpt Created On: 4/28/2023

Excerpt Range: 4501-4726

Murakoze, nimero yange ni 02 mfite imyaka 14 niga senior 2. Numva umwana w’umukobwa biterwa nuko impinduka agira aruko amera amabere, agatangira kwiyitaho akagira isuku, akagera nigihe cy’uko igi rye rishya akajya mu mihango.

Title: Mulinga Girls transcribed.docx

Doc Creator: tcuhawenimana11

Doc Date: 3/25/2023

Codes Applied: Pshcological changes occuring during puberty-Girls

Linked Memos: 0

Excerpt Creator: tcuhawenimana11

Excerpt Created On: 4/28/2023

Excerpt Range: 4727-5000

Ibyongibyo bikaba biterwa nuko wenda nigihe agezemo ubuzima bwe hari byinshi atangira kwiyumvamo akumva yuko ibyo kugira isuku bitandukanye nuko yarameze cyane cyane agatangira kugira inshingano ahereye nko mu rugo akumva ataba yicaye hari umwanda nuko nabyumvaga murakoze.

Title: Mulinga Girls transcribed.docx

Doc Creator: tcuhawenimana11

Doc Date: 3/25/2023

Codes Applied: Boys and girls know about the causes leading to the physiological changes during puberty

Linked Memos: 0

Excerpt Creator: tcuhawenimana11

Excerpt Created On: 4/28/2023

Excerpt Range: 5036-5267

Izo mpinduka ndumva zaba ziterwa n’igihe umubiri we waba ugezemo cyangwa uko yiyumva bitewe nukuntu muriwe yumva ameze cyangwa igihe agezemo cy’imyaka agatangira kumva yuko igihe agezemo aricyo kwitekerezaho cyane agakora ibikwiye.

Title: Mulinga Girls transcribed.docx

Doc Creator: tcuhawenimana11

Doc Date: 3/25/2023

Codes Applied: Physiological changes occuring during puberty-Girls

Linked Memos: 0

Excerpt Creator: tcuhawenimana11

Excerpt Created On: 4/28/2023

Excerpt Range: 5333-5513

Murakoze nomero yange ni 01 imyaka mfite 17 niga muwa 3. Ngewe uko mbyumva ibimenyetso biba ku mukobwa ubwo rero nge ndumva atangira kujya mu mihango, agatangira gupfundura amabere

Title: Mulinga Girls transcribed.docx

Doc Creator: tcuhawenimana11

Doc Date: 3/25/2023

Codes Applied: Pshcological changes occuring during puberty-Girls

Linked Memos: 0

Excerpt Creator: tcuhawenimana11

Excerpt Created On: 4/28/2023

Excerpt Range: 5515-5724

Ndetse bamwe muri twebwe utangira kuvuga nyine ntago nava mu rugo nsa nabi oya, uti se ubu umuntu uri bumbone nsa nabi, ibintu nkibyo ngibyo nyine agatangira no kwiyumvamo kuba yagira inshuti yumuhungu baganira

Title: Mulinga Girls transcribed.docx

Doc Creator: tcuhawenimana11

Doc Date: 3/25/2023

Codes Applied: Boys and girls know about the causes leading to the physiological changes during puberty

Linked Memos: 0

Excerpt Creator: tcuhawenimana11

Excerpt Created On: 4/28/2023

Excerpt Range: 5725-5913

nkaba nkeka yuka byaba biterwa nkuko twabivuze kuva mu cyiciro kimwe ujya mu kindi, muricyo gihe uba ugezemo uba ushobora kuba wabyara ugatwita ubwo rero biba bisaba kwirinda nuko mbyumva.

Title: Mulinga Girls transcribed.docx

Doc Creator: tcuhawenimana11

Doc Date: 3/25/2023

Codes Applied: Physiological changes occuring during puberty-Girls

Linked Memos: 0

Excerpt Creator: tcuhawenimana11

Excerpt Created On: 4/28/2023

Excerpt Range: 5962-6119

Nimero yange ni 06 mfite imyaka 14 niga muwa 1 segonderi. Ibimenyetso biranga umwana w’umukobwa ugeze mu gihe cy’ubwangavu apfundura amabere, ajya mu mihango

Title: Mulinga Girls transcribed.docx

Doc Creator: tcuhawenimana11

Doc Date: 3/25/2023

Codes Applied: Pshcological changes occuring during puberty-Girls

Linked Memos: 0

Excerpt Creator: tcuhawenimana11

Excerpt Created On: 4/28/2023

Excerpt Range: 6121-6451

atangirira kwigirira isuku ye bwite ku mubiri we no mu rugo bisanzwe ndetse ashobora yagira n’inshuti ye y’umuhungu aba yumva muri we ariko abitekereza nange bijya bimbaho, inshuti y’umuhungu natekerezaga kuzayigira ako nubundi ndayifite hari igihe umuntu ageramo nko muricyo gihe ukumva urayikeneye bikaba ngombwa ko uyigira rero

Title: Mulinga Girls transcribed.docx

Doc Creator: tcuhawenimana11

Doc Date: 3/25/2023

Codes Applied: Boys and girls know about the causes leading to the physiological changes during puberty

Linked Memos: 0

Excerpt Creator: tcuhawenimana11

Excerpt Created On: 4/28/2023

Excerpt Range: 6452-6731

nkakeka ko ibyo bintu biterwa nigihe umuntu aba agezemo. Iyo uri umwana ibyo bintu ntabwo nabitekerezaga ariko muriki gihe ngezemo nsigaye mbitekereza ubwo rero ndatekereza ko impamvu ari igihe umuntu avamo akava mu gihe cyubwana akagera muricyo gihe cybwangavu niko mbitekereza.

Title: Mulinga Girls transcribed.docx

Doc Creator: tcuhawenimana11

Doc Date: 3/25/2023

Codes Applied: Pshcological changes occuring during puberty-Girls

Linked Memos: 0

Excerpt Creator: tcuhawenimana11

Excerpt Created On: 4/28/2023

Excerpt Range: 6837-7395

Murakoze nimero yange ni 5 imyaka ni 15 niga muwa 2. Nange nyine ukuntu mbyumva iyo umukobwa ageze mu bwangavu atangira kwiyumva nyine ashaka ko abantu bose bamureba nkuko abandi babivuze akumva akeneye nk’inshuti y’umuhungu bazaza baganira ibyongibyo rero nkumva ko biterwa nuko umukobwa aba avuye mu cyiciro kimwe ajya mu kindi bigatuma rero ashaka kwiyumva, kugira uwo aganira nawe, isuku mu rugo, rimwe na rimwe rero bikaba binadusaba kwirinda kugiranga ngo nyine twifate kuko iyo tugeze mu bwangavu bituma twandura indwara ninda zitateganyijwe murakoze.

Title: Mulinga Girls transcribed.docx

Doc Creator: tcuhawenimana11

Doc Date: 3/25/2023

Codes Applied: Physiological changes occuring during puberty-Girls

Linked Memos: 0

Excerpt Creator: tcuhawenimana11

Excerpt Created On: 4/28/2023

Excerpt Range: 7431-7697

Nimero yange ni 03 imyaka yange 16 niga mu cyiciro cyamashuri yisumbuye muwa 3 kibanza. Ngewe ukuntu mbyumva ndumva ko umukobwa ugeze mu gihe cy’umbwangavu ashobora kumera amabere, ashobora kujya mu mihango ndetse ashobora no kwaguka mu matako aribyo twita nkamataye

Title: Mulinga Girls transcribed.docx

Doc Creator: tcuhawenimana11

Doc Date: 3/25/2023

Codes Applied: Pshcological changes occuring during puberty-Girls

Linked Memos: 0

Excerpt Creator: tcuhawenimana11

Excerpt Created On: 4/28/2023

Excerpt Range: 7698-7773

ndetse ashobora kumva muri we akeneye nk’umuntu baganira badahuje igitsina.

Title: Mulinga Girls transcribed.docx

Doc Creator: tcuhawenimana11

Doc Date: 3/25/2023

Codes Applied: Boys and girls know about the causes leading to the physiological changes during puberty

Linked Memos: 0

Excerpt Creator: tcuhawenimana11

Excerpt Created On: 4/28/2023

Excerpt Range: 7774-7924

Ibyongibyo rero nkakeka ko byab biterwa no kuba umuntu abba avuye mu cyiciro cyo kuba umwana ageze mu cyiciro cyo kuba umuntu mukuru uko niko mbyumva.

Title: Mulinga Girls transcribed.docx

Doc Creator: tcuhawenimana11

Doc Date: 3/25/2023

Codes Applied: Physiological changes occuring during puberty-Girls

Linked Memos: 0

Excerpt Creator: tcuhawenimana11

Excerpt Created On: 4/28/2023

Excerpt Range: 7945-8181

Murakoze, nomero yange ni 04 imyaka ni 16 niga senioe 3. Ngewe uko mbyumva bagenzi uko babivuze umwana w’umukobwa ugeze mu kigero cy’umbwangavu atangira kumera amabere akajya mu mihango akazana nizindi mpinduka zitandukanye ku mubiri we

Title: Mulinga Girls transcribed.docx

Doc Creator: tcuhawenimana11

Doc Date: 3/25/2023

Codes Applied: Pshcological changes occuring during puberty-Girls

Linked Memos: 0

Excerpt Creator: tcuhawenimana11

Excerpt Created On: 4/28/2023

Excerpt Range: 8182-8414

ukuntu mbyumva icyaba kibitera ni imyaka abagezemo imutera kumva ko akeneye umuhungu baba inshuti cg boyfriend bakaba baganira kd umukobwa ugeze mu gihe cy’ubwangavu aba agomba kwirinda kubera yuko gushukika biba byoroshye murakoze.

Title: Mulinga Girls transcribed.docx

Doc Creator: tcuhawenimana11

Doc Date: 3/25/2023

Codes Applied: Precautions to take during puberty for girls

Linked Memos: 0

Excerpt Creator: tcuhawenimana11

Excerpt Created On: 4/28/2023

Excerpt Range: 8309-8414

kd umukobwa ugeze mu gihe cy’ubwangavu aba agomba kwirinda kubera yuko gushukika biba byoroshye murakoze.

Title: Mulinga Girls transcribed.docx

Doc Creator: tcuhawenimana11

Doc Date: 3/25/2023

Codes Applied: Physiological changes occuring during puberty-Girls

Linked Memos: 0

Excerpt Creator: tcuhawenimana11

Excerpt Created On: 4/28/2023

Excerpt Range: 8474-8655

Nimero yange ni 01 imyaka yange ni 17 niga muwa 3. Ikindi kintu nakongeraho umukobwa ugeze mu gihe cy’ubwangavu ashobora kumera incakwaha, akamera insya na bimwe bita amaribori yego

Title: Mulinga Girls transcribed.docx

Doc Creator: tcuhawenimana11

Doc Date: 3/25/2023

Codes Applied: Pshcological changes occuring during puberty-Girls

Linked Memos: 0

Excerpt Creator: tcuhawenimana11

Excerpt Created On: 4/28/2023

Excerpt Range: 8656-8907

ikindi kintu rero numva bamwe tuba twibonaho tuba tunashaka umuntu udu kontorora. Mama akaba yakubwira ati mwana wange kora iki ati oya mama ubwo urabo batanseka? Mwana wange jya kugura umunyu ndasa nabi ntabwo najyayo banseka ibintu nkibyo murakoze.

Title: Mulinga Girls transcribed.docx

Doc Creator: tcuhawenimana11

Doc Date: 3/25/2023

Codes Applied: How girls experience changes occuring to them during puberty

Linked Memos: 0

Excerpt Creator: tcuhawenimana11

Excerpt Created On: 4/28/2023

Excerpt Range: 9162-9838

Nimero yange ni 5 imyaka ni 15 niga muwa. Nge nyine ubwa mbere njya mu mihango byarantunguye gusa nyine kuko nabanaga na bakuru bange ntabwo byigeze bintungura cyane barabimbwiraga bakabinganiriza ndetse nyine na mummy akabimbwira noneho nyine nabigiyemo ntago nyine narimbisobanukiwe neza gusa nyine nabigiyemo bintera ubwoba ako ntabwo byigeze binkanga cyane ndagenda mbibwira mukuru wange niwe wa mbere nabibwiye arambwiye ngo rero kubera ko byose twabikwigishije, ibintu byose narimbizi kwifungira pad nyine byose narinzi kubyikorera, ndagenda nyine ni nawe wabimbwiye murugo nyine singe wabibibwiriye gusa nyine ntabwo byigeze binkanga cyane nge ndumva aricyo nabivugaho.

Title: Mulinga Girls transcribed.docx

Doc Creator: tcuhawenimana11

Doc Date: 3/25/2023

Codes Applied: How girls experience changes occuring to them during puberty

Linked Memos: 0

Excerpt Creator: tcuhawenimana11

Excerpt Created On: 4/28/2023

Excerpt Range: 9839-11129

Nimero yange ni 01 imyaka ni 17 niga muwa 3. Rero ku mpinduka zaba zarambayeho kugiti cyange ndabyibuka nabanaga na papa gusa nuko njya mu mihango bwa mbere ntabwo nabanaga na mama cyane rero ntabyo narinzi ngo barakwicaza barakubwiye ngo bigenze bite. Nuko nari nicaye ntebe turimo turareba televiziyo nuko ndahaguruka papa arambwira ngo ngo wiyanduje wenda we yarazi ko nsanzwe mbijyamo kuko ntabwo yarazi, hanyuma ndahindukira ndebe ijipo ndamubwira ngo ni imiti yikaramu yangiyeho nuko nyine ndangije njya muri toilet ndabibona ko bimeze bityo. Ndabyibuka nararize ndavuga ni nitemye papa arambaza ati se witemye hehe nigiki cyakubaze ibintu nkibyo nyine birantungura bintera ubwoba, ntera ubwoba papa ngo tujye kwa muganga we agirango kuko ari ibintu bisanzwe biba agirango wenda hari ikindi kibazo cyavutsemo tujye kwa muganga. Turagenda tugezeyo barambaza ngo ese wagiye mu mihanga? Ati se nibiki ibyongibyo? Nyine tubwire wagiye mumihango? Ati wapi none ibi nibiki urimo? Ati nyine nabonye amaraso aza ntabwo nzi aho nitemye gusa ndumva mbabara. Urarababara hehe? Ndababwira ngo mu nda ati niba ariho nitemye ntabwo mbizi, muganga araseka bajya kugura pad barayimfungira hanyuma aunt ahita aza mu rugo angira inama ukuntu bigenda arabinganiriza nyine ibintu nkibyo ngibyo murakoze.

Title: Mulinga Girls transcribed.docx

Doc Creator: tcuhawenimana11

Doc Date: 3/25/2023

Codes Applied: How girls experience changes occuring to them during puberty

Linked Memos: 0

Excerpt Creator: tcuhawenimana11

Excerpt Created On: 4/28/2023

Excerpt Range: 11204-12136

Nimero yange ni 06 mfite imyaka 14 niga muwa 1. Impinduka zambayeho nge ku giti cyange ndabyibuka narimfite imyaka 13 narindi mu rugo narintetse icyayi nicaye kuga stool mama ntawaruhari gusa ariko ndangije ndahaguruka. Mpagurutse nari nambaye ijipo mpagurutse ndebye kuga stool mbona hariho ibintu by’amaraso ndangije ndebye nyine kwijipo nsanga hagiyeho amaraso gusa nge ntabwo byantunguye kuko ni ibintu narimbinyereye mama n’ubundi yari yarabimbwiye kenshi nkiri umwana yakundaga kubinganiriza ntabwo byigeze bintungura naravuge ni ubwo nange nakuze ntabwo ari ibintu byigeze bintungura cyane kuko mama yakundaga kubimbwira akabinganiriza akenshi nyine nicyo kiganiro akabinganiriza uko umwana wumukobwa iyo agiye mu mihango agomba kwitwara nimpinduka zingenda zikubaho nyine zose arazimbwira ntabwo byigeze bintungura kuko ni ibintu narinsanzwe numva mbizi ba tante ba masenge barabimbwiraga ntabwo byigeze bintungura, urakoze.

Title: Mulinga Girls transcribed.docx

Doc Creator: tcuhawenimana11

Doc Date: 3/25/2023

Codes Applied: How girls experience changes occuring to them during puberty

Linked Memos: 0

Excerpt Creator: tcuhawenimana11

Excerpt Created On: 4/28/2023

Excerpt Range: 12264-12923

Murakoze, nimero yange ni 03 mfite imyaka 16 niga muwa 3 secondary. Ngewe impinduka zambere nabonye nameze amabere ndangije kuko nakinanaga nabana bo murungano arko nyamera mbere yabo bo batari bayamera noneho bakajya birirwa banseka ngo reba runaka yameze amabere mbere yacu kd tumuruta noneho nge nkumva binteye ipfunwe noneho ngenda negera mama ndamubaza ni ese mama kuki banseka kd mbona namwe muyafite ntakibazo? Ati impamvu baguseka nyine baracyari abana ntabwo muri mukicyiro kimwe arambwira ati uzagenda ubona nizindi mpinduka ku mubiri wawe arko nyine uzamenye uko ubyitwaramo nyine agenda anyigisha zimwe kurizo mpinduka n’ukuntu nzajya mbyitwaramo.

Title: Mulinga Girls transcribed.docx

Doc Creator: tcuhawenimana11

Doc Date: 3/25/2023

Codes Applied: How girls experience changes occuring to them during puberty

Linked Memos: 0

Excerpt Creator: tcuhawenimana11

Excerpt Created On: 4/28/2023

Excerpt Range: 12957-13245

Nko kujya mu mihango, yarambwiye ati rero nujya mu mihango bwa mbere uzambwire ngufashe kuko ntabwo uzaba ubisobanukiwe neza nyine ndagenda ndamwegera ndamubwira amfasha kugura ibikoresho byisuku ndangije anyigisha nukuntu mbikoresha ubwongubwo nange ngenda mbimenyera gake gake murakoze.

Title: Mulinga Girls transcribed.docx

Doc Creator: tcuhawenimana11

Doc Date: 3/25/2023

Codes Applied: How girls experience changes occuring to them during puberty

Linked Memos: 0

Excerpt Creator: tcuhawenimana11

Excerpt Created On: 4/28/2023

Excerpt Range: 13348-14109

Murakoze, nimero yange ni 04 imyaka ni 16 niga senior 3. Nge impinduka zambayeho nyine nameze amabere numva binteye isoni cyane najya mu muhanda isoni zikanyica nkakunda kwifubika cyane no kuzuba noneho kubera ko kubera kwambara ikote cyane kuzuba karitsiye yacu batangira kuvuga ngo akose uriya mwana afite ikihe kibazo abantu benshi bakanavuga ngo buriya aba atwite aba ahisha inda kandi atari nabyo. mama nyine yaje kumbwira ati ibyo ibintu biba bisanzwe ku mwana wumukobwa ati ubwo ni icyerekana ko utangiye gukura nza kubimenyera nkajya nambara imipira isanzwe ntashyizeho ibikote iminsi ishize ndabimenyera tuuh gusa najyaga murungano nkumva mfite isoni nange nkajya kwiga nifubika rimwe na rimwe ku kigo umupira bakawutwara gusa nkihangana tuuh murakoze.

Title: Mulinga Girls transcribed.docx

Doc Creator: tcuhawenimana11

Doc Date: 3/25/2023

Codes Applied: How girls experience changes occuring to them during puberty

Linked Memos: 0

Excerpt Creator: tcuhawenimana11

Excerpt Created On: 4/28/2023

Excerpt Range: 14430-15113

Murakoze nyine, ngewe umuhungu nyine ndabyibuka umuhungu aza kumbwira bwa mbere ko ankunda hhhh narinkiri umwana gusa bwa mbere barazaga nkababwira wapi ntabwo mbizi ibintu byo gukundana nkanabahakanira. Gusa kuko nakuze mbona abantu bakuru bahoraga bambwira ngo ntihazagire umuhungu ugukorakora, iyo bagukorakoye nyine bihita birangira noneho umuhungu yaza no kumbwira nyine ngo ndagukunda ngo dukundane nkabanza kumubwira nge nge ntabwo ntakundana nawe nge nta muhungu ujya unkorakora, abahungu bose rimwe na rimwe nabo dukundanye pe bakabyemera ntihagire numwe unabinkora ibyongibyo nabyo byatumye hari bimwe nyine menya no kubana nabantu bakuru nabyo nyine byanyigishije byinshi.

Title: Mulinga Girls transcribed.docx

Doc Creator: tcuhawenimana11

Doc Date: 3/25/2023

Codes Applied: How girls experience changes occuring to them during puberty

Linked Memos: 0

Excerpt Creator: tcuhawenimana11

Excerpt Created On: 4/28/2023

Excerpt Range: 15153-17414

Murakoze, nimero yange ni 02 mfite 14 niga senior 2. Ngewe wenda ahantu natekerezaga nimpamvu byambayeho bikantungura buriya iyo umuntu ari umwana wenda abona abantu bafite amabere agakina nibintu byabana ugasanga afashe nkibintu byibibuto yishyizeho. Nyine nyitangira gupfundura amabere narabyishimiye hanyuma rero bigenze gutyo impinduka zabayeho zikantungura nuburyo bakundaga kumbwira ngo iyo umukobwa yagiye mu mihango aba afite ikindi kigero yagezemo yatandukanye nuko yarari nyine bakambwira uburyo umukobwa yatangira kwitwara. Noneho ngewe icyo gihe nagiye mu mihango ndimo ndajya kwishuri none ngeze munzira numva ibintu ndavuga ati wenda sinzi uko bigenze ndakomeza ndagenda ngeze kwishuri njya muri toilet nyine nsanga amaraso yaje kuko nubundi mu rugo bakundaga kubivugaho ndavuga ni niyo umunyeshuri nyine aragenda ansabira pad anyereka ukuntu bayambara ndayambara ndavuga kubera mu rugo hari hariyo nyine abantu bazambara ndavuga ati ntabwo nabivuga banseka ndaceceka nyine igihe cyambere narinze nyivamo ntanumuntu ubimenye kuko navugaga ati nibabimenya noneho tuzajya twicara munzu baseke ngo nagiye mu mihango, bakambaza ati se wabaye iki nkababwira ati ntakibazo mfite nkababwira ati nuko nyine numva narushye cyane nageze cyane kwishuri. Izo mpinduka zimbonekaho gusa najyaga nitinya kugirango mbibwire ababyeyi gusa icyatumwe nitinyuka noneho nkabibwira ababyeyi nuko nayigiyemo bwa kabiri ndavuga ati ntabwo nabona amafaranga yo kugura ibikoresho nabandi ntabyo bafite ngo abe aribyo nkoresha nyine ndabibabwira baranseka nyine bo bakagirango nyigiyemo bwa mbere bakanseka ngo runaka yagiye mu mihango nyine ubwo baba bazamuye nkikiganiro turi kurya bakanseka nkagira ubwoba nyine nkagenda nkaryama nkavuga ni ntangiye no kubyanga ni bibi noneho uko umubiri ugenda uhinduka nkagenda niyumvamo izindi mpinduka zuko nyine ntagomba kuva mu rugo hasa nabi ko bataseka ababyeyi bange ahubwo aringe baseka nkatangira kwiyumvamo ko ngeze mu kigero cyuko ngomba kwiyitaho kuburyo naho genda nyura bavuga ngo runaka ameze gutya bituma nyine nterwa ishema no kugira isuku yange no mu rugo kugirango wenda batazavuga ngo runaka wenda aba asa neza ako iwabo hasa nabi rero bintera ishema ryo kumva ko hari ahandi hantu ngeze ngombye kwiyitaho murakoze.

Title: Mulinga Girls transcribed.docx

Doc Creator: tcuhawenimana11

Doc Date: 3/25/2023

Codes Applied: Information that girls need

Linked Memos: 0

Excerpt Creator: tcuhawenimana11

Excerpt Created On: 4/28/2023

Excerpt Range: 17740-18476

Murakoze, nimero yange ni 01 imyaka yange ni 17 niga muwa 3. Ngewe inama nakumva yakabaye agirwa ndumva yakabaye wenda agirwa nk’inama wenda nkababyeyi be bakamukangurira bakamubwira ati ibihe ugezemo ntago ari byiza kuko buriya iyo tugeze muri biriya bihe cyangwa abandi iyo bageze muri biriya bihe habamo kuba washukwa kuba havamo ibishuko kuba wakwiyangiriza ejo hazaza mu bintu byimikino byahongaho nizo nshuti twavugaga zabahungu yego ntago ari bibi arko nanone buri kimwe iyo ugikoresheje nabi cyaba kibi. Ubwo rero hakavamo nko kuba watwara inda, kwandura indwara ibintu nkibyongibyo ubwo rero ababyeyi bacu ndumva batwicaza bakatuganiriza bakabitubwira mbere yuko bitubaho kuburyo bizajya bitubaho twarangije kubimenya murakoze.

Title: Mulinga Girls transcribed.docx

Doc Creator: tcuhawenimana11

Doc Date: 3/25/2023

Codes Applied: Suggestions to improve SRH during puberty and adolescence by girls

Linked Memos: 0

Excerpt Creator: tcuhawenimana11

Excerpt Created On: 4/28/2023

Excerpt Range: 18514-18992

Nimero yange ni 06 mfite imyaka 14 niga senior 1. Ndatekereza ko ababyeyi bacu bagakwiye kutuganiriza mbere yigihe tukiri abana tutarakura dore ko iyo dukuze turatangira tukananirana wamubwira ntiyumve. Ndatekereza ko rero bazajya batugira inama tukiri bato kugirango tuzazikurikize tubumvire bakatubwira bakatwigisha bakatubwira ibijyanye nubuzima bwacu bwimyororokere bakatuganiriza ku bijyanye nimihindagurikire yumubiri wacu bakanatubwira nyine uko twakagombye kubyitwaramo.

Title: Mulinga Girls transcribed.docx

Doc Creator: tcuhawenimana11

Doc Date: 3/25/2023

Codes Applied: Information that girls need

Linked Memos: 0

Excerpt Creator: tcuhawenimana11

Excerpt Created On: 4/28/2023

Excerpt Range: 19173-19855

Murakoze, nimero yange ni 03 mfite imyaka 17 niga muwa 3. Ngewe amakuru numva umwana w’umukobwa yagakwiriye guhabwa ni nko kuba yaganirizwa kubuzima bujyanye no kujya mu mihango yaba abavandimwe be ndetse nababyeyi be bakabimufashamo kugirango abashe kubibamo kd abibemo ntabwoba bimuteye ntanikiniga bimuteye cyangwa isoni mu bandi. Ubwo ngubwo rero indi nama cyangwa se ibindi umuntu yakwifashisha kuba umubyeyi wawe yakugira inama zo kugira inshuti nziza akareka kukugira inama yakuroha kuko nyine uwonguwo aba arumubyeyi wawe cg umuvandimwe wawe, aba agomba kukugira inama zatuma ugira ahazaza heza hatangiritse cg ngo hononwe nibyo tubona hanze ahangaha bibi byanduye murakoze.

Title: Mulinga Girls transcribed.docx

Doc Creator: tcuhawenimana11

Doc Date: 3/25/2023

Codes Applied: Information that girls need Suggestions to improve SRH during puberty and adolescence by girls

Linked Memos: 0

Excerpt Creator: tcuhawenimana11

Excerpt Created On: 4/28/2023

Excerpt Range: 19893-20590

Murakoze, nimero yange ni 02 mfite imyaka 14 niga senior 2. Ndumva inama umwana wumwangavu yagirwa wenda mu mashuri hari ukuntu umwarimu yaza akatuganiriza aktubwira ngo gukora imibonanompuzabitsina ntabwo aribyo kuko duhuriramo nibibazo byinshi tukaba twakwandura indwara zandurira mu mibonanompuzabitsina cg tukaba twacikisha amashuri tukiri bato tugatwara ninda ugasanga tugoye abarwayi bacu bitari bikwiye bakatubwira ngo ntabwo mugombye kugenda ijoro kuko ntabwo aba aribyo cg ngo umubyeyi akohereze kwishuri kwiga ngo wowe ahubwo ureke kwiga ngo uhite wikatira mu zindi nzira niyo nama numvaga twebwe nkabana babakobwa tuba dukwiye kugirwa kugirango tubashe kumenya uko ubuzima bwacu bumeze.

Title: Mulinga Girls transcribed.docx

Doc Creator: tcuhawenimana11

Doc Date: 3/25/2023

Codes Applied: Information that girls need

Linked Memos: 0

Excerpt Creator: tcuhawenimana11

Excerpt Created On: 4/28/2023

Excerpt Range: 20783-21058

Murakoze ndi nimero 07 niga muwa 3. Numva amakuru twakagombye kubera numva ababyeyi bakagomye kutwitaho ako si ababyeyi gusa nabandi bantu babitwigisha kandi tukabifata tubishatse bakatwigisha kwirinda akavuyo, imico mibi nkubusambanyi, kugenda ijoro ndetse nibindi murakoze.

Title: Mulinga Girls transcribed.docx

Doc Creator: tcuhawenimana11

Doc Date: 3/25/2023

Codes Applied: Information that girls need Suggestions to improve SRH during puberty and adolescence by girls

Linked Memos: 0

Excerpt Creator: tcuhawenimana11

Excerpt Created On: 4/28/2023

Excerpt Range: 21111-21828

Murakoze, nimero yange ni 08 niga senior 3. Inama numva twagirwa nkabakobwa nyine bageze mu gihe cy’ubwangavu nuko ababyeyi bacu batubwira yuko wenda igihe umukobwa yageze mu gihe cy’ubwangavu yagiye mu mihango bakatubwira yuko wenda ashobora kuba yasama inda, bakatubwira yuko kuba twakiga ingeso mbi zo kuba twahura nabahungu cyangwa abandi bose baturuta badushuka yuko Atari byiza. Kandi ahandi twakura inama no ku baganga hari igihe batumaho urubyiruko tukagenda bakatugira inama zibyo byose nyine bijyanye nimyororokere ubwo ngubwo rero kubwizo mpamvu zose zigatuma twiga tukaba twanamenya nuko twagira inama abandi bagenzi bacu batabashije kugirwa inama nababyeyi cg ababarera cg kwamuganga batagiyeyo murakoze.

Title: Mulinga Girls transcribed.docx

Doc Creator: tcuhawenimana11

Doc Date: 3/25/2023

Codes Applied: Information that boys need Suggestions to improve SRH during puberty and adolescence by girls

Linked Memos: 0

Excerpt Creator: tcuhawenimana11

Excerpt Created On: 4/28/2023

Excerpt Range: 22179-23037

Murakoze ndi nimero 5 mfite imyaka 15 niga muwa 2. Nyine nange ku makuru yumuhungu nabo bagakwiye kwicarana nababyeyi babo batitaye ngo numugabo cyangwa se numugore cyangwa se nka gutya mwabikoze bakadushyiriraho gahunda yo kuzaza baza bakagira nkabantu nka bake bazana bakatwigisha, bakigisha batarobanuye ngo nabakobwa cg se nabahungu, bakegera no kubigo nderabuzima cyangwa se no kubigo byamashuri bagashyiraho abantu bazaza badusobanurira batwigisha batagendeye ngo numukobwa cyangwa se numuhungu murakoze Nko muri radiyo Rwanda hariho ikiganiro cya ninyampinda nacyo tujya tugikurikirana tukagikurikira tutitaye ngo nabakobwa cyangwa se nabahungu kuko hazaho umu mama bita shangazi nyine aratuganiriza akaganiriza abakobwa cyangwa se nabahungu ndetse no kuri kiss fm hari ikindi kibaho kitwa kumbi ibyo byose rero nabashishikariza kubikurikira murakoze.

Title: Mulinga Girls transcribed.docx

Doc Creator: tcuhawenimana11

Doc Date: 3/25/2023

Codes Applied: Information that boys need Suggestions to improve SRH during puberty and adolescence by girls

Linked Memos: 0

Excerpt Creator: tcuhawenimana11

Excerpt Created On: 4/28/2023

Excerpt Range: 23132-23703

Nimero yange ni 01 imyaka yange ni 17 niga muwa 3. Amakuru baba bakeneye, mbese kubijyanye nubuzima bwimyororokere ndetse naya mihindagurikire yubuzima, abenshi bavuga yuko iyo bavuga ngo ubuzima bwimyororokere bumva abakobwa, imihango ibintu nkibyo. Akenshi abahungu ntago baba bumva ko bibareba rero bareka kwiyumvisha iyo myumvire bakicarana nababyeyi babo ndetse nka kuriya bajya kwa muganga, bimwe bagenzi bange bavugaga, iyo myumvire ikavaho bakajya bajya kwa muganga bakabasobanurira bakababwira ngo igihe ugezemo ushobora gutera inda ibintu nkibyongibyo murakoze.

Title: Mulinga Girls transcribed.docx

Doc Creator: tcuhawenimana11

Doc Date: 3/25/2023

Codes Applied: Information that boys need

Linked Memos: 0

Excerpt Creator: tcuhawenimana11

Excerpt Created On: 4/28/2023

Excerpt Range: 23884-24529

Murakoze nimero yange ni 06 mfite imyaka 14 niga muwa 2 secondary. Abahungu bagakwiye kwicarana nababyeyi babo bakabagira inama bakabatega amatwi. Akenshi abahungu bavuga ko ubundi bakunda kwirara bakumva abakobwa nibo bakaganiriye nababyeyi babo kd sibyo, bagakwiye gutega amatwi radiyo bakumva ibiganiro bicaho, bakumva inama zababyeyi babo, cyangwa se nibyo kwa muganga nabo bajyayo bakabagira inama nge niko mbyumva murakoze. Inama ku makuru ajyanye niki? Tubwire; Inama ku makuru ajyanye kubuzima by’imyororokere yabo nimihindagurikire yimibiri yabo bakabaganiriza bakababwira ati rero ndumva babaganiriza no kubintu bijyanye nimyororokere.

Title: Mulinga Girls transcribed.docx

Doc Creator: tcuhawenimana11

Doc Date: 3/25/2023

Codes Applied: Suggestions to improve SRH during puberty and adolescence by girls

Linked Memos: 0

Excerpt Creator: tcuhawenimana11

Excerpt Created On: 4/28/2023

Excerpt Range: 23884-24312

Murakoze nimero yange ni 06 mfite imyaka 14 niga muwa 2 secondary. Abahungu bagakwiye kwicarana nababyeyi babo bakabagira inama bakabatega amatwi. Akenshi abahungu bavuga ko ubundi bakunda kwirara bakumva abakobwa nibo bakaganiriye nababyeyi babo kd sibyo, bagakwiye gutega amatwi radiyo bakumva ibiganiro bicaho, bakumva inama zababyeyi babo, cyangwa se nibyo kwa muganga nabo bajyayo bakabagira inama nge niko mbyumva murakoze

Title: Mulinga Girls transcribed.docx

Doc Creator: tcuhawenimana11

Doc Date: 3/25/2023

Codes Applied: Information that boys need

Linked Memos: 0

Excerpt Creator: tcuhawenimana11

Excerpt Created On: 4/28/2023

Excerpt Range: 24717-25429

Murakoze, ndi nimero 07 mfite imyaka 18 niga senior 3. Kukijyanye namakuru ku buzima bwimyororokere hari igihe usanga umuhungu yirara akavuga ati reka reka ibi bintu sinabikora sinajya kuganira nababyeyi bambwira biriya abakobwa nibo bireba kubera ko nibo bagira ibibazo byinshi naho kubera ko ngewe musore kuko ntakintu bimbwiye rwose ntabwo nakwicarana numukobwa ngo bangire inama, akumva ko nyine ari umugabo ntakintu bimubwiye kwicarana nabakobwa ngo bamugire inama rero bagakwiye gucisha make bakita ku nama babagira yaba ababyeyi nababahungu wenda bagendana. Hari igihe waba ugendana ninshuti zawe arko zikugira inama arko wowe ukumva ntakintu bikubwiye kuko wumva biba bigomba mushiki wawe byose murakoze.

Title: Mulinga Girls transcribed.docx

Doc Creator: tcuhawenimana11

Doc Date: 3/25/2023

Codes Applied: Sources of information received during puberty

Linked Memos: 0

Excerpt Creator: tcuhawenimana11

Excerpt Created On: 4/28/2023

Excerpt Range: 25784-26136

Murakoze nimero yange ni 04 imyaka ni 16 niga senior 3. Amakuru abantu bagiye bampa ku kigo nigaho cg ababyeyi bange cg nabandi bose ni ukwitwara neza, kwirinda ingeso mbi, kwirinda inshuti mbi ubona ntacyo yakugezaho yagushora mu ngeso mbi, kwirinda kugenda ijoro, kwirinda cyane cyane igikundi cyabantu batari beza no kwrinda ibiyobyabwenge murakoze.

Title: Mulinga Girls transcribed.docx

Doc Creator: tcuhawenimana11

Doc Date: 3/25/2023

Codes Applied: Types of information provided to boys and girls during puberty

Linked Memos: 0

Excerpt Creator: tcuhawenimana11

Excerpt Created On: 4/28/2023

Excerpt Range: 25917-26136

ni ukwitwara neza, kwirinda ingeso mbi, kwirinda inshuti mbi ubona ntacyo yakugezaho yagushora mu ngeso mbi, kwirinda kugenda ijoro, kwirinda cyane cyane igikundi cyabantu batari beza no kwrinda ibiyobyabwenge murakoze.

Title: Mulinga Girls transcribed.docx

Doc Creator: tcuhawenimana11

Doc Date: 3/25/2023

Codes Applied: Sources of information received during puberty Types of information provided to boys and girls during puberty

Linked Memos: 0

Excerpt Creator: tcuhawenimana11

Excerpt Created On: 4/28/2023

Excerpt Range: 26301-26492

Murakoze, nimero yange ni 01 imyaka ni 17 niga muwa 3. Amakuru naba naragiye mpabwa cg mbwirwa ku buzima bwimyororokere mu gihe cyubigimbi cg ubwangavu navuga wenda nko kuvuga ku babyeyi bacu

Title: Mulinga Girls transcribed.docx

Doc Creator: tcuhawenimana11

Doc Date: 3/25/2023

Codes Applied: Types of information provided to boys and girls during puberty

Linked Memos: 0

Excerpt Creator: tcuhawenimana11

Excerpt Created On: 4/28/2023

Excerpt Range: 26301-27047

Murakoze, nimero yange ni 01 imyaka ni 17 niga muwa 3. Amakuru naba naragiye mpabwa cg mbwirwa ku buzima bwimyororokere mu gihe cyubigimbi cg ubwangavu navuga wenda nko kuvuga ku babyeyi bacu hari igihe batubwira ati ibyo bintu urimo ibi nibi, nkurugero hari igihe ushobora kugenda ugahura wenda ninshuti mukaba mwacataho ugataha nka saa moya cg saa kumi nebyiri wagera murugo bati eeee urabona ko utangiye gukora ibyongibyo gutaha ijoro uzaboneramo ibibi ibintu nkibyongibyo basaza bacu bakagenda batugira inama irinde ibigushuka nkaho kwa muganga wajyayo ibintu nkibyo nyine nugera mu mihango uzabyitwaremo gutya, abahungu nabo bakaba bababwira ati hazabaho wenda nko kwiroteraho ibintu nkibyo bidafusha wenda kuba tuzabigeramo tubizi murakoze.

Title: Mulinga Girls transcribed.docx

Doc Creator: tcuhawenimana11

Doc Date: 3/25/2023

Codes Applied: Sources of information received during puberty Types of information provided to boys and girls during puberty

Linked Memos: 0

Excerpt Creator: tcuhawenimana11

Excerpt Created On: 4/28/2023

Excerpt Range: 27105-27252

Murakoze, nimero yange ni 03 mfite imyaka 16 niga muwa 3. Amakuru naba narahawe kubijyanye nubuzima bwimyororokere nuko ababyeyi bange nkiri umwana

Title: Mulinga Girls transcribed.docx

Doc Creator: tcuhawenimana11

Doc Date: 3/25/2023

Codes Applied: Types of information provided to boys and girls during puberty

Linked Memos: 0

Excerpt Creator: tcuhawenimana11

Excerpt Created On: 4/28/2023

Excerpt Range: 27105-27591

Murakoze, nimero yange ni 03 mfite imyaka 16 niga muwa 3. Amakuru naba narahawe kubijyanye nubuzima bwimyororokere nuko ababyeyi bange nkiri umwana bakundaga kumbwira ikintu kijyanye no kujya mu mihango ndetse we nabavandimwe bange babifatanyije. Nyine bakagenda banyigisha nibimbaho uko nzabyitwaramo kuburyo byagiye kungeeraho mbisobanukiwe neza bityo rero numva ko ababyeyi bose bagakwiye kumenya ko yaba abana babakobwa cg abahungu bose bakeneye kigirwa inama muribyo bihe murakoze.

Title: Mulinga Girls transcribed.docx

Doc Creator: tcuhawenimana11

Doc Date: 3/25/2023

Codes Applied: Types of information provided to boys and girls during puberty

Linked Memos: 0

Excerpt Creator: tcuhawenimana11

Excerpt Created On: 4/28/2023

Excerpt Range: 27648-28355

Murakoze nimero yange ni 07 mfite imyaka 18 niga senior 3. Amakuru menshi nagiye numva ajyanye nubwangavu cg nubugimbi akenshi cyane... impinduka ijyanye nubuzima bwimyororokere, impinduka zijyanye nimihindagurikire yo mu gihe cy’ubugimbi cyangwa ubwangavu. Impinduka ubundi mbere narwaye ibiheri mu maso barabwira ngo ubundi ibintu bikiza ibiheri nugukora imibonano mpuzabitsina utayikoze ubwo ntabwo byakira nyine uzashaka ukuntu wayikora urebe ko byakira cg ntutayikora uzagumya urware ibiheri wenda nyine nuko ako numva ko atariyo nzira nyine kandi numva abahungu bakiri abana bakura ati bahinduye amajwi nyine barakuze ibintu nkibyo ukumva impinduka nyinshi abantu bagenda bavuga bitandukanye murakoze.

Title: Mulinga Girls transcribed.docx

Doc Creator: tcuhawenimana11

Doc Date: 3/25/2023

Codes Applied: Sources of information received during puberty Types of information provided to boys and girls during puberty

Linked Memos: 0

Excerpt Creator: tcuhawenimana11

Excerpt Created On: 4/28/2023

Excerpt Range: 28557-28689

Nimero yange ni 08 mfite imyaka 17 niga senior 3. Amakuru nahawe ajyanye nimyororokere ku bwangavu cg ku bugimbi nuko ababyeyi bange

Title: Mulinga Girls transcribed.docx

Doc Creator: tcuhawenimana11

Doc Date: 3/25/2023

Codes Applied: Types of information provided to boys and girls during puberty

Linked Memos: 0

Excerpt Creator: tcuhawenimana11

Excerpt Created On: 4/28/2023

Excerpt Range: 28557-29514

Nimero yange ni 08 mfite imyaka 17 niga senior 3. Amakuru nahawe ajyanye nimyororokere ku bwangavu cg ku bugimbi nuko ababyeyi bange bakundanga kumbwira iyo wenda uri umukobwa ukagera muri icyo gihe watangiye kumera amabere, kujya mu mihango, kuzana amataye ushobora nko guca ku bantu abasore cg wenda abagabo bakakubwira ati mbega ukuntu uteye neza ati ndabona usa neza cyane wenda nawe muricyo gihe ugakururwa akakubwira ngo zana nkoreho numve noneho warangiza ukajya ushiduka nkabo bakaba bagushukisha ayo mafaranga cg telephone akakubwira ati najya nkubonera namavuta meza noneho bitewe nuko ntanama uba warahawe ibyo bintu ukabyakira ugasanga umugabo aguteye inda cg aguteye izindi ndwara aguteye sida ugasanga amashuri yawe ntabwo uyize bityo rero ababyeyi bange babingiriyeho inama kugeza ndakeka ngize imyaka 12 aribwo wenda natangiye kujya mu mihango izo nama rero kuba narazikurikije nizo zitumye ngera ahangaha ngeze, ubwo ayo niyo makuru nahawe.

Title: Mulinga Girls transcribed.docx

Doc Creator: tcuhawenimana11

Doc Date: 3/25/2023

Codes Applied: Sources of information received during puberty

Linked Memos: 0

Excerpt Creator: tcuhawenimana11

Excerpt Created On: 4/28/2023

Excerpt Range: 29564-29826

Murakoze nimero yange ni 06 mfite imyaka 12 niga senior 1. Amakuru nagiye mpabwa ababyeyi bange, abavandimwe bange nabandi bantu muri rusange yaba abo duturanye yaba abo tudaturanye, yeah abantu bose nyine banzi cg se yaba ntabatanzi bose bagendaga bangira inama

Title: Mulinga Girls transcribed.docx

Doc Creator: tcuhawenimana11

Doc Date: 3/25/2023

Codes Applied: Types of information provided to boys and girls during puberty

Linked Memos: 0

Excerpt Creator: tcuhawenimana11

Excerpt Created On: 4/28/2023

Excerpt Range: 29827-30137

bakambwira ngo ujye wirinda kugenda ijoro ibibi byose akenshi ngo bikorwa mwijoro bakangira inama bakabwira ati iyo ugeze muriki kigero waratangiye kujya mu mihango ugira uko witwara singombwa ko buri muntu wese wakubwira ngo ibi nibi ngo bimpe ntabwo ari ngombwa ko wabimuha ntanubwo biba byemewe ko wabimuha.

Title: Mulinga Girls transcribed.docx

Doc Creator: tcuhawenimana11

Doc Date: 3/25/2023

Codes Applied: Sources of information received during puberty

Linked Memos: 0

Excerpt Creator: tcuhawenimana11

Excerpt Created On: 4/28/2023

Excerpt Range: 30362-30617

Murakoze nge ayo makuru navanye mubabyeyi, muri bakuru bange, kuri radio zimwe na zimwe ngenda numva, ku bantu nyine baza kutuganiriza nkuku nguku, abarimu baturera ibyongibyo byose rero niho nagiye mbikura. Vuga nimero. Nomero 5 imyaka ni 15 niga muwa 2.

Title: Mulinga Girls transcribed.docx

Doc Creator: tcuhawenimana11

Doc Date: 3/25/2023

Codes Applied: Sources of information received during puberty

Linked Memos: 0

Excerpt Creator: tcuhawenimana11

Excerpt Created On: 4/28/2023

Excerpt Range: 30668-31016

Murakoze nimero yange ni 02 mfite imyaka 14 niga senior 2. Amakuru yubuzima bwimyororokere nagiye nyakura nko ku nshuti zange twigana, abavandimwe, ku bantu nyine bababyeyi bagiye bandera, ugasanga nyine kuri televiziyo barabivugaho, ku maradiyo ahantu henshi bitewe nikiganiro bariho ku buzima bwimyororokere nabyumva nkagira ibyo nigamo murakoze.

Title: Mulinga Girls transcribed.docx

Doc Creator: tcuhawenimana11

Doc Date: 3/25/2023

Codes Applied: Sources of information received during puberty

Linked Memos: 0

Excerpt Creator: tcuhawenimana11

Excerpt Created On: 4/28/2023

Excerpt Range: 31061-31318

Murakoze nimero yange ni 01 imyaka ni 17 niga muwa 3. Amakuru ahantu naba narayavanye wenda mu barimu abarezi baturera, abaje kudusura nko ku mashuri yacu, ababyeyi bacu ndetse rimwe na rimwe naba naherekeje nababyeyi bange kwa muganga ibintu nkibyo ngibyo.

Title: Mulinga Girls transcribed.docx

Doc Creator: tcuhawenimana11

Doc Date: 3/25/2023

Codes Applied: Sources of information received during puberty

Linked Memos: 0

Excerpt Creator: tcuhawenimana11

Excerpt Created On: 4/28/2023

Excerpt Range: 31363-31603

Murakoze nimero yange ni 06 imyaka 12 niga senior 1. Amakuru ajyane nubuzima bwimyororokere nagiye nyavana mu nshuti zange, ku babyeyi, abavandimwe, abarezi baturera, ababa baje kudusura wenda nko mu gihe tuba twiga ndetse nahandi murakoze.

Title: Mulinga Girls transcribed.docx

Doc Creator: tcuhawenimana11

Doc Date: 3/25/2023

Codes Applied: Sources of information received during puberty

Linked Memos: 0

Excerpt Creator: tcuhawenimana11

Excerpt Created On: 4/28/2023

Excerpt Range: 31648-32040

Murakoze nimero yange ni 08 mfite imyaka 17 nkaba niga muwa 3. Amakuru ahantu nayakuye bwa mbere ntabwo wenda nayakuye mu babyeyi bange kuko nakuze mbana numukecuru rero hari umugoroba wurubyiruko wajyaga uba kwa muganga noneho nkajya njyayo buri saa munani bakatugira inama nyine ku buzima bwimyororokere bakaduha nudutabo tugasoma no kwishuri nkabyumva nababyeyi aho niho nayakuye murakoze.

Title: Mulinga Girls transcribed.docx

Doc Creator: tcuhawenimana11

Doc Date: 3/25/2023

Codes Applied: Sources of information received during puberty

Linked Memos: 0

Excerpt Creator: tcuhawenimana11

Excerpt Created On: 4/28/2023

Excerpt Range: 32081-32401

Nimero yange 07 imyaka yange ni 18 niga senior 3. Bwa mbere nge nyimenya ayo makuru bakuru bange nibo bayampaga cyange kuebra ko mama ntabwo yakundaga kuba ahari yabaga yagiye nko mu kazi, bakuru bange mbere nibo bayampaga inshuti nabavvandimwe gutyo gutyoarko mbere nayakuraga kuri bakuru bange nibo bayampaga murakoze.

Title: Mulinga Girls transcribed.docx

Doc Creator: tcuhawenimana11

Doc Date: 3/25/2023

Codes Applied: Obstacles and risks experienced by boys during the sexual and reproductive health changes occuring during puberty and adolescence

Linked Memos: 0

Excerpt Creator: tcuhawenimana11

Excerpt Created On: 4/28/2023

Excerpt Range: 32850-33405

Murakoze nimero ni 02 imyaka ni 14 niga senior 2. Ingorane abana babahungu bahura nazo bitwaye nabi ashobora nko gutera umukobwa inda atarageza igihe bakamufunga cyangwa mu gihe batanamufashe akaba yatoroka akava nkiwabo akajya nkahandi hantu akabaho yihisha akagira ubuzima bubi bwo kwimenyera buri kimwe kubera ko yakoze ikosa cg bikaba ngombwa yuko kwiga abireka bakamutegeka yuko agomba gufasha uwo mukobwa, mugihe agomba kumufasha ubwo bikaba ngombwa ko agomba kureka ishuri akajya ajya gushaka amafaranga yo gufasha uwo mukobwa yateye inda murakoze.

Title: Mulinga Girls transcribed.docx

Doc Creator: tcuhawenimana11

Doc Date: 3/25/2023

Codes Applied: Obstacles and risks experienced by boys during the sexual and reproductive health changes occuring during puberty and adolescence

Linked Memos: 0

Excerpt Creator: tcuhawenimana11

Excerpt Created On: 4/28/2023

Excerpt Range: 33559-33964

Murakoze nimero yange ni 5 imyaka ni 15 niga muwa 2. Ingorane abahungu numva bahura nazo rimwe na rimwe iyo batumvwa nyine nababyeyi ntibabashe kuba babaganiriza ibyo nabyo byatuma bagira inzitizi zitari nziza. Inzitizi zitari nziza ni igihe iyo atumvishwe ngo abashe kuganirizwa agira kuba yatera umukobwa inda cg se akagenda akajya mu mico mibi akajya kunywa ibiyobyabwenge bigatuma ata ishuri murakoze.

Title: Mulinga Girls transcribed.docx

Doc Creator: tcuhawenimana11

Doc Date: 3/25/2023

Codes Applied: Obstacles and risks experienced by boys during the sexual and reproductive health changes occuring during puberty and adolescence

Linked Memos: 0

Excerpt Creator: tcuhawenimana11

Excerpt Created On: 4/28/2023

Excerpt Range: 34117-34664

Murakoze nimero yange ni 02 mfite imyaka 18 niga senior 3. Ingorane cg inzitizi zikunze kubaho ku bana babahungu hari igihe umuhungu aba atagejeje imyaka yo gushaka ariko muri we akumva yashaka bitewe nukuntu abona umukobwa amukunze akumva wenda yakwihutira gushaka igihe kitageze namashuri yewe atayashoje akaba yumva yashaka cg akaba yumva yamutera inda cg bagakora nkikosa akamutera inda hakabaho ibyo bintu byo kuvuga ntiyabona uko afasha umukobwa yateye inda, ese aziga ate? Ese azubaka he? Agatekereza ibintu nkibyo kd akiri umwana murakoze.

Title: Mulinga Girls transcribed.docx

Doc Creator: tcuhawenimana11

Doc Date: 3/25/2023

Codes Applied: Obstacles and risks experienced by boys during the sexual and reproductive health changes occuring during puberty and adolescence

Linked Memos: 0

Excerpt Creator: tcuhawenimana11

Excerpt Created On: 4/28/2023

Excerpt Range: 34699-35069

Ingorane mbona mu buzima bbwanaba babahungu bageze mu gihe cyubugimbi hari nkukuntu umwana wumuhungu ahura nundi muhungu mugenzi we akamushuka ngo bajye mu biyobyabwenge hari naho hazava kwa kunanirana bakajya murabo bakobwa babana bu Rwanda bakabangiza ugasanga babateye inda ugasanga batanabizi cg nabo arinabana batabasha kubafasha nyine inzitizi nizo ngizo murakoze.

Title: Mulinga Girls transcribed.docx

Doc Creator: tcuhawenimana11

Doc Date: 3/25/2023

Codes Applied: Obstacles and risks experienced by boys during the sexual and reproductive health changes occuring during puberty and adolescence

Linked Memos: 0

Excerpt Creator: tcuhawenimana11

Excerpt Created On: 4/28/2023

Excerpt Range: 35210-35785

Murakoze nimero yange ni 01 imyyaka ni 17 niga muwa 3. Ingorane abahungu numva bahura nazo mu buzima mu gihe bageze muri kiriya gihe ndumva wenda hari nkukuntu ababyeyi bakubwira ati ibi bintu ntago aribyo, nka kwakundi twavugaga mbere wenda bakakubwira oya ibi ni ibyabakobwa gusa ntago nge nabireba reka reka ngo nge kwicarana na mama muri salon ngo barangira inama? Wapi ntago byakunda ukaba wakwandura indwara zimwe na zimwe sida ibintu nkibyo, imitezi bimwe na bimwe bikaba byakugora, noneho byabindi wangaga kugirwa inama ahubwo ukazigirwa unameze nabi urwaye murakoze.

Title: Mulinga Girls transcribed.docx

Doc Creator: tcuhawenimana11

Doc Date: 3/25/2023

Codes Applied: Obstacles and risks experienced by girls during the sexual and reproductive health changes occuring during puberty and adolescence

Linked Memos: 0

Excerpt Creator: tcuhawenimana11

Excerpt Created On: 4/28/2023

Excerpt Range: 36120-36765

Murakoze, mu gihe cyabangavu iyo ugirwa inama kenshi ntuzumve bakubwira ati wigenda ijoro ukanga kumva, bakubwira ngo ntugashukishwe ibintu byose ujye unyurwa nibyo mu rugo bafite ukanga kumva hari igihe utwara inda ugacikisha amashuri kandi ugasanga wowe ni wowe bigizeho ingaruka ndende ugasanga wenda wowe ucikishije amashuri arko umuhungu we akomeje kwiga cg ugasanga wanduye indwara zandurira mu mibonanompuzabitsina nka sida, imitezi nizindi ndwara nyinshi cg ugasanga utwaye inda bitewe nuko iwanyu bakubwira ngo ntuzayizane hano ugasanga habayeho ingaruka zuko ushobora kuyikuramo bikakuviramo ubundi burwayi ukica umwana cg nawe ugapfa.

Title: Mulinga Girls transcribed.docx

Doc Creator: tcuhawenimana11

Doc Date: 3/25/2023

Codes Applied: Obstacles and risks experienced by girls during the sexual and reproductive health changes occuring during puberty and adolescence

Linked Memos: 0

Excerpt Creator: tcuhawenimana11

Excerpt Created On: 4/28/2023

Excerpt Range: 36785-37711

Murakoze ndi nimero 08 mfite imyaka 17 niga senior 3. Ingorane numva abana babakobwa twahura nazo hari ukuntu wenda ntago twese tuba dufite imiryango yifashije kuburyo baduha ibyo dukeneye byose. Wenda nkabona nka mugenzi wange kwishuri ahora azana inkweto azihinduranya, agahora mbona asa neza nkamubaza ngo ese ayo mavuta wowe uyakura he? Ese kuki uhora wambaye gutyo ubigenza ute? Akambwira uti wowe ntabyo uzi ati hari abantu muriki gihe basigaye batanga amafaranga ati wowe umbwire nkurangire ati hari ikigabo (babyita gukura) ati hari ikibago ushobora kuba wakura ukabona I pantaro nziza, inkweto nziza nkuko ubyifuza. Bitewe nange no kutumva cg gushidukira ibyongibyo kugira nange nse neza nkuwonguwo nange nkaba nishoye muri bya bindi nsanze wamugabo ubwo ngubwo akaba wenda anteye sida, anteye inda ugasanga byabundi byose naririraga cg se no gusa neza ugasanga byose bipfuye ubusa, ndumva iyo ariyo ngorane murakoze.

Title: Mulinga Girls transcribed.docx

Doc Creator: tcuhawenimana11

Doc Date: 3/25/2023

Codes Applied: Obstacles and risks experienced by girls during the sexual and reproductive health changes occuring during puberty and adolescence

Linked Memos: 0

Excerpt Creator: tcuhawenimana11

Excerpt Created On: 4/28/2023

Excerpt Range: 37877-38254

Murakoze ndi nomero 04 imyaka ni 16 niga senior 3. Ingorane numva umwana wumukobwa yahura nazo mu gihe cyubwangavu nukwanga kumva inama nziza agirwa nababyeyi cg ninshuti akishora mu ngeso mbi zirimo izubusambanyi, ibiyobyabwenge nibindi bintu bibi bigeye bitandukanye nko gushukwa nabo ba sugardaddy akenshi na kenshi biterwa no kutanyurwa nuko ubayeho cg ibyo ufite murakoze.

Title: Mulinga Girls transcribed.docx

Doc Creator: tcuhawenimana11

Doc Date: 3/25/2023

Codes Applied: Barriers leading to the risks and obstacles girls and boys experience during puberty and adolescence

Linked Memos: 0

Excerpt Creator: tcuhawenimana11

Excerpt Created On: 4/28/2023

Excerpt Range: 37877-38254

Murakoze ndi nomero 04 imyaka ni 16 niga senior 3. Ingorane numva umwana wumukobwa yahura nazo mu gihe cyubwangavu nukwanga kumva inama nziza agirwa nababyeyi cg ninshuti akishora mu ngeso mbi zirimo izubusambanyi, ibiyobyabwenge nibindi bintu bibi bigeye bitandukanye nko gushukwa nabo ba sugardaddy akenshi na kenshi biterwa no kutanyurwa nuko ubayeho cg ibyo ufite murakoze.

Title: Mulinga Girls transcribed.docx

Doc Creator: tcuhawenimana11

Doc Date: 3/25/2023

Codes Applied: Obstacles and risks experienced by girls during the sexual and reproductive health changes occuring during puberty and adolescence

Linked Memos: 0

Excerpt Creator: tcuhawenimana11

Excerpt Created On: 4/28/2023

Excerpt Range: 38319-38836

Murakoze nimero ni 01 imyaka ni 17 niga muwa 3. Ingorane numva umwana wumukobwa yahura nazo mu gihe atumviye cg rimwe na rimwe atagendeye ku nama bamugira hari nko kuba wenda wakwandura nkizo ndwara bagenzi bange bavuze, ikindi hari ukubaho wicuza ubuzima bwawe bwose ukiyicira future yawe izaza. Urugero wenda utaye inda, twese ntago twifashije nkuko bose babivuze, hanyuma urayitwaye iwanyu ntibifashije uwo mwana ntufite ikizamurera ugasanaga abayeho nabi nawe ubayeho nabi kubera byose bitewe no kutumva murakoze.

Title: Mulinga Girls transcribed.docx

Doc Creator: tcuhawenimana11

Doc Date: 3/25/2023

Codes Applied: Obstacles and risks experienced by girls during the sexual and reproductive health changes occuring during puberty and adolescence

Linked Memos: 0

Excerpt Creator: tcuhawenimana11

Excerpt Created On: 4/28/2023

Excerpt Range: 39151-39714

Murakoze nomero ni 02 imya ni 14 niga senior 2. Ngewe uko mbyumva inzitizi umwana wumukobwa yagira kuburyo atabasha kwitwara nkuko bikwiriye ushobora kuba ufite ababyeyi babi batagufasha ukaba wabaka ibikoresha bakabikwima wenda hakaba hari umuntu akakugira inama mbi ati wenda ujye ugenda usambane wenda bazajya babiguha bigatuma agenda gukora imibonanompuzabitsina bikamuviramo ingaruka kugirango abone ibyo bikoresho yagakwiye kubona arko wenda bitewe nuko abyumva bigatuma akora ibitagakwiye gukorwa bitewe nababyeyi be batamuhaye ibyo akeneye byose murakoze.

Title: Mulinga Girls transcribed.docx

Doc Creator: tcuhawenimana11

Doc Date: 3/25/2023

Codes Applied: Barriers leading to the risks and obstacles girls and boys experience during puberty and adolescence

Linked Memos: 0

Excerpt Creator: tcuhawenimana11

Excerpt Created On: 4/28/2023

Excerpt Range: 39915-40691

Murakoze nimero yange ni 01 imyaka ni 17 niga muwa 3. Impamvu wenda numva yaba ibitera imiryango imwe nimwe ntago iba yifashije arko nanone kutifasha ntago ariko kujya muribyi ngibyo akenshi ahubwo ntago tunyurwa ugasanga uko ubayeho ntago unyurwa nuko uri, urabona wanamugani umwana ahinduranya inkweto buri munsi uti kuki se nge ntaba kuriya? Kuki se nge batandeba? Nage reka nge muri biriya. Ikindi numva cyaba kibitera akenshi ntago ababyeyi bacu baduha inaama nkuko bikwiye, hari ababyeyi batinya bati uyu mwana wange buriya nzajya ku mwicaza iyo myumvire rero igatuma abana babo bangirika reka mbigemo nubundi mama ntakintu yigeze ambwira papa ntakintu yigeze ambwira, iyo icyo gihe uhuye nabyo ntago byaba ari impamvu zawe akenshi nabo baba babifitemo uruhare murakoze.

Title: Mulinga Girls transcribed.docx

Doc Creator: tcuhawenimana11

Doc Date: 3/25/2023

Codes Applied: Barriers leading to the risks and obstacles girls and boys experience during puberty and adolescence

Linked Memos: 0

Excerpt Creator: tcuhawenimana11

Excerpt Created On: 4/28/2023

Excerpt Range: 40745-41206

Murakoze nimero yange ni 06 mfite imyaka 14 niga muwa 2 secondary. Ndumva inzitizi zaba ku mwana wumukobwa akenshi ababyeyi ntabwo baguha inama nkuko bikwiye, ntibatuganiriza cyane usanga ahri ababyeyi batinya abana babo bakavuga ngo nzamuhera he, ubuse reba uko angana asigaye anduta ubuse nzaherahe mwicaza ngo mubwire ku bijyanye nubuzima bwimyororokere ugasanga ababyeyi akenshi ntago batwitaho uko bikwiye ngo batugire nizo nama nge niko mbyumva murakoze.

Title: Mulinga Girls transcribed.docx

Doc Creator: tcuhawenimana11

Doc Date: 3/25/2023

Codes Applied: Barriers leading to the risks and obstacles girls and boys experience during puberty and adolescence

Linked Memos: 0

Excerpt Creator: tcuhawenimana11

Excerpt Created On: 4/28/2023

Excerpt Range: 41256-41732

Murakoze nimero yange ni 03 mfite imyaka 17 niga muwa 3. Rero ngewe inzitizi numva nukugira inshuti mbi no kugendera mu kigare. Iyo ufite inshuti mbi iragushuka, inshuti mbi ikwereka uburyo nimba we azi ubwo buzima bwo kuba abayeho agenda ntakintu akoreye iwabo, agenda murizo ngeso mbi zose akumva ko ninshuti ariko yagakwiriye kumera rero ibyongibyo nibyo bizavamo ingaruka zo kubaho nabi mu gihe kizaza hakangirika ugasanga ibintu byose bibaye uko utabitekerezaga murakoze.

Title: Mulinga Girls transcribed.docx

Doc Creator: tcuhawenimana11

Doc Date: 3/25/2023

Codes Applied: Barriers leading to the risks and obstacles girls and boys experience during puberty and adolescence

Linked Memos: 0

Excerpt Creator: tcuhawenimana11

Excerpt Created On: 4/28/2023

Excerpt Range: 41764-42181

Murakoze nimero yange ni 07 mfite imyaka 18 niga senior 3. Inzitizi nge numva akenshi ku ngimbi cg kubangavu arukutanyurwa nibyo bafite cg ubuzima babayeho nkumukobwa akaba yumva yashaka amafaranga iwabo wenda bakaba bafite ubushobozi bwo kumuha ibihumbi bitanu ngo agure inkweto ako we akaba yumva yagura iyicumi kubera ko abona mugenzi we ariyo yambaye akaba yumva nyine yashaka kugenda nkawe kd bidakwiye murakoze.

Title: Mulinga Girls transcribed.docx

Doc Creator: tcuhawenimana11

Doc Date: 3/25/2023

Codes Applied: Suggestions to improve SRH during puberty and adolescence by girls

Linked Memos: 0

Excerpt Creator: tcuhawenimana11

Excerpt Created On: 4/28/2023

Excerpt Range: 42475-43039

Murakoze nomero yange ni 01 imyaka ni 17 niga muwa 3. Ikindi kintu numva nakwifuza kuvuga kubuzima bwimyororokere ku bakobwa cyangwa kubahungu nuko twakwitwara neza kubera yuko ntabwo tubikora akenshi tuzi ko ari bibi cyangwa mo kimwe tukaba tunabizi arko tukabikora tubizi gusa bikatwicira ejo haza gusa nkuko nabivugaga nkaba nifuza kuba umuganga arko kubera ko mbyariye mu rugo iwacu batifashije niyo buruse nari kuzabona nkakererwa ibintu nkibyongibyo rero turasabwa kwirinda cyane tukamenya ko ejo hazaza aritwe twahitegurira kd aritwe Rwanda rwejo murakoze.

Title: Mulinga Girls transcribed.docx

Doc Creator: tcuhawenimana11

Doc Date: 3/25/2023

Codes Applied: Suggestions to improve SRH during puberty and adolescence by girls

Linked Memos: 0

Excerpt Creator: tcuhawenimana11

Excerpt Created On: 4/28/2023

Excerpt Range: 43180-43405

Murakoze nomero yange ni 5 imyaka ni 15 niga muwa 2. Nange ikindi kintu numva navuga nuko bino byakomeza kubaho byo kutuganiriza kugirango nyine tubashe kunguka ubundi bwenge kubyo twari tuzi bizatume twigisha abacu murakoze.

Title: Mulinga Girls transcribed.docx

Doc Creator: tcuhawenimana11

Doc Date: 3/25/2023

Codes Applied: Suggestions to improve SRH during puberty and adolescence by girls

Linked Memos: 0

Excerpt Creator: tcuhawenimana11

Excerpt Created On: 4/28/2023

Excerpt Range: 43435-43756

Murakoze nimero yange ni 02 imyaka 14 senior 2. Numva yuko ngewe ikindi kintu nakongera aruko twagombye kwirinda tukanyurwa nibyo ababyeyi bacu baduha tukitabira ibiganira ku buzima bwimyororokere tukagerageza kumvira ababyeyi tukumva inama zose batugira kuko umubyeyi yarakubwiye ntago yapfa kukugira inama mbi murakoze.

Title: Mulinga Girls transcribed.docx

Doc Creator: tcuhawenimana11

Doc Date: 3/25/2023

Codes Applied: Precautions to take during puberty for girls

Linked Memos: 0

Excerpt Creator: tcuhawenimana11

Excerpt Created On: 4/28/2023

Excerpt Range: 43773-44099

Murakoze nomero yange ni 04 imyaka ni 16 niga senior 3. Nange icyo nakongeraho kubijyanye nubuzima bwimyororokere abana babakobwa baba bagomba kwitwara neza mu mpinduka bagira mumubiri wabo ziterwa nimihindagurikire yumubiri cg nimyaka bagezemo bakumvira inama za buri wese bumva ko ari nziza zibaganisha ahantu heza murakoze.

Title: Mulinga Girls transcribed.docx

Doc Creator: tcuhawenimana11

Doc Date: 3/25/2023

Codes Applied: Suggestions to improve SRH during puberty and adolescence by girls

Linked Memos: 0

Excerpt Creator: tcuhawenimana11

Excerpt Created On: 4/28/2023

Excerpt Range: 44203-44540

Murakoze nimero yange ni 06 imyaka ni 12 niga senior 1. Ikindi kintu nakumva navuga kubijyanye nubuzima bwimyororokere navuga ko iyi gahunda yo kuganiriza abana wenda, yo kuza kutuganiriza nkurubyiruka yakozmeza ikaza, bagakomeza bakatuganiriza, hakaza nababyeyi tukagerageza kumvira ababyeyi bacu tugakurikiza inama batubwira murakoze.

Title: Mulinga Girls transcribed.docx

Doc Creator: tcuhawenimana11

Doc Date: 3/25/2023

Codes Applied: Precautions to take during puberty for girls

Linked Memos: 0

Excerpt Creator: tcuhawenimana11

Excerpt Created On: 4/28/2023

Excerpt Range: 44564-45181

Murakoze nimero yange ni 08 mfite imyaka 17 niga senior 3. Ikindi kintu navuga kijyanye no kubuzima bwimyororokere nuko wenda ku mukobwa mu gihe naba ngeze mu gihe cyubwangavu ngomba gukurikiza inama zose mpabwa nababyeyi cg abandi bantu muri rusange noneho icyo navuga ku bahungu numuhungu ntiyumve yuko niba akuze akazana ayo matuza akagira niryo jwi ntago arukumva ko yaruse ababyeyi be ngo areke kubumviira ngo azajye agenda atahe ijoro hahandi yatahiye ijoro agahura nabo bantu bose inshuti mbi ni hahandi anyway ibiyobwabwenge bikaba byamuviramo gukora ingeso zose zitari nziza nge iyo niyo nama numva nabagira.

Title: Mulinga Girls transcribed.docx

Doc Creator: tcuhawenimana11

Doc Date: 3/25/2023

Codes Applied: Suggestions to improve SRH during puberty and adolescence by girls

Linked Memos: 0

Excerpt Creator: tcuhawenimana11

Excerpt Created On: 4/28/2023

Excerpt Range: 45196-45861

Inama nagira urubyiruko ku bijyanye nubuzima bwimyororokere nuko ababishinzwe badufasha bagashishikariza urubyiruko kwitabira gahunda zose zigisha ibijyanye nubuzima bwimyororokere. Nomero yange ni 03 mfite imyaka 16 niga muuwa 3.

Murakoze nimero yange ni 01 mfite imyaka 17 niga muwa 3. Nge ikintu nakongera kubyo nari navuze tugomba kumenya twe nkabakobwa cg nabahungu ntago ari abakobwa bonyine bashukwa nabahungu barashukwa, ubwo rero muriyi ya none ibishuko byabaye byinshi ubwo rero natwe nkabahungu cg abakobwa tugomba gutangira kwiga uburyo twabyitwaramo nkuko haduka ibindi bishuko byinshi natwe twadure ubundi buryo bwo kubyirindamo kuko ntabwo ari byiza.

Title: Mulinga Girls transcribed.docx

Doc Creator: tcuhawenimana11

Doc Date: 3/25/2023

Codes Applied: Precautions to take during puberty for girls

Linked Memos: 0

Excerpt Creator: tcuhawenimana11

Excerpt Created On: 4/28/2023

Excerpt Range: 46009-46374

Murakoze nimero yange ni 07 imyaka ni 18 niga senior 3. Nge ndumva ku kijyanye nubuzima bwimyororokere yaba ku ngimbi cyangwa ku bangavu nugukumira ikintu kitaraba ukumva amabwiriza usabwa kubuzima bwimyororokere ku mukobwa imihindagurikire yumubiri wawe ukayimenya no kumuhungu ukayimenya nyine bigufasha kugira ejo hazaza heza kd ukagira nubuzima buzima murakoze.

Title: Gs Munyiginya girls.docx

Doc Creator: tcuhawenimana11

Doc Date: 4/13/2023

Codes Applied: Physiological changes occuring during puberty-Boys

Linked Memos: 0

Excerpt Creator: tcuhawenimana11

Excerpt Created On: 4/28/2023

Excerpt Range: 508-858

(yego) murakoze, nshimishijwe nokuba muri iki gikorwa cyubushakashatsi, nimero yange ni kabiri (02), mfite imyaka 19, nkaba niga mumwaka wamashuri yisumbuye mumwaka wa gatunu (5).

Impinduka naba nzi k'umuhungu ugeze mu gihe cy'ubugimbi, Wenda navuga kwaguka mu igituza, kuniga ijwi ndetse no kumera ubucakwaha, ndetse nimisatsi kubice bye by'ibanga.

Title: Gs Munyiginya girls.docx

Doc Creator: tcuhawenimana11

Doc Date: 4/13/2023

Codes Applied: Physiological changes occuring during puberty-Girls

Linked Memos: 0

Excerpt Creator: tcuhawenimana11

Excerpt Created On: 4/28/2023

Excerpt Range: 1265-1356

nomero 2, twavugamo wendaaa; nko kujya mumihango kubakobwa, kugira amataye, uruhu runyerera

Title: Gs Munyiginya girls.docx

Doc Creator: tcuhawenimana11

Doc Date: 4/13/2023

Codes Applied: Physiological changes occuring during puberty-Girls

Linked Memos: 0

Excerpt Creator: tcuhawenimana11

Excerpt Created On: 4/28/2023

Excerpt Range: 1406-1461

nomero 02, araseka kwaguka nyine nko kukibuno namatako.

Title: Gs Munyiginya girls.docx

Doc Creator: tcuhawenimana11

Doc Date: 4/13/2023

Codes Applied: Physiological changes occuring during puberty-Girls

Linked Memos: 0

Excerpt Creator: tcuhawenimana11

Excerpt Created On: 4/28/2023

Excerpt Range: 1656-1722

nyine ijwi ry’umukobwa riba rinyerera wumva nyine yuko rivuga neza

Title: Gs Munyiginya girls.docx

Doc Creator: tcuhawenimana11

Doc Date: 4/13/2023

Codes Applied: Physiological changes occuring during puberty-Boys

Linked Memos: 0

Excerpt Creator: tcuhawenimana11

Excerpt Created On: 4/28/2023

Excerpt Range: 1765-1859

yego, ariko umuhungu we aba avuga mbaze nyine byumvikana rikomeye ko ageze mugihe cy’ubugabo.

Title: Gs Munyiginya girls.docx

Doc Creator: tcuhawenimana11

Doc Date: 4/13/2023

Codes Applied: Physiological changes occuring during puberty-Boys

Linked Memos: 0

Excerpt Creator: tcuhawenimana11

Excerpt Created On: 4/28/2023

Excerpt Range: 2032-2230

murakoze, nimero yange ni zero kane (04), imyaka ni 18, niga mumwaka w’amashuri yisumbuye niga muwa gatanu (5); impinduka numva ziba kubahungu bageze mugihe cyubugimbi, agira igihe cyo kwiroteraho,

Title: Gs Munyiginya girls.docx

Doc Creator: tcuhawenimana11

Doc Date: 4/13/2023

Codes Applied: Psychological changes occuring during puberty-Boys

Linked Memos: 0

Excerpt Creator: tcuhawenimana11

Excerpt Created On: 4/28/2023

Excerpt Range: 2230-2372

akagera muri cyagihe yumva yakora imibonano mpuzabitsina, akumva amaze kuba umugabo hahandi usanga nawe yumva yategeka. Nuko mbyumva murakoze.

Title: Gs Munyiginya girls.docx

Doc Creator: tcuhawenimana11

Doc Date: 4/13/2023

Codes Applied: Physiological changes occuring during puberty-Girls

Linked Memos: 0

Excerpt Creator: tcuhawenimana11

Excerpt Created On: 4/28/2023

Excerpt Range: 2445-2541

umukobwa nawe ugeze mubwangavu, uuh mugenzi wange wange yigeze kubivugaho, hari ukubona imihango

Title: Gs Munyiginya girls.docx

Doc Creator: tcuhawenimana11

Doc Date: 4/13/2023

Codes Applied: Pshcological changes occuring during puberty-Girls

Linked Memos: 0

Excerpt Creator: tcuhawenimana11

Excerpt Created On: 4/28/2023

Excerpt Range: 2543-2679

nyuma yo kubona iyo mihango mumubiri we harahinduka nawe akumva yakora imibinano mpuzabitsina, akumva yifuje wawundi badahuje igitsina.

Title: Gs Munyiginya girls.docx

Doc Creator: tcuhawenimana11

Doc Date: 4/13/2023

Codes Applied: Physiological changes occuring during puberty-Girls

Linked Memos: 0

Excerpt Creator: tcuhawenimana11

Excerpt Created On: 4/28/2023

Excerpt Range: 2850-3081

murakoze, nange nishimiye kuba ndi murubu bushakashatsi, nimero nicumi(10), imyaka nicumi n’irindwi(17), umwaka wamashuri numwaka wa 5 wamashuri yisumbuye, impinduka ziba kubakobwa zimwe bari bazivuze, ariko bari bibagiwe namabere.

Title: Gs Munyiginya girls.docx

Doc Creator: tcuhawenimana11

Doc Date: 4/13/2023

Codes Applied: Physiological changes occuring during puberty-Girls

Linked Memos: 0

Excerpt Creator: tcuhawenimana11

Excerpt Created On: 4/28/2023

Excerpt Range: 3133-3262

amabere nyine kubakobwa iyo bageze mugihe cyubwangavu, amabere arakura, akarenga uko yarameze wenda uko yarasanzwe ameze murakoze

Title: Gs Munyiginya girls.docx

Doc Creator: tcuhawenimana11

Doc Date: 4/13/2023

Codes Applied: Pshcological changes occuring during puberty-Girls

Linked Memos: 0

Excerpt Creator: tcuhawenimana11

Excerpt Created On: 4/28/2023

Excerpt Range: 3264-3393

iyo bageze mugihe cyubwagamvu baribona bagatangira bagahindura igendo bamwe baranirata bakanasuzugura igero zose na babyeyi babo.

Title: Gs Munyiginya girls.docx

Doc Creator: tcuhawenimana11

Doc Date: 4/13/2023

Codes Applied: Physiological changes occuring during puberty-Girls

Linked Memos: 0

Excerpt Creator: tcuhawenimana11

Excerpt Created On: 4/28/2023

Excerpt Range: 3394-3463

Ibiheri ibyo bakunda kwita ibishishi cg iyo augasanga agahu kanyereye

Title: Gs Munyiginya girls.docx

Doc Creator: tcuhawenimana11

Doc Date: 4/13/2023

Codes Applied: How girls experience changes occuring to them during puberty

Linked Memos: 0

Excerpt Creator: tcuhawenimana11

Excerpt Created On: 4/28/2023

Excerpt Range: 3549-3831

Ngewe nd’umukobwa bwa mbere mera amabere numvaga fite isoni nkajya gerageza kugenda mbihishahisha nambara umupira nkagenda mpese umugongo cg nkambara umupira ariko baraganirije babwira ko nyine ari bintu bibaho gerageza kubyakira numva yuko nyine nakuze mbasha kubyakira ndabyakira.

Title: Gs Munyiginya girls.docx

Doc Creator: tcuhawenimana11

Doc Date: 4/13/2023

Codes Applied: How girls experience changes occuring to them during puberty

Linked Memos: 0

Excerpt Creator: tcuhawenimana11

Excerpt Created On: 4/28/2023

Excerpt Range: 3845-4099

nomero ya 1 : hari izindi mpinduka zibz ku bakobwa nkajye bwa mbere nagiye mumihango ngira ubwoba cyane ndetse rimwe kumva ko ari bintu bitunguranye kandi ukumva umfite ubwoba.negera umubyeyi nyine arabisobanurira kandi abwira ko bibaho ku bakobwa bose.

Title: Gs Munyiginya girls.docx

Doc Creator: tcuhawenimana11

Doc Date: 4/13/2023

Codes Applied: How girls experience changes occuring to them during puberty

Linked Memos: 0

Excerpt Creator: tcuhawenimana11

Excerpt Created On: 4/28/2023

Excerpt Range: 4114-4732

nomero 07, mu mwaka nigamo ni senior 5 muri MEG mfite imyaka 17, rero nange bwambere mbijyamo jya mumihago ntabwo nyine nigeze mbibwira murugo nyine narfitei ubwoba ndetse ni isoni mvuga ngo ma mere azajya agenda mvuga ko umwana yarakuze jya kumutipeti nduturajye ndabimubwira konayigiyemo ampa cortex ntanubwo narinzi kucyambara nyine yabinyigishije uko ngomba ku najya nyine naragenda kigatakara nabigiyemo nkicuro nke shanu murugo batarabimenya. Ma mere yaje kubimbaza niba narajya mumihango mubwira ko yayigiyemo Ariko nyine Araganiriza nyine abwira iyo umuntu uyigiyemo ukuntu yitwara ari mumihango Nibyo ngibyo

Title: Gs Munyiginya girls.docx

Doc Creator: tcuhawenimana11

Doc Date: 4/13/2023

Codes Applied: Information that girls need

Linked Memos: 0

Excerpt Creator: tcuhawenimana11

Excerpt Created On: 4/28/2023

Excerpt Range: 4984-5016

ni 08, bakeneye nabo kuganirizwa

Title: Gs Munyiginya girls.docx

Doc Creator: tcuhawenimana11

Doc Date: 4/13/2023

Codes Applied: Information that girls need

Linked Memos: 0

Excerpt Creator: tcuhawenimana11

Excerpt Created On: 4/28/2023

Excerpt Range: 5160-5591

murakoze nimero ni icumi ; amakuru nyine umuntu akeneye abenshi muri twebwe hari abantu benshi bakiri muri RIB, hhhhh. Nyine amakaru dukeneye dukeneye amakuru ajyanye ni kubuzima bw’imyororokere bakatwigisha kubijyanye n’inda zitateganyijwe nyine ndakeka twese, hari utaragiye mumihango ra ? ndakeka twese twarayigiyemo bakatwigisha kubijyanye n’ubuzima bw’imyororokere hanze aha hari ibishuko byinshi tukamenya uburyo twakwirinda.

Title: Gs Munyiginya girls.docx

Doc Creator: tcuhawenimana11

Doc Date: 4/13/2023

Codes Applied: Information that boys need

Linked Memos: 0

Excerpt Creator: tcuhawenimana11

Excerpt Created On: 4/28/2023

Excerpt Range: 6051-6176

numero i 08, imyaka ni 18, nigwa mumwaka w’amashuri wa 3. Numva abahungu nabo ibintu baba bakeneye baba bakeneye kuganirizwa.

Title: Gs Munyiginya girls.docx

Doc Creator: tcuhawenimana11

Doc Date: 4/13/2023

Codes Applied: Types of information provided to boys and girls during puberty

Linked Memos: 0

Excerpt Creator: tcuhawenimana11

Excerpt Created On: 4/28/2023

Excerpt Range: 6327-6826

INOMERO ni 04. Aho tugenda aho tuba abanyeshuri tubana cyangwa se n’ababyeyi bacu batubwira ko umukobwa iyo yageze mubwangavu aba yamaze gukura nyine. Hanyuma rero ubwo aba agomba kwirinda kuba yakundana n’umuhungu kuko ngo niho zanda zitateganijwe zituruka. Ubwo rero bakatubwira ngo mujyw murindira biba biriho kuko ni ikindi cy’iciro uba wagezemo bakatubwira ngo nukwihangana mugakundana wamaze kuba bakuru kuko iyo mukundanye ukiri muto ibishuko nabyo biba byinshi nabwo biturutse kuri zangimbi.

Title: Gs Munyiginya girls.docx

Doc Creator: tcuhawenimana11

Doc Date: 4/13/2023

Codes Applied: Types of information provided to boys and girls during puberty

Linked Memos: 0

Excerpt Creator: tcuhawenimana11

Excerpt Created On: 4/28/2023

Excerpt Range: 6897-7361

Murakoze. Numero ni 05 niga mumwaka wa 2 w’amashuri y’isumbuye. Nyine andi makuru numvise bambwiye nuko nyine umuntu ugeze mugihecy’ubwangavu n’ubugimbi, bamwira ko umuntu nyine watangiye kujya mumihango yazanye amabere nyine byabimenyetso byose bavuze aba yumva yakuze ariko nyine aba atarageza igihe cyo gukora imibonano mpuzabitsina, nyine bamwira ko umuntu uba uri munsi y’imyaka 20 aba atemerewe gukora imibonano mpuzabitsina kandi aba bakeneye inama nyinshi.

Title: Gs Munyiginya girls.docx

Doc Creator: tcuhawenimana11

Doc Date: 4/13/2023

Codes Applied: Information that girls need

Linked Memos: 0

Excerpt Creator: tcuhawenimana11

Excerpt Created On: 4/28/2023

Excerpt Range: 7405-7592

murakoze numero ni 01 imyaka ni 13. Umwaka w’amashuri ndi muwa 1. Ibindi bitu dukenera nyine ni inama umukobwa ari mumihango nyine umuntu aba akeneye isuku ihagije mumyanya ndangagitsina.

Title: Gs Munyiginya girls.docx

Doc Creator: tcuhawenimana11

Doc Date: 4/13/2023

Codes Applied: Sources of information received during puberty

Linked Memos: 0

Excerpt Creator: tcuhawenimana11

Excerpt Created On: 4/28/2023

Excerpt Range: 7822-8005

murakoze ni nimero 7. Imyaka ni 17, umwaka w’amashuri ni muwa 2. Amakuru ahantu tuyakura akenshi ni kunshuti, kubabyeyi, batubwira uburyo twakwirinda bakatubwira n’uburyo twakwitwara.

Title: Gs Munyiginya girls.docx

Doc Creator: tcuhawenimana11

Doc Date: 4/13/2023

Codes Applied: Sources of information received during puberty

Linked Memos: 0

Excerpt Creator: tcuhawenimana11

Excerpt Created On: 4/28/2023

Excerpt Range: 8062-8213

nyine yaramaze kubivyga numero ni 10 amakuru tuyakura kubabyeyi bacu, kubavandimwe n’inshuti no kukiginderabuzima. Tujyayo kukigonderabuzima kuyashaka.

Title: Gs Munyiginya girls.docx

Doc Creator: tcuhawenimana11

Doc Date: 4/13/2023

Codes Applied: Obstacles and risks experienced by boys during the sexual and reproductive health changes occuring during puberty and adolescence

Linked Memos: 0

Excerpt Creator: tcuhawenimana11

Excerpt Created On: 4/28/2023

Excerpt Range: 8364-8675

murakoze. Harigihe abana iyo bageze mugihe cy ‘ubwangavu n’ubugimbi bareka ishuri bakajya guhiga amafaranga, hanyuma abana b’abakobwa bakararikira nyine imyenda igezweho, iwabo ntanubushobozi bafite bwo kuyimugurira akagenda agahura nuwo muhungu akayimugurira akamutera n’inda nyine iyo ni ingorane ahura nayo.

Title: Gs Munyiginya girls.docx

Doc Creator: tcuhawenimana11

Doc Date: 4/13/2023

Codes Applied: Obstacles and risks experienced by girls during the sexual and reproductive health changes occuring during puberty and adolescence

Linked Memos: 0

Excerpt Creator: tcuhawenimana11

Excerpt Created On: 4/28/2023

Excerpt Range: 8790-8946

numero yanjye ni 09 ingorane abageze mubwagaviu cg ubugimbi bagira iyo umukobwa bamuteye inda akiri umwana arwara indwara mumyanya ndangagitsina na fisitile

Title: Gs Munyiginya girls.docx

Doc Creator: tcuhawenimana11

Doc Date: 4/13/2023

Codes Applied: Obstacles and risks experienced by boys during the sexual and reproductive health changes occuring during puberty and adolescence Obstacles and risks experienced by girls during the sexual and reproductive health changes occuring during puberty and adolescence

Linked Memos: 0

Excerpt Creator: tcuhawenimana11

Excerpt Created On: 4/28/2023

Excerpt Range: 8998-9432

murakoze. Nimero yanjye ni 6. Ingaruka abantu bageze mubwangavu, urumva nyine iyo amazee kugera mubwangavu aba yumva na mere we amurenzeho ati mere afite amabere nanjye ndayafite, cyangwa na viye iyo umutipe ageze mubugimbi akareba viye ati se nanjye natera inda noneho yahura n’abandi bajyama wenda bafata kubiyobyabwenge akajyana nabo kuba muri geto ugasanga nyine abayeho nabi cg nibyo biyobyabwenge ugasanga bimwiciye ejo hazaza.

Title: Gs Munyiginya girls.docx

Doc Creator: tcuhawenimana11

Doc Date: 4/13/2023

Codes Applied: Obstacles and risks experienced by girls during the sexual and reproductive health changes occuring during puberty and adolescence

Linked Memos: 0

Excerpt Creator: tcuhawenimana11

Excerpt Created On: 4/28/2023

Excerpt Range: 9483-9706

yego barahari cyane, abakobwa bananiye iwabo barabinywa. Rero umukobwa iyo yumv yamaze kurenga kuri mere we ajya kwirebera abatipe kuko nawe aba yumva yabyara umwana ubwo ugasanga abyaye imburagihe kariyeri ye irangiritse.

Title: Gs Munyiginya girls.docx

Doc Creator: tcuhawenimana11

Doc Date: 4/13/2023

Codes Applied: Obstacles and risks experienced by girls during the sexual and reproductive health changes occuring during puberty and adolescence

Linked Memos: 0

Excerpt Creator: tcuhawenimana11

Excerpt Created On: 4/28/2023

Excerpt Range: 9820-10060

MURAKOZE ! izindi ngorane, ubundi iyo wageze mugihe cy’ubwangavu, usanga hari abantu benshi bagufitiye irari ugasanga bagufashe kungufu baguteye inda ugasanga bakwangirije intego niba warufite intego ntuyigezeho ugahangayika ukigunga nyine.

Title: Gs Munyiginya girls.docx

Doc Creator: tcuhawenimana11

Doc Date: 4/13/2023

Codes Applied: Obstacles and risks experienced by girls during the sexual and reproductive health changes occuring during puberty and adolescence

Linked Memos: 0

Excerpt Creator: tcuhawenimana11

Excerpt Created On: 4/28/2023

Excerpt Range: 10089-10334

murakoze !nomero yanjye ni gatatu hari abangavu bahura n’ingorane, barangiza bakananiranwa n’iwabo bakaba indaya bakava iwabo bakajya kunywa ibiyobyabwenge barangiza bakabatera amada bagata abana babo ugasanga ni bamwe baba baragiye barangisha.

Title: Gs Munyiginya girls.docx

Doc Creator: tcuhawenimana11

Doc Date: 4/13/2023

Codes Applied: Obstacles and risks experienced by boys during the sexual and reproductive health changes occuring during puberty and adolescence

Linked Memos: 0

Excerpt Creator: tcuhawenimana11

Excerpt Created On: 4/28/2023

Excerpt Range: 10377-10826

Murakoze ! nanjye icyo nakongeraho, kubahungu bageze mugihe cy’ubugimbi bashobora guhura nababamama bitwa ba shuga mami. Urugero niba mwumva urunana hari uriya witwa cedrick oya Jacky ingaruka mbi yagize nuko yararikiye ibintu atabasha kubona ubwo rero ahura na Jacky aramushuka aviramo kuva mu ishuri nubwo jacky bamufunze ariko cedrick aracyatsimbaraye kuri jacky, ubwo rero ba shuga dadi kubahungu naba shuga mami kubakobwa bajya bateza ingorane.

Title: Gs Munyiginya girls.docx

Doc Creator: tcuhawenimana11

Doc Date: 4/13/2023

Codes Applied: Obstacles and risks experienced by girls during the sexual and reproductive health changes occuring during puberty and adolescence

Linked Memos: 0

Excerpt Creator: tcuhawenimana11

Excerpt Created On: 4/28/2023

Excerpt Range: 11297-11662

murakoze nomero ni 8, imyaka ni 17, mumwaka wa 3. Rero ndatekereza inzitizi bashobora guura nazo nkiyo bataye ishuri, ku ishuri tuhamenyera byinshi kiyo tugize amahirwe bakadusura bakatuganiriza, kenshi nkiyo abaganga badusuye baratuganiriza, bakatuganiriza kubuzima bw’imyororokere, ubwo rero uwonguwo wataye ishuri ntabyo ashobora kumenya kuko ntaho ahurira nabyo

Title: Gs Munyiginya girls.docx

Doc Creator: tcuhawenimana11

Doc Date: 4/13/2023

Codes Applied: Obstacles and risks experienced by girls during the sexual and reproductive health changes occuring during puberty and adolescence

Linked Memos: 0

Excerpt Creator: tcuhawenimana11

Excerpt Created On: 4/28/2023

Excerpt Range: 11708-11778

Nimero yanjye ni 09, inzitizi nyine bahura nazo ni ukudahabwa amakuru.

Title: Gs Munyiginya girls.docx

Doc Creator: tcuhawenimana11

Doc Date: 4/13/2023

Codes Applied: Barriers leading to the risks and obstacles girls and boys experience during puberty and adolescence

Linked Memos: 0

Excerpt Creator: tcuhawenimana11

Excerpt Created On: 4/28/2023

Excerpt Range: 11908-12561

muraoze. Inomero ni 06. Inzitizi bahura nazo hari igihe uba ufite umumere usepera cyane ugasanga ntanubwo akuganiriza, ubwo nyine iyo atakuganiriza nyine nawe ukora ibyo wumva birimo kukuzamo. Hari n’ababyeyi nyine batajya bita kubana babo ngo babaze nyine uko ubuzima bwabo bumeze cg ntabone n’amakuru ajyanye n’ubuzima akumva ko niyo yajya mumihango ari ibintu biraho niyo yakora imibonano mpuzabitsina asambanye ntabwo yatwara unda, ubwo akishoramo gutyo nabo babyeyi babo baba batabareba nyine. Cyangwa hari igihe umumere aba afite akazi kenshi aban bitabwaho n’umukozi ugasanga umukaozi aravuga ati ni akazi abo nanjye naje gushaka moni ntabidanje.

Title: Gs Munyiginya girls.docx

Doc Creator: tcuhawenimana11

Doc Date: 4/13/2023

Codes Applied: Barriers leading to the risks and obstacles girls and boys experience during puberty and adolescence

Linked Memos: 0

Excerpt Creator: tcuhawenimana11

Excerpt Created On: 4/28/2023

Excerpt Range: 12687-12796

ugasanga nyine umukozi ntabona niyo time yo kuganiriza umwana ugasanga nyine ajya murizo ngeso mbi. Murakoze.

Title: Gs Munyiginya girls.docx

Doc Creator: tcuhawenimana11

Doc Date: 4/13/2023

Codes Applied: Barriers leading to the risks and obstacles girls and boys experience during puberty and adolescence

Linked Memos: 0

Excerpt Creator: tcuhawenimana11

Excerpt Created On: 4/28/2023

Excerpt Range: 12828-13024

murakoze, nimero ni 5. Nk’abana b’abakobwa inzitizi dukunda guhura nazo, harubwo ushobora kwitinya bitewe nabo babyeyo wenda mutemeranyaho cyangwa ubazanyeho ibyo bintu by’ubuzima bw’imyororokere.

Title: Gs Munyiginya girls.docx

Doc Creator: tcuhawenimana11

Doc Date: 4/13/2023

Codes Applied: Barriers leading to the risks and obstacles girls and boys experience during puberty and adolescence

Linked Memos: 0

Excerpt Creator: tcuhawenimana11

Excerpt Created On: 4/28/2023

Excerpt Range: 13074-13122

wenda ufite ipfunwe ryo kuba wabivuga. Murakoze.

Title: Gs Munyiginya girls.docx

Doc Creator: tcuhawenimana11

Doc Date: 4/13/2023

Codes Applied: Barriers leading to the risks and obstacles girls and boys experience during puberty and adolescence

Linked Memos: 0

Excerpt Creator: tcuhawenimana11

Excerpt Created On: 4/28/2023

Excerpt Range: 13198-13247

Wenda bitewe nuko wiyumva muri wowe ufite ubwoba.

Title: Gs Munyiginya girls.docx

Doc Creator: tcuhawenimana11

Doc Date: 4/13/2023

Codes Applied: Barriers leading to the risks and obstacles girls and boys experience during puberty and adolescence

Linked Memos: 0

Excerpt Creator: tcuhawenimana11

Excerpt Created On: 4/28/2023

Excerpt Range: 13296-13777

IKINDI. Hari ukuntu umwana aba afite ababyeyi batagira icyo bitaho. Bakaba babana mubuzima bwa burimunsi ariko batamwitayeho.ugasanga umwana w’umukobwa akundanye n’umuungu, atahanye ikintu ejo atahanye ikindi, umubyeyi arabibona kandi ntacyo yamuguriye ntakurikirane ngo amubaze aho yabikuye, akabona afite amafaranga kandi ntakazi afite ntakurirane ngo amubaze aho yayakuye akamureka. Ugasanga rero nabo baba munzitizi kuko ugasanga barabareka bakirera batyo. Nuko ndimo kubyumva.

Title: Gs Munyiginya girls.docx

Doc Creator: tcuhawenimana11

Doc Date: 4/13/2023

Codes Applied: Suggestions to improve SRH during puberty and adolescence by girls

Linked Memos: 0

Excerpt Creator: tcuhawenimana11

Excerpt Created On: 4/28/2023

Excerpt Range: 14031-14338

ikindi kintu wenda tutavuzeho, nkababyeyi wenda twabatuma mukatubwirira ababyeyi bakajya bafata umwanya bakaganiriza abana kubuzima bw’imyororokere kuko iyo batabaganirije ni hamwe usanga bicuza ndetse ugasanga aragaya ababyeyi be mugihe ingaruka zo kuba bataraganirijwe n’ababyeyi be zatangiye kubageraho.

Title: Gs Munyiginya girls.docx

Doc Creator: tcuhawenimana11

Doc Date: 4/13/2023

Codes Applied: Obstacles and risks experienced by girls during the sexual and reproductive health changes occuring during puberty and adolescence

Linked Memos: 0

Excerpt Creator: tcuhawenimana11

Excerpt Created On: 4/28/2023

Excerpt Range: 14600-15158

murkoze. Nimero ni 10. Uko mbibona nyine aakonbwa babyariye iwabo bagenda bahura n’inzitizi nyinshi nkuko nyine mugenzi wanjye yigeze kuvuga kurunana nka mutesi wo murunana aba arwumva. Mutesi wo murunana nyine ntababyeyi yagirag barapfuye abana na musaza we hanyuma akajya ashaka uko atunga abavandimwe be, ariko mutesi we ntanyurwe agashaka kwisiga ngo ase neza ajyane n’abahungu bamubone. Bakamugurir mukorogo akajya yisiga biza kuvamo ko bamutera indi ubundi yajyaga akoresha agakingirizo. Bamutera inda none ubu yarwaye fisitire kubera kubyara ari muto.

Title: Gs Munyiginya girls.docx

Doc Creator: tcuhawenimana11

Doc Date: 4/13/2023

Codes Applied: Obstacles and risks experienced by girls during the sexual and reproductive health changes occuring during puberty and adolescence

Linked Memos: 0

Excerpt Creator: tcuhawenimana11

Excerpt Created On: 4/29/2023

Excerpt Range: 15243-15938

Abo duturanye hari uwabyaye, iwabo ntabwo bari bishoboye barakodeshaga. Yigaga muwa 3 bamuter inda byaje no kumuviramo ko aza muri Ukubera ko kubera n’inda atabonaga uko yiga nyine. Icyo gihe yaratwite kubera n’iwabo ntamafaranga abantu bajya gutera ikiraka bakajya guhingira igihumbi kugirango baze nuwo mwana babone uko bamutunga ndetse bikamugora rimwe na rimwe bakicwa n’inzara ntibabone ibyo kurya mbese ntibarye burimunsi uko babishatse kandi umuntu ufite umwana uko aba angana kose aba akeneye kurya. Uhuriramo n’imbogamizi nyinshi nyine shobora no gupfa yishwe niyo nzara, akabura imyenda yo kwambara ikamusaziraho akabur isabune yo kumesera umwana yewe akabura n’imyenda yo kumwambika.

Title: Gs Munyiginya girls.docx

Doc Creator: tcuhawenimana11

Doc Date: 4/13/2023

Codes Applied: Precautions to take during puberty for girls

Linked Memos: 0

Excerpt Creator: tcuhawenimana11

Excerpt Created On: 4/29/2023

Excerpt Range: 16626-16811

murakoze. Igihe nyine. Icyambere ubundi ni ukwifata, mugihe nyine kwifata byanze i gombwa ngo ukoreshe ako gakingirizo kugira ngo wirinde izo nda ndetse n’indwara zandurira mumibonano

Title: GS kiziguro Girls transc.docx

Doc Creator: tcuhawenimana11

Doc Date: 4/13/2023

Codes Applied: Physiological changes occuring during puberty-Boys

Linked Memos: 0

Excerpt Creator: tcuhawenimana11

Excerpt Created On: 4/29/2023

Excerpt Range: 355-559

Murakoze!!!impinduka ziba kumuhungu (ubaza: WAGERAGEZA UKAZAMURA IJWI), impinduka zibaho kubahungu ninko kuba bageze mugihe cyo kuniga ijwi barangiza bakarwara ibiheri, bakiroteraho nibyongibyo naba numva

Title: GS kiziguro Girls transc.docx

Doc Creator: tcuhawenimana11

Doc Date: 4/13/2023

Codes Applied: Boys and girls know about the causes leading to the physiological changes during puberty

Linked Memos: 0

Excerpt Creator: tcuhawenimana11

Excerpt Created On: 4/29/2023

Excerpt Range: 633-699

wenda kwiroteraho biterwa nimihindagurikire y’imyaka baba bagezemo

Title: GS kiziguro Girls transc.docx

Doc Creator: tcuhawenimana11

Doc Date: 4/13/2023

Codes Applied: Physiological changes occuring during puberty-Boys

Linked Memos: 0

Excerpt Creator: tcuhawenimana11

Excerpt Created On: 4/29/2023

Excerpt Range: 922-1217

Murakoze !!! code yanjye ni , ziro ziro three ,Imyaka ni Eighteen years twenty twenty two, I study this seniors six .impinduka nyine zigaragara kubahungu bamwe nabamwe tujya tubona nko kuba bamera incakwaha, kuba bazana ubwanwa , kuba wabonaga umuhungu nyine uko yavugaga ugasanga birahindutse,

Title: GS kiziguro Girls transc.docx

Doc Creator: tcuhawenimana11

Doc Date: 4/13/2023

Codes Applied: Psychological changes occuring during puberty-Boys

Linked Memos: 0

Excerpt Creator: tcuhawenimana11

Excerpt Created On: 4/29/2023

Excerpt Range: 1217-1471

ugasanga n’imyitwarire yarafite muri bagenzibe ugasanga nyine atangiye kugenda agira ibintu byamasoni, ugasanga niba yagiraga akavuyo mubandi ugasanga nyine atangiye kugenda acisha macye bitewe nimyaka agezemo , rimwe narimwe nyine kubahungu bajya bagira

Title: GS kiziguro Girls transc.docx

Doc Creator: tcuhawenimana11

Doc Date: 4/13/2023

Codes Applied: Physiological changes occuring during puberty-Boys

Linked Memos: 0

Excerpt Creator: tcuhawenimana11

Excerpt Created On: 4/29/2023

Excerpt Range: 1473-1542

nyine nigihe cyo bageramo bakamera n’ubwanwa, murakoze nibyo numvaga.

Title: GS kiziguro Girls transc.docx

Doc Creator: tcuhawenimana11

Doc Date: 4/13/2023

Codes Applied: Boys and girls know about the causes leading to the physiological changes during puberty

Linked Memos: 0

Excerpt Creator: tcuhawenimana11

Excerpt Created On: 4/29/2023

Excerpt Range: 1593-1670

biterwa nyine nimihindagurikire yubuzima bwabo yigihe baba bagezemo n’imyaka.

Title: GS kiziguro Girls transc.docx

Doc Creator: tcuhawenimana11

Doc Date: 4/13/2023

Codes Applied: Boys and girls know about the causes leading to the physiological changes during puberty

Linked Memos: 0

Excerpt Creator: tcuhawenimana11

Excerpt Created On: 4/29/2023

Excerpt Range: 1713-2145

murakoze. nimero ni zeru zeru rimwe, imyaka ni cumi numunani, niga senior six.

Mwunganiye ahongaho ikintu ikintu nzi cyaba gitera imihindagurikire, ubundi iyo umuhungu ageze mubugimbi hari imisemburo itangira kuvuburwa itajyaga ivuburwa igihe yarari yarari mumyaka mitoya nyine, iyo misemburo rero iyo itangiye kwivubura niho haba iyo mihindagurikire yo kumera incakwaha, guhindura ijwi niyo myitwarire yose yagiye avuga, murakoze.

Title: GS kiziguro Girls transc.docx

Doc Creator: tcuhawenimana11

Doc Date: 4/13/2023

Codes Applied: Physiological changes occuring during puberty-Boys

Linked Memos: 0

Excerpt Creator: tcuhawenimana11

Excerpt Created On: 4/29/2023

Excerpt Range: 2200-2411

code yanjye ni zeru zeru rimwe, imyaka ni cumi nirindwi niga muwagatanu segonderi

Njyewe icyo navuga kubahungu iyo bageze mugihe cyubugimbi, byinshi babivuze harimo uko kuba bazana incakwaha, kuba bakwiroteraho,

Title: GS kiziguro Girls transc.docx

Doc Creator: tcuhawenimana11

Doc Date: 4/13/2023

Codes Applied: Boys and girls know about the causes leading to the physiological changes during puberty

Linked Memos: 0

Excerpt Creator: tcuhawenimana11

Excerpt Created On: 4/29/2023

Excerpt Range: 2412-2502

icyo numva kibitera cyase ikibitera nigihe baba bagezemo bitewe nimyaka bagezemo murakoze.

Title: GS kiziguro Girls transc.docx

Doc Creator: tcuhawenimana11

Doc Date: 4/13/2023

Codes Applied: Boys and girls know about the causes leading to the physiological changes during puberty

Linked Memos: 0

Excerpt Creator: tcuhawenimana11

Excerpt Created On: 4/29/2023

Excerpt Range: 2645-2808

Ikintunumva kibitera, biterwa nuko umubiri wabo uba warahindaguritse kandi nimyaka baba bagezemo iba yeme ibemerera kugira iyo mihindagurikire y’umubiri, murakoze.

Title: GS kiziguro Girls transc.docx

Doc Creator: tcuhawenimana11

Doc Date: 4/13/2023

Codes Applied: Physiological changes occuring during puberty-Boys

Linked Memos: 0

Excerpt Creator: tcuhawenimana11

Excerpt Created On: 4/29/2023

Excerpt Range: 2899-2946

kwiroteraho, no kuniga ijwi no kumera ubwanwa.

Title: GS kiziguro Girls transc.docx

Doc Creator: tcuhawenimana11

Doc Date: 4/13/2023

Codes Applied: Physiological changes occuring during puberty-Boys

Linked Memos: 0

Excerpt Creator: tcuhawenimana11

Excerpt Created On: 4/29/2023

Excerpt Range: 3024-3161

Murakoze nimero yanjye ni cumi na rimwe

Jyewe mbona umubiri wabahungu imihindagurikire yabo arukumera incakwaha no kuzana ibiheri mumaso,

Title: GS kiziguro Girls transc.docx

Doc Creator: tcuhawenimana11

Doc Date: 4/13/2023

Codes Applied: Boys and girls know about the causes leading to the physiological changes during puberty

Linked Memos: 0

Excerpt Creator: tcuhawenimana11

Excerpt Created On: 4/29/2023

Excerpt Range: 3162-3233

bikaba biterwa nigihe imyaka yabo igezemo, bikaba biterwa numubiri wabo

Title: GS kiziguro Girls transc.docx

Doc Creator: tcuhawenimana11

Doc Date: 4/13/2023

Codes Applied: Physiological changes occuring during puberty-Boys

Linked Memos: 0

Excerpt Creator: tcuhawenimana11

Excerpt Created On: 4/29/2023

Excerpt Range: 3323-3467

murakoze nimero yanjye ni cumi na gatatu , njywe ibintu nyine numva batavuze ,ninko kuba bamera insya uuu ,nibyongibyo numva batigeza bavuze .

Title: GS kiziguro Girls transc.docx

Doc Creator: tcuhawenimana11

Doc Date: 4/13/2023

Codes Applied: Boys and girls know about the causes leading to the physiological changes during puberty

Linked Memos: 0

Excerpt Creator: tcuhawenimana11

Excerpt Created On: 4/29/2023

Excerpt Range: 3542-3575

no kuba bageze mumyaka yubugimbi.

Title: GS kiziguro Girls transc.docx

Doc Creator: tcuhawenimana11

Doc Date: 4/13/2023

Codes Applied: Physiological changes occuring during puberty-Boys

Linked Memos: 0

Excerpt Creator: tcuhawenimana11

Excerpt Created On: 4/29/2023

Excerpt Range: 3912-4078

nimero yanjye ni zeru cumin a kane niga mumwaka wa gatandatu wamashuri abanza

Nanjye ikintu mvuga kubahungu iyo bageze mugihe cyubugimbi batangira kugira ibituzabini.

Title: GS kiziguro Girls transc.docx

Doc Creator: tcuhawenimana11

Doc Date: 4/13/2023

Codes Applied: Boys and girls know about the causes leading to the physiological changes during puberty

Linked Memos: 0

Excerpt Creator: tcuhawenimana11

Excerpt Created On: 4/29/2023

Excerpt Range: 4214-4261

byaba byaba biterwa nigihe bagezemo cyubugimbi.

Title: GS kiziguro Girls transc.docx

Doc Creator: tcuhawenimana11

Doc Date: 4/13/2023

Codes Applied: Physiological changes occuring during puberty-Girls

Linked Memos: 0

Excerpt Creator: tcuhawenimana11

Excerpt Created On: 4/29/2023

Excerpt Range: 4466-4824

murakoze, code yanjye ni zeru zeru rimwe, imyaka ni cumi nirindwi, muwa niga muwa gatandatu segonderi.

Kuuu kubijyanye nimpinduka ziba kubakobwa mugihe cy’uwbwangavu navugamo nko kuba yamera amabere, kujya mu mihango, kuba nawe yamera izo ncakwaha no kuba ijwi rye we ntabwo ari niga ahubwo navugako riiibamo ritoya ukuntu nawe ijwirye rirahinduka yeah eeee.

Title: GS kiziguro Girls transc.docx

Doc Creator: tcuhawenimana11

Doc Date: 4/13/2023

Codes Applied: Boys and girls know about the causes leading to the physiological changes during puberty

Linked Memos: 0

Excerpt Creator: tcuhawenimana11

Excerpt Created On: 4/29/2023

Excerpt Range: 4825-4884

impamvu ibitera nawe nigihe aba agezemo imyaka aba agezemo.

Title: GS kiziguro Girls transc.docx

Doc Creator: tcuhawenimana11

Doc Date: 4/13/2023

Codes Applied: Physiological changes occuring during puberty-Girls

Linked Memos: 0

Excerpt Creator: tcuhawenimana11

Excerpt Created On: 4/29/2023

Excerpt Range: 4904-5145

murakoze, code yanjye ni zeru zeru gatandatu, mfite imyaka cumi nirindwi , niga muwakane segonderi.impinduka ziba kubakobwa mugihe cyubwangavu.

Nukujya mumihango ,kumera incakwaha, guhinduka ijwi ndetse bakiyitaho bishoboka bakigirira isuku

Title: GS kiziguro Girls transc.docx

Doc Creator: tcuhawenimana11

Doc Date: 4/13/2023

Codes Applied: Pshcological changes occuring during puberty-Girls

Linked Memos: 0

Excerpt Creator: tcuhawenimana11

Excerpt Created On: 4/29/2023

Excerpt Range: 5197-5362

eeeeee ukumvako utasohoka cyangwa ngo ube wajya kumuhanda utakarabye cyangwase utafuze nyine ibyobyose biterwa nigihe umuntu aba agezemo cy’imyaka yimihindagurikire.

Title: GS kiziguro Girls transc.docx

Doc Creator: tcuhawenimana11

Doc Date: 4/13/2023

Codes Applied: Physiological changes occuring during puberty-Girls

Linked Memos: 0

Excerpt Creator: tcuhawenimana11

Excerpt Created On: 4/29/2023

Excerpt Range: 5474-5634

murakoze nimero yanjye ni 008 nkaba mfite imyaka 16,

Nkaba niga mumwaka wa kane wamashuri yisumbuye.

Impinduka ziba kumukobwa nukumera amabere, akajya mumihango

Title: GS kiziguro Girls transc.docx

Doc Creator: tcuhawenimana11

Doc Date: 4/13/2023

Codes Applied: Pshcological changes occuring during puberty-Girls

Linked Memos: 0

Excerpt Creator: tcuhawenimana11

Excerpt Created On: 4/29/2023

Excerpt Range: 5635-5669

nyine akumva nyine arakuze muriwe.

Title: GS kiziguro Girls transc.docx

Doc Creator: tcuhawenimana11

Doc Date: 4/13/2023

Codes Applied: Pshcological changes occuring during puberty-Girls

Linked Memos: 0

Excerpt Creator: tcuhawenimana11

Excerpt Created On: 4/29/2023

Excerpt Range: 5819-5878

uuuuuuu aba yumva atajya ahantu adasaneza ,kwigirira isuku

Title: GS kiziguro Girls transc.docx

Doc Creator: tcuhawenimana11

Doc Date: 4/13/2023

Codes Applied: Boys and girls know about the causes leading to the physiological changes during puberty

Linked Memos: 0

Excerpt Creator: tcuhawenimana11

Excerpt Created On: 4/29/2023

Excerpt Range: 5879-5942

Nkumva arero ikibitera nimisemburo ibaaa iri mumubiriwe nyine.

Title: GS kiziguro Girls transc.docx

Doc Creator: tcuhawenimana11

Doc Date: 4/13/2023

Codes Applied: Physiological changes occuring during puberty-Girls

Linked Memos: 0

Excerpt Creator: tcuhawenimana11

Excerpt Created On: 4/29/2023

Excerpt Range: 6050-6316

murakoze, nimero yanjye ni 009 imyaka yanjye ni 17 ni 9 niga muwakane mumashuri yisumbuye .

Imihindagurikire igaragara kumwana wumukobwa harimo; kujya mumihango ,kumera amabere ,kumera incakwaha ,agira uruhu runyereye rutandukanye nurwo yarafite akiri umwana mutoya

Title: GS kiziguro Girls transc.docx

Doc Creator: tcuhawenimana11

Doc Date: 4/13/2023

Codes Applied: Pshcological changes occuring during puberty-Girls

Linked Memos: 0

Excerpt Creator: tcuhawenimana11

Excerpt Created On: 4/29/2023

Excerpt Range: 6317-6465

,hanyuma akiyitaho,akigirira isuku, akumva yahora asaneza igihe cyose ,akumva murugo iwabo yahora ahakora isuku ahantu hagahora hasukuye hanakropye.

Title: GS kiziguro Girls transc.docx

Doc Creator: tcuhawenimana11

Doc Date: 4/13/2023

Codes Applied: Boys and girls know about the causes leading to the physiological changes during puberty

Linked Memos: 0

Excerpt Creator: tcuhawenimana11

Excerpt Created On: 4/29/2023

Excerpt Range: 6466-6600

Hanyuma ikintu kibitera aba yumva akuze muriwe, nimisemburo igenda ivuburwa numubiriwe, akumva agomba kwiyitaho ahantu hose. Murakoze

Title: GS kiziguro Girls transc.docx

Doc Creator: tcuhawenimana11

Doc Date: 4/13/2023

Codes Applied: Physiological changes occuring during puberty-Girls

Linked Memos: 0

Excerpt Creator: tcuhawenimana11

Excerpt Created On: 4/29/2023

Excerpt Range: 6659-6879

murakoze ,nimero yanjye ni 010,imyaka ni 17 ,niga mumwaka wa 4. Njyewe ikintu navuga kumpinduka ziba kumwana w’umukobwa, umwana wumukobwa iyo akuze ; aratangira akamera amabere, akajya mumihango akazana nikibuno kinini .

Title: GS kiziguro Girls transc.docx

Doc Creator: tcuhawenimana11

Doc Date: 4/13/2023

Codes Applied: Pshcological changes occuring during puberty-Girls

Linked Memos: 0

Excerpt Creator: tcuhawenimana11

Excerpt Created On: 4/29/2023

Excerpt Range: 6879-6955

urebye kikimu abahindutse muriwe nyine, mumubiriwe abayumva ahindutse cyane.

Title: GS kiziguro Girls transc.docx

Doc Creator: tcuhawenimana11

Doc Date: 4/13/2023

Codes Applied: Physiological changes occuring during puberty-Girls

Linked Memos: 0

Excerpt Creator: tcuhawenimana11

Excerpt Created On: 4/29/2023

Excerpt Range: 7029-7210

murakoze nimero yanjye ni 005, mfite imyaka 16, niga muri s3.

Mumpinduka ziba kumukobwa agira amataye, ijwirye riba riseseka, yiyitaho cyane akigirira isuku, nibyo narinzi murakoze.

Title: GS kiziguro Girls transc.docx

Doc Creator: tcuhawenimana11

Doc Date: 4/13/2023

Codes Applied: Pshcological changes occuring during puberty-Girls

Linked Memos: 0

Excerpt Creator: tcuhawenimana11

Excerpt Created On: 4/29/2023

Excerpt Range: 7254-7550

ibindi basize batatubwiye nyine umukobwa ageze mugihe cy’ubwangavu, atangira kugira udusoni yaca kubahungu akabona birimo kumureba kandi ntanumwitayeho, akumva atangiye gukunda abahungu cyane nawe nyine nibintu bimuzamo, ehe ikindi nyine kugitsina amaera ibyo bita isya, nibyo batavuze murakoze.

Title: GS kiziguro Girls transc.docx

Doc Creator: tcuhawenimana11

Doc Date: 4/13/2023

Codes Applied: Physiological changes occuring during puberty-Girls

Linked Memos: 0

Excerpt Creator: tcuhawenimana11

Excerpt Created On: 4/29/2023

Excerpt Range: 7635-7949

murakoze, nimero yanjye ni 012, nkaba niga mumwaka waamashuri yisumbuye wa 3 nkaba niga muri GS kiziguro, nkaba numva izindi mpinduka abatavuze ziboneka kumwana wumukobwa w’umwangavu arukuzana ibiheri cyangwa ibyo bita ibishishi rimwe narimwe kubakobwa bagiye guta “azana ibiheri cyngwa ibishishi” eeeeeee murakoze

Title: GS kiziguro Girls transc.docx

Doc Creator: tcuhawenimana11

Doc Date: 4/13/2023

Codes Applied: Pshcological changes occuring during puberty-Girls

Linked Memos: 0

Excerpt Creator: tcuhawenimana11

Excerpt Created On: 4/29/2023

Excerpt Range: 8128-8295

murakoze, kuruhare rw’umukobwa, harigihe iyo ageze muri icyogihe cyo kujya mumihango aribwo ubushutibwe bwiyongera kumubyeyi w’umumama, cyaneko aba ariwe yisanzuyeho

Title: GS kiziguro Girls transc.docx

Doc Creator: tcuhawenimana11

Doc Date: 4/13/2023

Codes Applied: Pshcological changes occuring during puberty-Girls

Linked Memos: 0

Excerpt Creator: tcuhawenimana11

Excerpt Created On: 4/29/2023

Excerpt Range: 8342-8572

umubyeyi w’umumama kumukobwa, ugasanga nyine niwe yisanzuyeho aba amwaka izo padi, aba amubwirako igihe cye cyo kujya mumihango cyageze, ugasanga nyine rimwe narimwe aritinya kuba yagira icyo yabwira papa we kubijya nye nimihango.

Title: GS kiziguro Girls transc.docx

Doc Creator: tcuhawenimana11

Doc Date: 4/13/2023

Codes Applied: How girls experience changes occuring to them during puberty

Linked Memos: 0

Excerpt Creator: tcuhawenimana11

Excerpt Created On: 4/29/2023

Excerpt Range: 8982-9480

ok kuberako ubundi muri kino gihe abanyeshuri uba umwana washyitse muri p5&p6 abayatangiye kubyigaho ariko nyine mubyukuri umuntu ntabwo aba arabibona uko biba bimeze, ariko nyine njyewe bimbaho bwambere nahise menya ibyaribyo kuko narigaga ego hanyuma mbibwira mama nyine ampa izo padi ziramfasha murakoze ,anambwira nuburyo ngomba kwitwara .ubwo urumva umubyeyi abayumvako wakuze aba atinyako hari nizindi ngaruka zakubaho nko gutwara inda akaba yakubwira ati uramemye witwararike gutya na gutya.

Title: GS kiziguro Girls transc.docx

Doc Creator: tcuhawenimana11

Doc Date: 4/13/2023

Codes Applied: How girls experience changes occuring to them during puberty

Linked Memos: 0

Excerpt Creator: tcuhawenimana11

Excerpt Created On: 4/29/2023

Excerpt Range: 9585-10169

murakoze nimeri yanjye ni 007, imyaka ni 20, niga S6.

Kumpinduka nagiye mbona mubuzima bwanjye nyine nkimara kubona imihango habayehonyine kumva nyineko nabaye umuntu nyine nakuze ubwana nyine nabushyize hasi ,noneho ibyongibyo mbibonye mbyereka umubyeyi mama niko guhita ampa impanuro akambwira ati ubwo wabonye imihango ushobora kuba nyine wasama watwara inda nyine uramutse ukubaganye uramutse wishoye mumibonano mpuza bitsina, ubwo rero akamba hafi akampa izo padi ,kugirango nyine nigirire isuku ntaba habamo no kuba nahandurira nizindi ndwara zandurira mumyanya ndanga gitsina.

Title: GS kiziguro Girls transc.docx

Doc Creator: tcuhawenimana11

Doc Date: 4/13/2023

Codes Applied: How to take care of themselves when phsyiological changes occur for girls

Linked Memos: 0

Excerpt Creator: tcuhawenimana11

Excerpt Created On: 4/29/2023

Excerpt Range: 10017-10169

ubwo rero akamba hafi akampa izo padi ,kugirango nyine nigirire isuku ntaba habamo no kuba nahandurira nizindi ndwara zandurira mumyanya ndanga gitsina.

Title: GS kiziguro Girls transc.docx

Doc Creator: tcuhawenimana11

Doc Date: 4/13/2023

Codes Applied: How girls experience changes occuring to them during puberty

Linked Memos: 0

Excerpt Creator: tcuhawenimana11

Excerpt Created On: 4/29/2023

Excerpt Range: 10219-10544

mbere ntaramera amabere nambaraga ikanzu ikanjyamo ariko nyameze ikanzu nambaraga yanze kunjyamo, ubworero mbaza ababyei ngo nibiki byambayeyo mama nyine arambwirango nyine ubwo wakuze jya kongeresha iyo kanzu iraza kukujyamo mugatuza nyine, ndagenda ndayongeresha ariko byari byangoye nyine, nyumz nza kumenya uko babigenza.

Title: GS kiziguro Girls transc.docx

Doc Creator: tcuhawenimana11

Doc Date: 4/13/2023

Codes Applied: How girls experience changes occuring to them during puberty

Linked Memos: 0

Excerpt Creator: tcuhawenimana11

Excerpt Created On: 4/29/2023

Excerpt Range: 10648-10952

murakoze nomero yanjye ni 13 niga kukigo cya GS kiziguro, umwaka ni S1. Njywe impinduka zambayeho nukuzana ibiheri mumaso noneho ndavuga ibiheri bije gute? Nikoze mumaso numva ibiheri mumaso nibwo bwambere mbibone, mbaza ababyeyi banjye nyine barambwirango umajije gukura ugire uko ubyitwaramo, murakoze.

Title: GS kiziguro Girls transc.docx

Doc Creator: tcuhawenimana11

Doc Date: 4/13/2023

Codes Applied: How girls experience changes occuring to them during puberty

Linked Memos: 0

Excerpt Creator: tcuhawenimana11

Excerpt Created On: 4/29/2023

Excerpt Range: 11067-11268

murakoze nomero yanjye ni 014, njyewe nkimara kubona ntangiye gupfundura amabere natangiye kubyibazaho ngenda mbaza ababyeyi uko byagenze barabinsobanurira mpita mbimenya sinakongera kubyibazaho cyane.

Title: GS kiziguro Girls transc.docx

Doc Creator: tcuhawenimana11

Doc Date: 4/13/2023

Codes Applied: How girls experience changes occuring to them during puberty

Linked Memos: 0

Excerpt Creator: tcuhawenimana11

Excerpt Created On: 4/29/2023

Excerpt Range: 11347-11591

murakoze nimero yanjye ni 010, mfite imyaka 17, niga mumwaka wa kane. Njyewe nkimara kujya mumihango nabibwiye ababyeyi barambwira ngo rero aha birahindutse nyine igihe warurimo ntabwo aricyo ugezemo ugomba kugiraimyitwarire ugomba kwitwaramo

Title: GS kiziguro Girls transc.docx

Doc Creator: tcuhawenimana11

Doc Date: 4/13/2023

Codes Applied: Sources of information received during puberty

Linked Memos: 0

Excerpt Creator: tcuhawenimana11

Excerpt Created On: 4/29/2023

Excerpt Range: 11679-11708

nabibwiye umubyeyi w’umumama

Title: GS kiziguro Girls transc.docx

Doc Creator: tcuhawenimana11

Doc Date: 4/13/2023

Codes Applied: How girls experience changes occuring to them during puberty

Linked Memos: 0

Excerpt Creator: tcuhawenimana11

Excerpt Created On: 4/29/2023

Excerpt Range: 11997-12051

murakoze njyewe nkimara kubona imihango nabibwiye mama

Title: GS kiziguro Girls transc.docx

Doc Creator: tcuhawenimana11

Doc Date: 4/13/2023

Codes Applied: How girls experience changes occuring to them during puberty

Linked Memos: 0

Excerpt Creator: tcuhawenimana11

Excerpt Created On: 4/29/2023

Excerpt Range: 12206-12345

njyewe impinduka nahisengira nahise nahisee murugo barangiriye inama menya yuko ngomba kwirinda abasore cyane babandi bashora mubusambanyi.

Title: GS kiziguro Girls transc.docx

Doc Creator: tcuhawenimana11

Doc Date: 4/13/2023

Codes Applied: How girls experience changes occuring to them during puberty How to take care of themselves when phsyiological changes occur for girls

Linked Memos: 0

Excerpt Creator: tcuhawenimana11

Excerpt Created On: 4/29/2023

Excerpt Range: 12428-12686

yego, njyewe njyewe nkimara kujya mumihango nemeje yuko ntazigera nkora imibonano mpuza bitsina kugirango ntazabyara inda ntateganyije kuburyo numuhungu yansaba nkamwima simuhe, nimba ashaka ko dukundana ashaka ko tubikora nkamubwirako nkamubwira akigendera.

Title: GS kiziguro Girls transc.docx

Doc Creator: tcuhawenimana11

Doc Date: 4/13/2023

Codes Applied: How girls experience changes occuring to them during puberty

Linked Memos: 0

Excerpt Creator: tcuhawenimana11

Excerpt Created On: 4/29/2023

Excerpt Range: 13136-13553

njyewe nyine abashuti banjye bahoraga bambwira ati rero umaze gukura kandi nyine ntamuntu ujya muri segonderi adafite cher, ugomba kugira cher kugirango hagire nibintu byishi bihinduka kuri wowe, iyo umuntu afite cher haribintu byishi bihinduka, aba afite amafaranga, aba afite buri kimwe cyose ndetse yagira nicyo agusaba mukore ukabyemera “ubaza: niko abasore bakubwiraga?” abashuti banjye babakobwa bagenzi banjye.

Title: GS kiziguro Girls transc.docx

Doc Creator: tcuhawenimana11

Doc Date: 4/13/2023

Codes Applied: How girls experience changes occuring to them during puberty

Linked Memos: 0

Excerpt Creator: tcuhawenimana11

Excerpt Created On: 4/29/2023

Excerpt Range: 13672-14111

ariko nyine nkurikije ibyo tugenda twiga nubuhamya abantu beshi bagenda batanga naje gusanga nyine izo shuti ibyo zimbwira atari ukuri. Kuko rimwe narimwe abantu tujya tubona bagiye bagirwa inama nishuti zabo ati mukore iki cyangwa mwishore muribi tujya tugenda nyine tubona ingaruka ziababaho, nanjye rero niyompamvu nahise mfata umwanzuro ndavuga ati kuko ishuti zanjye ibyo bambwiye nabonye ataribyo ngomba gufata umwanzuro batazashuka.

Title: GS kiziguro Girls transc.docx

Doc Creator: tcuhawenimana11

Doc Date: 4/13/2023

Codes Applied: How girls experience changes occuring to them during puberty

Linked Memos: 0

Excerpt Creator: tcuhawenimana11

Excerpt Created On: 4/29/2023

Excerpt Range: 14438-15442

marakujya mumihango nyine numvise aribintu bitabaho numva ngizubwoba numva nibintu bitabaho yewe ndanarira kuko narindi kwa mucyecuru ntabwo narindi murugo ariko nyine naje gutaha mama arambwira ko ari ibintu bisanzwe nyine angira inama yego murungano harabakugira inama nziza hari nabakugira inama mbi rero murabo bose harangiraginama bakambwira umaze gukura jya ukura ibyinyo abagabo nabahungu nyine hahahah ,umuntu ufute amafaranga ntagucike ariko nyine njyewe izonama zose bangiraga narazumvaga ariko ntabwo ariko zose nazikurikizaga ,kuko mushuti zose ntabwo ariko bose bangiraga inama mbi hari nabangiraga inama barangiza bakambwira bati wowe icyambere nukwiga ugashaka amafaranga yawe ubwawe kuko abongabo bazizana nyine ntabwo aringombwa ngo ,cyangwa bakanavuga ngo iyo ukoze sex ibishishi byo mumaso birakira nkagenda nkabaza mama akambwira ati ibyongibyo ntabwo aribyo nibyo baba babeshya beshya ,bakambwira nibindi byinshiii najye koko nkagenda mbona ko ataribyo nyine nkabireha eeeh murakoze.

Title: GS kiziguro Girls transc.docx

Doc Creator: tcuhawenimana11

Doc Date: 4/13/2023

Codes Applied: How girls experience changes occuring to them during puberty

Linked Memos: 0

Excerpt Creator: tcuhawenimana11

Excerpt Created On: 4/29/2023

Excerpt Range: 15488-15999

murakoze, njyewe nkimara kumera amabere naragiye mbibwira umwana twigana ,arambwira ngo ubwo wameze amabere wakuze ndamubwira atise iyo umuntu afite amabere aba yakuze arambwira ngo yigo noneho hashize niminsi kwishuri turabyiga abatubwira uko tugomba kwitwarako umwana ugeze kumyaka 12 kuzamura aba yajya no mumihano kandi akaba yakora imibonanao mpuza bitsina agasama ,batugira inama rero yo kubyirinda ubwo natwe turavuga ko ntamuntu ugomba kudushuka tugomba kwirinda gukora imibonano mpuzabitsina, murakoze.

Title: GS kiziguro Girls transc.docx

Doc Creator: tcuhawenimana11

Doc Date: 4/13/2023

Codes Applied: How girls experience changes occuring to them during puberty

Linked Memos: 0

Excerpt Creator: tcuhawenimana11

Excerpt Created On: 4/29/2023

Excerpt Range: 16091-16352

murakoze njyewe nkimera amabere kuko nari muto nagizengo wenda ni ibise kuko narababaraga cyane nkagirango nyine ndavuga wenda ndayarwaye uburwiyi nti buriya wenda azakira, nkajya ngendambaza abantu bakambwira nyine wakuze nyine namabere wazanye mbimenya gutyo.

Title: GS kiziguro Girls transc.docx

Doc Creator: tcuhawenimana11

Doc Date: 4/13/2023

Codes Applied: How to take care of themselves when phsyiological changes occur for girls

Linked Memos: 0

Excerpt Creator: tcuhawenimana11

Excerpt Created On: 4/29/2023

Excerpt Range: 16387-16443

ati hoya ntashutse barambwiye bati nyine nuko bitangira.

Title: GS kiziguro Girls transc.docx

Doc Creator: tcuhawenimana11

Doc Date: 4/13/2023

Codes Applied: How girls experience changes occuring to them during puberty

Linked Memos: 0

Excerpt Creator: tcuhawenimana11

Excerpt Created On: 4/29/2023

Excerpt Range: 16474-17743

murakoze nkimara kujya mumihango nyine rimwe narimwe nababaraga munda ugasanga ndababara cyane nyine mbaza bagenzi bajye nyine baransobanurira, bamwe bakambwira ngo kugirango ugabanye kuribwa munda nyine nuko wakora sex, hanyuma nza kubaza nundi mugenzi wanjye nti ese wowe ko ujya ujya mumihango wowe ujya ubabara munda? Arambwira ati njyewe ntabwio mbabara ati ariko hakurya harukundi kuntu wabigenza, ndamubaza nti ese nabigenza gute ngo munda hajye hareka kundya? Noneho arambwira ati ushobora kujya nko kwa muganga ukabitekerereza muganga akaguha nkibinini bigabanya ububabare mugihe wumva wababaye, ariko nyine muribyo byose iyo umuntu amaze gukura utangira kubona ishuti zabahungu bakagusaba urukundo nyine nizabagabo bakagusaba urukundo rimwe narimwe bakagushukisha ayomafaranga bakubwira bati urimwizaaa turyamanye ntakibazooo ibintu nkibyo ,ariko nawe nyine kuberako uba ufite izompunjyenjye ukabaza nabayeyi uti eseko bajyenda bambwira gutya nagutya ngo barashaka ko turyamana nakora iki? U mubyiyi nawe akaguha impanuro akakubwira ati ntabwo ari byiza kuba wakora sex ahubwo rindira igihe cyawe kizagera akakubwira ati ushobora gukora iyo mibonano usanga utwaye inda imburagihe cyangwase ukaba wahandurira imitezi, mburugu, sida nizindindwara zose murakoze.

Title: GS kiziguro Girls transc.docx

Doc Creator: tcuhawenimana11

Doc Date: 4/13/2023

Codes Applied: How to take care of themselves when phsyiological changes occur for girls

Linked Memos: 0

Excerpt Creator: tcuhawenimana11

Excerpt Created On: 4/29/2023

Excerpt Range: 16796-17743

Arambwira ati njyewe ntabwio mbabara ati ariko hakurya harukundi kuntu wabigenza, ndamubaza nti ese nabigenza gute ngo munda hajye hareka kundya? Noneho arambwira ati ushobora kujya nko kwa muganga ukabitekerereza muganga akaguha nkibinini bigabanya ububabare mugihe wumva wababaye, ariko nyine muribyo byose iyo umuntu amaze gukura utangira kubona ishuti zabahungu bakagusaba urukundo nyine nizabagabo bakagusaba urukundo rimwe narimwe bakagushukisha ayomafaranga bakubwira bati urimwizaaa turyamanye ntakibazooo ibintu nkibyo ,ariko nawe nyine kuberako uba ufite izompunjyenjye ukabaza nabayeyi uti eseko bajyenda bambwira gutya nagutya ngo barashaka ko turyamana nakora iki? U mubyiyi nawe akaguha impanuro akakubwira ati ntabwo ari byiza kuba wakora sex ahubwo rindira igihe cyawe kizagera akakubwira ati ushobora gukora iyo mibonano usanga utwaye inda imburagihe cyangwase ukaba wahandurira imitezi, mburugu, sida nizindindwara zose murakoze.

Title: GS kiziguro Girls transc.docx

Doc Creator: tcuhawenimana11

Doc Date: 4/13/2023

Codes Applied: How girls experience changes occuring to them during puberty

Linked Memos: 0

Excerpt Creator: tcuhawenimana11

Excerpt Created On: 4/29/2023

Excerpt Range: 17824-18153

murakoze, nimero yanjye 13 niga mukigo cya GS kiziguro umwaka S1A, mfite imyaka 16. Njyewe bwambere nyine narinziko bwambere umwana ajya mumihango afite imyaka 12,13 gutyo, njyewe ngeze 12 ndategereza ndibaza njyewe ko ntatayijyamo, noneho mbaza ababyeyi bati ntabwo ibyo ngibyo aribyo bagenderaho na 15,16 bayijyamo ndategereza.

Title: GS kiziguro Girls transc.docx

Doc Creator: tcuhawenimana11

Doc Date: 4/13/2023

Codes Applied: Received information is misleading and risky

Linked Memos: 0

Excerpt Creator: tcuhawenimana11

Excerpt Created On: 4/29/2023

Excerpt Range: 18216-18652

murakoze njyewe ikintu numva cyambereye ikibazo kera narinzi yuko umukobwa iyo ari gupfundura amabere kugirango atamurya cyangwa ngo aze neza Harabana bishuti yanjye kera bajyaga bajya kumugezi bagafata udukoko two mumazi bita inyagaruzi bakavuga ngo iyo udushyizeho amabere ameraneza bituma atakurya kandi akaba Manini kandi ukaba inkumi njyewe ndabaza nsanga ataribyo amabere arizana uretseko njyewe ntatwo nashyizeho naranadutinyaga.

Title: GS kiziguro Girls transc.docx

Doc Creator: tcuhawenimana11

Doc Date: 4/13/2023

Codes Applied: Pshcological changes occuring during puberty-Girls

Linked Memos: 0

Excerpt Creator: tcuhawenimana11

Excerpt Created On: 4/29/2023

Excerpt Range: 18951-19367

wenda wenda ahongaho kuba wajya mumihango ugakenera nkishuti yawe y’umuhungu wnda sinavugango bikunda kumbaho ariko harabantu beshi bikunda kubaho ugasanga wenda akavuga ati njyewe mugihe ndimo ndumva nkeneye ishuti nka cher wanjye wenda kurubwo buryo “ubaza ati ubyitwaramo gute kuko nicyo gihe kibi” iyo ninjiye muricyo gihe nyine umuntu aba afite feeling nkamarangamutima nyine nkimyiyumvire sinzukuntu nabivuga ,

Title: GS kiziguro Girls transc.docx

Doc Creator: tcuhawenimana11

Doc Date: 4/13/2023

Codes Applied: Perceptions about the use of condom during sex

Linked Memos: 0

Excerpt Creator: tcuhawenimana11

Excerpt Created On: 4/29/2023

Excerpt Range: 19367-19609

nyine ibyiyumviro byumubiri nyine icyo gihe byaba byaje harukuntu nkishuti yawe yaza mukaganira ariko akaba yakubwira ngo mukore sex bikaba ngombwa ko nyine wenda ukamubwira uti uti tuyikore nyine washaka nko kwirinda ugakoresha agakingirizo.

Title: GS kiziguro Girls transc.docx

Doc Creator: tcuhawenimana11

Doc Date: 4/13/2023

Codes Applied: How to take care of themselves when phsyiological changes occur for girls How girls experience changes occuring to them during puberty

Linked Memos: 0

Excerpt Creator: tcuhawenimana11

Excerpt Created On: 4/29/2023

Excerpt Range: 19818-20335

murakoze, njywewe izo feeling zije ntabwo numvako shobora kubikora gusa nyine harabonzi babikora arikonjye “ubaza ati tubwire experience yawe itariyabandi “ njyewe nyine biraza pe ariko mukuza ngira uko mbyitwaramo “ ubaza ati watubwira murimacye uko witwara wenda ukaba uhamagara kuri phone cyangwa ishuti yanjye itanta muri risk wenda hankigihe “ njyewe kubyitwaramo iyo bije mbona atarintu bitwara igihe kinin kuburyo kubyihanganira byananira ndihangana bikarangira ntabwo nafatiraho ngo ngomba kubikora murakoze.

Title: GS kiziguro Girls transc.docx

Doc Creator: tcuhawenimana11

Doc Date: 4/13/2023

Codes Applied: How girls experience changes occuring to them during puberty

Linked Memos: 0

Excerpt Creator: tcuhawenimana11

Excerpt Created On: 4/29/2023

Excerpt Range: 20381-20592

murakoze, njyewe icyo gihe iyo kije ndirinda nkirinda guhura numuhungu nkajye nko murugo nkirinda kumva amagambo ambwira Atari meza ashingiye mugukora imibonano mpuza bitsina nkirinda kuba nahura nawe murakoze.

Title: GS kiziguro Girls transc.docx

Doc Creator: tcuhawenimana11

Doc Date: 4/13/2023

Codes Applied: Received information is misleading and risky

Linked Memos: 0

Excerpt Creator: tcuhawenimana11

Excerpt Created On: 4/29/2023

Excerpt Range: 20601-20740

murakoze nimere yanjye ni 13 ,njyewe nkizana ibishishi umuntu umukobwsa wishuti yanjye nyine yarambwiye ngo ibyongibyo bikizwa nabahungu,

Title: GS kiziguro Girls transc.docx

Doc Creator: tcuhawenimana11

Doc Date: 4/13/2023

Codes Applied: How girls experience changes occuring to them during puberty

Linked Memos: 0

Excerpt Creator: tcuhawenimana11

Excerpt Created On: 4/29/2023

Excerpt Range: 20602-21260

murakoze nimere yanjye ni 13 ,njyewe nkizana ibishishi umuntu umukobwsa wishuti yanjye nyine yarambwiye ngo ibyongibyo bikizwa nabahungu, ndagenda mbaza undiwundi wishutiyanjye arambwira ngo vayo muri club ibyo ngibyo uzabyigiramo ndagenda turabyiga batubwira bati ibyo ngibyo ntabwo bikizwa nabahungu ahubwo birikiza “ ubaza ati twashakaga kubaza kubigendaye no gushaka abahungu sex feeling how do you react ubugenza gute nigute wi protect how do you protect yourself , because sexual feeling that you have is a part of physiological change so that’s natural how do you react, ubigenza gute urahunga wirinda centre yabahungu tubwire?” Njyewe nguma murugo.

Title: GS kiziguro Girls transc.docx

Doc Creator: tcuhawenimana11

Doc Date: 4/13/2023

Codes Applied: Received information is right and provides sufficient information helping boys and girls during puberty

Linked Memos: 0

Excerpt Creator: tcuhawenimana11

Excerpt Created On: 4/29/2023

Excerpt Range: 20741-20921

ndagenda mbaza undiwundi wishutiyanjye arambwira ngo vayo muri club ibyo ngibyo uzabyigiramo ndagenda turabyiga batubwira bati ibyo ngibyo ntabwo bikizwa nabahungu ahubwo birikiza

Title: GS kiziguro Girls transc.docx

Doc Creator: tcuhawenimana11

Doc Date: 4/13/2023

Codes Applied: How girls experience changes occuring to them during puberty

Linked Memos: 0

Excerpt Creator: tcuhawenimana11

Excerpt Created On: 4/29/2023

Excerpt Range: 21551-22072

murakoze kubitekerezo byiza mwazatwongereraho utundi.

Undi ati: murakoze njyewe iyo ibyo bije mbasha kwiyumvishako byazangiraho ingaruka mugihe kizaza ugahita ushaka nkintego wazajya ukoresha ukavuga uti ninzajya niyumvushamo ibibitekerezo bizajya bituma njya gshaka abo bahungu ariko pe numubiri biriza ariko ukoresha intego wihaye ukavugati niba wumvayuko usaka umuhungu igihe kizaza bizakugirahpo ingaruka, nimba iri kwiga ugahita utekereza baguteye inda amashuri ukayasubika bigatuma nyine ushaka uburyo ubyitwaramo.

Title: GS kiziguro Girls transc.docx

Doc Creator: tcuhawenimana11

Doc Date: 4/13/2023

Codes Applied: Information that girls need

Linked Memos: 0

Excerpt Creator: tcuhawenimana11

Excerpt Created On: 4/29/2023

Excerpt Range: 22406-22834

murakoze, umwana w’umwangavu amakuru akenye kuba yahabwa harimo nyine kwirinda abahungu kugirango batamushora mubusambanyi, ikindinharimo kuba nyine yatanga amakuru igihe habayeho nkumuntu ushaka kumuhohotera, ikindi andi makuru umwana wumukobwa akenera ninko kuba yaganirizwa kumihindagurikire y’ubuzima kubo bishobora gutuma nabyo byamuzanira ingaruka mubuzima bwiwe nko gutwara izonda, kwandura Sida nizindi ngaruka murakoze.

Title: GS kiziguro Girls transc.docx

Doc Creator: tcuhawenimana11

Doc Date: 4/13/2023

Codes Applied: Information that girls need

Linked Memos: 0

Excerpt Creator: tcuhawenimana11

Excerpt Created On: 4/29/2023

Excerpt Range: 23002-23422

murakoze, ababyeyibe baba bagomba kumwegera bakamuganiriza uko yakwitwara mugihe yagezemo nyine bakamubwirako niba ageze mugihe kimihango agomba kwirinda abahungu kandi akumvako nawe nyine igihe agezemo ibyo ababyeyibe bamubwiye agomba kubikurikiza ubundi yamara bakamuganiriza nyine ibyongibyo byubuzima bw’imyororokere, ababyeyibe nabo baba babibonako umwana wabo aba yakuze kandi nawe akagomba kubyubahiriza murakoze.

Title: GS kiziguro Girls transc.docx

Doc Creator: tcuhawenimana11

Doc Date: 4/13/2023

Codes Applied: Information that girls need

Linked Memos: 0

Excerpt Creator: tcuhawenimana11

Excerpt Created On: 4/29/2023

Excerpt Range: 23518-23730

murakoze, amakuru y’ingenzi umwana w’umwangavu akenera, nuko ababyeyibe bamuganiriza kkandi akamenya amakuru yingenzi “ubaza ati ayingenzi niyo dushaka nayahe” kumubwiza ukuri amakuru yubuzima bw’imyororokere.

Title: GS kiziguro Girls transc.docx

Doc Creator: tcuhawenimana11

Doc Date: 4/13/2023

Codes Applied: Information that girls need

Linked Memos: 0

Excerpt Creator: tcuhawenimana11

Excerpt Created On: 4/29/2023

Excerpt Range: 23785-24095

murakoze, andi makuru yingenzi umukobwa akenera agomba kumenyako hari nabagabo bashuka abakobwa nkaka rifuti akamutwara ugasanga aramufashe, ikindi agomba kwirinda umukobwa nkigihe aba yafashwe kungufu ntaabiceceka agahita abibwira ababyeyibe bagahita abamujyana kwamuganga kuko harigihe byamugiraho ingaruka.

Title: GS kiziguro Girls transc.docx

Doc Creator: tcuhawenimana11

Doc Date: 4/13/2023

Codes Applied: Information that girls need

Linked Memos: 0

Excerpt Creator: tcuhawenimana11

Excerpt Created On: 4/29/2023

Excerpt Range: 24113-24242

Andi makuru umukobwa akenera mugihe ageze mugihe cy’ubwanagavu, ashobora kuba atazi uko azakoresha ibikoresho by’isuku azakenera.

Title: GS kiziguro Girls transc.docx

Doc Creator: tcuhawenimana11

Doc Date: 4/13/2023

Codes Applied: Information that boys need

Linked Memos: 0

Excerpt Creator: tcuhawenimana11

Excerpt Created On: 4/29/2023

Excerpt Range: 24330-24712

murakoze, umuhungu amakuru agomba kumenya nukwitwararika neza nyine igihe ari kumwe numukobwa mbese akirinda kujya mungeso mbi nko kumywa inzoga cyangwa se agatabi kugirango bitamushora muri bwa busambayi bikaba byamuviramo no kwandura indwara zo mumyanya ndanga bitsina nkokuba yakwandura izoza mburugu, imitezi, sida cyangwase nizindi ndwara zose zo mumyanya myibarukiro murakoze.

Title: GS kiziguro Girls transc.docx

Doc Creator: tcuhawenimana11

Doc Date: 4/13/2023

Codes Applied: Sources of information received during puberty

Linked Memos: 0

Excerpt Creator: tcuhawenimana11

Excerpt Created On: 4/29/2023

Excerpt Range: 24999-25159

murakoze, amakuru mfite ayambere nayakuye kw’ishuri, andi nababyeyi bagenda batuganiriza kubijyanye nubuzima bw’imyororokere ndetse no kumaradiyo Na television.

Title: GS kiziguro Girls transc.docx

Doc Creator: tcuhawenimana11

Doc Date: 4/13/2023

Codes Applied: Sources of information received during puberty

Linked Memos: 0

Excerpt Creator: tcuhawenimana11

Excerpt Created On: 4/29/2023

Excerpt Range: 25227-25335

ayomfite nayakuye kuri mugenzi wanjye ayandi ni kunnshuti zanjye andi ni kuri trainings umuntu agenda abona.

Title: GS kiziguro Girls transc.docx

Doc Creator: tcuhawenimana11

Doc Date: 4/13/2023

Codes Applied: Sources of information received during puberty

Linked Memos: 0

Excerpt Creator: tcuhawenimana11

Excerpt Created On: 4/29/2023

Excerpt Range: 25387-25440

njyewe amakuru mfite ahandi nayakuye ni kwa muganga.

Title: GS kiziguro Girls transc.docx

Doc Creator: tcuhawenimana11

Doc Date: 4/13/2023

Codes Applied: Sources of information received during puberty

Linked Memos: 0

Excerpt Creator: tcuhawenimana11

Excerpt Created On: 4/29/2023

Excerpt Range: 25478-25641

njyewe ahantu nayakuye amwe namwe nayakuye muma clubs yabana tuba dufite abantu bagenda bayatugezaho

Undi ati njyewe amakuru mfite nayakuye kucyumba cy’urubyiruko.

Title: GS kiziguro Girls transc.docx

Doc Creator: tcuhawenimana11

Doc Date: 4/13/2023

Codes Applied: Sources of information received during puberty

Linked Memos: 0

Excerpt Creator: tcuhawenimana11

Excerpt Created On: 4/29/2023

Excerpt Range: 25785-25998

hari nkukuntu nyine ujya nko kuri google ukabaza nyine hari nkukuntu uba wabyumvishije abakobwa bajya mumihango bakagusobanurira byose nuko ugomba kwitwara, ayo makuru tuya sachinga kuri google nyine naza yutube.

Title: GS kiziguro Girls transc.docx

Doc Creator: tcuhawenimana11

Doc Date: 4/13/2023

Codes Applied: Obstacles and risks experienced by girls during the sexual and reproductive health changes occuring during puberty and adolescence

Linked Memos: 0

Excerpt Creator: tcuhawenimana11

Excerpt Created On: 4/29/2023

Excerpt Range: 26358-26508

murakoze, ingorane abana babakobwa bahuranazo mugihe cy’ubwanagavu nigihe Babura ababagira inama, kubura ibikoresho by’isuku mugihe yagiye mumihango.

Title: GS kiziguro Girls transc.docx

Doc Creator: tcuhawenimana11

Doc Date: 4/13/2023

Codes Applied: Obstacles and risks experienced by girls during the sexual and reproductive health changes occuring during puberty and adolescence

Linked Memos: 0

Excerpt Creator: tcuhawenimana11

Excerpt Created On: 4/29/2023

Excerpt Range: 26547-26775

murakoze, izindi ngorane ninkokuba nyine watwaye nk’inda noneho nkababyeyi bawe nyine bakakwamagana cyangwase wagenda nko kumuntu waguteye iyonda umwna akamwihakana akakubwirako akaguha nyine inzira nyine yagufasha mukayikuramo.

Title: GS kiziguro Girls transc.docx

Doc Creator: tcuhawenimana11

Doc Date: 4/13/2023

Codes Applied: Obstacles and risks experienced by girls during the sexual and reproductive health changes occuring during puberty and adolescence

Linked Memos: 0

Excerpt Creator: tcuhawenimana11

Excerpt Created On: 4/29/2023

Excerpt Range: 26807-26997

ikindi nakongeraho nyine umwna w’umwangavu ahuranazo, ingorane navuga iyo ahise muricyo kiciro ninkabahungu cyangwase abagabo bamutesha umutwe nayo ningorane, baramustesa bamutesha umutwe.

Title: GS kiziguro Girls transc.docx

Doc Creator: tcuhawenimana11

Doc Date: 4/13/2023

Codes Applied: Obstacles and risks experienced by girls during the sexual and reproductive health changes occuring during puberty and adolescence

Linked Memos: 0

Excerpt Creator: tcuhawenimana11

Excerpt Created On: 4/29/2023

Excerpt Range: 27006-27235

ingorane nyishi bazivuze bakunda guhura ningorane zo gufatwa kungufu ugasanga nyine nimba abuze ibikoresho byisuku ugasanga aravuze ati nasambana arabona amafaranga abigure ugasanaga atway inda, ndumva arizo ngorane yahura nazo.

Title: GS kiziguro Girls transc.docx

Doc Creator: tcuhawenimana11

Doc Date: 4/13/2023

Codes Applied: Obstacles and risks experienced by girls during the sexual and reproductive health changes occuring during puberty and adolescence

Linked Memos: 0

Excerpt Creator: tcuhawenimana11

Excerpt Created On: 4/29/2023

Excerpt Range: 27245-27339

izindi ngorane abana babakobwa bahura nazo nukaba bakorerwa ihohoterwa ntibakorerwe ubuvugizi.

Title: GS kiziguro Girls transc.docx

Doc Creator: tcuhawenimana11

Doc Date: 4/13/2023

Codes Applied: Obstacles and risks experienced by girls during the sexual and reproductive health changes occuring during puberty and adolescence

Linked Memos: 0

Excerpt Creator: tcuhawenimana11

Excerpt Created On: 4/29/2023

Excerpt Range: 27349-27518

izindi ngaruka bahura nazo nukutakira ingaruka zababayeho cyangwase kutakira ibibazo bahuye nabyo muriryo sambana ugasanga nkumwana w’umukobwa yivukije ubuzima murakoze.

Title: GS kiziguro Girls transc.docx

Doc Creator: tcuhawenimana11

Doc Date: 4/13/2023

Codes Applied: Obstacles and risks experienced by girls during the sexual and reproductive health changes occuring during puberty and adolescence

Linked Memos: 0

Excerpt Creator: tcuhawenimana11

Excerpt Created On: 4/29/2023

Excerpt Range: 27528-27750

izindi ngaruka umwana wumukobwa yahuranazo nko mugihe atabonye amakuru ahagije numva njyewe igihe atabonye amakuru ahagije yimihindagurikire yumubiri igihe yamugezeho akumvako yasebye imbere yabandi cyangwa ntajye mubandi.

Title: GS kiziguro Girls transc.docx

Doc Creator: tcuhawenimana11

Doc Date: 4/13/2023

Codes Applied: Obstacles and risks experienced by girls during the sexual and reproductive health changes occuring during puberty and adolescence

Linked Memos: 0

Excerpt Creator: tcuhawenimana11

Excerpt Created On: 4/29/2023

Excerpt Range: 27760-27875

murakoze, igihe umwana wumukobwa ahura nibibazo cyangwa ningorane bishobora gutuma agira ipfunwe ryo kujya mubandi.

Title: GS kiziguro Girls transc.docx

Doc Creator: tcuhawenimana11

Doc Date: 4/13/2023

Codes Applied: Barriers leading to the risks and obstacles girls and boys experience during puberty and adolescence

Linked Memos: 0

Excerpt Creator: tcuhawenimana11

Excerpt Created On: 4/29/2023

Excerpt Range: 27885-28021

izindi ngorane umwana w’umukobwa ashobora guhura nazo ninko kuba umubyiyi wawe ataguha amakuru kugihe ugasanga numwana no kwiga ntiyiga.

Title: GS kiziguro Girls transc.docx

Doc Creator: tcuhawenimana11

Doc Date: 4/13/2023

Codes Applied: Obstacles and risks experienced by boys during the sexual and reproductive health changes occuring during puberty and adolescence

Linked Memos: 0

Excerpt Creator: tcuhawenimana11

Excerpt Created On: 4/29/2023

Excerpt Range: 28135-28355

kumuhungu navuga ko iyo amajije kuba nkingimbi akeshi nkababyeyibe harigihe bamuvunisha bakamubwira bati wowe utangiye gukura ukeneye gukora ibintu ibi nibi ugasanga nyine baramubwira atangire gufata nkishingano nyine.

Title: GS kiziguro Girls transc.docx

Doc Creator: tcuhawenimana11

Doc Date: 4/13/2023

Codes Applied: Obstacles and risks experienced by boys during the sexual and reproductive health changes occuring during puberty and adolescence

Linked Memos: 0

Excerpt Creator: tcuhawenimana11

Excerpt Created On: 4/29/2023

Excerpt Range: 28560-29040

umwana wumuhungu igihe atabonye amakuru ahagije harigihe yaba agabwe akora imibonano mpuza bitsina ariko atazi cyangwa yumva yikinira bimwe byabana yaba atarasobanuriwe imihindagurikire ye yumubiri byaba byaranamugezeho ntabyiteho ugasanga ateye umukobwa wabandi inda azingo aracyari gukina ibyabana agahura ningaruka nko kumufunga cyangwa agacikiriza amashuri ugasanga nkababyeyi be bamuhaye ishingano bati urarera umwana wawe numugore wawe ushake naho mubana ikindi bakagufunga.

Title: GS kiziguro Girls transc.docx

Doc Creator: tcuhawenimana11

Doc Date: 4/13/2023

Codes Applied: Barriers leading to the risks and obstacles girls and boys experience during puberty and adolescence

Linked Memos: 0

Excerpt Creator: tcuhawenimana11

Excerpt Created On: 4/29/2023

Excerpt Range: 28560-29040

umwana wumuhungu igihe atabonye amakuru ahagije harigihe yaba agabwe akora imibonano mpuza bitsina ariko atazi cyangwa yumva yikinira bimwe byabana yaba atarasobanuriwe imihindagurikire ye yumubiri byaba byaranamugezeho ntabyiteho ugasanga ateye umukobwa wabandi inda azingo aracyari gukina ibyabana agahura ningaruka nko kumufunga cyangwa agacikiriza amashuri ugasanga nkababyeyi be bamuhaye ishingano bati urarera umwana wawe numugore wawe ushake naho mubana ikindi bakagufunga.

Title: GS kiziguro Girls transc.docx

Doc Creator: tcuhawenimana11

Doc Date: 4/13/2023

Codes Applied: Obstacles and risks experienced by boys during the sexual and reproductive health changes occuring during puberty and adolescence

Linked Memos: 0

Excerpt Creator: tcuhawenimana11

Excerpt Created On: 4/29/2023

Excerpt Range: 29050-29173

izindi ngorane ninkuko umuhungu yaba yaranize ijwi akaba afite isoni zo kuvuga mubandi nyine ngo atumvikana iryojwi yanize.

Title: GS kiziguro Girls transc.docx

Doc Creator: tcuhawenimana11

Doc Date: 4/13/2023

Codes Applied: Obstacles and risks experienced by boys during the sexual and reproductive health changes occuring during puberty and adolescence

Linked Memos: 0

Excerpt Creator: tcuhawenimana11

Excerpt Created On: 4/29/2023

Excerpt Range: 29183-29409

murakoze umuhungu iyo ageze mugihe cy’ubugimbi atangira kwiyumvako ari umusore agahura nishutize zigatangira kumujyana mubusambanyi no mubiyobyabwenge ugasanga acikirije amashuri akiri muto bikamwangiriza ubuzima bwe murakoze.

Title: GS kiziguro Girls transc.docx

Doc Creator: tcuhawenimana11

Doc Date: 4/13/2023

Codes Applied: Barriers leading to the risks and obstacles girls and boys experience during puberty and adolescence

Linked Memos: 0

Excerpt Creator: tcuhawenimana11

Excerpt Created On: 4/29/2023

Excerpt Range: 29685-29852

kubura amakuru umwana nyine niba aba wenyine ntababyeyi afite umugira inama wese kuberako ababyeyi bagira abana babo inama yubuzima, nyine kubura amakuru yubuzima bwe

Title: GS kiziguro Girls transc.docx

Doc Creator: tcuhawenimana11

Doc Date: 4/13/2023

Codes Applied: Barriers leading to the risks and obstacles girls and boys experience during puberty and adolescence

Linked Memos: 0

Excerpt Creator: tcuhawenimana11

Excerpt Created On: 4/29/2023

Excerpt Range: 29853-30100

Undi ati ikindi nyine gishobora guterwa na poor family nyine nicyu numva gishobora kuba inzitizi kubangavu n’ingimbi.

Undi ati ikindi bishobora guterwa nishuti mbi cyangwa kuba ababyeyibe bataramuhaye information zifatika kubijyanye nimyororokere.

Title: GS kiziguro Girls transc.docx

Doc Creator: tcuhawenimana11

Doc Date: 4/13/2023

Codes Applied: Suggestions to improve SRH during puberty and adolescence by girls

Linked Memos: 0

Excerpt Creator: tcuhawenimana11

Excerpt Created On: 4/29/2023

Excerpt Range: 30396-30636

murakoze, icyo nakongeraho kubijyanye nimyigishirize kubijyanye nubuzima bwi imyororokere, dukwiye kubaha no kumva ababyeyi mubyo baba batubwira nicyo nakongeraho no kugirango bongere imbaraga mukutwigisha ubuzima bw’imyororokere murakoze.

Title: GS kiziguro Girls transc.docx

Doc Creator: tcuhawenimana11

Doc Date: 4/13/2023

Codes Applied: Suggestions to improve SRH during puberty and adolescence by girls

Linked Memos: 0

Excerpt Creator: tcuhawenimana11

Excerpt Created On: 4/29/2023

Excerpt Range: 30714-30898

murakoze, nukuvuga ngo abantu bose ntabwo bamera nkababyeyi bawe, harigihe ubona numubyeyi wawe nawe ntakuntu ameze ariko harigihe uba ufite ishuti, rero wegera ishuti zawe vukababaza.

Title: GS kiziguro Girls transc.docx

Doc Creator: tcuhawenimana11

Doc Date: 4/13/2023

Codes Applied: Suggestions to improve SRH during puberty and adolescence by girls

Linked Memos: 0

Excerpt Creator: tcuhawenimana11

Excerpt Created On: 4/29/2023

Excerpt Range: 30908-31096

njyewe icyo nakongeraho kwamuganga hari icyumba gihari bakoreramo ubuzima bw’imyororokere rero byagucanze wabuze uko ubigenza wajya kwa muganga bakakugira inama zimihindagurikire yumubiri.

Title: GS kiziguro Girls transc.docx

Doc Creator: tcuhawenimana11

Doc Date: 4/13/2023

Codes Applied: Precautions to take during puberty for girls

Linked Memos: 0

Excerpt Creator: tcuhawenimana11

Excerpt Created On: 4/29/2023

Excerpt Range: 31098-31330

Undi ati murakoze nanjye ikindi nakongeraho nukwirinda nyina kwemera amakuru yose ubonye kuko nyine rimwe narimwe haba harimo atariyo bikaba byiza ko wabanza kugisha ababyeyi bawe inma kugirango wumve neza ko ibyo bakubwira arukuri.

Title: GS kiziguro Girls transc.docx

Doc Creator: tcuhawenimana11

Doc Date: 4/13/2023

Codes Applied: Suggestions to improve SRH during puberty and adolescence by girls

Linked Memos: 0

Excerpt Creator: tcuhawenimana11

Excerpt Created On: 4/29/2023

Excerpt Range: 31331-31517

Undi ati icyo nakongeraho hakongerwa amahugurwa yaba kubabyeyi no kubana babo abakobwa cyangwa abahungu kugirango babashe kuba bagira amakuru ahamye kandi yukuri uko bakwitwara murakoze.

Title: G S RWAMAGANA Girls TRANSCRIBED.docx

Doc Creator: tcuhawenimana11

Doc Date: 3/25/2023

Codes Applied: Physiological changes occuring during puberty-Boys

Linked Memos: 0

Excerpt Creator: tcuhawenimana11

Excerpt Created On: 4/28/2023

Excerpt Range: 215-534

Ndi nimero ya mbere mfite imyaka 14 niga mu wa mbere. Umuhungu atangira kwiroteraho, akamera incakwaha, akamera insya, agatangira no guhindura amwe mu miyumviro ye. Uravuze ngo atangira no guhindura amwe mu miyumviro ye, ayo miyumviro ninkayahe? Atangira kumva atangiye kuba undi umuntu nka gute? Nko gukunda umukobwa.

Title: G S RWAMAGANA Girls TRANSCRIBED.docx

Doc Creator: tcuhawenimana11

Doc Date: 3/25/2023

Codes Applied: Physiological changes occuring during puberty-Boys

Linked Memos: 0

Excerpt Creator: tcuhawenimana11

Excerpt Created On: 4/28/2023

Excerpt Range: 659-838

Nimero yange ni 5 mfite imyaka 16 niga muwa mbere. Ikindi nyine umuhungu aba yumva yagira uruhare runini mu rugo haba wenda nko kugisha inama akumva niwe wabijyamo cyane murakoze.

Title: G S RWAMAGANA Girls TRANSCRIBED.docx

Doc Creator: tcuhawenimana11

Doc Date: 3/25/2023

Codes Applied: Psychological changes occuring during puberty-Boys

Linked Memos: 0

Excerpt Creator: tcuhawenimana11

Excerpt Created On: 4/28/2023

Excerpt Range: 890-1195

Nimero yange ni 7 mfite imyaka 19 niga muwa gatanu. Ikindi numva umuhungu yagira mu mpinduka ze igihe yatangira kuba ingimbi atangira kumva yubashwye akumva ko ahantu hose yaba ari yaba yubashwye wenda nko mu rugo akumva ko niba afite bashiki be. Ibyo bagiye gukora byose babanza kumugisha inama murakoze.

Title: G S RWAMAGANA Girls TRANSCRIBED.docx

Doc Creator: tcuhawenimana11

Doc Date: 3/25/2023

Codes Applied: Physiological changes occuring during puberty-Boys

Linked Memos: 0

Excerpt Creator: tcuhawenimana11

Excerpt Created On: 4/28/2023

Excerpt Range: 1290-1442

Murakoze ndi nimero 9 mfite imyaka 17 niga mu mwaka wa gatanu w’amashuri yisumbuye. Iyo umuhungu atangiye kugera mu gihe cyingimbi atangira kuniga ijwi,

Title: G S RWAMAGANA Girls TRANSCRIBED.docx

Doc Creator: tcuhawenimana11

Doc Date: 3/25/2023

Codes Applied: Psychological changes occuring during puberty-Boys

Linked Memos: 0

Excerpt Creator: tcuhawenimana11

Excerpt Created On: 4/28/2023

Excerpt Range: 1443-1881

atangira kumva yafata inshingano zo mu rugo akagira ibyo afasha ababyeyi, atangira kumva hari uruhare yagira mwiterambere rya family cyangwa mu rugo muri rusange, atangira ko nawe ubwe hari ibitekerezo yakwigirira akaba afasha ababyeyi wenda nko kwishakira amakayi, gushaka akazi ko muri karitsiye wenda nko gushaka akazi ko kubaka, kwasa inkwi z’abantu, kuvomera abantu wenda agashaka nkigare akajya atunda amazi gutyo mu cyaro murakoze.

Title: G S RWAMAGANA Girls TRANSCRIBED.docx

Doc Creator: tcuhawenimana11

Doc Date: 3/25/2023

Codes Applied: Boys and girls know about the causes leading to the physiological changes during puberty

Linked Memos: 0

Excerpt Creator: tcuhawenimana11

Excerpt Created On: 4/28/2023

Excerpt Range: 2022-2505

Impinduka ziterwa wenda nimyaka aba agezemo kuko hari icyiciro kimwe aba avuyemo nicyo aba agezemo, aba avuye mu bwana agiye mubugimbi nyine hari ibyo aba abona bakuru be bakoze cyangwa abaturanyi be akavuga ati nange ngomba gutera ikirenge mu cyabo. Akavuga ati wenda niba mfite mukuru wange akaba yarakuze mbona ashakisha amafaranga mu gihe murugo haba hari ubukene cyangwa batishoboye nawe akavuga ati ngomba kwirwanaho bigaterwa nigihe agezemo nyine guhinduka kumubiri. murakoze.

Title: G S RWAMAGANA Girls TRANSCRIBED.docx

Doc Creator: tcuhawenimana11

Doc Date: 3/25/2023

Codes Applied: Physiological changes occuring during puberty-Girls

Linked Memos: 0

Excerpt Creator: tcuhawenimana11

Excerpt Created On: 4/28/2023

Excerpt Range: 2743-2983

Ndi nimero ya mbere mfite imyaka 14 niga muwa mbere. Umukobwa ajya mu mihango, amera incakwaha ni nsya, ndetse yumva ko yakuze muri we kuko hari aho aba yavuye naho aba yageze, atangira kumva ko yakuze nawe no mu byiyumviro bye bigahinduka.

Title: G S RWAMAGANA Girls TRANSCRIBED.docx

Doc Creator: tcuhawenimana11

Doc Date: 3/25/2023

Codes Applied: Pshcological changes occuring during puberty-Girls

Linked Memos: 0

Excerpt Creator: tcuhawenimana11

Excerpt Created On: 4/28/2023

Excerpt Range: 2847-2983

ndetse yumva ko yakuze muri we kuko hari aho aba yavuye naho aba yageze, atangira kumva ko yakuze nawe no mu byiyumviro bye bigahinduka.

Title: G S RWAMAGANA Girls TRANSCRIBED.docx

Doc Creator: tcuhawenimana11

Doc Date: 3/25/2023

Codes Applied: Pshcological changes occuring during puberty-Girls

Linked Memos: 0

Excerpt Creator: tcuhawenimana11

Excerpt Created On: 4/28/2023

Excerpt Range: 3046-3405

Ndi nimero 10 niga muwa kabiri mfite imyaka 18. Mu byiyumviro by’umukobwa atangira kumva nyine ari mukuru. Ari mukuru gute mu gihagararo mu bitekerezo gerageza. Mu bitekerezo icyo yumva adashoboye akabaza ababyeyi be, akajya nyine yumva yafasha ababyeyi be, akumva yakora ibintu byose nyine agafasha ababyeyi, mu byiyumviro bye akajya yumva ariwe mwana nyine.

Title: G S RWAMAGANA Girls TRANSCRIBED.docx

Doc Creator: tcuhawenimana11

Doc Date: 3/25/2023

Codes Applied: Physiological changes occuring during puberty-Girls

Linked Memos: 0

Excerpt Creator: tcuhawenimana11

Excerpt Created On: 4/28/2023

Excerpt Range: 3510-3695

Murakoze, ndi nimero 3 mfite imyaka 13 niga mu mwaka wamashuri yisumbuye. Impinduka harimo kumera amabere, kumera ubwoya ku mubiri, no kugira ibishishi ku mu biri, no kujya mu mihango.

Title: G S RWAMAGANA Girls TRANSCRIBED.docx

Doc Creator: tcuhawenimana11

Doc Date: 3/25/2023

Codes Applied: Boys and girls know about the causes leading to the physiological changes during puberty

Linked Memos: 0

Excerpt Creator: tcuhawenimana11

Excerpt Created On: 4/28/2023

Excerpt Range: 3823-3861

Ni ukubera imihindagurukire y’umuburi.

Title: G S RWAMAGANA Girls TRANSCRIBED.docx

Doc Creator: tcuhawenimana11

Doc Date: 3/25/2023

Codes Applied: Pshcological changes occuring during puberty-Girls

Linked Memos: 0

Excerpt Creator: tcuhawenimana11

Excerpt Created On: 4/28/2023

Excerpt Range: 3894-4221

Murakoze ndi nimero 2 mfite imyaka 15 nkaba niga muri senior two. Nyine ikintu navuga iyo umukobwa atangiye kugera mu gihe cy’imihindagurikire atangira kugenda yitwara ukundi kuntu, yabona abasore akiha iyindi ngendo nyine akumva atangiye kugenda ajya mu bintu byo gukunda abahungu cyane ndumva nta kindi nakongeraho murakoze.

Title: G S RWAMAGANA Girls TRANSCRIBED.docx

Doc Creator: tcuhawenimana11

Doc Date: 3/25/2023

Codes Applied: Pshcological changes occuring during puberty-Girls

Linked Memos: 0

Excerpt Creator: tcuhawenimana11

Excerpt Created On: 4/28/2023

Excerpt Range: 4270-4509

Murakoze ndi nimero 6 mfite imyaka 14 niga muwa mbere. Icyo nakongeraho ku mukobwa ugiye mu gihe cy’yumbwangavu atangira kugira isuku, kumva nyine yarabaye undi muntu nyine yarakuze akumva ibintu byose niwe ubikora cyangwa se ubizi, nibyo.

Title: G S RWAMAGANA Girls TRANSCRIBED.docx

Doc Creator: tcuhawenimana11

Doc Date: 3/25/2023

Codes Applied: Pshcological changes occuring during puberty-Girls

Linked Memos: 0

Excerpt Creator: tcuhawenimana11

Excerpt Created On: 4/28/2023

Excerpt Range: 4550-4729

Ni iyumuburiri we. Ni umubiri we naho atuye nyine. Isuku ni nkiyihe ra? Wenda nkiyo atarasanzwe akora ku mubiri we noneho yagera mu gihe mu bwangavu akabona itandukanye niyambere?

Title: G S RWAMAGANA Girls TRANSCRIBED.docx

Doc Creator: tcuhawenimana11

Doc Date: 3/25/2023

Codes Applied: Precautions to take during puberty for girls

Linked Memos: 0

Excerpt Creator: tcuhawenimana11

Excerpt Created On: 4/28/2023

Excerpt Range: 4747-5012

Murakoze nimero yange ni 5 mfite imyaka 16 niga senior 1. Umukobwa ugeze mu gihe cy’ubwangavu akunda kwigirira isuku ku mubiri we cyane cyane mu gihe ageze mu gihe cy’imihango, akigirira nyine isuku haba ku myambaro ndetse naho arara ndetse no kumubiri we murakoze.

Title: G S RWAMAGANA Girls TRANSCRIBED.docx

Doc Creator: tcuhawenimana11

Doc Date: 3/25/2023

Codes Applied: Precautions to take during puberty for girls

Linked Memos: 0

Excerpt Creator: tcuhawenimana11

Excerpt Created On: 4/28/2023

Excerpt Range: 5013-5210

Ku mubiri se ubwo ni nka gute? Ntiyarasanzwe akaraba wenda mbere yiyo myaka? Cyane cyane nyine nkiyo umukobwa ageze mu mihango nibwo aba anakwiriye no kugira isuku cyane cyane ayigirira umubiri we.

Title: G S RWAMAGANA Girls TRANSCRIBED.docx

Doc Creator: tcuhawenimana11

Doc Date: 3/25/2023

Codes Applied: Pshcological changes occuring during puberty-Girls

Linked Memos: 0

Excerpt Creator: tcuhawenimana11

Excerpt Created On: 4/28/2023

Excerpt Range: 5285-6134

Murakoze ndi nimero 7 mfite imyaka 19 niga mu mwaka wa gatanu wamashuri yisumbuye. Iyo umukobwa atangiye kugira imihindagurikire yumubiri we atangira kwigirira isuku. Uburyo yakwigirira isuku iyo ari umwana aba yumva nyine koga ntanikintu bimubwiye niyo yakoga rimwe ku munsi cyangwa nimugoroba akumva ubuzima arubwo ariko iyo yamaze kugera mu gihe cy’ubwangavu atangira kumva ko agomba kugira isuku akayigirira umubiri we akayigirira ndetse naho atuye, ikindi aba yumva ko agomba kugira izindi nshingano mu rugo nko gukora imirimo itandukanye yo mu rugo, igihe cyose yaba ari mu rugo akumva ashatse kwegera mama we akagira bimwe amusobanuza kugira nawe mu gihe azaba yageze mu gihe cyo kubaka urugo hatazagira bimwe bimunanira, ikindi nakumva yakora iyo agiye mu bandi atangira kumva akuze agomba no kwiyubaha kandi akubaha na bagenzi be. Murakoze.

Title: G S RWAMAGANA Girls TRANSCRIBED.docx

Doc Creator: tcuhawenimana11

Doc Date: 3/25/2023

Codes Applied: Pshcological changes occuring during puberty-Girls

Linked Memos: 0

Excerpt Creator: tcuhawenimana11

Excerpt Created On: 4/28/2023

Excerpt Range: 6220-6723

Iyo tukiri abana hari ibintu bimwe dukora bitandukanye nibyo umuntu mukuru akora wenda nkurugero nkumwana wiga muwa mbere ashobora nko gutaha ntamakayi afite ntabikoresho by’ishuri afite cyangwa yaba ari mu rugo akaba akora ibintu bigiye bitandukanye by’ubwana ariko iyo umaze gukura uba ugomba kumenya ko niba bakuguriye amakayi uri buyatahane, niba uhuye n’umuntu utagomba kumusuzugura ahubwo ugomba kumwubaha kandi nawe utagomba gusuzugurwa ahubwo uko wubashye ariko nawe bagomba kukubaha, murakoze.

Title: G S RWAMAGANA Girls TRANSCRIBED.docx

Doc Creator: tcuhawenimana11

Doc Date: 3/25/2023

Codes Applied: Precautions to take during puberty for girls

Linked Memos: 0

Excerpt Creator: tcuhawenimana11

Excerpt Created On: 4/28/2023

Excerpt Range: 6746-6903

Ndi nimero 3 mfite imyaka 13 niga muwa 1. Icyo nashakaga kuvuga ku bijyanye nisuku iyo umukobwa ageze mu gihe cy’ubwangavu agira nisuku ku gitsina, murakoze.

Title: G S RWAMAGANA Girls TRANSCRIBED.docx

Doc Creator: tcuhawenimana11

Doc Date: 3/25/2023

Codes Applied: Boys and girls know about the causes leading to the physiological changes during puberty

Linked Memos: 0

Excerpt Creator: tcuhawenimana11

Excerpt Created On: 4/28/2023

Excerpt Range: 7040-7366

Murakoze ndi nimero 4 mfite imyaka 17 niga mu mwaka wa 2. Impinduka wenda zishobora kugera ku mwana w’umukobwa zishobora wenda kuba ziterwa nikigero yaba agezemo hari igihe wenda abakobwa ntabwo bahinduka kimwe, hari ugira wenda imyaka 14 ataramera amabere ariko hari ugira impinduka akamera amabere wenda afite imyaka nka 12.

Title: G S RWAMAGANA Girls TRANSCRIBED.docx

Doc Creator: tcuhawenimana11

Doc Date: 3/25/2023

Codes Applied: How girls experience changes occuring to them during puberty

Linked Memos: 0

Excerpt Creator: tcuhawenimana11

Excerpt Created On: 4/28/2023

Excerpt Range: 7493-7772

Nyine wenda nk’umwana utarageza imyaka 15 ataramera amabere yumva yarihebye cyangwa se akumva atajya no mu bandi yahura numwana wenda mutoya yarameze amabere akumva wenda afite nkipfunwe akavuga ati uriya mwana ko ari mutoya kuri nge kubera iki wenda yameze amabere mbere yange?

Title: G S RWAMAGANA Girls TRANSCRIBED.docx

Doc Creator: tcuhawenimana11

Doc Date: 3/25/2023

Codes Applied: Received information is misleading and risky

Linked Memos: 0

Excerpt Creator: tcuhawenimana11

Excerpt Created On: 4/28/2023

Excerpt Range: 8074-8448

Ndi nimero 3 mfite imyaka 13 niga muri senior 1. Bashobora kukubeshya ngo iyo mugiye kwidumbaguza nkabantu ushyiraho agakoko kitwa inyagaruzi ngo kakaruma kwibere ngo agakura, noneho bashobora no kukubeshya ugeze mu gihe cy’ubwangavu ngo iyo abahungu bakoze ku mabere yawe ngo ashobora gukura cyangwa bakakubeshya ibindi cyangwa iyo wakora imibonanompuzabitsina ngo yakura.

Title: G S RWAMAGANA Girls TRANSCRIBED.docx

Doc Creator: tcuhawenimana11

Doc Date: 3/25/2023

Codes Applied: Boys and girls know about the causes leading to the physiological changes during puberty

Linked Memos: 0

Excerpt Creator: tcuhawenimana11

Excerpt Created On: 4/28/2023

Excerpt Range: 8615-8773

Murakoze ndi nimero 8 mfite imyaka 13 niga mu mwaka wa mbere wamashuri yisumbuye. Ndumva izo mpinduka zaba ziterwa nikigero umuntu aba agezemo ndumva aribyo.

Title: G S RWAMAGANA Girls TRANSCRIBED.docx

Doc Creator: tcuhawenimana11

Doc Date: 3/25/2023

Codes Applied: Boys and girls know about the causes leading to the physiological changes during puberty

Linked Memos: 0

Excerpt Creator: tcuhawenimana11

Excerpt Created On: 4/28/2023

Excerpt Range: 8820-9015

Murakoze ndi nimero 9 mfite imyaka 17 niga mu mwaka wa 5 w’amashuri yisumbuye. Izo mpinduka abakobwa bagira bageze mu gihe cy’ubwangavu hari igihe ziterwa wenda nabantu bari kumwe muri sosiyete.

Title: G S RWAMAGANA Girls TRANSCRIBED.docx

Doc Creator: tcuhawenimana11

Doc Date: 3/25/2023

Codes Applied: Pshcological changes occuring during puberty-Girls Obstacles and risks experienced by girls during the sexual and reproductive health changes occuring during puberty and adolescence

Linked Memos: 0

Excerpt Creator: tcuhawenimana11

Excerpt Created On: 4/28/2023

Excerpt Range: 9015-10006

Hari igihe umwana akura wenda abona nka mukuru we hari group agenderamo cyangwa hari abantu birirwana nawe wenda akazajya muyindi group ikamushuka ati wenda kugirango uzana amabere nkuko mugenzi wange yabivuze nukujya mu busambanyi niyindi mico mibi iyariyo yose cyangwa bikanaterwa nuko aba yatangiye guhindura icyiciro arimo ati hari abantu wenda banyitayeho akumva hari ukundi kuntu yahindutse mu mitekerereze ye agatangira kwigirira isuku wenda ngo abahungu baramureba cyangwa abandi bantu baraho muri society imwe, agatangira kumenya kwigirira isuku ku mubiri, ku myambaro, niba wenda yakarabaga 3 mu cy’umweru akjya akaraba buri munsi akamabara imyenda ifuze, akambara imyenda y’imbere nayo ifuze buri munsi kuko iyo umuntu akiri umwana hari igihe wenda yambaraga nka suve wenda iminsi 3 idafurwa wenda ababyeyi batamwitayeho cyangwa ntanumukozi agira ariko iyo atangiye kugera mu gihe cy’ubwangavu atangira kuva ku ntera imwe ajya ku yindi akamenya kwigirira isuku ku mubiri murakoze.

Title: G S RWAMAGANA Girls TRANSCRIBED.docx

Doc Creator: tcuhawenimana11

Doc Date: 3/25/2023

Codes Applied: Pshcological changes occuring during puberty-Girls

Linked Memos: 0

Excerpt Creator: tcuhawenimana11

Excerpt Created On: 4/28/2023

Excerpt Range: 10055-10348

Murakoze ndi nimero 8 mfite imyaka 13 niga mu mwaka wa 1 wamashuri yisumbuye. Nanone izo mpinduka zishobora kuba ziterwa n’urungano rwe cyangwa na group agenderamo kuko bashobora kuba bakora ibindi bintu nawe akavuga ati nange reka mbigane bigatuma agira izindi mpinduka zishobora kumuhindura.

Title: G S RWAMAGANA Girls TRANSCRIBED.docx

Doc Creator: tcuhawenimana11

Doc Date: 3/25/2023

Codes Applied: Obstacles and risks experienced by girls during the sexual and reproductive health changes occuring during puberty and adolescence Pshcological changes occuring during puberty-Girls

Linked Memos: 0

Excerpt Creator: tcuhawenimana11

Excerpt Created On: 4/28/2023

Excerpt Range: 10520-11839

Murakoze ndi nimero 7 mfite imyaka 19 niga mu mwaka 5 wamashuri yisumbuye. Amagambo bakunda kubwira abana babakobwa bigatuma nabo bahinduka. Hari igihe ushobora guhura numwana wumukobwa wenda tuvuge nkurugero avuye kw’ishuri, niba avuye kw’ishuri mu byukuri asanzwe agira imico myiza ariko bitewe na group agenderamo nge nshobora kuba ngendana na bagenzi bange , bagenzi bange bafite ikigare, hanyuma bakambwira bati uhora wambaye ibintu bigera ku birenge, uhora wambaye ibipapa ntabwo ujya wambara udukweto twiza ngo usirimuke kandi iwacu ntabushobozi dufite bwo kujya kubigura, ubwo bushobozi ibyo ngibyo nambaye aribwo bushobozi iwacu dufite ariko bitewe na cya kigare ngenderamo agahita ambwira ati uzaze nkwereke umuntu uzajya agufata neza. Niba ambwira ngo ngende anyereke umuntu umfata neza, iyo nshuti yange ndayizera mba nzi ko nta kintu kibi yanjyanamo, mu byukuri ashobora kugenda akagushyikiriza nkumu sugar daddy yamara kumugushyikiriza akazajya aguha bya bindi byose ukeneye niba warusigaye wambara bodaboda cyangwa silipa agatangira kwambara udukweto twiza dufunze kandi duhenze, niba wambaraga ijipo igera ku birenge ugatangira kwambara utujipo tugufiya ukambara udukanzu twiza tugezweho, ibyo byose mubyukuri wabitewe nuko yaje akakubwira ngo uri umuturage wambara ibintu nyine bidasirimutse murakoze.

Title: G S RWAMAGANA Girls TRANSCRIBED.docx

Doc Creator: tcuhawenimana11

Doc Date: 3/25/2023

Codes Applied: Obstacles and risks experienced by girls during the sexual and reproductive health changes occuring during puberty and adolescence

Linked Memos: 0

Excerpt Creator: tcuhawenimana11

Excerpt Created On: 4/28/2023

Excerpt Range: 11911-12336

Murakoze nimero yange ni 2 mfite imyaka 15 niga senior 2. Ikintu nashakaga kongeraho cyatuma umukobwa ahinduka bitewe namagambo bamubwiye wenda ushobora kuba ukundana numusore akajya ahora akubwira ngo ni wowe mwiza, akajya ahora akubwira ngo ufite amabuno nyine akajya ahora agutakagiza nawe ukagenda ukiyemera nyine ugatangira guhindura ingendo yawe, imyifatire bitewe nukuntu yagiye agutakagiza birenze urugero, murakoze.

Title: G S RWAMAGANA Girls TRANSCRIBED.docx

Doc Creator: tcuhawenimana11

Doc Date: 3/25/2023

Codes Applied: How girls experience changes occuring to them during puberty

Linked Memos: 0

Excerpt Creator: tcuhawenimana11

Excerpt Created On: 4/28/2023

Excerpt Range: 12531-13054

Murakoze nimero yange 5 mfite imyaka 16 niga senior 1. Murizo mpinduka bavuze impinduka ya mbere yambayeho nabanje kumera amabere iyindi mpinduka ya kabiri nagiye mu mihango iyindi mpinduka nyine ya 3 nameze ubwoya nyine ku myanya yibanga ngira nincakwaha. Maze nyine kujya mu mihango nagishije inama ababyeyi mbabwira ko nyine nayigiyemo nabo bambwira uko ngomba kubyitwaramo, amabere yo nyine narayameze kuko nabonaga abandi bana bayafite nkavuga nti ngewe nzayamera ryari rero nyine byaranshimishije kuko narayashakaga.

Title: G S RWAMAGANA Girls TRANSCRIBED.docx

Doc Creator: tcuhawenimana11

Doc Date: 3/25/2023

Codes Applied: How girls experience changes occuring to them during puberty

Linked Memos: 0

Excerpt Creator: tcuhawenimana11

Excerpt Created On: 4/28/2023

Excerpt Range: 13185-13656

Murakoze ndi nimero 6 niga mu mwaka wa 1. Izo mpinduka zimwe mu zambayeho nameze amabere njya no mu mihango. Icyaje mbere niki? Amabere. Amaze kuza wabyitwayemo ute? Yaje nyashaka nyine. Imihango yo? Udusangize uburyo wabyitwayemo; nabibwiye mama aramabwira ngo nyine nakuze ambwira ngo nukwigirira isuku, ambwira nyine uburyo ngomba kwitwaramo. Yakubwiye ko ugomba kubyitwaramo gute se? nyine yarambwiye ngo nzajye nkunda kugira isuku cyane ngishe inama mubyo ntumvishe.

Title: G S RWAMAGANA Girls TRANSCRIBED.docx

Doc Creator: tcuhawenimana11

Doc Date: 3/25/2023

Codes Applied: How girls experience changes occuring to them during puberty

Linked Memos: 0

Excerpt Creator: tcuhawenimana11

Excerpt Created On: 4/28/2023

Excerpt Range: 13732-14591

Ndi nimero 10 niga mu mwaka 2 mfite imyaka 18. Murizo mpinduka nge nagiye mu mihango mera namabere. Imihango wayigiyemo ufute imyaka ingahe? 12. Nge bwa mbere njya mu mihango numvishije bimbangamiye cyane numvaga ngewe ntabishaka numvaga ibyo bintu bitanasanzwe. Ntamakuru se warufite ku mihango ku myaka 12? Narabyumvaga nyine babivuga arko nge nkumva ntabyo nshaka. Impamvu wumvaga utabishaka se ni iyihe? Ntabwo nari nakamenye nyine icyo bimaze. Imaze kuza se wabyitwayemo ute? Nabibwiye mama arambwira ngo ntabishaka ariko bifite akamaro arambwira ngo nge nitwara neza nyine ntazajya mpura nabahungu bakankoraho bakambangamira ngo sinkabe inshuti namabungu cyane ngo hatazagira ibyo bankorere gishingiye ku mibonanompuzabitsina kuko nshobora guhita nsama inda. Amabere yo wayameze ku myaka ingahe? 11. None se byo wabyakiriye ute? Numvaga ntacyo bitwaye.

Title: G S RWAMAGANA Girls TRANSCRIBED.docx

Doc Creator: tcuhawenimana11

Doc Date: 3/25/2023

Codes Applied: Sources of information received during puberty

Linked Memos: 0

Excerpt Creator: tcuhawenimana11

Excerpt Created On: 4/28/2023

Excerpt Range: 14213-14228

Nabibwiye mama

Title: G S RWAMAGANA Girls TRANSCRIBED.docx

Doc Creator: tcuhawenimana11

Doc Date: 3/25/2023

Codes Applied: How girls experience changes occuring to them during puberty

Linked Memos: 0

Excerpt Creator: tcuhawenimana11

Excerpt Created On: 4/28/2023

Excerpt Range: 14661-15385

Ndi nimero ya 1 mfite imyaka 12 niga muwa 1. Impinduka zambayeho nukumera amabere ariko akenshi ntabwo nayishimiye nari mfite imyaka 11 kuko hari imyenda ujya kwambara ukabona urakubangamiye bitewe nukuntu ikwegereye kandi mbere itaragufataga ntabwo rero ubyishimira ariko nyine uba wumva ko wakuze mu bandi kuko hari ahantu nshobora kujya bakavuga ngo wowe uri umwana ntanamabere ufite nkavuga ati nange narakuze. Yakongereye icyizere. Yego kuko uba wumva warakuze kuko haraho uba waravuye nange haruwo nshobora kubwira ngo uri umwana kuko ibingibi ntabyo ufite kandi ngewe ndabifite. Imihango nayo ntago umuntu ayishimira cyane kuko iyo utayimenyereye ishobora kugusebya ahantu utarubyiteguye ntabwo rero uba ubyishimiye.

Title: G S RWAMAGANA Girls TRANSCRIBED.docx

Doc Creator: tcuhawenimana11

Doc Date: 3/25/2023

Codes Applied: How girls experience changes occuring to them during puberty

Linked Memos: 0

Excerpt Creator: tcuhawenimana11

Excerpt Created On: 4/28/2023

Excerpt Range: 15494-16073

ngewe ntago yigeze intungura cyane ngo insebye kuko narindi mu rugo ndi kumwe na mama gusa usibye ko byambangamiye kubera ukuntu uribwa munda kandi ntabwo uba ubizi wumva ko ari ibisanzwe gusa mama yarabimbwira nkamubwira ati basi ibyo bintu bizaze mfite nka 20 akambwira ati nejobundi ariko nkumva ngewe ntabyo nshaka ariko ntakundi byagenda kuko biba bigomba kubaho. Umenya uburyo witwara ukambara imyenda yisuku ugakoresha nibikoresho biba biri ngombwa kugirango ukomeze mwisuku yawe kuko iyo utagizi isuku ushobora guhura na zimwe mu ndwara zitari nziza kandi uri umwangavu.

Title: G S RWAMAGANA Girls TRANSCRIBED.docx

Doc Creator: tcuhawenimana11

Doc Date: 3/25/2023

Codes Applied: How to take care of themselves when phsyiological changes occur for girls

Linked Memos: 0

Excerpt Creator: tcuhawenimana11

Excerpt Created On: 4/28/2023

Excerpt Range: 15864-16073

Umenya uburyo witwara ukambara imyenda yisuku ugakoresha nibikoresho biba biri ngombwa kugirango ukomeze mwisuku yawe kuko iyo utagizi isuku ushobora guhura na zimwe mu ndwara zitari nziza kandi uri umwangavu.

Title: G S RWAMAGANA Girls TRANSCRIBED.docx

Doc Creator: tcuhawenimana11

Doc Date: 3/25/2023

Codes Applied: Precautions to take during puberty for girls

Linked Memos: 0

Excerpt Creator: tcuhawenimana11

Excerpt Created On: 4/28/2023

Excerpt Range: 16138-16199

Ushobora kutigirira isuku ukandwara indwara ziterwa numwanda.

Title: G S RWAMAGANA Girls TRANSCRIBED.docx

Doc Creator: tcuhawenimana11

Doc Date: 3/25/2023

Codes Applied: Precautions to take during puberty for girls

Linked Memos: 0

Excerpt Creator: tcuhawenimana11

Excerpt Created On: 4/28/2023

Excerpt Range: 16254-16405

Umuntu uri mu mihango akenshi uba ugomba kwirinda abahungu ukirinda ibishuko, bisaba rero kugira imbaraga nyinshi ndetse no gukomera ku gisubizo cyawe.

Title: G S RWAMAGANA Girls TRANSCRIBED.docx

Doc Creator: tcuhawenimana11

Doc Date: 3/25/2023

Codes Applied: Precautions to take during puberty for girls

Linked Memos: 0

Excerpt Creator: tcuhawenimana11

Excerpt Created On: 4/28/2023

Excerpt Range: 16550-16780

Umuhungu ashobora kugushuka uri mu mihango ugatwara inda ariko hari nigihe akenshi bamwe batwara inda aruko batari babyiteguye ashobora kuba azi ko yayivuyemo ariko kuruwo munsi ikamutungura ikaza biba bisaba rero kubyitwararika.

Title: G S RWAMAGANA Girls TRANSCRIBED.docx

Doc Creator: tcuhawenimana11

Doc Date: 3/25/2023

Codes Applied: How girls experience changes occuring to them during puberty

Linked Memos: 0

Excerpt Creator: tcuhawenimana11

Excerpt Created On: 4/28/2023

Excerpt Range: 17028-17522

Murakoze ndi nimero 2 mfite imyaka 15 niga muri senior 2. Nyine mbere narabanje mfite imyaka 12 mera amabere noneho ntangiye kumera amabere natangiye kugenda nunamye nkamera nkumuntu uyahishe nkahora nambaye umupira wimbeho ntabwo amabere nayishimiye akiz, ariko ubu narabyakiriye. Noneho ngize imyaka 13 njya mu mihango nayigiyemo ndi kwishuri ndi mu kigo mbamo nyine abanyeshuri benshi barimo abantu bakuru barakuganiriza ukumva ari ibintu bisanzwe, ntago nge imihango yambangamiye, murakoze.

Title: G S RWAMAGANA Girls TRANSCRIBED.docx

Doc Creator: tcuhawenimana11

Doc Date: 3/25/2023

Codes Applied: How girls experience changes occuring to them during puberty

Linked Memos: 0

Excerpt Creator: tcuhawenimana11

Excerpt Created On: 4/28/2023

Excerpt Range: 17679-17923

Murakoze ndi nimero 3 mfite imyaka 13 niga muri senior 1. Kumera amabere byonyine byatangiye ari ibere rimwe ari kamwe gakura akandi ari gato noneho nkazajya nambara imipira 2 nishati kugirango rya rindi ritagaragara noneho rirakura yose araza.

Title: G S RWAMAGANA Girls TRANSCRIBED.docx

Doc Creator: tcuhawenimana11

Doc Date: 3/25/2023

Codes Applied: How girls experience changes occuring to them during puberty

Linked Memos: 0

Excerpt Creator: tcuhawenimana11

Excerpt Created On: 4/28/2023

Excerpt Range: 18164-21452

Murakoze ndi nimero 7 mfite imyaka 19 niga mu mwaka wa 5 wa mashuri yisumbuye. Ngewe impinduka zambayeho mu gihe cyubwangavu ikintu cyambere cyambayeho cyari ikigare, ikigare nakigize niga mu mwaka wa 3 wamashuri yisumbuye nibwo narimfite abana 6 nako ntabwo nakigiyemo niga muwa 3 naringeze muwa 1 secondary. Twaje muwa 1 turi abana 6 kandi baba mugakaristiye kamwe rero twazanaga kwishuri tukanatahana ibintu byose nakoraga twabaga turi kumwe. Nkahano kwishuri niga muwa 1 igipangu bari bataragishyiraho hari hariho nyine imiyenzi saa sita zagera bakambwira ngo dutoroke tujye kurya isambusa kugenda tukajya kurya isambusa tukagaruka mu kigo. Ndibuka neza ko igiihembwe cya mbere ko nagize amanota 53 icyakabiri nkagira 32, nzanye ayo manota papa yarankubise pe cyane ndetse arangije kuko narimfite na musaza wange wiga ahangaha aramubwira ati agira abandi bana birirwa bagendana ako ngewe barabimbwiraga nkumva bari kumbangamira nkumva sinanabireka nkumva aribwo buzima. Igihembwe cya 3 haza kuza undi mwana waruvuye ku kindi kigo atangira kugenda ambwira ati dore abangaba baragushuka ako ntabwo nabyemeye numvaga ambangamiye nka saa yine tukajya nkahantu hatari abantu benshi tukiga twamara kwiga nkongera nkasubira muri cya kigare ariko nanagiye no kwiga ku ruhande birangiye nongeye kugira amanota meza papa angarurira icyizere. Ngeze muwa kabiri nabwo kuko twari 6 bamwe bajya mwishuri rimwe nyine baradutandukanya bamaze kudutandukanya ariko tukongera tugahura saa sita nubundi bigakomeza bigenda gutyo byaje kurangira 2 batwaye inda muwa 2 tuba dusigaye turi 4. Tugeze muwa 3 noneho mpura nabandi bana baba baravuye kubindi bigo bitadukanye banywa itabi ndetse ninzoga ako nabwo ntabwo nigeze nkireka kuko nicaraga ku ntebe yambere ariko nkimuka nkabasanga nyine biza kurangira umwarimu aduhaye imyanya bamwe abashyira imbere abandi abashyira inyuma banyicazamo hagati bamaze kunyicazamo hagati cya kigare kigakomeza ako biza kurangira cya kigare gifashe ishuri ryose, kimaze gufata ishuri ryose hari amasomo nyine tutigaga nka chimie, imibare ndetse na phyisique ntabwo twayigaga, umwarimu yarazaga umwana umwe akandika urupapuro ngo twiryamire rukazenguruka ishuri ryose noneho umwarimu yahindukira agasanga ishuri ryose abana baryamye yabaza ngo ko mwaryamye tukamubwira ngo turashonje yabaza ati se ntabwo mwiga tukamubwira ngo ntabwo twiga dushonje nyine bikamucanga akabasigira note yarangiza akigendera. Wenda muribyo bintu byose twakoraga ikintu cyaje kubamo abana babisiramu bakoreyemo fete nyine basiga batahakoreye isuku neza hasigaramo amasahane ariho ibryo nigikoma mugitondo kuwa 1 tuje kwiga baratubwira ati nimubikureho nyine imitwe sinzi niba bari bamaze kuyi format ati nimugende mubwire animateur aze abikureho ibyo bintu byatubayeho byageze naho directeur atwandikisha ibaruwa aratubwira ati nimugende muzagaruka muje gukora icya leta, umva twese nyine twumvaga ntanikintu bitubwiye tukumva nyine ko tuzagenda tukajya mu rugo ariko ntitumenye ko bashobora kutwirukana gutyo bamwe bakabashakira umwarimu uzajya abigisha iwabo cyangwa bakuru babo abandi bakaguma mu rugo ugasanga nyine mu kizamini cya leta tugize amanota mabi nyine kuko twagiraga animateur waduhaga ibihano ariko nyine yagiraga nimpuhwe agenda adusabira imbabazi kwa directeur aratubabarira.

Title: G S RWAMAGANA Girls TRANSCRIBED.docx

Doc Creator: tcuhawenimana11

Doc Date: 3/25/2023

Codes Applied: How girls experience changes occuring to them during puberty

Linked Memos: 0

Excerpt Creator: tcuhawenimana11

Excerpt Created On: 4/28/2023

Excerpt Range: 21639-21962

Oya ngewe nubwo bwose abo bose banywaga inzoga ndetse nitabi ngewe ntabwo nigeze nzinywa, ngewe nabikoraga ubundi nicecekeye ntabwo nakundaga kuvuga bambwira ngo tujye aha tukagenda tugatoroka bakagenda bakagura izo zonga bakaza tukicara tukazinywa ariko ngewe inzoga nitabi ntabwo nigeze mbinywa. Wagize Imana irakurokoye.

Title: G S RWAMAGANA Girls TRANSCRIBED.docx

Doc Creator: tcuhawenimana11

Doc Date: 3/25/2023

Codes Applied: Information that girls need

Linked Memos: 0

Excerpt Creator: tcuhawenimana11

Excerpt Created On: 4/28/2023

Excerpt Range: 22241-22637

Murakoze ndi nimero 8 mfite imyaka 13 niga mu mwaka mbere wamashuri yisumbuye. Ndumva amakuru yakenera namakuru nyine ajyanye nubuzima bwe akaba yayasobanuza akayamenya nimihindagurikire yibintu nyine birimo biragenda bimubaho atumva neza cyangwa ntabisobanukirwe ashobora nabyo kubikeneraho amakuru cyangwa se nibindi bintu bigenda bimubaho muricyo gihe nibyo ashobora gukeneraho amakuru cyane.

Title: G S RWAMAGANA Girls TRANSCRIBED.docx

Doc Creator: tcuhawenimana11

Doc Date: 3/25/2023

Codes Applied: Information that girls need

Linked Memos: 0

Excerpt Creator: tcuhawenimana11

Excerpt Created On: 4/28/2023

Excerpt Range: 22730-23495

Murakoze ndi nimero 9 mfite imyaka 17 niga mu mwaka wa 5 wamashuri yisumbuye. Umwana wumwangavu agomba kumenya amakuru yubuzima bwimyororokere akamenya mu gihe umuntu agiye mu mihango uko yakwitwara ibikoresho akoresha uko abikoresha uko yabigira nyuma yo kubikoresha akagira zimwe mu ngeso mbi areka bakamuha nubusobanuro ku buzima bwimyororokere akareka ikigare akareka kugendera muma group yamujyana mu ngeso mbi nkubusambanyi nizindi ngeso mbi izarizo zose, agomba kumenya amakuru kubijyanye na sosiyete turimo dore irahindagurika abantu bose baba bakugira inama siko bose baba bagukunda umubyeyi akagusobanurira akakubwira ko abantu bose bari hanze atari abantu beza, akakubwira ugomba kumenya inzira ucamo kuko igihe cyose ntabwo mwahora muri kumwe, murakoze

Title: G S RWAMAGANA Girls TRANSCRIBED.docx

Doc Creator: tcuhawenimana11

Doc Date: 3/25/2023

Codes Applied: Information that girls need

Linked Memos: 0

Excerpt Creator: tcuhawenimana11

Excerpt Created On: 4/28/2023

Excerpt Range: 23527-24496

Murakoze ndi nimero 7 mfite imyaka 19 niga muwa5. Ngewe numva amakuru umwana wumwangavu yagira ikintu cya mbere bagomba kumugira inama kubera ko iyo tutagirwa inama ntago ubuzima tuba dufite nyine uko tumeze ntago ariko tuba tumeze. Twebwe iyo tumaze kugera mu gihe cyubwangavu tuba dufite inshuti nyinshi zitujyana mu bigare bitandukanye. Inshuti yawe iraza ikakubwira iti tujye ahangaha ukagenda indi ikaza ikakubwira iti tunyure ahangaha ukagenda, muri izo nshuti 2 zose ntabwo ziba zitwifuriza ibyiza, zishobora kutujyana mu bintu bigiye bitandukanye ariko iyo dufite umuntu utugira inama aratubwira ati umuntu wese ukubwira ati ca aha ntabwo aruko aba agomba kugucisha mu nzira nziza, ikindi wenda nakongeraho bagomba nyine kumenya amakuru yimihindagirikire yimibiri yabo kubera ko abana benshi muri iki gihe barimo gutwara inda ariko ntabwo batwra inda aruko babikunze cyangwa aruko babishaka ahubwo nuko ntamakuru aba ahagije aba bafite ku mibiri yabo, murakoze.

Title: G S RWAMAGANA Girls TRANSCRIBED.docx

Doc Creator: tcuhawenimana11

Doc Date: 3/25/2023

Codes Applied: Sources of information received during puberty

Linked Memos: 0

Excerpt Creator: tcuhawenimana11

Excerpt Created On: 4/28/2023

Excerpt Range: 24546-25035

Murakoze, ahantu wakura ayo makuru bwa mbere ni kuba babyeyi bawe, ukagenda ukegera umubyeyi wawe umubyeyi ukamugisha inama ukamubaza icyo utumva akakubwira kuko iyo uteze amatwi bagenzi bawe nko mwishuri haba harimo amakuru menshi agiye atandukanye kandi amakuru aba ari mwishuri ntabwo yose aba ahagije ahubwo ushobora kugenda ukegera umubyeyi wawe, ukegera mukuru wawe, umuntu wisanzuraho wumuturanyise cyangwa ibyo byose wabona ntamuntu uri kwisanzuraho ukajya mu cyumba cyurubyiruko.

Title: G S RWAMAGANA Girls TRANSCRIBED.docx

Doc Creator: tcuhawenimana11

Doc Date: 3/25/2023

Codes Applied: Information that boys need

Linked Memos: 0

Excerpt Creator: tcuhawenimana11

Excerpt Created On: 4/28/2023

Excerpt Range: 25346-25793

Murakoze ndi nimero 2 mfite imyaka 15 niga senior 2. Wenda amakuru umuhungu nyine ageze mu gihe cyubugimbi yakenera hari igihe nyine umuhungu atangira kuzana ibishishi agatangira kuniga ijwi akajya yumva nyine adatuje akumva nyine kujya kuvugira mu bandi basore bimuteye ipfunwe biba bisaba ngo umubyeyi amuganirize amubwire ko ageze mu gihe cyimihindagurikire akajya mu bandi ntapfinwe afite akajya avuga kandi bimuvuye ku mutima ndumva ntakindi.

Title: G S RWAMAGANA Girls TRANSCRIBED.docx

Doc Creator: tcuhawenimana11

Doc Date: 3/25/2023

Codes Applied: Information that boys need

Linked Memos: 0

Excerpt Creator: tcuhawenimana11

Excerpt Created On: 4/28/2023

Excerpt Range: 26005-26653

Murakoze nimero yange ni 5 mfite imyaka 16 niga senior 1. Amakuru numva umwana wumuhungu akwiriye kugira numva ko yazajya yegera nkumubyeyi we papa we akamugira inama ndetse hari nigihe uba utisanzura ku babyeyi wenda ukareba nkinshuti yawe ukayigisha inama cg ukareba nkundi mubyeyi muturanye nawe ukamugisha inama. Urabona iyo umuhungu akundanye numukobwa akenshi abahungu benshi baba bashakaga ngo bakundane numukobwa bakore imibonanompuzabitsina hari nkigihe rero umuhungu agira nkicyo kibazo akavuga atise ibingibi ndamutse mbikoze byaba ari byiza? Akagira gushidikanya noneho bikaba ngombwa ko yabaza umubyeyi we cyangwa inshuti ye, murakoze.

Title: G S RWAMAGANA Girls TRANSCRIBED.docx

Doc Creator: tcuhawenimana11

Doc Date: 3/25/2023

Codes Applied: Sources of information received during puberty

Linked Memos: 0

Excerpt Creator: tcuhawenimana11

Excerpt Created On: 4/28/2023

Excerpt Range: 26847-27587

Murakoze nimero yange ni 4 mfite imyaka 17 niga mu mwaka wa 2. Amakuru naba narahawe wenda naba narayakuye ku babyeyi bange, inshuti zange cyangwa wenda ku bigo byurubyiruko. Amakuru yose naba narahawe wenda umubyeyi arakwicaza akakubwira ati mwana wange ndabona umaze gukura mu gihagararo se wameze amabere wagiye no mu mihango ugomba kugira uko witwara ukirinda abagushuka wenda hari igihe uhura numugabo akakubwira ngo mukundane wenda nawe wabyemera mugakundana ariko nyine igihe kiragera agashaka kugukoresha imibonanompuzabitsina ariko iyo umubyeyi wawe yakubwiye ati uramenye ntuzakore imibonanompuzabitsina ushobora kuyikora ugatwara inda cyangwa se wanayikora mukikingira wenda kwifata byabananiye umubyeyi wawe akabikugiramo inama.

Title: G S RWAMAGANA Girls TRANSCRIBED.docx

Doc Creator: tcuhawenimana11

Doc Date: 3/25/2023

Codes Applied: Types of information provided to boys and girls during puberty

Linked Memos: 0

Excerpt Creator: tcuhawenimana11

Excerpt Created On: 4/28/2023

Excerpt Range: 27022-27587

Amakuru yose naba narahawe wenda umubyeyi arakwicaza akakubwira ati mwana wange ndabona umaze gukura mu gihagararo se wameze amabere wagiye no mu mihango ugomba kugira uko witwara ukirinda abagushuka wenda hari igihe uhura numugabo akakubwira ngo mukundane wenda nawe wabyemera mugakundana ariko nyine igihe kiragera agashaka kugukoresha imibonanompuzabitsina ariko iyo umubyeyi wawe yakubwiye ati uramenye ntuzakore imibonanompuzabitsina ushobora kuyikora ugatwara inda cyangwa se wanayikora mukikingira wenda kwifata byabananiye umubyeyi wawe akabikugiramo inama.

Title: G S RWAMAGANA Girls TRANSCRIBED.docx

Doc Creator: tcuhawenimana11

Doc Date: 3/25/2023

Codes Applied: Types of information provided to boys and girls during puberty

Linked Memos: 0

Excerpt Creator: tcuhawenimana11

Excerpt Created On: 4/28/2023

Excerpt Range: 27734-28487

Murakoze ndi nimero 9 mfite imyaka 17 niga mu mwaka wa 5. Amakuru naba narahawe ajyanye nimyororokere ku mukobwa ni nkukuntu umuntu ugeze mu gihe cy’imihango ukuntu yakitwara, ukuntu yakwambara cotex mu gihe byamubayeho ari kwishuri cyangwa ari mu rugo, ukuntu umuntu yakwirinda ubusambanyi mu gihe ageze mu gihe cy’ubwangavu kuko hari igihe baba bagushuka ngo mukore ubwo busambanyi, ukuntu umuntu yakitwara muri bagenzi be akareka ibintu byo kugendera mu kigare cyangwa kwishinga abandi akararikira ibyo abandi bafite kuba bafite urukweto rwiza imyenda myiza bakagendera mu kigare. Andi makuru nukuntu wajya ugisha inama abakuruta ku bintu utarusanzwe wiyiziho ukagisha inama ababyeyi cyangwa bakuru bawe cyangwa abandi mugira icyo mupfana, murakoze.

Title: G S RWAMAGANA Girls TRANSCRIBED.docx

Doc Creator: tcuhawenimana11

Doc Date: 3/25/2023

Codes Applied: Types of information provided to boys and girls during puberty

Linked Memos: 0

Excerpt Creator: tcuhawenimana11

Excerpt Created On: 4/28/2023

Excerpt Range: 28541-28794

Murakoze ndi nimero 8 mfite imyaka 13 niga mu mwaka wa mbere wamashuri yisumbuye. Amakuru naba narahawe ni nkukuntu wakitwara mu kigero ugenda ugeramo ibyo wakwirinda ningaruka zirimo nyine ugahabwa ayo makuru niyo makuru numva naba narahawe, murakoze.

Title: G S RWAMAGANA Girls TRANSCRIBED.docx

Doc Creator: tcuhawenimana11

Doc Date: 3/25/2023

Codes Applied: Sources of information received during puberty

Linked Memos: 0

Excerpt Creator: tcuhawenimana11

Excerpt Created On: 4/28/2023

Excerpt Range: 28921-29189

Nimero 1 mfite imyaka 14 niga muwa mbere mu mashuri yisumbuye. Ahantu dukura izo nama ni ku babyeyi ndetse no mu byumba bimwe na bimwe bigenda bibasobanurira ku buzima bwimyororokere nimihindagurikire yumubiri no muri bagenzi bacu bamwe na bamwe tugenda tuganira nabo.

Title: G S RWAMAGANA Girls TRANSCRIBED.docx

Doc Creator: tcuhawenimana11

Doc Date: 3/25/2023

Codes Applied: Sources of information received during puberty

Linked Memos: 0

Excerpt Creator: tcuhawenimana11

Excerpt Created On: 4/28/2023

Excerpt Range: 29402-30106

Ndi nimero 10 mfite imyaka 18 niga mu mwaka wa 2. Ahantu dukura izo nama ahambere ni ku babyeyi, si inama namakuru, no mu bantu mugendana nyine ushobora nko guca ahantu bakabivuga ukabyumva cyangwa se abantu mugendana cyangwa se abakuru bakaba babikugiramo inama. Ayo makuru wenda dufate wakuye kubabyeyi cyangwa se wagiye wumva amenshi uyakura ku babyeyi cyangwa mu nshuti? Dusangize; amakuru menshi ngewe nayakuye mwishuri ku mwarimu wacu utwigisha Biology niwe wakundaga kutugira inama cyane numubyeyi wange. Wamara kuyiga ukaba wagenda ugasobanuza umubyeyi? Ese ababyeyi mubisangaho kuburyo wamubaza ikintu cyose kijyanye nimihindagurikire? Ngewe umubyeyi wange mwisanzuraho ibintu byose ndabimubaza.

Title: G S RWAMAGANA Girls TRANSCRIBED.docx

Doc Creator: tcuhawenimana11

Doc Date: 3/25/2023

Codes Applied: Sources of information received during puberty Types of information provided to boys and girls during puberty

Linked Memos: 0

Excerpt Creator: tcuhawenimana11

Excerpt Created On: 4/28/2023

Excerpt Range: 30225-30618

Murakoze, ndi nimero 2 mfite imyaka 15 niga senior 2. Nyine amakuru ahantu nyakura mbere na mbere ubanza kubaza umubyeyi, hari ibintu bisigaye bikunda kuba muri ino rwamagana bagahamagaza abakobwa bakagenda bakatugira inama bakakwigisha ukuntu wakwirinda wenda ngo niba ugiye gusura nkumuhungu ngo wenda witwaze prudence bakakwereka ukuntu bayambara mbese baba barimo barigisha ibintu byinshi.

Title: G S RWAMAGANA Girls TRANSCRIBED.docx

Doc Creator: tcuhawenimana11

Doc Date: 3/25/2023

Codes Applied: Sources of information received during puberty

Linked Memos: 0

Excerpt Creator: tcuhawenimana11

Excerpt Created On: 4/28/2023

Excerpt Range: 30685-31192

Niba baratubwiye ngo baba baturutse mu kigo cyabasirikare nyine bagiye mu bintu byubwangavu batumiye abakobwa gusa, murakoze. Amakuru baba baraguhaye se uretse kukubwira ngo nujya gusura umuhungu ku mihindagurikire yo ntamakuru babahaye? Baraduhaye.

Murakoze ndi nimero 3 mfite imyaka 13 niga muri senior 1. Ahantu twaba tuyakura hari umushinga witwa dreams tujya kwigamo buri wa 6 witwa dreams baduha ayo makuru buri wa 6 tujyayo nabakobwa turagenda bakatubwira uko twakwitwara nizindi nama murakoze cyane.

Title: G S RWAMAGANA Girls TRANSCRIBED.docx

Doc Creator: tcuhawenimana11

Doc Date: 3/25/2023

Codes Applied: Obstacles and risks experienced by boys during the sexual and reproductive health changes occuring during puberty and adolescence

Linked Memos: 0

Excerpt Creator: tcuhawenimana11

Excerpt Created On: 4/28/2023

Excerpt Range: 31335-31810

Murakoze ndi nimero 4 mfite imyaka 17 niga nu mwaka wa 2. Ingorane abana babahungu bashobora guhura nazo mu gihe wenda bageze mu gihe cyubugimbi hari igihe wenda umwana aba yarakuze nko mu muryango wenda ufitanye amakimbirane atabasha, kwisanzura wenda ku mubyeyi we ngo amusobanuze wenda bimwe mu bimenyetso aba agenda yibonaho atagira wenda ninshuti cyangwa se wenda undi muntu yakwisanzuraho ngo amubaze nyine iyo ikaba ingorane ashobora guhura nayo yo kutamenya amakuru.

Title: G S RWAMAGANA Girls TRANSCRIBED.docx

Doc Creator: tcuhawenimana11

Doc Date: 3/25/2023

Codes Applied: Obstacles and risks experienced by girls during the sexual and reproductive health changes occuring during puberty and adolescence

Linked Memos: 0

Excerpt Creator: tcuhawenimana11

Excerpt Created On: 4/28/2023

Excerpt Range: 31875-32175

Ndi nimero 6 mfite imyaka 13 niga muwa 1. Ingorane umwana wumukobwa yagira mu gihe cyubwangavu atabonye umugira inama ngo amenye ibintu nyine byinshi ashobora kujya gusambana maze ubundi agatwara inda, ashobora no kurwara indwara zandurira mu mibonano mpuzabitsina nka sida cyangwa se mburugu n’ibyo.

Title: G S RWAMAGANA Girls TRANSCRIBED.docx

Doc Creator: tcuhawenimana11

Doc Date: 3/25/2023

Codes Applied: Obstacles and risks experienced by girls during the sexual and reproductive health changes occuring during puberty and adolescence

Linked Memos: 0

Excerpt Creator: tcuhawenimana11

Excerpt Created On: 4/28/2023

Excerpt Range: 32274-32988

Murakoze nimero yange ni 5 mfite imyaka 16 niga senior 1. Ingorane umwana wumwangavu ashobora guhura nazo icyambere iyo umukobwa atagirwa inama cyane ashobora kugira ingaruka nyinshi kandi mbi. Iya mbere iyo umukobwa ageze mu gihe cy’imihango aba akeneye inama nyinshi cyane kuko mubyiyumviro bye aba yumva agomba kugira inshuti yumuhungu ndetse naho ari hose akumva abakobwa ntabwo abiyumvamo cyane ahubwo akumva agomba guhora aganira nabahungu, muruko kuganira nabahungu atagize nyine umuba hafi ngo amugire inama amukosore ati ibyo urimo ni byiza cyangwa ntabwo ari byiza kugirango ubone uko ubyitwaramo. Iyo atagize umujyanama umugira inama muricyo gihe aba agezemo rero bimugiraho ingaruka nyinshi kandi mbi.

Title: G S RWAMAGANA Girls TRANSCRIBED.docx

Doc Creator: tcuhawenimana11

Doc Date: 3/25/2023

Codes Applied: Obstacles and risks experienced by girls during the sexual and reproductive health changes occuring during puberty and adolescence

Linked Memos: 0

Excerpt Creator: tcuhawenimana11

Excerpt Created On: 4/28/2023

Excerpt Range: 33322-33598

Murakoze ndi nimero 8 mfite imyaka 13 niga mu mwaka wa mbere wamashuri yisumbuye. Ikintu kibitera ntago ababyeyi bakibegera ngo babagiire inama cyane ahubwo hasigaye haraje ikoranabuhanga rituma batandukira nyine bakareba ibintu bitandukanye ababyeyi ntibabahane bakabihorera.

Title: G S RWAMAGANA Girls TRANSCRIBED.docx

Doc Creator: tcuhawenimana11

Doc Date: 3/25/2023

Codes Applied: Obstacles and risks experienced by girls during the sexual and reproductive health changes occuring during puberty and adolescence

Linked Memos: 0

Excerpt Creator: tcuhawenimana11

Excerpt Created On: 4/28/2023

Excerpt Range: 33621-34476

Murakoze ndi nimero 9 mfite imyaka 17 niga mu mwaka wa 5. Inzitizi urubyiruko ruhura nazo rugeze mu gihe cyubugimbi nubwangavu ababyeyi bakunze kuba bitaye ku kazi cyane cyane kuruta abana bakavuga ngo ntamwanya bababonera nuwo babonye muto bakaruhuka abana bakajya kumbugankoranyambaga bakaba arizo bahugiraho bakaba ariho bashakira amakuru rimwe na rimwe bakabaha ayibihuha cyangwa atariyo bakagendera muruwo murongo bikabayobya kandi bitwaga ko ariho bagiye kubonera ibisubizo cyangwa ababyeyi bakaba batameranye neza bahora mu ntonganya ntamwanya wo kuganiriza abana babo nta mwanya wo kubitaho umwana akavuga ati aho kugirango nicare ntamakuru mfite akabaza inshuti ye wenda ifite nkibyo bibazo ikayamuha nabi, akabaza abandi bantu atizeye bakayamuha nabi akagendera mu kigare we ashobora kuba yumva atari namakosa bikamushora mu ngeso mbi, murakoze.

Title: G S RWAMAGANA Girls TRANSCRIBED.docx

Doc Creator: tcuhawenimana11

Doc Date: 3/25/2023

Codes Applied: Suggestions to improve SRH during puberty and adolescence by girls

Linked Memos: 0

Excerpt Creator: tcuhawenimana11

Excerpt Created On: 4/28/2023

Excerpt Range: 34715-35409

Murakoze ndi nimero 9 mfite imyaka 17 niga mu mwaka wa 5. Ikindi nakongeraho ni nkinyunganizi naha nkababyeyi ko bagira umwanya baharira abana cyangwa bakuru bacu ko bagira amakuru baduha bakareka guhugira mu kazi nibindi bibahugije. Nkongera no gushisikariza urubyiruko kutita kubabashuka no kugendera mu makuru batizeye bakaba bakwegera ababyeyi bakaba babaza abavandimwe bo muri famille amakuru kandi bakareka kumva ko kuba bageze mu gihe cyubwangavu nubugimbi bagomba kunanirana ntibumve ibyo ababyeyi bababwira ntibakurikize inama zabakuru bakumva bakigenga cyangwa bakurikiza inama zaba group bagenzi babo bityo bikaba byabashora mu ngeso mbi kandi aribo rubyiruko rwejo hazaza, murakoze.

Title: G S RWAMAGANA Girls TRANSCRIBED.docx

Doc Creator: tcuhawenimana11

Doc Date: 3/25/2023

Codes Applied: Suggestions to improve SRH during puberty and adolescence by girls

Linked Memos: 0

Excerpt Creator: tcuhawenimana11

Excerpt Created On: 4/28/2023

Excerpt Range: 35540-36889

Murakoze ndi nimero 7 mfite imyaka 19 niga mu mwaka wa 5. Nange ikintu nakongeraho nugushishikariza urubyiruko bagenzi bange ko batagomba kumva amakuru yabantu bose, abantu bose bagenda baduha amakuru ntabwo ariko bagenda baduha amakuru meza hari bamwe bagenda badushuka baduha amakuru yo kutujyana mu ngeso zitari nziza. Ikindi nakongera kubabwira nukugisha inama ibintu byose biba kubuzima bwacu ntago aruko tuba tubifitiye ibisubizo ahubwo tuba tugomba kwegera umuntu twisanzuyeho tukegera nkababyeyi cyane cyane, ukegera nka mukuru wawe igihe umufite wisanzuraho, abaturanyi wisanzuraho, icyo gihe iyo ubonye abo bose ntanumwe wisanzuraho ukajya mu cyumba cyurubyiruko ukegera umuganga ubona wisanzura ukagenda ukamuganiriza ukamusobanuza nyine ku buzima bwimyororokere akagusobanurira. Ikindi nabwira urubyiruko nuko tugomba kunyurwa nuko tubayeyo kuko twese muriyi si ntabwo tubayeho ubuzima bumwe, hari ababayeho mu buzima butari bwiza ariko hari nababayeho mu buzima bwiza nyine bumva bishimiye. Urubyiruko rero ntabwo tugomba gutega abantu bose amatwi cyangwa gukurikira amakuru ku mbuga nkoranyambaga kuko ibintu byose bivugirwaho ntago aba ari byiza ahubwo tugomba kureba umuntu twizeye twisanzuraho kuburyo dushobora kumubwira buri kimwe cyose akatubwira amakuru yingenzi byatunanira tukajya kukigonderabuzima bakadusobanurira, murakoze.

Title: MUKARANGE GIRLS transcri.docx

Doc Creator: tcuhawenimana11

Doc Date: 3/25/2023

Codes Applied: Physiological changes occuring during puberty-Boys

Linked Memos: 0

Excerpt Creator: tcuhawenimana11

Excerpt Created On: 4/29/2023

Excerpt Range: 106-857

Murakoze, nitwa nimero 5 mfite imyaka 17 niga mu wa kabiri

Kuniga ijwi, no kumera ubwanwa, kumera insya, ubucakwaha

Nitwa nimero 4 mfite imyaka 16 niga mu mwaka wa mbere impinduka nyine naba nzi ndumva yazivuze

Murakoze, nimero yanjye ni 6 umwaka ni uwa kabiri impinduka zigaragara ku muhungu harimo kumera insya, kumera ubucakwaha, kuniga ijwi n igihe agezemo cyo kwiroteraho.

Nitwa nimero 7 imyaka 15 umwaka wa mbere impinduka nzi nanjye bazivuze ariko icyo gihe gitangira kuza iyo umwana w umuhungu ageze mu myaka 14, murakoze.

Nimero yanjye ni 6 umwaka ni uwa kabiri, imyaka ni 18, iyindi mpinduka iba k umuhungu harimwo no kumera impemwe, ikindi hari igihe wenda nki imisemburo imubana myinshi mu mubiri akaba yagira ni ibiheri akabigaragaza.

Title: MUKARANGE GIRLS transcri.docx

Doc Creator: tcuhawenimana11

Doc Date: 3/25/2023

Codes Applied: Psychological changes occuring during puberty-Boys

Linked Memos: 0

Excerpt Creator: tcuhawenimana11

Excerpt Created On: 4/29/2023

Excerpt Range: 858-1175

Murakoze, nimero ni 8 imyaka 15 niga mu wambere, impinduka nzi ndumva bazivuze gusa nyine izindi nziko umuhungu iyo ageze mu gihe cyu ubugimbi abayumva nyine yakuze nawe ageze muri cyagihe nyine yumva yarabaye umugabo kuriwe nyine mu ibitekerezo bye, ku buryo nyine usanga atangiye kubaka imibiri yubaka ituza nyine,

Title: MUKARANGE GIRLS transcri.docx

Doc Creator: tcuhawenimana11

Doc Date: 3/25/2023

Codes Applied: Physiological changes occuring during puberty-Boys

Linked Memos: 0

Excerpt Creator: tcuhawenimana11

Excerpt Created On: 4/29/2023

Excerpt Range: 1175-1392

azana izo mpinduka zitandukanye ku mubiri, azana ibishishi mu maso,baniga injwi, nyine muri bimwe bagiye bavuga, nawe nyine yakwireba akavuga ati”nabaye umusore sinkiri umwana” hari nubwo atangira ka niteretere abana.

Title: MUKARANGE GIRLS transcri.docx

Doc Creator: tcuhawenimana11

Doc Date: 3/25/2023

Codes Applied: Psychological changes occuring during puberty-Boys

Linked Memos: 0

Excerpt Creator: tcuhawenimana11

Excerpt Created On: 4/29/2023

Excerpt Range: 1440-1729

Urabona wenda guturuka ku 10 kumanura hasi,aba abona nyine iwabo bamwita Kadogo nyine ni injwi rye rikamugaragaza ariko yagera muri iyi myaka nyine yo kuba ingimbi agatangira ijwi e akumva niba na bakuru be atangiye kuvuga nkabo ati ubu nanjye sinkiri umwana nakuze irya kadogo akaryanga.

Title: MUKARANGE GIRLS transcri.docx

Doc Creator: tcuhawenimana11

Doc Date: 3/25/2023

Codes Applied: Boys and girls know about the causes leading to the physiological changes during puberty

Linked Memos: 0

Excerpt Creator: tcuhawenimana11

Excerpt Created On: 4/29/2023

Excerpt Range: 1730-1956

Urakoze ndi nimero kabiri nkaba niga mu mwaka wa wa kabiri w amashuri yisumbuye mfite imyaka 15 impamvu mbona ibyo byose biba ni imisemburo ni imisemburo y umuntu ibitera hari iyitwa presisetogene ntabwo mbashije kuyibuka.

Title: MUKARANGE GIRLS transcri.docx

Doc Creator: tcuhawenimana11

Doc Date: 3/25/2023

Codes Applied: Boys and girls know about the causes leading to the physiological changes during puberty

Linked Memos: 0

Excerpt Creator: tcuhawenimana11

Excerpt Created On: 4/29/2023

Excerpt Range: 1996-2052

Nibimenyetso bigaragaza umuhungu ugeze mugihe cy ubukure

Title: MUKARANGE GIRLS transcri.docx

Doc Creator: tcuhawenimana11

Doc Date: 3/25/2023

Codes Applied: Physiological changes occuring during puberty-Boys

Linked Memos: 0

Excerpt Creator: tcuhawenimana11

Excerpt Created On: 4/29/2023

Excerpt Range: 2085-2112

Ni uguhera nyine yiroteyeho

Title: MUKARANGE GIRLS transcri.docx

Doc Creator: tcuhawenimana11

Doc Date: 3/25/2023

Codes Applied: Boys and girls know about the causes leading to the physiological changes during puberty

Linked Memos: 0

Excerpt Creator: tcuhawenimana11

Excerpt Created On: 4/29/2023

Excerpt Range: 2190-2240

Murakoze nimero yanjye ni 6 niuko nyine aba yakuze

Title: MUKARANGE GIRLS transcri.docx

Doc Creator: tcuhawenimana11

Doc Date: 3/25/2023

Codes Applied: Boys and girls know about the causes leading to the physiological changes during puberty

Linked Memos: 0

Excerpt Creator: tcuhawenimana11

Excerpt Created On: 4/29/2023

Excerpt Range: 2362-2494

Murakoze ndi nimero ya 3 niga mu mwaka wa 2 w amashuri yisumbuye izo mpinduka mbona ziterwa n imisemburo nk uko babivuze ku bahungu.

Title: MUKARANGE GIRLS transcri.docx

Doc Creator: tcuhawenimana11

Doc Date: 3/25/2023

Codes Applied: Physiological changes occuring during puberty-Girls

Linked Memos: 0

Excerpt Creator: tcuhawenimana11

Excerpt Created On: 4/29/2023

Excerpt Range: 2495-2624

Impinduka ya mbere igaragara ku mwana w umukobwa ni ukumera amabere, incakwaha, insya, kuzana ibishishi mu maso kujya mu mihango.

Title: MUKARANGE GIRLS transcri.docx

Doc Creator: tcuhawenimana11

Doc Date: 3/25/2023

Codes Applied: Boys and girls know about the causes leading to the physiological changes during puberty

Linked Memos: 0

Excerpt Creator: tcuhawenimana11

Excerpt Created On: 4/29/2023

Excerpt Range: 2646-2657

Imisemburo.

Title: MUKARANGE GIRLS transcri.docx

Doc Creator: tcuhawenimana11

Doc Date: 3/25/2023

Codes Applied: Physiological changes occuring during puberty-Girls

Linked Memos: 0

Excerpt Creator: tcuhawenimana11

Excerpt Created On: 4/29/2023

Excerpt Range: 2775-2798

Habanje kumera amabere

Title: MUKARANGE GIRLS transcri.docx

Doc Creator: tcuhawenimana11

Doc Date: 3/25/2023

Codes Applied: How girls experience changes occuring to them during puberty

Linked Memos: 0

Excerpt Creator: tcuhawenimana11

Excerpt Created On: 4/29/2023

Excerpt Range: 2943-3274

Nta makuru nari nyifiteho ahubwo byarantunguye cyane, nyine mfite nk imyaka nka 13, najyaga mbona abandi bafite amabere nkavuga nti ese buriya njye azaza noneho arimyoza njye araseka ariko yarandiye.

Imihango nyine nkiyijyamo byarantunguye naratekereje nti ese ibi ni ibiki byagenze bite mbanza no kugira ngo wenda nanakomeretse.

Title: MUKARANGE GIRLS transcri.docx

Doc Creator: tcuhawenimana11

Doc Date: 3/25/2023

Codes Applied: How girls experience changes occuring to them during puberty

Linked Memos: 0

Excerpt Creator: tcuhawenimana11

Excerpt Created On: 4/29/2023

Excerpt Range: 3351-3407

Nyine twarabyigaga ariko njewe nyine nkumwa bitazanabaho

Title: MUKARANGE GIRLS transcri.docx

Doc Creator: tcuhawenimana11

Doc Date: 3/25/2023

Codes Applied: How girls experience changes occuring to them during puberty

Linked Memos: 0

Excerpt Creator: tcuhawenimana11

Excerpt Created On: 4/29/2023

Excerpt Range: 3497-3647

Ntabwo nayihereranye nashatse nyine umuntu mukuru nizeye ndabimutekerereza mubwira uko byangendekeye aranggije arambwira ati nyine ubwo urimo urakura.

Title: MUKARANGE GIRLS transcri.docx

Doc Creator: tcuhawenimana11

Doc Date: 3/25/2023

Codes Applied: How girls experience changes occuring to them during puberty

Linked Memos: 0

Excerpt Creator: tcuhawenimana11

Excerpt Created On: 4/29/2023

Excerpt Range: 3719-3930

wo muntu mukuru rero nabibwiye ni nshuti yanjye naragiye ndabiyibwira nyine nyitekerereza ukuntu nyine byagenze byose ntacyo nyihishe irangije irambwira ati nabandi bibabaho ihangane si wowe wenyine bibayeho.

Title: MUKARANGE GIRLS transcri.docx

Doc Creator: tcuhawenimana11

Doc Date: 3/25/2023

Codes Applied: How girls experience changes occuring to them during puberty

Linked Memos: 0

Excerpt Creator: tcuhawenimana11

Excerpt Created On: 4/29/2023

Excerpt Range: 4007-4225

Narayibibwiye irangije nyine irambwira ati ubigenza gutya na gutya ufata cotex ukabigira gutya na gutya ndangije ndayibwira ati nibinyereke nanjye mbyige uwo munsi irabinyereka ndabyiga ndabimenya cotex yarayimpaye

Title: MUKARANGE GIRLS transcri.docx

Doc Creator: tcuhawenimana11

Doc Date: 3/25/2023

Codes Applied: Sexuality related practices girls do during puberty and adolescence

Linked Memos: 0

Excerpt Creator: tcuhawenimana11

Excerpt Created On: 4/29/2023

Excerpt Range: 4284-4798

Nawe yarabimenye ariko we nabimubwiye nyuma

Nimero yanjye ni 4 mfite imyaka 16 nanjye ndikumva nyine impinduka hari ukuntu nyine iyo umukobwa ageze mu ntuza mu bwangavu batangira kumubwira ukutu baca imyeyo biriya by imihango nyine bakabimubwira mama yari yarabinganirijeho byose nyine bikiza ntabwo byigeze bintungura cyane kubera ko nyine mama namwisanzuragaho byose ndabimubwira ambwira uko nyine bakoresha cotex ni aho ngaho nyine nabimenyeye guca imyeyo byo ni umwana w inshuti yanjye wabinyeretse.

Title: MUKARANGE GIRLS transcri.docx

Doc Creator: tcuhawenimana11

Doc Date: 3/25/2023

Codes Applied: Sexuality related practices girls do during puberty and adolescence

Linked Memos: 0

Excerpt Creator: tcuhawenimana11

Excerpt Created On: 4/29/2023

Excerpt Range: 4863-5058

Ikintu nyine yambwiye yarambwiye ati ntiwashaka umugabo utaraciye imyeyo ibindi byo ntabyo yigeze ambwira gusa nyine gusa nyine mba numva izo mpinduka ziterwa n imisemburo myinshi yo mu mubiri.

Title: MUKARANGE GIRLS transcri.docx

Doc Creator: tcuhawenimana11

Doc Date: 3/25/2023

Codes Applied: How girls experience changes occuring to them during puberty

Linked Memos: 0

Excerpt Creator: tcuhawenimana11

Excerpt Created On: 4/29/2023

Excerpt Range: 5189-5274

Nyine batubwira ko ari imisembura yo mu mubiri gusa nyine ntabwo tuzi ibyo ari byo.

Title: MUKARANGE GIRLS transcri.docx

Doc Creator: tcuhawenimana11

Doc Date: 3/25/2023

Codes Applied: Physiological changes occuring during puberty-Girls

Linked Memos: 0

Excerpt Creator: tcuhawenimana11

Excerpt Created On: 4/29/2023

Excerpt Range: 5275-5444

Murakoze nimero yanjye ni 5 mfite imyaka 17 niga mu wa kabiri ndumva impinduka ari ukumera amabere ,kumera insya kujya mu mihango, incakwaha , kugira ibishishi mu maso .

Title: MUKARANGE GIRLS transcri.docx

Doc Creator: tcuhawenimana11

Doc Date: 3/25/2023

Codes Applied: How girls experience changes occuring to them during puberty

Linked Memos: 0

Excerpt Creator: tcuhawenimana11

Excerpt Created On: 4/29/2023

Excerpt Range: 5445-6008

Njyewe nkijya mu miango naricuye nyine mu gitondo mbona haje amaraso ndibaza ati byagenze gute ubwoba buranyica nyine mbyutsa mama ndamubaza gusa nta kintu yari yarugeze ambwira nibyo bikoreshonyine ngo ni cotex yarambwiraga ngo ni imigati sinari nzi ibyo ari byo nkamubaza aransobanurira arangije arambwira ati ubu wakuze witware neza ushobora gukora uburara ugakora imibonano mpuza bitsina ukaba watwara inda anyereka nizo cotex uko zikoreshwa nyine ngira ubwoba najya ngenda no mumuhanda nkabona bari kubibona ,nkabona bari kunseka nyine ni uko byaje.

Title: MUKARANGE GIRLS transcri.docx

Doc Creator: tcuhawenimana11

Doc Date: 3/25/2023

Codes Applied: How to take care of themselves when phsyiological changes occur for girls Precautions to take during puberty for girls

Linked Memos: 0

Excerpt Creator: tcuhawenimana11

Excerpt Created On: 4/29/2023

Excerpt Range: 6050-6200

Yambwiya ko nakuze amwira ko umukobwa uwariwe wese wabashije kujya mu mihango yatwara inda ngo ashobora gukora imibonano mpuzabitsina gatwara inda.

Title: MUKARANGE GIRLS transcri.docx

Doc Creator: tcuhawenimana11

Doc Date: 3/25/2023

Codes Applied: Received information is misleading and risky

Linked Memos: 0

Excerpt Creator: tcuhawenimana11

Excerpt Created On: 4/29/2023

Excerpt Range: 6277-6364

Nyine wenda nka nyuma y iminsi 8 cg 9uvuye mu mihango ngo ntago ushobora gusama inda .

Title: MUKARANGE GIRLS transcri.docx

Doc Creator: tcuhawenimana11

Doc Date: 3/25/2023

Codes Applied: Received information is misleading and risky

Linked Memos: 0

Excerpt Creator: tcuhawenimana11

Excerpt Created On: 4/29/2023

Excerpt Range: 6423-6529

Iyo minsi 7 cg 8 iyo irangiye uvuye mu mihango ushobora gusama ariki igihe uri mu mihango ndumva utasama

Title: MUKARANGE GIRLS transcri.docx

Doc Creator: tcuhawenimana11

Doc Date: 3/25/2023

Codes Applied: Physiological changes occuring during puberty-Girls

Linked Memos: 0

Excerpt Creator: tcuhawenimana11

Excerpt Created On: 4/29/2023

Excerpt Range: 6623-6869

Amazina yanje ni 6 umwaka ni uwa 2 imyaka ni 18 njyewe ukuntu mbyumva impinduka zigaragara ku mukobwa igihe yabaye umwangavu hari ukuba yamera amabere , kuba yajya mu mihango kuba nyine yatangira kugira ikibuno no kuba yazana ibishishi mu maso .

Title: MUKARANGE GIRLS transcri.docx

Doc Creator: tcuhawenimana11

Doc Date: 3/25/2023

Codes Applied: How girls experience changes occuring to them during puberty

Linked Memos: 0

Excerpt Creator: tcuhawenimana11

Excerpt Created On: 4/29/2023

Excerpt Range: 6943-7001

Oya biterwa nyine n ukuntu nyine imisemburo ye iba imeze.

Title: MUKARANGE GIRLS transcri.docx

Doc Creator: tcuhawenimana11

Doc Date: 3/25/2023

Codes Applied: Received information is misleading and risky

Linked Memos: 0

Excerpt Creator: tcuhawenimana11

Excerpt Created On: 4/29/2023

Excerpt Range: 7043-7339

Umuti? Hari igihe bakubwira ngo nukora imibonano mpuzabitsina biragenda gusa ntabwo aba aribyo nyine hari nk umukobwa ugenda akabiganiriza nk cheri we kamubwira ati nagize ikibazo nazanye ibiheri mbwira nyine umuti akamubwira ngo nakora imibonano mpuzabitsina bigenda we agamije kuba mwaryamana.

Title: MUKARANGE GIRLS transcri.docx

Doc Creator: tcuhawenimana11

Doc Date: 3/25/2023

Codes Applied: Boys and girls know about the causes leading to the physiological changes during puberty

Linked Memos: 0

Excerpt Creator: tcuhawenimana11

Excerpt Created On: 4/29/2023

Excerpt Range: 7359-7437

Niyihe? Gsa ntabwo nzi izina ryayo, twarayize ariko ntabwo nibuka izina ryayo.

Title: MUKARANGE GIRLS transcri.docx

Doc Creator: tcuhawenimana11

Doc Date: 3/25/2023

Codes Applied: How girls experience changes occuring to them during puberty

Linked Memos: 0

Excerpt Creator: tcuhawenimana11

Excerpt Created On: 4/29/2023

Excerpt Range: 7528-7773

Njyewe nyine ikintu cyambayeho nyine nkumva kirantunguye nazanye isya, numva ni ibintu bitabaho nangira kumvako ari indwara ndwaye muri njyewe,mbiganiriza mama arambwira ati wakuze ahubwo itwararike nyine ngirango nibyo ngibyo gusa nyine biraho.

Title: MUKARANGE GIRLS transcri.docx

Doc Creator: tcuhawenimana11

Doc Date: 3/25/2023

Codes Applied: How to take care of themselves when phsyiological changes occur for girls

Linked Memos: 0

Excerpt Creator: tcuhawenimana11

Excerpt Created On: 4/29/2023

Excerpt Range: 7838-7877

Ego nyine yarambwiye ngo ujye uzogosha.

Title: MUKARANGE GIRLS transcri.docx

Doc Creator: tcuhawenimana11

Doc Date: 3/25/2023

Codes Applied: How girls experience changes occuring to them during puberty

Linked Memos: 0

Excerpt Creator: tcuhawenimana11

Excerpt Created On: 4/29/2023

Excerpt Range: 7878-7901

Ikindi kujya mu mihango

Title: MUKARANGE GIRLS transcri.docx

Doc Creator: tcuhawenimana11

Doc Date: 3/25/2023

Codes Applied: How girls experience changes occuring to them during puberty

Linked Memos: 0

Excerpt Creator: tcuhawenimana11

Excerpt Created On: 4/29/2023

Excerpt Range: 7951-8025

Njyewe nagize ubwoba cyane, nkumva nyine nshobora kuba nakomeretse ntabizi

Title: MUKARANGE GIRLS transcri.docx

Doc Creator: tcuhawenimana11

Doc Date: 3/25/2023

Codes Applied: How girls experience changes occuring to them during puberty

Linked Memos: 0

Excerpt Creator: tcuhawenimana11

Excerpt Created On: 4/29/2023

Excerpt Range: 8071-8302

Amakuru yo nari narayumviseho nyine bakabwirako nyine iyo umukobwa ageze mu igihe cyu ubwangavu ajya mu mihango gusa nyine njyewe nkumva ari inzozi kuri njyewe ko ntanjya mu mihango nkumva nyine ari ibya abandi ko njye bitangeraho.

Title: MUKARANGE GIRLS transcri.docx

Doc Creator: tcuhawenimana11

Doc Date: 3/25/2023

Codes Applied: How girls experience changes occuring to them during puberty

Linked Memos: 0

Excerpt Creator: tcuhawenimana11

Excerpt Created On: 4/29/2023

Excerpt Range: 8396-8488

Oya nyine urumva numvaga ko nyine njye bitangereho, ntabwo nabitekerezaga ngo mbihe umwanya.

Title: MUKARANGE GIRLS transcri.docx

Doc Creator: tcuhawenimana11

Doc Date: 3/25/2023

Codes Applied: How girls experience changes occuring to them during puberty

Linked Memos: 0

Excerpt Creator: tcuhawenimana11

Excerpt Created On: 4/29/2023

Excerpt Range: 8533-8732

Nkumva nyine ntacyintu bintwaye gusa nabigiyemwo nabibwiye mummy arambwira nyine ambwira ukuntu cotex cg pade ukuntu zikoreshwa igihe wagiye mu mihango,arabinyigisha ndabimenya ntagira kujya mbikora.

Title: MUKARANGE GIRLS transcri.docx

Doc Creator: tcuhawenimana11

Doc Date: 3/25/2023

Codes Applied: How girls experience changes occuring to them during puberty

Linked Memos: 0

Excerpt Creator: tcuhawenimana11

Excerpt Created On: 4/29/2023

Excerpt Range: 9060-9435

Nimero yanjye ni 7, njyewe njya mu mihango nyine narinsanzwe byumva babivuga, byuka mu gitondo nyine mbona nagiye mu mihango ariko nyine mbanza kugiramwo ubwoba, ndagenda mbwira mama ngo ko mbona ndikuva amaraso? Mama arambwirango nyine wagiye mu mihango wakuze, ampereza cotex anyereka uko nyine bayikoresha,arambwira ati ujye ugira isuku,ukarabe nyine nka abakobwa bakuze.

Title: MUKARANGE GIRLS transcri.docx

Doc Creator: tcuhawenimana11

Doc Date: 3/25/2023

Codes Applied: How girls experience changes occuring to them during puberty

Linked Memos: 0

Excerpt Creator: tcuhawenimana11

Excerpt Created On: 4/29/2023

Excerpt Range: 9611-9803

Narababaye munda, nyine mama nibwo yaje akansobanurira uti iyo umukobwa yagiye mu mihango buri wese afite uko ababara, hari ubabara umutwe nahandi hatandukanye arabwira ati nyine nuko bigenda.

Title: MUKARANGE GIRLS transcri.docx

Doc Creator: tcuhawenimana11

Doc Date: 3/25/2023

Codes Applied: How girls experience changes occuring to them during puberty

Linked Memos: 0

Excerpt Creator: tcuhawenimana11

Excerpt Created On: 4/29/2023

Excerpt Range: 10047-10164

Oya, byaje rimwe birahagarara ,nyine mbi mbwiye mama nyine arambwira uti kubera ari ubwa mbere uyigiyemwo bizagaruka.

Title: MUKARANGE GIRLS transcri.docx

Doc Creator: tcuhawenimana11

Doc Date: 3/25/2023

Codes Applied: How girls experience changes occuring to them during puberty

Linked Memos: 0

Excerpt Creator: tcuhawenimana11

Excerpt Created On: 4/29/2023

Excerpt Range: 10258-12617

Murakoze, ndi nimero 8, njyewe nyigera nyine mu guhe cyu ubwangavu,ubundi njyewe inshuti zanjye ko bo bigaga muwa 6 kandi aribo banduta babanje kubijyamwo tukiri ku kigo, nkabona nyine bararira barikanda munda nkumva njyewe rwose ni ibintu binsekeje uti nkibyo bintu bibabaho mukikanda gutyo ngo ni imihango ni ngo njye icyayimpa,kuko nyine njye sinarinziko iyo uyigiyemwo mba mbere ikubabaza,ariko ubwo mu byara wanjye nawe nkajya mbona abijyamwo ariko simenye ngo haza amaraso noneho sinarinzingo pade ikora iki ahubwo njye narinziko pade ari pampegisi za abantu bakuru nkabona arashyira mu ikariso ariko icyikintu ntuyu aba ashaka kunyara mu myenda araseka si narinzingo pade impara iki,noneho mu gihe nanjye nyigiyemwo narabyutse ari mu gitondo nsanga nyine nanabimenye ngiye muri w.c,ngiye ku nyara manuye ikariso mbona yuzuye amaraso ndapfuye noneho mama aragirango naryamanye yari yarambwiye ngo nuramuka uryamanye nkabimeya nzakwica noneho ndamubwirako nakomeretse ndimubiki, nuko yinjiye munzu kuko twararanaga abona ishuka yuzuyeho amaraso noneho ni inyuma ku ikanzu nari nanduye ariko ntatabibonye,ndagenda arambwirango wakuze se muko? Ndaceceka,ngo do reba ibyo wankoreye kumashuka,ndaceceka ariko munda hatangira kurya bigeze aho birakara nti mama ndapfuye ati ese ubundi wabaye iki?ariko we abizi ngo nagiye muri w.c nsanga ikariso yanjye iriho amaraso, ubwo wagiye mu mihango ariko we na musaza wanjye babivugaga mu marenga, musaza wanjye akavuga ngo ese uwo mukobwa wawe ntabwo aranjya muruhango? Ariko ubwo mu ruhango bavuga njye nkumva ari mu ruhango bajya naho ni imihango bari kuvuga ngo reka reka ngo ese ko yatinze kandi mukuru we yayigiyemwo afite imyaka mike? Ariko bikancanga ngo ntabwo aranjya mu ruhango? Uwo munsi rero nayigiyemwo ahita avuga ngo vayo tugende ngo reka njye kukuzanira pade, musaza awanjye yararyamye ahita avuga ngo yagiye muruhango? Nkibaza ukuntu nagiye mu ruhango kandi ndi murugo,aragenda nyine azana pade ariko nyine siyambwiyengo ngo bayambara gute kuko nabibonaga kuri mubyara wanjye uko yabyambaraga ndambara nkimaramwo iminsi 3 ariko ubwo ntari ntaramenya gukoresha pade uko bayikoresha kuko nayivagamwo nayo yashize uko nabonaga igiyeho amaraso nahitaga njugunya nuko nyine bageraho baranyigisha ngo nyine umukobwa wagiya mu mihango agomba nyine kuzajya akaraba kugirango bitazanuka na abndi bakabyumva.

Title: MUKARANGE GIRLS transcri.docx

Doc Creator: tcuhawenimana11

Doc Date: 3/25/2023

Codes Applied: Pshcological changes occuring during puberty-Girls

Linked Memos: 0

Excerpt Creator: tcuhawenimana11

Excerpt Created On: 4/29/2023

Excerpt Range: 12653-12968

Nonese ukuntu watangira kumva ko wakuze, mu gihe uri mu myaka 5 kuzamuka kugeza 10, byo ntabwouba wakabyibonaho, naho iyo ugeze ahandi ugatangira kubona ugiye mu mihango,uzanye amabere nyine iwanyu barakubwirango wakuze nyine itwararike wabaye inkumi, ubwo nyine nawe ntabwo wabitereho comment nawe wumva ko wakuze.

Title: MUKARANGE GIRLS transcri.docx

Doc Creator: tcuhawenimana11

Doc Date: 3/25/2023

Codes Applied: Pshcological changes occuring during puberty-Girls

Linked Memos: 0

Excerpt Creator: tcuhawenimana11

Excerpt Created On: 4/29/2023

Excerpt Range: 13060-13302

Mu mitekerereze nyine wumvako nawe wakuze mu mikorere nyine iyo warukiri hasi nyine mu bwana wabaga uri wamuntu usa nabi, ugira umwanda ariko iyo ugeze muri icyo kiciro bakakubwira ngo wakuze utangira kwitwara egoko nka abakobwa nyine bakuze.

Title: MUKARANGE GIRLS transcri.docx

Doc Creator: tcuhawenimana11

Doc Date: 3/25/2023

Codes Applied: Pshcological changes occuring during puberty-Girls

Linked Memos: 0

Excerpt Creator: tcuhawenimana11

Excerpt Created On: 4/29/2023

Excerpt Range: 13341-13529

Ingendo irahinduka,imyambarire igahinduka,ijwi rigahinduka, no mumikorere nyine ibintu byawe byose ubundi iwanyu nyine bakaba bakuziho nyine imyaka warufite utaragera mu ghe cyu ubwangavu.

Title: MUKARANGE GIRLS transcri.docx

Doc Creator: tcuhawenimana11

Doc Date: 3/25/2023

Codes Applied: Pshcological changes occuring during puberty-Girls

Linked Memos: 0

Excerpt Creator: tcuhawenimana11

Excerpt Created On: 4/29/2023

Excerpt Range: 13664-14037

Nimero ni 6 umwaka ni uwa kabiri, imyaka ni 18,iyo babikubwiye nawe muri wowe wumvako nubundi wabaye inkumi, niba wenda wamaraga iminsi nki 3 udakaraba bagukubitira koga icyo gihe nawe wumvako wakuze ugatangira nawe kuzajya wigirira isuku,ukamenya gukaraba, ukamenya niba uyu munsi ngomba gukaraba nejo nkazakaraba ibyo gusiba nyine ukamara iminsi ibyo urabisiba ukabireka

Title: MUKARANGE GIRLS transcri.docx

Doc Creator: tcuhawenimana11

Doc Date: 3/25/2023

Codes Applied: Pshcological changes occuring during puberty-Girls

Linked Memos: 0

Excerpt Creator: tcuhawenimana11

Excerpt Created On: 4/29/2023

Excerpt Range: 14155-14253

Iyo wageze nyine mu cyiciro nyine nkicyo ngicyo nawe muri wowe uriyumva ukumvako nyine wahindutse.

Title: MUKARANGE GIRLS transcri.docx

Doc Creator: tcuhawenimana11

Doc Date: 3/25/2023

Codes Applied: Pshcological changes occuring during puberty-Girls

Linked Memos: 0

Excerpt Creator: tcuhawenimana11

Excerpt Created On: 4/29/2023

Excerpt Range: 14268-14378

Iyo wageze muri icyo gihe utangira guteretwa, utangira kumvako aho uciye hose nyine umuntu ari bukurebe, yego.

Title: MUKARANGE GIRLS transcri.docx

Doc Creator: tcuhawenimana11

Doc Date: 3/25/2023

Codes Applied: Pshcological changes occuring during puberty-Girls

Linked Memos: 0

Excerpt Creator: tcuhawenimana11

Excerpt Created On: 4/29/2023

Excerpt Range: 14510-14767

Njyewe se? njyewe ntabwo nari nanjya mu mihango.

Mugihe umwana wu umukobwa ageze mu gihe cyu ubwanagavu wumva ari ayahe makuru yakenera mu bijyanye nu ubuzima bwi imyororokere ndetse ni imihindagurukire yu ubuzima bwe,ni ayahe makuru wumva yakenera kumenya?

Title: MUKARANGE GIRLS transcri.docx

Doc Creator: tcuhawenimana11

Doc Date: 3/25/2023

Codes Applied: How to take care of themselves when phsyiological changes occur for girls

Linked Memos: 0

Excerpt Creator: tcuhawenimana11

Excerpt Created On: 4/29/2023

Excerpt Range: 14823-14991

Amakuru wakenera kumenya nku umwana wu umukobwa ugeze mu gihe cyu ubwangavu? Ukuntu wakwitwara?

Ni ukugira isuku, aho unyuze hose ukazanjya uhanyura usa neza wakarabye.

Title: MUKARANGE GIRLS transcri.docx

Doc Creator: tcuhawenimana11

Doc Date: 3/25/2023

Codes Applied: How to take care of themselves when phsyiological changes occur for girls

Linked Memos: 0

Excerpt Creator: tcuhawenimana11

Excerpt Created On: 4/29/2023

Excerpt Range: 15010-15051

Nyine wambaye imyambaro imeshe yi imbere.

Title: MUKARANGE GIRLS transcri.docx

Doc Creator: tcuhawenimana11

Doc Date: 3/25/2023

Codes Applied: How to take care of themselves when phsyiological changes occur for girls

Linked Memos: 0

Excerpt Creator: tcuhawenimana11

Excerpt Created On: 4/29/2023

Excerpt Range: 15081-15292

Nyine umwambaro w imbere niwo utuma umuntu aho unyuze hose niwo ukugaragaza ko ufite umwanda kuko iyo uhanyunze uba uhumura nabi bigatuma umuntu wese ahita akumenya kandi uwumeshe ukanakaraba ntabwo bakuvumbura.

Title: MUKARANGE GIRLS transcri.docx

Doc Creator: tcuhawenimana11

Doc Date: 3/25/2023

Codes Applied: Information that girls need

Linked Memos: 0

Excerpt Creator: tcuhawenimana11

Excerpt Created On: 4/29/2023

Excerpt Range: 15325-15601

Nimero yanjye ni 2 umwaka wa kabiri mfite imyaka 15, njye numva amakaru umukobwa ukigera mu bwangavu yakenera ari nko kumenya uko cotex ikoreshwa, akamenya uko yitwara igihe yageze mu mihango, akamenya ni isuku yo mu myanya yi ibanga ye no kumyenda ye yi imbere, nuko mbyumva.

Title: MUKARANGE GIRLS transcri.docx

Doc Creator: tcuhawenimana11

Doc Date: 3/25/2023

Codes Applied: Information that girls need

Linked Memos: 0

Excerpt Creator: tcuhawenimana11

Excerpt Created On: 4/29/2023

Excerpt Range: 15680-15796

Nanjye nihereyeho nyiri umwana nagiraga amatsiko yaho umwana aturuka rero mba numva nka ababyeyi baganiriza ku bana.

Title: MUKARANGE GIRLS transcri.docx

Doc Creator: tcuhawenimana11

Doc Date: 3/25/2023

Codes Applied: Information that girls need

Linked Memos: 0

Excerpt Creator: tcuhawenimana11

Excerpt Created On: 4/29/2023

Excerpt Range: 15828-15925

Amakuru mbifitewo njyewe nyine umukobwa yamenya uko yitwara akamenya ubuzima bwe ntasamare nyine.

Title: MUKARANGE GIRLS transcri.docx

Doc Creator: tcuhawenimana11

Doc Date: 3/25/2023

Codes Applied: Information that girls need

Linked Memos: 0

Excerpt Creator: tcuhawenimana11

Excerpt Created On: 4/29/2023

Excerpt Range: 16006-16034

Ndacyayakeneye kuko ndakiga.

Title: MUKARANGE GIRLS transcri.docx

Doc Creator: tcuhawenimana11

Doc Date: 3/25/2023

Codes Applied: Information that girls need

Linked Memos: 0

Excerpt Creator: tcuhawenimana11

Excerpt Created On: 4/29/2023

Excerpt Range: 16035-16118

Kugeza ubu ntabwo uzi aho umwana aca, uracyazi ko aca mu mukondo?

Oya, nzi aho aca.

Title: MUKARANGE GIRLS transcri.docx

Doc Creator: tcuhawenimana11

Doc Date: 3/25/2023

Codes Applied: Girls have much information about SRH

Linked Memos: 0

Excerpt Creator: tcuhawenimana11

Excerpt Created On: 4/29/2023

Excerpt Range: 16119-16399

Acahe?

Aca mu gitsina cy umugore.

Byagenze gute?

Igihe umugore aba yaratwise amezi 9 yashira akabyara.

Nawe watwita?

Yego.

Bigenze bite?

Kuko nyine nageze mu gihe cyo kujya mu mihango?

Bigenze gute?

Natwita aruko ntewe inda.

Baba bayiguteya mukihe gihe? Ayo makuru yo waba uyazi?

Title: MUKARANGE GIRLS transcri.docx

Doc Creator: tcuhawenimana11

Doc Date: 3/25/2023

Codes Applied: Received information is misleading and risky

Linked Memos: 0

Excerpt Creator: tcuhawenimana11

Excerpt Created On: 4/29/2023

Excerpt Range: 16441-16496

Mugihe hashize nki iminsi 8 cyangwa 9 mvuye mu mihango.

Title: MUKARANGE GIRLS transcri.docx

Doc Creator: tcuhawenimana11

Doc Date: 3/25/2023

Codes Applied: Information that girls need

Linked Memos: 0

Excerpt Creator: tcuhawenimana11

Excerpt Created On: 4/29/2023

Excerpt Range: 16497-16854

Murakoze, ndi nimero ya 3, niga muwa kabiri secondary mfite imyaka 16, njyewe ndumva ku bwanjye amakuru nakenera nageze nyine muri icyo gihe cyu ubwangavu amakuru ndikumva nakenera kwicara nkaganirizwa namenya uko nzitwara nkamenya nyine buri kimwe cyose nkamenya nyine uko nakwitwara nyine bakanganiriza bakambwira ko hanze hari ibishuko ngomba kubyirinda.

Title: MUKARANGE GIRLS transcri.docx

Doc Creator: tcuhawenimana11

Doc Date: 3/25/2023

Codes Applied: Precautions to take during puberty for girls

Linked Memos: 0

Excerpt Creator: tcuhawenimana11

Excerpt Created On: 4/29/2023

Excerpt Range: 17133-17535

Murakoze, ndi nimero 8, ubundi iyo umukobwa nyine yageze mu cyiciro cyu ubwangavu muri we, mu mubiri we yumva atangiye nkibyo ngibyo byo kuvugango batubuza ngo nki imibonano mpuzabitsina nyine ntabwo habura kumva agatima kabiteyeho ariko kuba uziko baba barakubwiye uti isaha ni saha iyariyo yose ushobora kubikoraho ushobora gutwara inda nabwo agatima gahita kagaruka uti oya ka ndeke ntazatwara inda.

Title: MUKARANGE GIRLS transcri.docx

Doc Creator: tcuhawenimana11

Doc Date: 3/25/2023

Codes Applied: Girls have much information about SRH

Linked Memos: 0

Excerpt Creator: tcuhawenimana11

Excerpt Created On: 4/29/2023

Excerpt Range: 17595-17645

Ashobora kubikora nyine ntatware inda ariko nyine,

Title: MUKARANGE GIRLS transcri.docx

Doc Creator: tcuhawenimana11

Doc Date: 3/25/2023

Codes Applied: Girls have much information about SRH

Linked Memos: 0

Excerpt Creator: tcuhawenimana11

Excerpt Created On: 4/29/2023

Excerpt Range: 17666-17709

Iyo wakoresheje agakingirizo ntabwo yasama.

Title: MUKARANGE GIRLS transcri.docx

Doc Creator: tcuhawenimana11

Doc Date: 3/25/2023

Codes Applied:

Linked Memos: 0

Excerpt Creator: tcuhawenimana11

Excerpt Created On: 4/29/2023

Excerpt Range: 17845-18296

Nyine iyo wumvise kwifata bikunaniye ntakindi ukoresha uretse agakingirizo ariko nabwo urabona icyiciro turimwo ntabwo aricyo kwifata ngo tugiye gutangire ngo turyamane ngo kuko twumvise nyine mumubiri twabishatse niyo wumvishije bikujeho, wumvishije nawe ati uwageragezaho, urabanza ukabwira umubyeyi ati se ma ko numva muri njyewe byanze agatima kari kuza ngo uwakora imibonano umubyeyi arakubwira ati oya ntabwo urageza igihe cyo gukora imibonano.

Title: MUKARANGE GIRLS transcri.docx

Doc Creator: tcuhawenimana11

Doc Date: 3/25/2023

Codes Applied: Types of information provided to boys and girls during puberty

Linked Memos: 0

Excerpt Creator: tcuhawenimana11

Excerpt Created On: 4/29/2023

Excerpt Range: 18408-18844

Oya ntabwo barambwirako bagerageje ahubwo babwirango natwe turabyifuza,natwe tugerageze twumve uko bimeze.

Nyine bambwirako nyine baba babyifuza ariko nyine ntabwo barabwirango barabikoze,ariko nyine nanjye iyo babimbwiye ngo reka tuzagerageze ndababwira nti oya njye mu rugo barambujije mube mwitonze kuko njye barambwiye ngo umuntu yemerewe gukora imibonano nu umugabo bashakanye bagasezerana ni imbere yi Imana, uko niko mbizi rero.

Title: MUKARANGE GIRLS transcri.docx

Doc Creator: tcuhawenimana11

Doc Date: 3/25/2023

Codes Applied: Information that boys need

Linked Memos: 0

Excerpt Creator: tcuhawenimana11

Excerpt Created On: 4/29/2023

Excerpt Range: 19103-19945

Murakoze,ndi nimero 1 nkaba mfite imyaka 14 nkaba niga mu mwaka wa kabiri, umuhungu ugeze mu gihe cyu ubugimbi akenewe kuganirizwa akareka gushuka umwana wu umukobwa kuko abahungu benshi nibo bashukana akagushuka akaza akakubwira ngo tugende turyamane wagenda mukaryamana nyine ugatwita nyine umuhungu amakuru yakenera ni ukumwigisha kugirango ka mbanze mbitekereze…

Murakoze, ndi nimero 2 mfite imyaka15 niga muwa kabiri njye numva amakuru umuhungu agomba kumenya,ari ukumenyako igihe yiroteyeho uko agomba kubyitwaramwo ko agomba no kubaza na abakuze akunguka ubundi bumenyi ikindi, akagomba kumenyako atagomba gufata ibiyobyambwenge niba yumva ko yakuze nki inzoga cyangwa inshinge akamenya nuko yitwara hagati simvuze ngo ntakavugane na abakobwa ariko baba incuti ariko akirinda nko kumukorakora ibyo nibyo bikurura imibonano mpuzabitsina

Title: MUKARANGE GIRLS transcri.docx

Doc Creator: tcuhawenimana11

Doc Date: 3/25/2023

Codes Applied: How girls experience changes occuring to them during puberty

Linked Memos: 0

Excerpt Creator: tcuhawenimana11

Excerpt Created On: 4/29/2023

Excerpt Range: 19946-20031

Aho bakunda kubakorakora ni nka he?

Nko kumukorakora ku mabere ku matako ahantu hose.

Title: MUKARANGE GIRLS transcri.docx

Doc Creator: tcuhawenimana11

Doc Date: 3/25/2023

Codes Applied: Pshcological changes occuring during puberty-Girls

Linked Memos: 0

Excerpt Creator: tcuhawenimana11

Excerpt Created On: 4/29/2023

Excerpt Range: 20093-20240

Njye sindabyumva ariko nk uko twabyize teacher yatubwiye ko iyo umukobwa akozwe ku ibere ahita yiyumvamo ubushake bwo gukora imibonano mpuzabitsina

Title: MUKARANGE GIRLS transcri.docx

Doc Creator: tcuhawenimana11

Doc Date: 3/25/2023

Codes Applied: Obstacles and risks experienced by girls during the sexual and reproductive health changes occuring during puberty and adolescence

Linked Memos: 0

Excerpt Creator: tcuhawenimana11

Excerpt Created On: 4/29/2023

Excerpt Range: 20362-21024

Hari igihe ubibwirwa nabo mungana cyangwa n abakuze.

Abakuze ubwo n ink abangana iki?

Hari n igihe uhura n abantu b abagabo baba papa bakajya bagushukisha utubombo

Abo ba papa se muhurira he muba muvuye ku ishuri cyangwa se

Hari igihe uhura n umuntu mukuru akakubwira ngo reka nguhe telephone tuzajye tuvugana ukabyemera ugasanga agiye akajya agushukashuka vayo unsure wajya wajya kumusura ugasanga byabaye uuh.

Hari uwo uzi byabayeho cyangwa nawe byaba byarakubayeho?

Njye ntabwo byambayeho nta nshuti yanjye nzi birabaho.

Nimero ni 6 umwaka ni uwa 2 imyaka ni 18 amakuru umuhungu yakenera harimo kumenya ukuntu yakwitwara mu gihe yageze mu gihe cy ubugimbi.

Title: MUKARANGE GIRLS transcri.docx

Doc Creator: tcuhawenimana11

Doc Date: 3/25/2023

Codes Applied: Information that boys need

Linked Memos: 0

Excerpt Creator: tcuhawenimana11

Excerpt Created On: 4/29/2023

Excerpt Range: 21101-21426

Amakuru yakenera iyo yageze muri icyo cyiciro hari ubwo aba yumva ashaka gukora imibonano mpuzabitsina cyane n igihe aganirizwa nyine ni ukamenya kuba yarinda igitsina cye no kuba nyine yakumva ko igihe cyose yakora imibonano mpuzabitsina ashobora gutera umukobwa inda akajya mu gihe cyo kuba kurera umwana kdi atarabiteguye.

Title: MUKARANGE GIRLS transcri.docx

Doc Creator: tcuhawenimana11

Doc Date: 3/25/2023

Codes Applied: Obstacles and risks experienced by girls during the sexual and reproductive health changes occuring during puberty and adolescence

Linked Memos: 0

Excerpt Creator: tcuhawenimana11

Excerpt Created On: 4/29/2023

Excerpt Range: 21475-21772

Iyo wenda nk iyo break iyo igeze umuhungu ikiganiro akunze kuganiraho cyane n umukobwa ni ikijyanye n imibinano mpuzabitsina akumvisha nyine uburyo ari byiza ubikoze nyine ukumva uburyo waryoherwa gusa nyine iyo udafite umutima wo kuba wahagarika ukuntu uba wiyumva muri ako kanya wahita ubikora.

Title: MUKARANGE GIRLS transcri.docx

Doc Creator: tcuhawenimana11

Doc Date: 3/25/2023

Codes Applied: Pshcological changes occuring during puberty-Girls Psychological changes occuring during puberty-Boys

Linked Memos: 0

Excerpt Creator: tcuhawenimana11

Excerpt Created On: 4/29/2023

Excerpt Range: 21842-21972

Yego hari ukundi umuntu abigenza nk umuntu ubufite kwikinisha iyo yumvise abishatse nyine mubwonko aribyo nyine birimo arikinisha.

Title: MUKARANGE GIRLS transcri.docx

Doc Creator: tcuhawenimana11

Doc Date: 3/25/2023

Codes Applied: Pshcological changes occuring during puberty-Girls Sexuality related practices girls do during puberty and adolescence

Linked Memos: 0

Excerpt Creator: tcuhawenimana11

Excerpt Created On: 4/29/2023

Excerpt Range: 22095-22295

Hari ukuntu wenda aba yararebye film izo twita porono mu bwonko bwe nyine yo agiye kwikinisha haza nibyo atekereza uko atekereza nyine birimo birakorwa yumva ari kumwe n umuntu we barimo barabikorana.

Title: MUKARANGE GIRLS transcri.docx

Doc Creator: tcuhawenimana11

Doc Date: 3/25/2023

Codes Applied: Types of information provided to boys and girls during puberty

Linked Memos: 0

Excerpt Creator: tcuhawenimana11

Excerpt Created On: 4/29/2023

Excerpt Range: 22520-22994

Amakuru nahawe ameza ni uko iyo ushatse gukora imibonano mpuzabitsina iyo uganirije wenda nk inshuti yawe akubwira ko icyaba cyiza ari uko wakwitwararika niba wanabishatse ukarindira ugashaka umugabo ukajya ubikora igihe ubishakiye. Hari ni ukubwira ko niba wenda udashoboye kwifata ufite kuba wagenda ukiteza inshinge ukaba wajya muri onapo cg ugakoresha agakingirizo mu gihe nyine unaniwe kwifata ukanjya nyine ukora imibonano mpuza bitsina nibura warakoresheje izo nzira.

Title: MUKARANGE GIRLS transcri.docx

Doc Creator: tcuhawenimana11

Doc Date: 3/25/2023

Codes Applied: Types of information provided to boys and girls during puberty

Linked Memos: 0

Excerpt Creator: tcuhawenimana11

Excerpt Created On: 4/29/2023

Excerpt Range: 23046-23254

Iyo witje inshinge iyo ukoze imibonano mpuzabitsina ntabwo utwita cyangwa iyo ufitemo nk agapira, bafite agapira k imyaka 3 cyagwa 5 ushobora kubikora igihe wumva ubishakiye kuko ntabwo ushobora kuba watwita.

Title: MUKARANGE GIRLS transcri.docx

Doc Creator: tcuhawenimana11

Doc Date: 3/25/2023

Codes Applied: Types of information provided to boys and girls during puberty

Linked Memos: 0

Excerpt Creator: tcuhawenimana11

Excerpt Created On: 4/29/2023

Excerpt Range: 23323-23405

Yego urazihabwa nta kibazo iyo wumva nyine ubishaka uragenda nyine ukazihabwa uuh.

Title: MUKARANGE GIRLS transcri.docx

Doc Creator: tcuhawenimana11

Doc Date: 3/25/2023

Codes Applied: Pshcological changes occuring during puberty-Girls

Linked Memos: 0

Excerpt Creator: tcuhawenimana11

Excerpt Created On: 4/29/2023

Excerpt Range: 23490-23930

Nimero yanjye ni 4 niga muri S1 mfite imyaka 16 nyine hari ukuntu nyine iyo wageze muri iyo myaka utangira nyine kumva mu mubiri ushyushye ukumva nyine icya gucyemurira ikibazo ari ukujya gukora imibonano mpuzabitsina,ariko nyine njye ukuntu mbitekereza ntabwo ari byiza nyine urabitekereza mu mutima wawe ukabyihererana wakumva nyine kwifata bikunaniye niko kujya kwiteza urwo rushing cyangwa ibinini n ibindi wakoresha kugira ngo udasama.

Title: MUKARANGE GIRLS transcri.docx

Doc Creator: tcuhawenimana11

Doc Date: 3/25/2023

Codes Applied: Precautions to take during puberty for girls

Linked Memos: 0

Excerpt Creator: tcuhawenimana11

Excerpt Created On: 4/29/2023

Excerpt Range: 23965-24290

Nyine imyaka turimo ntabwo ibitwemerera ariko nyine wenda njye mba numva nyine ubundi si byiza kuko ushobora kubikora ugatwita wend anta bushobozi mfite bwo kurera umwana cyangwa se uwayiguteye nawe cyobikoze nawe agira ngo yikinire akaba atagufasha, ariko nyine njyewe mba numva umuntu ubyemerewe aba ari umuntu wasezeranye.

Title: MUKARANGE GIRLS transcri.docx

Doc Creator: tcuhawenimana11

Doc Date: 3/25/2023

Codes Applied: Girls have much information about SRH

Linked Memos: 0

Excerpt Creator: tcuhawenimana11

Excerpt Created On: 4/29/2023

Excerpt Range: 24394-24935

Makumyabiri wenda n ibiri kuzamura njye niko mbitekereza.

Murakoze nimero yanjye ni 5 niga mu wa 2 imyaka ni 17 njye ndumva igihe wenda nko mu gihe cy ubwangavu Atari ngombwa nyine gukora imibonano mpuzabitsina wenda nyine igihe wumva kwifata bikunaniye ukaba wakoresha ako gakingirizo ariko nyine hari n abatagakoresha cyangwa se bitewe n uko aba ari ubwa mbere nyine akakwereka ko agakoresheje cyangwa se yamara nyine akaba yanagakuramo ukaba wenda twara iyo nda cyangwa se waba udatwaye iyo nda ukaba wakwandura n izo ndwara zitandukanye.

Title: MUKARANGE GIRLS transcri.docx

Doc Creator: tcuhawenimana11

Doc Date: 3/25/2023

Codes Applied: Girls have much information about SRH

Linked Memos: 0

Excerpt Creator: tcuhawenimana11

Excerpt Created On: 4/29/2023

Excerpt Range: 24936-24974

Nkizihe ndwara?

Sida imitezi n izindi.

Title: MUKARANGE GIRLS transcri.docx

Doc Creator: tcuhawenimana11

Doc Date: 3/25/2023

Codes Applied: Sources of information received during puberty

Linked Memos: 0

Excerpt Creator: tcuhawenimana11

Excerpt Created On: 4/29/2023

Excerpt Range: 25040-25462

Nyine njyewe bwa mbere ni mama wayampaye nkijya mu mihango atangira nyine kumbuza kwanika imwyeda y imbere mu nzu , ambwira ko nzajya nyanika hanze nyine ku zuba ko haba harimo microbe zigomba gupfa nyine ambwira uko nitwara nyine ambwira niba kwifata binaniye nakoresha ako gakingirizo cyangwa se ugasanga niba bikunaniye ukoreshe agakingirizo ariko nyine waba utagakoresheje ugasanga atwaye iyo nda ndetse ni izo ndwara.

Title: MUKARANGE GIRLS transcri.docx

Doc Creator: tcuhawenimana11

Doc Date: 3/25/2023

Codes Applied: Types of information provided to boys and girls during puberty

Linked Memos: 0

Excerpt Creator: tcuhawenimana11

Excerpt Created On: 4/29/2023

Excerpt Range: 25040-25462

Nyine njyewe bwa mbere ni mama wayampaye nkijya mu mihango atangira nyine kumbuza kwanika imwyeda y imbere mu nzu , ambwira ko nzajya nyanika hanze nyine ku zuba ko haba harimo microbe zigomba gupfa nyine ambwira uko nitwara nyine ambwira niba kwifata binaniye nakoresha ako gakingirizo cyangwa se ugasanga niba bikunaniye ukoreshe agakingirizo ariko nyine waba utagakoresheje ugasanga atwaye iyo nda ndetse ni izo ndwara.

Title: MUKARANGE GIRLS transcri.docx

Doc Creator: tcuhawenimana11

Doc Date: 3/25/2023

Codes Applied: Sources of information received during puberty

Linked Memos: 0

Excerpt Creator: tcuhawenimana11

Excerpt Created On: 4/29/2023

Excerpt Range: 25620-25657

Ni mama wayampaye

\ni inshuti yanjye

Title: MUKARANGE GIRLS transcri.docx

Doc Creator: tcuhawenimana11

Doc Date: 3/25/2023

Codes Applied: Sources of information received during puberty

Linked Memos: 0

Excerpt Creator: tcuhawenimana11

Excerpt Created On: 4/29/2023

Excerpt Range: 25678-25857

Ndi nimero 7 mfite imyaka 15 niga S2 twari mu isomo turimo kwiga ‘

Byari muisomo cyangwa hajemo n ikintu cy ikiganiro?

Twarabanje turiga hanyuma mu isomo mwarimu atugira mo inama

Title: MUKARANGE GIRLS transcri.docx

Doc Creator: tcuhawenimana11

Doc Date: 3/25/2023

Codes Applied: Types of information provided to boys and girls during puberty

Linked Memos: 0

Excerpt Creator: tcuhawenimana11

Excerpt Created On: 4/29/2023

Excerpt Range: 25886-26103

Aratubwira nyine ngo abakobwa iyo bageze mu gihe cy uwangavu baba nyine bashaka gukundana yareba umuhungu akumva aramukunze atubwira ntine ko tuzajya twirinda ibishuko, abahungu nyine badushuka n ibindi bigabo bikuru.

Title: MUKARANGE GIRLS transcri.docx

Doc Creator: tcuhawenimana11

Doc Date: 3/25/2023

Codes Applied: Sources of information received during puberty

Linked Memos: 0

Excerpt Creator: tcuhawenimana11

Excerpt Created On: 4/29/2023

Excerpt Range: 26104-26461

Abakobwa ntibashobora gushukana hagati yabo?

Yego barashukana

Urakoze ndi nimero 8 njyewe amakuru makeya nyine nayakuye kuri mama ayo afite ayandi nayakuye ku ishuri ubundi ayandi nayasomaga ku Ninyampinga.

Murakoze ndi nimero 1 makuru ni mama wayambwiye.

Amakuru mfite nayakuye ku mubyeyi wanjye ni mama wagiye ayambwira adi nyakura ku ishuri turi kwiga.

Title: MUKARANGE GIRLS transcri.docx

Doc Creator: tcuhawenimana11

Doc Date: 3/25/2023

Codes Applied: Sources of information received during puberty

Linked Memos: 0

Excerpt Creator: tcuhawenimana11

Excerpt Created On: 4/29/2023

Excerpt Range: 26519-26685

Ntabwo ahagije dukeneye n ayandi.

Murakoze ndi nimero 2 mfite imyaka 13 niga mu wa 2 ahantu nakuye amakuru nayakuye muri ba aunt banjye bavukana na mama no mu ishuri.

Title: MUKARANGE GIRLS transcri.docx

Doc Creator: tcuhawenimana11

Doc Date: 3/25/2023

Codes Applied: Sources of information received during puberty

Linked Memos: 0

Excerpt Creator: tcuhawenimana11

Excerpt Created On: 4/29/2023

Excerpt Range: 26715-26913

Ni bakuru ni aba mama

Murakoze nanjye ndi nimero ya 3 niga mu mwaka wa 2 mfite imyaka 16 njyewe amakuru mfite mbere na mbere nayakuye mu nzu y urubyiruko ya kabiri ni inshuti yayambwiye inshutikazi.

Title: MUKARANGE GIRLS transcri.docx

Doc Creator: tcuhawenimana11

Doc Date: 3/25/2023

Codes Applied: Sources of information received during puberty

Linked Memos: 0

Excerpt Creator: tcuhawenimana11

Excerpt Created On: 4/29/2023

Excerpt Range: 26914-27266

Mfite 18 njyewe amakuru yose y ingenzi nyine mama yaricaraga akabinganiriza byose andi nyine nkayakura ku ishuri rimwe na rimwe nkayumva nko mu bana b ishuti zanjye nkumva barimo barabivuga nyine hari ibyo nabaga ntazi nko guca imyeyo numvaga nyine ari nko kujya guca imyeyo nyine wenda yo gukubuza ariko nyine byose mama yarabinganirije nza kubimenya.

Title: MUKARANGE GIRLS transcri.docx

Doc Creator: tcuhawenimana11

Doc Date: 3/25/2023

Codes Applied: Obstacles and risks experienced by boys during the sexual and reproductive health changes occuring during puberty and adolescence

Linked Memos: 0

Excerpt Creator: tcuhawenimana11

Excerpt Created On: 4/29/2023

Excerpt Range: 27367-27614

Njyewe icyo numva nyine ashobora nko gutera umukobwa inda kandi ntabushobozi wenda yiga wenda umukobwa akaza akmujugunyira umwana, nta bushobozi bwo kumurera afite ugasanga nyine bagiye kumureresha wenda nyina w umuhungu nawe nta mbaraga yifitiye.

Title: MUKARANGE GIRLS transcri.docx

Doc Creator: tcuhawenimana11

Doc Date: 3/25/2023

Codes Applied: Obstacles and risks experienced by boys during the sexual and reproductive health changes occuring during puberty and adolescence

Linked Memos: 0

Excerpt Creator: tcuhawenimana11

Excerpt Created On: 4/29/2023

Excerpt Range: 27714-28659

Ingaruka nibo baba bazizi kubera ko nyine nibo bibaho. None se ubwo wajya kumubaza uko byagenze? Cyakora byo nyine abo nzi, hari nkiyo aba yateye nk umukobwa inda yakwanga kumufasha umukobwa nawe akaza akamujugunya iwabo w umuhungu.

Murakoze ndi nimero 8 ingaruka ziba ku muhungu w ingimbi ashobora nyine gushukwa n umubiri akabwir umukobwa ati turyamane nta gakingirizo ubundi abahungu benshi uko numva bavuga ngo nta kurira bombo mw ishashi nyine bakaryamana nta gakingirizo ugasanga ateye umukobwa inda. \Natera uwo mukobwa inda iwabo w umukobwa nibabibona ntabwo bazaceceka bazamubwira ati ntabwo twabyemera uwo muhungu ni ukumufungisha kandi ari umuhungu ari umukobwa bose biraba bitumye nyine niba bari abanyeshuri amashuri yabo nyine ahagarikirwa aho naho umuhungu ashobora gufungwa kuko nyine yakinishje kuryamana n umukobwa w abandi kandi akanamutera inda kandi na wa mukobwa nyine bikamusigira ingaruka zo kuba mama kandi akiri umwana.

Title: MUKARANGE GIRLS transcri.docx

Doc Creator: tcuhawenimana11

Doc Date: 3/25/2023

Codes Applied: Obstacles and risks experienced by girls during the sexual and reproductive health changes occuring during puberty and adolescence

Linked Memos: 0

Excerpt Creator: tcuhawenimana11

Excerpt Created On: 4/29/2023

Excerpt Range: 28751-29163

Amashuri ye ntabwo ayakomeza kandi nyine no mu mudugudu abamubona ngo do dore wa mukobwa w ikirara nyine avugwa ibintu bitari byiza, nabyo nyine bimukomeretsa nyine nawe akabaho ubuzima butoroshye.

Ndi nimero kabiri ibyago ashobora guhura nabyo ku mubiri harimo nkindwara ya fisitile ituma umuntu ahora anuka nabi ashobora no gihinduka mu miterere ye niba yari afite mu nda zeru agahita inda nini yii nta bindi.

Title: MUKARANGE GIRLS transcri.docx

Doc Creator: tcuhawenimana11

Doc Date: 3/25/2023

Codes Applied: Barriers leading to the risks and obstacles girls and boys experience during puberty and adolescence

Linked Memos: 0

Excerpt Creator: tcuhawenimana11

Excerpt Created On: 4/29/2023

Excerpt Range: 29164-29982

Ni izihe nzitiza ingimbi n abangavu bahura nazo zituma batamenya kwitwara uko bikwiye mu bijyanye n ubuzima bw imyororokere n imihindagurikire yo mu gihe cy ubugimbi n ubwangavu?

Ashobora kuba ataraganirijwe cyangwa nta makuru ahagije afite cyangwa se wenda ashaka nko gukora imibonano mpuzabitsina agahura n umukobwa we atabishaka.

Murakoze ndi nimero rimwe nyine iyo umuhungu yiroteyeho akagenda akabisobanurira ababyeyi be bakamusobanurira bakamubwira ngo urakuze nawe yumva ko yakuze yabaye umugabo akagenda agatera umukobwa inda yarangiza yamara kuyimutera akamwihakana umukobwa yagenda yabibwira iwabo , iwabo bakamwirukana akagenda yasanga umuhungu, umuhungu nawe akamwihakana ngo nagende ashakeumuntu wamuteye inda nyine uwo muhungu akamwihakana , umukobwa yamwumvisha ko ariwe wayimuteyw umuhungu ntabyumve

Title: MUKARANGE GIRLS transcri.docx

Doc Creator: tcuhawenimana11

Doc Date: 3/25/2023

Codes Applied: Obstacles and risks experienced by girls during the sexual and reproductive health changes occuring during puberty and adolescence

Linked Memos: 0

Excerpt Creator: tcuhawenimana11

Excerpt Created On: 4/29/2023

Excerpt Range: 30165-30350

Murakoze nimero ni 6 inzitizi umukobwa nyine agira ni ukuba umuhungu amubwiye ko amukunda no kuba nyine igihe cyose umuhungu nyine iyo baganiriye amubwira gukora imibonano mpuzabitsina.

Title: MUKARANGE GIRLS transcri.docx

Doc Creator: tcuhawenimana11

Doc Date: 3/25/2023

Codes Applied: Obstacles and risks experienced by girls during the sexual and reproductive health changes occuring during puberty and adolescence

Linked Memos: 0

Excerpt Creator: tcuhawenimana11

Excerpt Created On: 4/29/2023

Excerpt Range: 30351-30550

Ubundi se umuntu akubwiye ngo aragukunda hari icyo byaba bitwaye?

Ntacyo byaba bitwaye gusa amagambo uvugana hagati yanyu niyo aganisha ku kuryamana.

Aganisha kukuryamana niyo nzitizi yonyine ihari?

Title: MUKARANGE GIRLS transcri.docx

Doc Creator: tcuhawenimana11

Doc Date: 3/25/2023

Codes Applied: Obstacles and risks experienced by girls during the sexual and reproductive health changes occuring during puberty and adolescence

Linked Memos: 0

Excerpt Creator: tcuhawenimana11

Excerpt Created On: 4/29/2023

Excerpt Range: 30636-30831

Murakoze, nimero 7 indi nzitia nyine n ink uko uba wagiye mu mihango bigatuma nyine bose ntago baba bameze kimwe hari abajyamo ukabona mu maso nyine bijimye bakumva nyine ibintu byabarangiriyeho.

Title: MUKARANGE GIRLS transcri.docx

Doc Creator: tcuhawenimana11

Doc Date: 3/25/2023

Codes Applied: Sexuality related practices girls do during puberty and adolescence

Linked Memos: 0

Excerpt Creator: tcuhawenimana11

Excerpt Created On: 4/29/2023

Excerpt Range: 31001-31291

Yee, ndi nimero 8 njye ikibazo nyine nashakaga kubaza nkuriya wavuze ibintu byo guca imyeyo ese ko bamwe ko njya numva ngo ni imihango ya kera koko se ni imihango yak era cyangwa se ni ibintu nyine n uko nyine n ubundi n abi iki gihe babikora cyangwa se ubundi guca iyo myeyo bigufasha iki?

Title: MUKARANGE GIRLS transcri.docx

Doc Creator: tcuhawenimana11

Doc Date: 3/25/2023

Codes Applied: Sexuality related practices girls do during puberty and adolescence

Linked Memos: 0

Excerpt Creator: tcuhawenimana11

Excerpt Created On: 4/29/2023

Excerpt Range: 31822-31967

None se kuyica bifasha iki umuntu kandi baravuze ngo iyo utayiciye umugabo arakwirukana kandi ngo iyo ugiye kubyara utarayiciye birakugora nibyo?

Title: G S Kayonza Girls transc.docx

Doc Creator: tcuhawenimana11

Doc Date: 3/25/2023

Codes Applied: Physiological changes occuring during puberty-Boys

Linked Memos: 0

Excerpt Creator: tcuhawenimana11

Excerpt Created On: 4/27/2023

Excerpt Range: 113-335

Nomero yange ni 4 mfite imyaka 16 nkaba niga mu mwaka wa 3. Impinduka 2 nizo nabasha kuvuga zibaho; aniga ijwi akanazana ubwanwa nizindi nce z’umubiri agatangira kuzana ubwoya. Hehe zivuge? Mu kwaha no ku gitsina murakoze!

Title: G S Kayonza Girls transc.docx

Doc Creator: tcuhawenimana11

Doc Date: 3/25/2023

Codes Applied: Physiological changes occuring during puberty-Boys

Linked Memos: 0

Excerpt Creator: tcuhawenimana11

Excerpt Created On: 4/27/2023

Excerpt Range: 394-534

Ikintu nzi ku mwana wumuhungu ugeze mu myaka yuburumbuke atangira kwiroteraho ikindi nzi nyine nubwoba buza ku gitsina no mu kwaha murakoze!

Title: G S Kayonza Girls transc.docx

Doc Creator: tcuhawenimana11

Doc Date: 3/25/2023

Codes Applied: Boys and girls know about the causes leading to the physiological changes during puberty

Linked Memos: 0

Excerpt Creator: tcuhawenimana11

Excerpt Created On: 4/27/2023

Excerpt Range: 724-754

Biterwa nimyaka baba bagezemo.

Title: G S Kayonza Girls transc.docx

Doc Creator: tcuhawenimana11

Doc Date: 3/25/2023

Codes Applied: Physiological changes occuring during puberty-Boys

Linked Memos: 0

Excerpt Creator: tcuhawenimana11

Excerpt Created On: 4/27/2023

Excerpt Range: 755-964

Urugero iyo ugeje imyaka 12 nibwo umuhungu atangira kwiroteraho agatangira nyine kuzana utuntu twutwoya akumva yakuze nge niko mbyumva ariko mba numva ariko biri kubera ko nange byambayeho mfite iyo myaka 12.

Title: G S Kayonza Girls transc.docx

Doc Creator: tcuhawenimana11

Doc Date: 3/25/2023

Codes Applied: Boys and girls know about the causes leading to the physiological changes during puberty

Linked Memos: 0

Excerpt Creator: tcuhawenimana11

Excerpt Created On: 4/27/2023

Excerpt Range: 1139-1219

Nyine biterwa n’imyaka kuva kuri 12 nibwo atangira kugira izo mpinduka kuzamura.

Title: G S Kayonza Girls transc.docx

Doc Creator: tcuhawenimana11

Doc Date: 3/25/2023

Codes Applied: Boys and girls know about the causes leading to the physiological changes during puberty

Linked Memos: 0

Excerpt Creator: tcuhawenimana11

Excerpt Created On: 4/27/2023

Excerpt Range: 1329-1453

Ndumva izo mpinduka akenshi na kenshi ziterwa nimisemburo yumuntu ako bikongera bigaterwa nanone nimyaka aba afite murakoze!

Title: G S Kayonza Girls transc.docx

Doc Creator: tcuhawenimana11

Doc Date: 3/25/2023

Codes Applied: Boys and girls know about the causes leading to the physiological changes during puberty

Linked Memos: 0

Excerpt Creator: tcuhawenimana11

Excerpt Created On: 4/27/2023

Excerpt Range: 1577-2094

Ukundi kuntu mbyumva biterwa no kuba warariye neza, nabyo bituma umuntu akura neza ukambona ukavuga ati uyu mwana afite imyaka nka 30 pe cyangwa ukamureba ukavuga ati ko mbona ameze nkagakecuru. Ukavuga ati uyu mwana afite imyaka myinshi yarashaje kubera ibibazo byimiryango. Rimwe na rimwe biterwa nibibazo by’imiryango urugero nko kubura umubyeyi wawe wakundaga. Ibaze ufite papa wawe waramukundaga agapfa uzi ukuntu bibabaza? Nyine ukavuga ati no kwiga ndabiretse ukitera icyizere kandi imbere ariho heza murakoze.

Title: G S Kayonza Girls transc.docx

Doc Creator: tcuhawenimana11

Doc Date: 3/25/2023

Codes Applied: Physiological changes occuring during puberty-Girls

Linked Memos: 0

Excerpt Creator: tcuhawenimana11

Excerpt Created On: 4/27/2023

Excerpt Range: 2327-2373

Nukujya mu mihango no kumera amabere murakoze.

Title: G S Kayonza Girls transc.docx

Doc Creator: tcuhawenimana11

Doc Date: 3/25/2023

Codes Applied: Physiological changes occuring during puberty-Girls

Linked Memos: 0

Excerpt Creator: tcuhawenimana11

Excerpt Created On: 4/27/2023

Excerpt Range: 2513-2568

Impinduka ziba kumukobwa azana incakwaha akazana ninsya

Title: G S Kayonza Girls transc.docx

Doc Creator: tcuhawenimana11

Doc Date: 3/25/2023

Codes Applied: Physiological changes occuring during puberty-Girls

Linked Memos: 0

Excerpt Creator: tcuhawenimana11

Excerpt Created On: 4/27/2023

Excerpt Range: 2749-2781

Atangira no gukura ahantu hose.

Title: G S Kayonza Girls transc.docx

Doc Creator: tcuhawenimana11

Doc Date: 3/25/2023

Codes Applied: Pshcological changes occuring during puberty-Girls

Linked Memos: 0

Excerpt Creator: tcuhawenimana11

Excerpt Created On: 4/27/2023

Excerpt Range: 2840-3006

Impinduka nzi ku mwana w’umukobwa iyo amaze kugera mu gihe cyubukuru atangira nyine kumva yashyushye mu mubiri we akumva ashaka gukora imibonanompuzabitsina murakoze.

Title: G S Kayonza Girls transc.docx

Doc Creator: tcuhawenimana11

Doc Date: 3/25/2023

Codes Applied: Pshcological changes occuring during puberty-Girls

Linked Memos: 0

Excerpt Creator: tcuhawenimana11

Excerpt Created On: 4/27/2023

Excerpt Range: 3069-3194

impinduka akenshi ziba ku mukobwa iyo yageze mu gihe cyo gukura atangira guhindura imyitwarire akajya agira nisuku murakoze.

Title: G S Kayonza Girls transc.docx

Doc Creator: tcuhawenimana11

Doc Date: 3/25/2023

Codes Applied: Physiological changes occuring during puberty-Girls

Linked Memos: 0

Excerpt Creator: tcuhawenimana11

Excerpt Created On: 4/27/2023

Excerpt Range: 3282-3351

Impinduka ziba kumukobwa mu gihe cyubwangavu atangira kuzana amatayi

Title: G S Kayonza Girls transc.docx

Doc Creator: tcuhawenimana11

Doc Date: 3/25/2023

Codes Applied: Pshcological changes occuring during puberty-Girls

Linked Memos: 0

Excerpt Creator: tcuhawenimana11

Excerpt Created On: 4/27/2023

Excerpt Range: 3354-3379

kwiyitaho cyane murakoze.

Title: G S Kayonza Girls transc.docx

Doc Creator: tcuhawenimana11

Doc Date: 3/25/2023

Codes Applied: Pshcological changes occuring during puberty-Girls

Linked Memos: 0

Excerpt Creator: tcuhawenimana11

Excerpt Created On: 4/27/2023

Excerpt Range: 3452-3736

nyine umukobwa mu gihe ageze mu gihe cy’ubwangavu atangira gutekereza cyane biri fast, akagira ubwenge kuburyo ushobora kuba ufite ikibazo ntukibwire mama wawe, ntukibwire papa wawe gusa ukumva yuko ari ibyawe gusa, ukagira isuku ihambaye kuburyo ibintu byawe byose ubikora vuba vuba.

Title: G S Kayonza Girls transc.docx

Doc Creator: tcuhawenimana11

Doc Date: 3/25/2023

Codes Applied: Physiological changes occuring during puberty-Girls

Linked Memos: 0

Excerpt Creator: tcuhawenimana11

Excerpt Created On: 4/27/2023

Excerpt Range: 3737-3883

Ngewe nyine urugero njya mu mihango bwa mbere ntawe nabibwiye numwe narabyikoreye ngenyine nyine ugira isuku nange niko navuga, isuku iba ikabije.

Title: G S Kayonza Girls transc.docx

Doc Creator: tcuhawenimana11

Doc Date: 3/25/2023

Codes Applied: Boys and girls know about the causes leading to the physiological changes during puberty

Linked Memos: 0

Excerpt Creator: tcuhawenimana11

Excerpt Created On: 4/27/2023

Excerpt Range: 4003-4058

Ziterwa n’imyaka cyangwa imisemburo y’umuntu murakoze.

Title: G S Kayonza Girls transc.docx

Doc Creator: tcuhawenimana11

Doc Date: 3/25/2023

Codes Applied: Boys and girls know about the causes leading to the physiological changes during puberty

Linked Memos: 0

Excerpt Creator: tcuhawenimana11

Excerpt Created On: 4/27/2023

Excerpt Range: 4107-4163

Izo mpinduka ziterwa nimisemburo cyangwa imyaka agezemo.

Title: G S Kayonza Girls transc.docx

Doc Creator: tcuhawenimana11

Doc Date: 3/25/2023

Codes Applied: Boys and girls know about the causes leading to the physiological changes during puberty

Linked Memos: 0

Excerpt Creator: tcuhawenimana11

Excerpt Created On: 4/27/2023

Excerpt Range: 4164-4283

Ndi nimero 4 mfite imyaka 16 nkaba niga muwa 3 nkaba numva biterwa nimyaka, imisemburo, imterere cyangwa imitekerereze.

Title: G S Kayonza Girls transc.docx

Doc Creator: tcuhawenimana11

Doc Date: 3/25/2023

Codes Applied: Boys and girls know about the causes leading to the physiological changes during puberty

Linked Memos: 0

Excerpt Creator: tcuhawenimana11

Excerpt Created On: 4/27/2023

Excerpt Range: 4361-4474

nkaba numva biterwa nukuntu umuntu aba atangiye kwiyumva ko yakuze, agatangira no guhindura ibitekerezo murakoze.

Title: G S Kayonza Girls transc.docx

Doc Creator: tcuhawenimana11

Doc Date: 3/25/2023

Codes Applied: How girls experience changes occuring to them during puberty

Linked Memos: 0

Excerpt Creator: tcuhawenimana11

Excerpt Created On: 4/27/2023

Excerpt Range: 4649-4836

Ndi nimero 5 mfite imyaka 16 nkaba niga mu mwaka wa 2 wamashuri yisumbuye. Impinduka zambayeho nyine nange njya mu mihango byarantunguye, nabimbwiye ababyeyi bange babimfashamo murakoze.

Title: G S Kayonza Girls transc.docx

Doc Creator: tcuhawenimana11

Doc Date: 3/25/2023

Codes Applied: How girls experience changes occuring to them during puberty

Linked Memos: 0

Excerpt Creator: tcuhawenimana11

Excerpt Created On: 4/27/2023

Excerpt Range: 4879-4984

Impnduka zambayeho nukumera amabere. Byarantunguye numva nakuze. Ntawe wabibwiye yaba mama cyangwa papa?

Title: G S Kayonza Girls transc.docx

Doc Creator: tcuhawenimana11

Doc Date: 3/25/2023

Codes Applied: How girls experience changes occuring to them during puberty

Linked Memos: 0

Excerpt Creator: tcuhawenimana11

Excerpt Created On: 4/27/2023

Excerpt Range: 5063-5236

Nyine ngewe nkijya mu mihango ibyambayeho nameze amabere nincakwaha nibindi. Wabyitwayemo ute? Nyine byarantunguye mbaza kwibaza ibyaribyo mbibwira ababyeyi baransobanurira.

Title: G S Kayonza Girls transc.docx

Doc Creator: tcuhawenimana11

Doc Date: 3/25/2023

Codes Applied: How girls experience changes occuring to them during puberty

Linked Memos: 0

Excerpt Creator: tcuhawenimana11

Excerpt Created On: 4/27/2023

Excerpt Range: 5237-5701

Wabiboneye kimwe byose se? oya. Icyo wabonye bwa mbere niki cyagutunguye? Namabere. Wabyifashemo ute? Nyine nahise numva nange nkuze. Wabibwiwe niki se ko iyo umuntu abonye amabere aba yakuze? Ntahandi wari warabibonye? Ntamakuru warufite? Byaragutunguye gusa? Oya, nyine nari narabibonye. Imihango ukiyibona nibwo bwa mbere warukiyumva? Ababyeyi bari barabinganirijeho. Kwishuri ntabwo wari warabyumvishije muri bagenzi bawe ahandi hantu hose? Naho narabyumvishe.

Title: G S Kayonza Girls transc.docx

Doc Creator: tcuhawenimana11

Doc Date: 3/25/2023

Codes Applied: How girls experience changes occuring to them during puberty

Linked Memos: 0

Excerpt Creator: tcuhawenimana11

Excerpt Created On: 4/27/2023

Excerpt Range: 5853-5972

Bwa mbere ubundi nabanje kumera amabere, bwa kabiri ngiye kubona mbona ngiye mu mihango mbibwira ababyeyi barabimfasha.

Title: G S Kayonza Girls transc.docx

Doc Creator: tcuhawenimana11

Doc Date: 3/25/2023

Codes Applied: How girls experience changes occuring to them during puberty

Linked Memos: 0

Excerpt Creator: tcuhawenimana11

Excerpt Created On: 4/27/2023

Excerpt Range: 6044-6712

Bwa mbere nange nabanje kumera amabere umuntu agatangira kugira isoni no gutangira gushaka kuyahisha ako nyine biranga nubundi akagaragara, bwa kabiri nyine mera incakwaha njya no mu mihango ariko narinsanzwe mbizi nanabyumvana bagenzi bange nyine mfite ninshuti bibaho nyine nange mbaza kugira isoni zo kubibwira mu rugo, mbwibwira mukuru wange nyine aramfasha atangira kunyereka ibikoresho bakoresha bimwe na bimwe ntarinzi nangira kumenya uko babikoresha nyine ambwira ko nzongera isuku kurusha iyo narimfite kuko iyo wageze mu gihe nyine cyubukuru ntukajye wikorera isuku nyine wananuka wabangamira abantu. Nyine ibyo byose bimbayeho na mama bimugeraho murakoze.

Title: G S Kayonza Girls transc.docx

Doc Creator: tcuhawenimana11

Doc Date: 3/25/2023

Codes Applied: How to take care of themselves when phsyiological changes occur for girls

Linked Memos: 0

Excerpt Creator: tcuhawenimana11

Excerpt Created On: 4/27/2023

Excerpt Range: 6368-6712

mbwibwira mukuru wange nyine aramfasha atangira kunyereka ibikoresho bakoresha bimwe na bimwe ntarinzi nangira kumenya uko babikoresha nyine ambwira ko nzongera isuku kurusha iyo narimfite kuko iyo wageze mu gihe nyine cyubukuru ntukajye wikorera isuku nyine wananuka wabangamira abantu. Nyine ibyo byose bimbayeho na mama bimugeraho murakoze.

Title: G S Kayonza Girls transc.docx

Doc Creator: tcuhawenimana11

Doc Date: 3/25/2023

Codes Applied: How girls experience changes occuring to them during puberty

Linked Memos: 0

Excerpt Creator: tcuhawenimana11

Excerpt Created On: 4/27/2023

Excerpt Range: 6800-7184

Nange nabanje kumera amabere najyaga nibaza ngo amabere ava kuri iyi stage akajya ku yindi, nyine nyameze ndayahisha nakundaga gukina umupira nkumva arambangamiye nkajya nyahisha cyane nyine bigatuma nambara nibi jumper binini kuko yaje niga senior 1 nkumva ntashaka kuyamera nyine noneho nyine birangora aba Manini ndakwambara ishati nyine abantu bakambwira ngo wabubuse ngo twaguye

Title: G S Kayonza Girls transc.docx

Doc Creator: tcuhawenimana11

Doc Date: 3/25/2023

Codes Applied: How to take care of themselves when phsyiological changes occur for girls

Linked Memos: 0

Excerpt Creator: tcuhawenimana11

Excerpt Created On: 4/27/2023

Excerpt Range: 7184-7524

ngo nshake agasutiye bintera isoni kubwira mama ko nshaka agasutiye nyine mbibwira umunyeshuri twiganaga arambwira ngo azakampa ndamubwira ngo sinakageza mu rugo mama ngiye kubona arakazanye kubera ko nawe yari yarabibonye arambwira ngo akira ujye wambara agasutiye nyine kayafate bintera isoni ariko nyine ndakambara ubu ntakintu bintwaye.

Title: G S Kayonza Girls transc.docx

Doc Creator: tcuhawenimana11

Doc Date: 3/25/2023

Codes Applied: How to take care of themselves when phsyiological changes occur for girls

Linked Memos: 0

Excerpt Creator: tcuhawenimana11

Excerpt Created On: 4/27/2023

Excerpt Range: 7525-8114

Ubwa kabiri njya mu mihango byo noneho ndatinya ndarira kuko nari natinze kuyijyamo nayijyiyemo niga s3 bintera ubwo ariko kubera ko bazaga kutuganiriza bakavuga ngo iyo utagiye mu mihango uba ufite ikibazo hanyuma ije nkajya ndira ako nkavuga ngo narimfite ikibazo ako ubwo ije nyine ni byiza bintera isoni kubwira mama nyine ko nayigiyemo nabakuru bange biga babamo mbwira nyine inshuti zange banjyana mu cyumba cyabakobwa banyereka uburyo bakoresha pad nyine ndagenda njya muri mari ya mukuru wange nkuramo ama pad nkajya nambara, izo nizo mpinduka zambayeho nyine ziranangora murakoze.

Title: G S Kayonza Girls transc.docx

Doc Creator: tcuhawenimana11

Doc Date: 3/25/2023

Codes Applied: How to take care of themselves when phsyiological changes occur for girls How girls experience changes occuring to them during puberty

Linked Memos: 0

Excerpt Creator: tcuhawenimana11

Excerpt Created On: 4/27/2023

Excerpt Range: 8114-8568

impamvu nyine byanteraga isoni kugira amabere najyaga mbona abakobwa twakinanaga umupira bafite amabere nkabona birabagora kwirukanka mu kibuga nkavuga ni noneho nange ngiye kureka gukina ako nyine ntago yabaye Manini nubu nakina. Imihango yo narayitinyaga hari abanyeshuri bajyaga batwicara imbera bagahaguruka tukabona nko kuma desk tukabona amaraso atakomeretse tukabitinya tukavuga ngo ibintu ntago byatubaho nyine bimbayeho ndavuga ngo kabaye nyine.

Title: G S Kayonza Girls transc.docx

Doc Creator: tcuhawenimana11

Doc Date: 3/25/2023

Codes Applied: How girls experience changes occuring to them during puberty

Linked Memos: 0

Excerpt Creator: tcuhawenimana11

Excerpt Created On: 4/27/2023

Excerpt Range: 8747-9512

Nayigiyemo ejobundi niga s3, nta muntu nabibwiye narabigumanye ndabiceceka nkazajya nkurikiza murumuna wange niwe wabinyigishije kandi nawe atabizi akanyigisha uburyo Bambara pad nkazajya nyambara ariko njya kurwara bwa mbere narwaye munda cyane kuburyo no kuryama byari byarananiye. Naryamaga nubitse inda munda hakandya nkarira gusa nagiye ngira inama za bakuru bange bigeraho birarangira noneho njya kumera amabere yarandiye cyane, icyo gihe nayameze niga p6 nakoraho ukagirango ni nkibishirira ukabura uburyo uryama noneho ibaze wiga p6 ufite gukora ikizamini cya leta uri kumera amabere kuryama bikakunanira ukarara umeze gutya wakoraho ukababara cyane ako nyine nkihangana gusa nyine abanhungu badukunda ariko twebwe tukabanga tukababwira ngo mutureke twige.

Title: G S Kayonza Girls transc.docx

Doc Creator: tcuhawenimana11

Doc Date: 3/25/2023

Codes Applied: How girls experience changes occuring to them during puberty

Linked Memos: 0

Excerpt Creator: tcuhawenimana11

Excerpt Created On: 4/27/2023

Excerpt Range: 9553-9915

Ngewe impinduka zambayeho nagiye mu mihango hanyuma mbibwira mama ambwira ukuntu bakoresha pad hanyuma ndabimenya murakoze. Ntago byaguteye ubwoba? Nge ntabwo byaneye ubwoba nabibonye ari kumanywa. Waruri kwishuri? Oya narindi mu rugo. Nge ntabwo byanteye ubwoba kuko nyine inshuti zange zarabimbwiraga byaje kuza ngewe narabimenye ntabwo byigeze bintera ubwoba.

Title: G S Kayonza Girls transc.docx

Doc Creator: tcuhawenimana11

Doc Date: 3/25/2023

Codes Applied: How to take care of themselves when phsyiological changes occur for girls

Linked Memos: 0

Excerpt Creator: tcuhawenimana11

Excerpt Created On: 4/27/2023

Excerpt Range: 9992-10219

Ngewe impinduka zambayeho nameze amabere nkajya nyahisha cyane nkatinya no kubibwira mama, nkajya ntinya kuburyo ntatashakaga numuntu umvugisha. Nizo mpinduka zangezeho zonyine. Ntazindi zabaye se? ntazindi. Nta mihango? Yego.

Title: G S Kayonza Girls transc.docx

Doc Creator: tcuhawenimana11

Doc Date: 3/25/2023

Codes Applied: How girls experience changes occuring to them during puberty

Linked Memos: 0

Excerpt Creator: tcuhawenimana11

Excerpt Created On: 4/27/2023

Excerpt Range: 10302-10706

Impinduka zambayeho nabanje kumera amabere nange nakundaga gukina umupira nkumva azambangamira, hanyuma bwa kabiri njya mu mihango ako nyine numvaga inshuti zange zibivuga nge ntarayijyamo nange nyigiyemo ejobundi numvaga inshuti zange zibivuga cyane ariko nyine nyigezemo mbwibwira mama abimfashamo ako nyine nange nabanje gutinya. Warutarabyumvaho? Nari narabyumviseho ku nshuti zange na mama abimbwira.

Title: G S Kayonza Girls transc.docx

Doc Creator: tcuhawenimana11

Doc Date: 3/25/2023

Codes Applied: How girls experience changes occuring to them during puberty

Linked Memos: 0

Excerpt Creator: tcuhawenimana11

Excerpt Created On: 4/27/2023

Excerpt Range: 10735-10860

Kumera ninshya namabere nubucakwaha. Mu mitekerereze se? uba wumva nyine ushaka koga buri mwanya, ugira isuku nyinshi nibyo.

Title: G S Kayonza Girls transc.docx

Doc Creator: tcuhawenimana11

Doc Date: 3/25/2023

Codes Applied: Information that girls need

Linked Memos: 0

Excerpt Creator: tcuhawenimana11

Excerpt Created On: 4/27/2023

Excerpt Range: 11200-11693

Ndumva amakuru umwana wumukobwa akenye ugeze mu gihe cyubwangavu aruguhuhabwa amahugurwa bakamwigisha uburyo agomba kwirinda gukora imibonanompuzabitsina idakingiye kuko nyine ashobora gukuramo indwara cyangwa ninda zitateganyijwe kandi nyine akabwirwa kwikorera isuku no kwirinda ibishuko nyine biri hanze kuko aba atangiye kugera mu gihe cyo kumva ashaka gukundana kandi hari byinshi ashobora guhura nabyo mugukundana nkibishuko byagushora mubusambanyi ugatwara inda zitateganyijwe murakoze.

Title: G S Kayonza Girls transc.docx

Doc Creator: tcuhawenimana11

Doc Date: 3/25/2023

Codes Applied: Information that girls need

Linked Memos: 0

Excerpt Creator: tcuhawenimana11

Excerpt Created On: 4/27/2023

Excerpt Range: 11825-12067

Amakuru nakenera ninko kumbwira uburyo iyo myaka ngezemo nayitwaramo ukanambwira aho bigomba ko nakorera iki ntaho ntagikorera ukanambwira ibikoresho nakenera ndi murubwo bwangavu aho nabikura nuburyo nabikoresha nuburyo nabinyuzamo murakoze.

Title: G S Kayonza Girls transc.docx

Doc Creator: tcuhawenimana11

Doc Date: 3/25/2023

Codes Applied: Girls have much information about SRH

Linked Memos: 0

Excerpt Creator: tcuhawenimana11

Excerpt Created On: 4/27/2023

Excerpt Range: 12129-12191

Igihe umwangavu yagiye mu mihango biba byashoboka ko yatwita.

Title: G S Kayonza Girls transc.docx

Doc Creator: tcuhawenimana11

Doc Date: 3/25/2023

Codes Applied: Received information is misleading and risky

Linked Memos: 0

Excerpt Creator: tcuhawenimana11

Excerpt Created On: 4/27/2023

Excerpt Range: 12303-12776

Uko nyine nabyize nuko mbyibuka batubwiye ko umuntu ashobora kudatwita mu minsi 4 ya nyuma yimihango ako iyo utarajya mu mihango nyine mbere yiminsi 4 ushobora gutwara inda kandi nyine utarabona nimihango, banatubwiye ko ushobora kuba uri mu mihango kandi ugatwita. Twe twari tuzi ko nyine bitakunda ko umuntu aba mu mihango agatwita ariko nyine batubwiye ko umuntu ashobora gukora imibonanompuzabitsina ari mu gihe cyimihango agasamiramo inda nubwo nyine bidakunze kubaho.

Title: G S Kayonza Girls transc.docx

Doc Creator: tcuhawenimana11

Doc Date: 3/25/2023

Codes Applied: Received information is right and provides sufficient information helping boys and girls during puberty

Linked Memos: 0

Excerpt Creator: tcuhawenimana11

Excerpt Created On: 4/27/2023

Excerpt Range: 13037-13154

Igihe umuntu ashobora gusama ni igihe yakoze imibonano idakingiye cyangwa se akanduriramo nibindi bintu bitandukanye.

Title: G S Kayonza Girls transc.docx

Doc Creator: tcuhawenimana11

Doc Date: 3/25/2023

Codes Applied: Received information is misleading and risky

Linked Memos: 0

Excerpt Creator: tcuhawenimana11

Excerpt Created On: 4/27/2023

Excerpt Range: 13182-13239

Title: G S Kayonza Girls transc.docx

Doc Creator: tcuhawenimana11

Doc Date: 3/25/2023

Codes Applied: Received information is misleading and risky

Linked Memos: 0

Excerpt Creator: tcuhawenimana11

Excerpt Created On: 4/28/2023

Excerpt Range: 13240-13474

Nimero yange ni 8 mfite imyaka 14 niga muwa 1. Igihe ushobora gutwitira ni igihe umukobwa abaye agiye mu mihango cyangwa habura iminsi mike ngo ayijyemo agakora imibonanompuzabitsina idakingiye. Iminsi mike gute? Habura nkumunsi umwe.

Title: G S Kayonza Girls transc.docx

Doc Creator: tcuhawenimana11

Doc Date: 3/25/2023

Codes Applied: Received information is misleading and risky

Linked Memos: 0

Excerpt Creator: tcuhawenimana11

Excerpt Created On: 4/28/2023

Excerpt Range: 13489-13657

Ndi nimero 5 mfite imyaka 16 nkaba niga mu mwaka 2 wamashuri yisumbuye. Igihe umuntu ashobors gutwita nigihe yagiye mu mihango agakora imibonanompuzabitsina idakingiye.

Title: G S Kayonza Girls transc.docx

Doc Creator: tcuhawenimana11

Doc Date: 3/25/2023

Codes Applied: Received information is misleading and risky

Linked Memos: 0

Excerpt Creator: tcuhawenimana11

Excerpt Created On: 4/28/2023

Excerpt Range: 13693-14083

Igihe yakoze imibonanompuzabitsina idakingiye. Igihe cyose? Wapi ngewe uko mbizi byaterwa nigihe mba naragiriye mu mihango nigihe nayiviriyemo. Wenda ushobora kuba uri mu mihango ugakora imibonanompuzabitsina ibyo sinzi ibyavamo ariko igihe ubura iminsi mike ngo uyijyemo ushobora gutwita (habura nkumunsi nkiminsi 2 cyangwa umwe nge niko mbyumva) ariko ikindi gihe uba uri muri free time.

Title: G S Kayonza Girls transc.docx

Doc Creator: tcuhawenimana11

Doc Date: 3/25/2023

Codes Applied: Information that boys need

Linked Memos: 0

Excerpt Creator: tcuhawenimana11

Excerpt Created On: 4/28/2023

Excerpt Range: 14243-14389

Ndi nimero 8 mfite imyaka 14 niga muwa 1. Amakuru wamuha nukwirinda gukora imibonanompuzabitsina idakingiye no kwirinda kwegerana nabakobwa cyane.

Title: G S Kayonza Girls transc.docx

Doc Creator: tcuhawenimana11

Doc Date: 3/25/2023

Codes Applied: Information that boys need

Linked Memos: 0

Excerpt Creator: tcuhawenimana11

Excerpt Created On: 4/28/2023

Excerpt Range: 14390-14552

Ndi nimero ya 1 mfite imyaka 15 niga mu mwaka wa 1 wamashuri yisumbuye. Ibintu umuhungu yakenera ni nko kwirinda gukora imibonanompuzabitsina idakingiye murakoze.

Title: G S Kayonza Girls transc.docx

Doc Creator: tcuhawenimana11

Doc Date: 3/25/2023

Codes Applied: Information that boys need

Linked Memos: 0

Excerpt Creator: tcuhawenimana11

Excerpt Created On: 4/28/2023

Excerpt Range: 14738-15068

Mba numva amakuru akeneye arukugira umuntu bwite umuganiriza wumukobwa kandi ntugirengo aba ashaka uwumugabo kandi nawe ari umugabo, bya serious ukamuganiriza ukamubwire ati rero ibintu uba urimo byuburaya ntabwo aribyo wowe iturize ugume iwanyu ujye unyurwa nibyo baguhaye niba nta phone baguhaye utuze gusa icyo ushaka ukibone.

Title: G S Kayonza Girls transc.docx

Doc Creator: tcuhawenimana11

Doc Date: 3/25/2023

Codes Applied: Information that boys need

Linked Memos: 0

Excerpt Creator: tcuhawenimana11

Excerpt Created On: 4/28/2023

Excerpt Range: 15093-15147

Agatuza agashyira make akumva ko atakwishyira hejuru.

Title: G S Kayonza Girls transc.docx

Doc Creator: tcuhawenimana11

Doc Date: 3/25/2023

Codes Applied: Information that boys need

Linked Memos: 0

Excerpt Creator: tcuhawenimana11

Excerpt Created On: 4/28/2023

Excerpt Range: 15148-15579

Ndi nimero 4 mfite imyaka 16 nkaba niga mu mwaka wa 3 wamashuri yisubumbuye. Amakuru aba akeneye namakuru yigihe aba arimo bijyanye no kumubwira ubwo bujyimbi arimo uburyo yakitwara, bakamuha amakuru yo gutoza umubiri we igihe agezemo, bakamuha amakuru yuburyo atakwishora mu bintu bibi bakamubwira ko ari imyaka agezemo nyine bakamuturisha nge ndumva ariyo makuru ndetse ninama zo kumubwira uburyo ibyo bintu abitwaramo murakoze.

Title: G S Kayonza Girls transc.docx

Doc Creator: tcuhawenimana11

Doc Date: 3/25/2023

Codes Applied: Information that boys need

Linked Memos: 0

Excerpt Creator: tcuhawenimana11

Excerpt Created On: 4/28/2023

Excerpt Range: 15580-15898

Ndi nimero ya 3 mfite imyaka 16 niga mu mwaka ya 3 wamashuri yisumbuye. Amakuru umwana wumuhungu numva yakenera ugeze mu gihe cyubugimbi ndumva akeneye kuganirizwa nyine mu gihe yumva ashaka nko gukora imibonanompuzabitsina ko yakwifata kandi nyine akirinda gukora imibonanompuzabitsina idakingiye nibyo nakumva nyine.

Title: G S Kayonza Girls transc.docx

Doc Creator: tcuhawenimana11

Doc Date: 3/25/2023

Codes Applied: Information that boys need

Linked Memos: 0

Excerpt Creator: tcuhawenimana11

Excerpt Created On: 4/28/2023

Excerpt Range: 16070-16367

Murakoze ndi nimero 7 mfite imyaka 15 nkaba niga mu mwaka wa 2 wamashuri yisumbuye. Inama mba numva yagirwa nukubwira kwirinda kwishora mubusambanyi kuko hari igihe ashobora kubujyamo akiri mutoya ugasanga ateye inda akiri numwana, aba agomba kugirwa inama akirinda ubusambanyi akanifata murakoze.

Title: G S Kayonza Girls transc.docx

Doc Creator: tcuhawenimana11

Doc Date: 3/25/2023

Codes Applied: Information that boys need

Linked Memos: 0

Excerpt Creator: tcuhawenimana11

Excerpt Created On: 4/28/2023

Excerpt Range: 16541-16844

Ndi nimero 5 nkaba mfite imyaka 16 nkaba niga mu mwaka wa 2 wamashuri yisumbuye. Inama umwana wumuhungu ugeze mu gihe cyubugimbi agirwa nukuganirizwa bakamubwira ko kwishora mu mibonanompuzabitsina idakingiye ashobora kwanduriramo indwara zitandukanye akanatera inda nyine akiga ugasanga biteje ibibazo.

Title: G S Kayonza Girls transc.docx

Doc Creator: tcuhawenimana11

Doc Date: 3/25/2023

Codes Applied: Boys and girls know about the causes leading to the physiological changes during puberty

Linked Memos: 0

Excerpt Creator: tcuhawenimana11

Excerpt Created On: 4/28/2023

Excerpt Range: 16922-17041

Imyitwarire aba yagezemo ndetse nuburyo umubiri we uba umeze nibyo bituma yumva ashaka gukora iyo mibonanompuzabitsina.

Title: G S Kayonza Girls transc.docx

Doc Creator: tcuhawenimana11

Doc Date: 3/25/2023

Codes Applied: Information that boys need

Linked Memos: 0

Excerpt Creator: tcuhawenimana11

Excerpt Created On: 4/28/2023

Excerpt Range: 17115-17399

Ndi nimero 4 nkaba mfite imya 16 nkaba niga mu mwaka wa 3 wamashuri yisumbuye indi nama uwo mwana wumuhungu aba akeneye nukutishora mu biyobyabwenge bitewe nimyitwarire cyangwa nimihindagurikire yumubiri we nuburyo arimo ariyumva niyo nama wayimugira kugirango atajya mubiyobyabwenge.

Title: G S Kayonza Girls transc.docx

Doc Creator: tcuhawenimana11

Doc Date: 3/25/2023

Codes Applied: Information that boys need

Linked Memos: 0

Excerpt Creator: tcuhawenimana11

Excerpt Created On: 4/28/2023

Excerpt Range: 17433-17657

Biterwa nuburyo iyo ngimbi irimo iriyumva. Abikura he se? abikura mu bikundi ninshuti yagize. Ahaaa uvuze ibikundi, ibyo bikundi bimeze bite tubwire? Nyine kwishora mu nshuti mbi akanasenga erega ukamugira inama yo gusenga.

Title: G S Kayonza Girls transc.docx

Doc Creator: tcuhawenimana11

Doc Date: 3/25/2023

Codes Applied: Information that boys need

Linked Memos: 0

Excerpt Creator: tcuhawenimana11

Excerpt Created On: 4/28/2023

Excerpt Range: 17711-17922

Rero inama namugira ni iyo kwirinda inshuti mbi zimushora muribyo biyobyabwenge akagana inshuti nziza zimugira inama nziza ikindi kandi akanasenga kugirango imana ibimufashemo abashe kwitwara neza muribyo bihe.

Title: G S Kayonza Girls transc.docx

Doc Creator: tcuhawenimana11

Doc Date: 3/25/2023

Codes Applied: Linked Memos: 0

Excerpt Creator: tcuhawenimana11

Excerpt Created On: 4/28/2023

Excerpt Range: 18112-18628

Ndi nimero 4 mfite imyaka 16 nkaba niga mu mwaka wa 3 wamashuri yisumbuye. Amakuru nahawe nuko ningera mu gihe cyimihindagurikire nkumva umubiri wange urimo urahindagurika nzifate, amakuru ajyanye no kwifata mu gihe wumva uri mu gihe cyimihindagurikire yumubiri wawe cyangwa se nibyanga nzakoreshe nagakingirizo kandi barambwira nyine ntinzishore mu nshuti mbi nzagenda ninshuti nziza kandi ikindi baranambwira ngo nge Nsenga nyine Imana ibimfashemo nyine ntinjye mu nshuti mbi cyangwa mu bindi bintu bibi murakoze.

Title: G S Kayonza Girls transc.docx

Doc Creator: tcuhawenimana11

Doc Date: 3/25/2023

Codes Applied: Types of information provided to boys and girls during puberty

Linked Memos: 0

Excerpt Creator: tcuhawenimana11

Excerpt Created On: 4/28/2023

Excerpt Range: 18708-19299

Ndi nimero ya 3 mfite imyaka 16 niga mu mwaka wa 3 wamashuri yisumbuye. Amakuru nahawe mu gihe ngeze mu gihe cyubwangavu nuko nyine ngomba kwirinda kugira inshuti mbi, nkirinda ibishuko biri hanze nyine cyangwa nyine kujya muribyo bikundi binywa ibiyobyabwenge kuko ibiyobyabwenge nabyo biri mu bintu bifasha umuntu kwishora mubusambanyi, nkamenya nuko nakwitwara mu gihe nyine umuhungu ashobora kukwaka ubushuti. Nshobora nyine kumva ko ntakintu kibi yakugirira kuko nyine mukundana se ariko nyine ashobora nawe kugushuka kuko mukundana se mugakora imibonanompuzabitsina itateguwe murakoze.

Title: G S Kayonza Girls transc.docx

Doc Creator: tcuhawenimana11

Doc Date: 3/25/2023

Codes Applied: Types of information provided to boys and girls during puberty

Linked Memos: 0

Excerpt Creator: tcuhawenimana11

Excerpt Created On: 4/28/2023

Excerpt Range: 19324-19905

Murakoze ndi nimero 7 mfite imyaka 15 niga mu mwaka wa 2 wamashuri yisumbuye. Amakuru nahawe nukwirinda ibishuko akenshi na kenshi kuko usanga aribyo bishora umwana mu mibonampuzabitsina, kwirinda ibishuko biri hanze, ukifata, ukirinda ninshuti mbi zikujyana mu bikundi byo kunywa ibiyobyabwenge bikaba aribyo byagutera kwishora mu mibonanompuzabitsina idakingiye. Mama wawe yaba yarakugiriye inama ki? Kwirinda ibishuko kuko akenshi iyo abantu batangiye kukubona wazanye amatayi wakuze nyine batangira kugushuka, bakangira inama yo kwirinda inshuti mbi nkirinda ibishuko murakoze.

Title: G S Kayonza Girls transc.docx

Doc Creator: tcuhawenimana11

Doc Date: 3/25/2023

Codes Applied: Sources of information received during puberty

Linked Memos: 0

Excerpt Creator: tcuhawenimana11

Excerpt Created On: 4/28/2023

Excerpt Range: 19688-19694

Mama

Title: G S Kayonza Girls transc.docx

Doc Creator: tcuhawenimana11

Doc Date: 3/25/2023

Codes Applied: Types of information provided to boys and girls during puberty

Linked Memos: 0

Excerpt Creator: tcuhawenimana11

Excerpt Created On: 4/28/2023

Excerpt Range: 20005-20393

Ni mama, mama yarambwiye ati ntukazajye wishinga ibyabandi ujye ukora ibyawe mu rugo utuze. Yarambwiye ngo ntuzigere ukora imibonanompuzabitsina numunsi numwe uzabona umugabo mwiza imana izagutoranyiriza. Gahunda zumunsi uzitangira ryari ukazirangiza ryari? Nzitangira nkibyuka nkakora akazi mu rugo narangiza nkiryamira cyangwa nkareba film nyine ninjoro nabwo nkongera nkarya nkaryama.

Title: G S Kayonza Girls transc.docx

Doc Creator: tcuhawenimana11

Doc Date: 3/25/2023

Codes Applied: Precautions to take during puberty for girls

Linked Memos: 0

Excerpt Creator: tcuhawenimana11

Excerpt Created On: 4/28/2023

Excerpt Range: 20981-21047

Yarambwiye ngo kugenda ninjoro ntabwo ari byiza ku mwana wumukobwa

Title: G S Kayonza Girls transc.docx

Doc Creator: tcuhawenimana11

Doc Date: 3/25/2023

Codes Applied: Precautions to take during puberty for girls

Linked Memos: 0

Excerpt Creator: tcuhawenimana11

Excerpt Created On: 4/28/2023

Excerpt Range: 21070-21132

Yarambwiye ngo si byiza kubera ko uhuriramo nibishuko byinshi.

Title: G S Kayonza Girls transc.docx

Doc Creator: tcuhawenimana11

Doc Date: 3/25/2023

Codes Applied: Pshcological changes occuring during puberty-Girls

Linked Memos: 0

Excerpt Creator: tcuhawenimana11

Excerpt Created On: 4/28/2023

Excerpt Range: 21321-21866

Ngewe nyine uko mu rugo bimeze twebwe iyo hari nkigitaramo dushaka kujyamo nyine ntago ubwira mama nyine ngo uraza izi saha kuko na kumanywa ibishuko wahura nabyo twebwe nyine hari ukuntu tuba turi kumwe nabakuru bacu nyine ntabwo yakohereza wenyine ngo wijyane, ujya mu gitaramo cya ninjoro uri kumwe na mama, niyo mama ashaka kudutuma ahantu nyine adutuma hakiri kare iyo bibaye ngombwa ko aho ashaka kugutuma biba bwije yigirayo cyangwa se nyine papa nyine akajyayo ako ntabwo yakwemera ko uva mu rugo nyine saa kumi nebyiri zarenze murakoze.

Title: G S Kayonza Girls transc.docx

Doc Creator: tcuhawenimana11

Doc Date: 3/25/2023

Codes Applied: Sources of information received during puberty

Linked Memos: 0

Excerpt Creator: tcuhawenimana11

Excerpt Created On: 4/28/2023

Excerpt Range: 22004-22158

Ndi nimero 7 mfite imyaka 15 niga mu mwaka wa 2 wamashuri yisumbuye. Akenshi amakuru usanga tuyakura mu nshuti no kubabyeyi no kwishuri nyine babibigisha.

Title: G S Kayonza Girls transc.docx

Doc Creator: tcuhawenimana11

Doc Date: 3/25/2023

Codes Applied: Sources of information received during puberty

Linked Memos: 0

Excerpt Creator: tcuhawenimana11

Excerpt Created On: 4/28/2023

Excerpt Range: 22159-22540

Murakoze ndi nimero 3 mfite imyaka 16 niga mu mwaka wa 3 wamashuri yisumbuye. Ahantu amakuru tuyakura ni kwishuri abarimu nyine batwigisha, mu rugo kubabyeyi cyangwa se nyine nko mu midugudu mu nama ziberamo murakoze. Ntahandi? Hari nigihe tugira abashyitsi kwishuri bakadusura baturutse mu bigo nyine nkibyo bishinzwe ubuzima bakabituganirizaho uburyo nyine tugomba kwitwara neza.

Title: G S Kayonza Girls transc.docx

Doc Creator: tcuhawenimana11

Doc Date: 3/25/2023

Codes Applied: Sources of information received during puberty

Linked Memos: 0

Excerpt Creator: tcuhawenimana11

Excerpt Created On: 4/28/2023

Excerpt Range: 22583-22863

Murakoze ndi nimero 4 mfite imyaka 16 nkaba niga mu mwaka wa 3 wamashuri yisumbuye. Amakuru ahantu nyakura ibiganiro bitangwa kuma televiziyo nko kuri RTV, nyine ishya kiriya kiganiro kiradufahsa no kuma radiyo za ninyampinga ziradufasha nyine ayo makuru yagiye amfasha murakoze.

Title: G S Kayonza Girls transc.docx

Doc Creator: tcuhawenimana11

Doc Date: 3/25/2023

Codes Applied: Sources of information received during puberty

Linked Memos: 0

Excerpt Creator: tcuhawenimana11

Excerpt Created On: 4/28/2023

Excerpt Range: 22912-23121

Murakoze ndi nimero 2 niga mu mwaka 1 wamashuri yisumbuye mfite imyaka 14. Aho nakuye amakuru nabaza mama ibibazo akansubiza no ku kigo cyurubyiruko no kubiganiro bica kumaradiyo nka ninyampinga byaramfashaga.

Title: G S Kayonza Girls transc.docx

Doc Creator: tcuhawenimana11

Doc Date: 3/25/2023

Codes Applied: Obstacles and risks experienced by boys during the sexual and reproductive health changes occuring during puberty and adolescence

Linked Memos: 0

Excerpt Creator: tcuhawenimana11

Excerpt Created On: 4/28/2023

Excerpt Range: 23785-24324

Murakoze ndi nimero ya 3 mfite imyaka 16 niga mu mwaka wa 3 wamashuri yisumbuye. Ingorane numva abahungu bahura nazo bageze mu gihe cyubugimbi bashobora nyine guhura nabakobwa nyine bibigusha, nabakobwa hari igihe bashuka abahungu cyangwa se nyine bakabararura cyangwa se nyine bagahura nudukundi twabahungu tunywa ibiyobyabwenge babashuka kujya nyine mu busambanyi bakababwira ukuntu nyine ari byiza cyangwa nyine abahungu batagirwa inama nababyeyi cyangwa bataza nyine nko muri ibi bintu twajemo byamahugurwa. Ingorane irimo nugushukwa.

Title: G S Kayonza Girls transc.docx

Doc Creator: tcuhawenimana11

Doc Date: 3/25/2023

Codes Applied: Obstacles and risks experienced by boys during the sexual and reproductive health changes occuring during puberty and adolescence

Linked Memos: 0

Excerpt Creator: tcuhawenimana11

Excerpt Created On: 4/28/2023

Excerpt Range: 24358-24806

Ndi nimero 3 mfite imyaka 18. Ikintu nababwira ingorane umuhungu yagira ari nazo umukobwa yagira, abahungu cyane cyane iyo bageze mu myaka 16 nibwo baba bameze nabi ariko iyo ugize 18 uba warabirenze ahubwo ukigisha abatarabicamo. Tubwire ingorane bahura nazo. Bagira ubushyuhe budashira bakumva bifuza abakobwa cyane kuburyo ashobora kubura umukobwa akanapfa. Cyonyine se? yegoko ni byinshi. Ngaho tubwire. Reka mvuge izo gusa kuko nizo zihutira.

Title: G S Kayonza Girls transc.docx

Doc Creator: tcuhawenimana11

Doc Date: 3/25/2023

Codes Applied: Obstacles and risks experienced by girls during the sexual and reproductive health changes occuring during puberty and adolescence

Linked Memos: 0

Excerpt Creator: tcuhawenimana11

Excerpt Created On: 4/28/2023

Excerpt Range: 24358-24806

Ndi nimero 3 mfite imyaka 18. Ikintu nababwira ingorane umuhungu yagira ari nazo umukobwa yagira, abahungu cyane cyane iyo bageze mu myaka 16 nibwo baba bameze nabi ariko iyo ugize 18 uba warabirenze ahubwo ukigisha abatarabicamo. Tubwire ingorane bahura nazo. Bagira ubushyuhe budashira bakumva bifuza abakobwa cyane kuburyo ashobora kubura umukobwa akanapfa. Cyonyine se? yegoko ni byinshi. Ngaho tubwire. Reka mvuge izo gusa kuko nizo zihutira.

Title: G S Kayonza Girls transc.docx

Doc Creator: tcuhawenimana11

Doc Date: 3/25/2023

Codes Applied: Obstacles and risks experienced by boys during the sexual and reproductive health changes occuring during puberty and adolescence

Linked Memos: 0

Excerpt Creator: tcuhawenimana11

Excerpt Created On: 4/28/2023

Excerpt Range: 24863-25126

Ndi nimero 4 mfite imyaka 16 nkaba niga mu mwaka wa 3 wamashuri yisumbuye. Izindi ngorane bahura nazo nukwishora mumibonanompuzabitsina, indi nukujya mubiyobyabwenge, kugendana ninshuti mbi, gutangira kumva ko wakuze ugasuzugura iwanyu, ugatangira gutaha ninjoro.

Title: G S Kayonza Girls transc.docx

Doc Creator: tcuhawenimana11

Doc Date: 3/25/2023

Codes Applied: Obstacles and risks experienced by boys during the sexual and reproductive health changes occuring during puberty and adolescence

Linked Memos: 0

Excerpt Creator: tcuhawenimana11

Excerpt Created On: 4/28/2023

Excerpt Range: 25139-25547

Bikurikirwa no kwangirika bigatera inda zitateganyijwe bakarwara indwara zanabaviramo nurupfu, ikindi kintu iyo batitwaye neza bajya no mu magereza bagafungwa. Kubera iki? Kubera ya mihindagurikire yumubiri hari igihe iyo batagiriwe inama kwifata byanga usanga bafashe nko kugufu kandi icyo ni ikibazo gihanirwa bakabafunga. Ikindi bagafungwa kubera iki? Kunywa ibiyobyabwenge izo zose ni ingorane murakoze.

Title: G S Kayonza Girls transc.docx

Doc Creator: tcuhawenimana11

Doc Date: 3/25/2023

Codes Applied: Obstacles and risks experienced by boys during the sexual and reproductive health changes occuring during puberty and adolescence

Linked Memos: 0

Excerpt Creator: tcuhawenimana11

Excerpt Created On: 4/28/2023

Excerpt Range: 25651-25848

Ndi nimero 5 mfite imyaka 16 nkaba niga mu mwaka wa 2 wamashuri yisumbuye. Ingorane umwana wumuhungu agira iyo ageze mu bugimbi gutera inda itateganyijwe, kunywa ibiyobyabwenge ndumva ari ibyo nzi.

Title: G S Kayonza Girls transc.docx

Doc Creator: tcuhawenimana11

Doc Date: 3/25/2023

Codes Applied: Obstacles and risks experienced by boys during the sexual and reproductive health changes occuring during puberty and adolescence

Linked Memos: 0

Excerpt Creator: tcuhawenimana11

Excerpt Created On: 4/28/2023

Excerpt Range: 25900-26329

Ndi nimero 7 mfite imyaka 15 niga mu mwaka wa2 wamashuri yisumbuye. Ndumva abo bahungu ingorane bahura nazo hari nkigihe baba banywa ibiyobyabwenge abo bari kubinywana ugasanga barabashutse uwo mwanya bamushoye mumibonanompuzabitsina ugasanga bateye inda bagiye no muri gereza bagakuramo nindwara zitandukanye. Ikindi bahura nacyo niki? Hari igihe banywa ibiyobyabwenge nyine bikabashuka ubwenge ugasanga bararwanye bagakomereka.

Title: G S Kayonza Girls transc.docx

Doc Creator: tcuhawenimana11

Doc Date: 3/25/2023

Codes Applied: Obstacles and risks experienced by girls during the sexual and reproductive health changes occuring during puberty and adolescence

Linked Memos: 0

Excerpt Creator: tcuhawenimana11

Excerpt Created On: 4/28/2023

Excerpt Range: 26557-26723

Ndi nimero 8 mfite imyaka 14 niga muwa 1. Ingorane umukobwa yagira ni nko gutwita no kugendera mugakungu bakamunywesha nawe ibiyobyabwenge no kwigira indaya murakoze.

Title: G S Kayonza Girls transc.docx

Doc Creator: tcuhawenimana11

Doc Date: 3/25/2023

Codes Applied: Obstacles and risks experienced by girls during the sexual and reproductive health changes occuring during puberty and adolescence

Linked Memos: 0

Excerpt Creator: tcuhawenimana11

Excerpt Created On: 4/28/2023

Excerpt Range: 26724-26955

Murakoze ndi nimero 1 mfite imyaka 15 niga mu mwaka wa 1 wamashuri yisumbuye. Ingorane umwana wumukobwa ashobora guhura nazo nugutwara inda zitateganyijwe akarwara nindwara zandurira mumibonanompuzabitsina murakoze numvaga ari izo.

Title: G S Kayonza Girls transc.docx

Doc Creator: tcuhawenimana11

Doc Date: 3/25/2023

Codes Applied: Obstacles and risks experienced by girls during the sexual and reproductive health changes occuring during puberty and adolescence

Linked Memos: 0

Excerpt Creator: tcuhawenimana11

Excerpt Created On: 4/28/2023

Excerpt Range: 27045-27180

Ingorane yahura nazo imiryango iramwanga agahura nibibazo kuburyo byamuviramo no kwiyahura kubera ibibazo byinshi byavuye kuri iyo nda.

Title: G S Kayonza Girls transc.docx

Doc Creator: tcuhawenimana11

Doc Date: 3/25/2023

Codes Applied: Obstacles and risks experienced by girls during the sexual and reproductive health changes occuring during puberty and adolescence

Linked Memos: 0

Excerpt Creator: tcuhawenimana11

Excerpt Created On: 4/28/2023

Excerpt Range: 27359-27590

Ndi nimero 7 mfite imyaka 15 niga mu mwaka wa 2. Hari igihe usanga nyine atwite ugasanga ishuri ahise arireka hanyuma ubundi ugasanga nimiryango ihise imwanga ugasanga akuyemo inda biramwishe ugasanga umwana arapfuye nawe arapfuye.

Title: G S Kayonza Girls transc.docx

Doc Creator: tcuhawenimana11

Doc Date: 3/25/2023

Codes Applied: Obstacles and risks experienced by girls during the sexual and reproductive health changes occuring during puberty and adolescence

Linked Memos: 0

Excerpt Creator: tcuhawenimana11

Excerpt Created On: 4/28/2023

Excerpt Range: 27630-28179

Ndi nimero 3 mfite imyaka 16 niga mu mwaka wa 3 wamashuri yisumbuye. Ngewe ingorane numva umwana wumukobwa ashobora kugira, iyo umwana wumukobwa amaze gukura nyine hari ukuntu iwabo baba bafite ubushobozi bukeye agahura nyine nabagabo bakuze hanze bakamushuka bamushukishije ibintu runaka nyine amatelephone ibintu byo kurya iwabo nyine badashobora kumugurira, bakamutera inda zitateganyijwe bakamutera indwara nka sida umwana bamara kumutera inda nyine akabyara ako nyine akabura ubushobozi bwo gutunga umwana we afite bigatuma aca mu buzima bubi.

Title: G S Kayonza Girls transc.docx

Doc Creator: tcuhawenimana11

Doc Date: 3/25/2023

Codes Applied: Obstacles and risks experienced by girls during the sexual and reproductive health changes occuring during puberty and adolescence

Linked Memos: 0

Excerpt Creator: tcuhawenimana11

Excerpt Created On: 4/28/2023

Excerpt Range: 28222-28472

Ba sugar daddy. Ingorane zo gushukwa na sugar daddy? Amushukisha bombo, telephone cyangwa se kumuha lift mu modoka nyine abagabo babakire akamushuka ko azajya amugeza kwishuri hakiri kare ariko akamuciha ahandi hantu nyine akamukoresha ibikorwa bibi.

Title: G S Kayonza Girls transc.docx

Doc Creator: tcuhawenimana11

Doc Date: 3/25/2023

Codes Applied: Obstacles and risks experienced by girls during the sexual and reproductive health changes occuring during puberty and adolescence

Linked Memos: 0

Excerpt Creator: tcuhawenimana11

Excerpt Created On: 4/28/2023

Excerpt Range: 28724-29004

Umwana nkuwo ashobora kubyara kuko adafite ubushobozi bwo kubyara akabyara abazwe noneho yamara kubagwa nyine bishobora no kumuviramo urupfu kuko aba adafite umwuka uhagije wo gusunika uwo mwana kuko nawe nyine aba ari umwana rero kuzarera umwana nawe ari umwana biba ari ikibazo.

Title: G S Kayonza Girls transc.docx

Doc Creator: tcuhawenimana11

Doc Date: 3/25/2023

Codes Applied: Obstacles and risks experienced by girls during the sexual and reproductive health changes occuring during puberty and adolescence

Linked Memos: 0

Excerpt Creator: tcuhawenimana11

Excerpt Created On: 4/28/2023

Excerpt Range: 29045-29482

Nyine ukuntu twize twize ko umuntu ashobora kuba afite nko mumatako hatoya akajya kubyara nyine bikamurushya bikamwicira nutugingo tumwe na tumwe aba afite mu buzima bwe akavanamo nubumuga. Ashobora kubyara akiri mutoya bikamuviramo no kuzabura urubyaro ingingo ze ziri mu mubiri cyangwa mu myanya myibarukiro zikangirika nyine kuko yabyaye atagejeje igihe cyangwa se umwana nkuriya akabyara adafite abere yo kubona amashereka yo konsa.

Title: G S Kayonza Girls transc.docx

Doc Creator: tcuhawenimana11

Doc Date: 3/25/2023

Codes Applied: Obstacles and risks experienced by girls during the sexual and reproductive health changes occuring during puberty and adolescence

Linked Memos: 0

Excerpt Creator: tcuhawenimana11

Excerpt Created On: 4/28/2023

Excerpt Range: 29552-29856

Urugero ushobora kuba ufite mukuru wawe yarabyariye mu rugo agata ishuri yaranihebye kuburyo ikintu cyose umubwiye atacyumva. Urugero nka mukuru wange niko ameze yabyariye mu rugo abana 2 tumushakira akazi akakanga kuberako yamaze kwiheba kuko hari nabandi bana 2 yabyaye barapfa rero yarihebye bikabije.

Title: G S Kayonza Girls transc.docx

Doc Creator: tcuhawenimana11

Doc Date: 3/25/2023

Codes Applied: Obstacles and risks experienced by boys during the sexual and reproductive health changes occuring during puberty and adolescence

Linked Memos: 0

Excerpt Creator: tcuhawenimana11

Excerpt Created On: 4/28/2023

Excerpt Range: 30074-30340

Ndi nimero 4 mfite imyaka 16 nkaba niga mu mwaka wa 3 wamashuri yisumbuye. Inzitizi bahura nazo harimo nubukene kuko iyo iwanyu muri abakene sometimes ukunda kwifuza rero ibyo bintu byo kwifuza bikaba byakuviramo nko gukora ibintu bibi. Ikindi harimo kureka ishuri.

Title: G S Kayonza Girls transc.docx

Doc Creator: tcuhawenimana11

Doc Date: 3/25/2023

Codes Applied: Obstacles and risks experienced by girls during the sexual and reproductive health changes occuring during puberty and adolescence

Linked Memos: 0

Excerpt Creator: tcuhawenimana11

Excerpt Created On: 4/28/2023

Excerpt Range: 30074-30339

Ndi nimero 4 mfite imyaka 16 nkaba niga mu mwaka wa 3 wamashuri yisumbuye. Inzitizi bahura nazo harimo nubukene kuko iyo iwanyu muri abakene sometimes ukunda kwifuza rero ibyo bintu byo kwifuza bikaba byakuviramo nko gukora ibintu bibi. Ikindi harimo kureka ishuri.

Title: G S Kayonza Girls transc.docx

Doc Creator: tcuhawenimana11

Doc Date: 3/25/2023

Codes Applied: Obstacles and risks experienced by boys during the sexual and reproductive health changes occuring during puberty and adolescence Obstacles and risks experienced by girls during the sexual and reproductive health changes occuring during puberty and adolescence

Linked Memos: 0

Excerpt Creator: tcuhawenimana11

Excerpt Created On: 4/28/2023

Excerpt Range: 30404-30679

Yego iyo uri umukene akenshi na kenshi urifuza wamara kwifuza ugatangira kujya wifuza ibyabandi ukajya kureba abo ba sugar daddy niwanyu abongabo wita abakene ntibabone umwanya wo kukugira inama kuko wabananiye ibyo bikaba byatuma uteshuka nyine izo nshingano zawe murakoze.

Title: G S Kayonza Girls transc.docx

Doc Creator: tcuhawenimana11

Doc Date: 3/25/2023

Codes Applied: Obstacles and risks experienced by girls during the sexual and reproductive health changes occuring during puberty and adolescence

Linked Memos: 0

Excerpt Creator: tcuhawenimana11

Excerpt Created On: 4/28/2023

Excerpt Range: 30680-31646

Murakoze ndi nimero 3 mfite imyaka 16 niga mu mwaka wa 3 wamashuri yisumbuye. Inzitizi abana bakunda guhura nazo nuko nyine iwanyu mushobora kuba mufite ubushobozi bukeya noneho nyine utiga iwanyu bagakunda kugutuma nkahantu ugenda ukaza guhura numuntu akakubwira buryo ki nyine umuntu yakurihira ishuri mu gihe ufite amatsiko yo kumenya abandi ibyo biga ariko akabaza akagira icyo agusaba ku bice byumubiri wawe akakubwira ati wenda turakora imibonanompuzabitsina kugirango wenda nkurihirire ishuri nyine nawe ukabyemera kugirango ubone iryo shuri ako nyuma bikakuviramo ingaruka cyangwa se nko mu miryango yanyu hakazamo ibintu byamakimbirane ukajya ubona mama wawe na papa wawe batabanye neza wowe ukumva ahandi babayeho neza ukifuza nyine kubaho nkabo bandi noneho abo bandi ukajya wumva baganira kubyiza bahura nabyo nawe ugacika iwanyu ukajya gushaka nkakazi mu mujyi rero kugirango ubone nkakazi bakagusaba gukora imibonanompuzabitsina nuko mbyumva murakoze.

Title: G S Kayonza Girls transc.docx

Doc Creator: tcuhawenimana11

Doc Date: 3/25/2023

Codes Applied: Obstacles and risks experienced by girls during the sexual and reproductive health changes occuring during puberty and adolescence

Linked Memos: 0

Excerpt Creator: tcuhawenimana11

Excerpt Created On: 4/28/2023

Excerpt Range: 31723-32047

Ndi nimero 7 mfite imyaka 15 niga mu mwaka wa 2 wamashuri yisumbuye. Ndumva inziti hari nigihe iwanyu muba mufite ubushobozi mwaba mubufite ababyeyi bawe birirwa mu kazi ntibabone umwanya wo kukugira inama ugahura ninshuti mbi mwahura bakagushora mubiyobyabwenge no mumibonanompuzabitsina ugasanga ukuyemo inda ukiri umwana.

Title: G S Kayonza Girls transc.docx

Doc Creator: tcuhawenimana11

Doc Date: 3/25/2023

Codes Applied: Suggestions to improve SRH during puberty and adolescence by girls

Linked Memos: 0

Excerpt Creator: tcuhawenimana11

Excerpt Created On: 4/28/2023

Excerpt Range: 32429-32663

Ndi nimero 4 mfite imyaka 16 nkaba niga mu mwaka wa 3 wamashuri yisumbuye. Icyo nongeraho kubyo twaganiriye nuko mwajya nababyeyi bacu mubagira inama nabo mukabakoresha nkinama mukababwira uburyo bajya batugira inama nibyo byadufasha.

Title: G S Kayonza Girls transc.docx

Doc Creator: tcuhawenimana11

Doc Date: 3/25/2023

Codes Applied: Suggestions to improve SRH during puberty and adolescence by girls

Linked Memos: 0

Excerpt Creator: tcuhawenimana11

Excerpt Created On: 4/28/2023

Excerpt Range: 32673-33026

Nange ibyo mugenzi wange avuze ndabyemera. Hari igihe tuba turi bazima bakadufata nkabasazi kubera ko batigeze bahugurwa, nange ibyifuzo mfite nuko mwafata ababyeyi bacu mukabagira inama mukababwira yuko abana baba bafite uburenganzira. Najyaga mbura uburenganzira murugo nkagira umujinya nkabwira mama ati se ko uri kumbuza kujya kwiga bizagenda bite?

Title: G S Kayonza Girls transc.docx

Doc Creator: tcuhawenimana11

Doc Date: 3/25/2023

Codes Applied: Suggestions to improve SRH during puberty and adolescence by girls

Linked Memos: 0

Excerpt Creator: tcuhawenimana11

Excerpt Created On: 4/28/2023

Excerpt Range: 33079-33385

Ndi nimero 7 mfite imyaka 15 niga mu mwaka wa 2 wamashuri yisumbuye. Ndumva nyine bagaruka ku babyeyi, hari igihe nyine usanga umwana atinya umubyeyi ugasanga atinye kumubwira ko yagiye nko mu mihango ugasanga abigiriyemo numwanda bikamuviramo nindwara. Gukangurira ababyeyi kwiyegereza abana ntibabatinye.

Title: G S Kayonza Girls transc.docx

Doc Creator: tcuhawenimana11

Doc Date: 3/25/2023

Codes Applied: Suggestions to improve SRH during puberty and adolescence by girls

Linked Memos: 0

Excerpt Creator: tcuhawenimana11

Excerpt Created On: 4/28/2023

Excerpt Range: 33386-34299

Murakoze ndi nimero 3 mfite imyaka 16 niga mu mwaka wa 3 wamashuri yisumbuye. Ikindi numva nakongeraho nuko nyine urubyiruko twese hamwe dufite imyumvire itandukanye ndumva nyine mwashyiraho abana nyine tugomba kugirwa inama nkuku turi mu biruhuko tuba twiriwe mu rugo nababyeyi bacu nyine bagiye ku kazi tutabana nabo cyane ugasanga nabo twagisha inama twese turangana nyine duhuje ubwo bwenge bukeya kuko tuba twumva nyine dutinyana ubwacu, rero nkubu hakabayeho nyine ibiganiro bifasha abana kuvugira mu ruhame nibibazo bafite bakabibwira abayobozi nkukunguku ababyeyi batababonera umwanya wo kubaganiriza ukumva watinye mama wawe kumubwira urinze ujya kubibwira undi muntu mutanegeranye ugahura niyo mbogamizi yuko ushobora guhura nikibazo uri imbere ya mama wawe ariwe wakabibwiye ukaba utabimubwira kandi uwo wakabibwiye mutanegeranye wenda uwo wumva wakisanzuraho nyine mukabwira ababyeyi bakatugira inama.
