# Supplementary material for: Adolescent awareness and experience of the pubertal changes: A qualitative study from Rwanda
Source: PLoS One. 2025 Jun 24;20(6):e0325502. doi: 10.1371/journal.pone.0325502 (PMC12186878; doi:10.1371/journal.pone.0325502)
Supplement: S2 File — (DOC) [file pone.0325502.s002.doc]

Title: Mukarange Boys Trnscribed.docx

Doc Creator: tcuhawenimana11

Doc Date: 3/20/2023

Codes Applied: Physiological changes occuring during puberty-Boys

Excerpt Creator: tcuhawenimana11

Excerpt Created On: 3/21/2023

Excerpt Range: 129-252

impinduka umuhungu agira iyo ageze mu kigero cya adolescence eeh atangira kwiroteraho cyane cyane ubundi akazana nu ubwanwa

Title: Mukarange Boys Trnscribed.docx

Doc Creator: tcuhawenimana11

Doc Date: 3/20/2023

Codes Applied: Psychological changes occuring during puberty-Boys

Excerpt Creator: tcuhawenimana11

Excerpt Created On: 3/21/2023

Excerpt Range: 257-414

rimwe na rimwe ko hari ni igihe impinduka nyine usanga iyo ari gukorakora wenda cg atekereza cyane mu bintu bijyanye wenda ni imibonano usanga yirangirijeho.

Title: Mukarange Boys Trnscribed.docx

Doc Creator: tcuhawenimana11

Doc Date: 3/20/2023

Codes Applied: Physiological changes occuring during puberty-Boys

Excerpt Creator: tcuhawenimana11

Excerpt Created On: 3/21/2023

Excerpt Range: 513-718

rero njye ukuntu mbitekereza numva impinduka ziba mu muntu uri muri adolescence w umuhungu, amera insya, atangira kwaguka mu gituza, kugira ijwi rinini kwaguka mu ijwi, kuzana ubwanwa no kuba yakwirotereho

Title: Mukarange Boys Trnscribed.docx

Doc Creator: tcuhawenimana11

Doc Date: 3/20/2023

Codes Applied: Psychological changes occuring during puberty-Boys

Excerpt Creator: tcuhawenimana11

Excerpt Created On: 3/21/2023

Excerpt Range: 727-786

akumva yaba ari kumwe n umukobwa inshuro nyinshi, murakoze.

Title: Mukarange Boys Trnscribed.docx

Doc Creator: tcuhawenimana11

Doc Date: 3/20/2023

Codes Applied: Physiological changes occuring during puberty-Boys

Excerpt Creator: tcuhawenimana11

Excerpt Created On: 3/21/2023

Excerpt Range: 890-1028

eeh nk impinduka zaba ku muhungu hari kumera wenda imwemwe, eeh kumera incakwaha eeh kuniga ijwi rinini eeh ndi kumva ari ibi nakongeraho.

Title: Mukarange Boys Trnscribed.docx

Doc Creator: tcuhawenimana11

Doc Date: 3/20/2023

Codes Applied: Physiological changes occuring during puberty-Boys

Excerpt Creator: tcuhawenimana11

Excerpt Created On: 3/21/2023

Excerpt Range: 1051-1322

cyo nongeraho ku byo bagenzi banjye bavuze byose nibyo ariko akenshi na kenshi usanga ni icyocyere kiwe cyangwa se impumuro yu umubiri we igenda ihinduka, agatangira kugenda abira nki ibyuya byinshi cyane bihumura ukundi kuntu bitari uko yaratuye ahumura, ni ibyo ngibyo.

Title: Mukarange Boys Trnscribed.docx

Doc Creator: tcuhawenimana11

Doc Date: 3/20/2023

Codes Applied: Physiological changes occuring during puberty-Boys

Excerpt Creator: tcuhawenimana11

Excerpt Created On: 3/21/2023

Excerpt Range: 1351-1795

impinduka umwana w umuhungu ugeze mu gihe cyu ubugimbi, impinduka zimubaho aah ubundi mu isura ye hahita hazamwo niho ubonera impinduka ya mbere, amera ibishishi, agatangira kumera ubwanwa, kubyo mugenzi wanjye yavuze impumuro cyangwa se icyokere abira kiba kinshi kandi kigahinduka kikazana indi mpumuro itari nziza cyane ko ku myanya yi ibanga ahita amera insya, akazana nu ubucakwaha, akaba mugari mugituza akagira indeshyo yihuse, murakoze.

Title: Mukarange Boys Trnscribed.docx

Doc Creator: tcuhawenimana11

Doc Date: 3/20/2023

Codes Applied: Psychological changes occuring during puberty-Boys

Excerpt Creator: tcuhawenimana11

Excerpt Created On: 3/21/2023

Excerpt Range: 1825-2084

Yeah amarangamutima ye ahita yi improver cyane ko we ndavugako buri munsi cyangwa isaha ku yindi aba ashaka kumva yahorana n umukobwa baganira kubyo imyororekere baganira kubyo imibonano mpuzabitsina cyane ko aba yumva ashaka no gukora imibonano mpuzabitsina.

Title: Mukarange Boys Trnscribed.docx

Doc Creator: tcuhawenimana11

Doc Date: 3/20/2023

Codes Applied: Psychological changes occuring during puberty-Boys

Excerpt Creator: tcuhawenimana11

Excerpt Created On: 3/21/2023

Excerpt Range: 2136-2381

ibindi bimuhindukaho atangira kumvako amaze gukura agashyiraho inyogosho, agatangira kumva nyine ibintu byose abi fitinzemwo agatangira kuba ari kumwe na abakobwa kenshi na kenshi akumvako buri mukobwa wese abonye bakundana, baryamana murakoze.

Title: Mukarange Boys Trnscribed.docx

Doc Creator: tcuhawenimana11

Doc Date: 3/20/2023

Codes Applied: Psychological changes occuring during puberty-Boys

Excerpt Creator: tcuhawenimana11

Excerpt Created On: 3/21/2023

Excerpt Range: 2522-2711

izindi mpinduka ni ukuba yumva yakora imibonano mpuzabitsina ikindi atangira gukora nkibyo yakoraga akiri umwana agatangira kubikora kubera abayumva abantu benshi bamureba eeh nibyo ngibyo.

Title: Mukarange Boys Trnscribed.docx

Doc Creator: tcuhawenimana11

Doc Date: 3/20/2023

Codes Applied: Psychological changes occuring during puberty-Boys

Excerpt Creator: tcuhawenimana11

Excerpt Created On: 3/21/2023

Excerpt Range: 2813-3244

izindi mpinduka umwana w umuhungu ugeze mu gihe cy ubugimbi agira mubijyanye n amarangamutima atangira kumva yasoretse yumvako ntakintu cyamushobora ahantu hose yumvako nyine yagira nki ibitekerezo byagutse wenda tuvuge nko mu muryango we yagira nawe uruhare rwiwe, hari impinduka ziza nabwo atangira kumva afite inyota yo gushaka gukorere amafaranga ngo agire ibyo hari ibyo abona iwabo bamuhereza bitari ku rwego rwiwe akumvakoo

Title: Mukarange Boys Trnscribed.docx

Doc Creator: tcuhawenimana11

Doc Date: 3/20/2023

Codes Applied: SRH issues young boys and girls might might not be aware of during their puberty

Excerpt Creator: tcuhawenimana11

Excerpt Created On: 3/21/2023

Excerpt Range: 3316-3710

Ingaruka zo kuba ashaka ibyo iwabo badafitiye ubushobozi cyangwa se abona ibyo bamuhaga biri ku rwego rutoya ku urwo agezeho nuko bimutera bishobora gutuma ajya nko gukorera nka ahantu amafaranga bikavamwo kumuhohotera, tuvuge wenda agiye nko murugo rwu umudamu gusabayo akazi wenda agasanga ari nkako kumusaba ko basambana kugirango amwishyure bitso akaba nyine yahandurira nki indwara runaka.

Title: Mukarange Boys Trnscribed.docx

Doc Creator: tcuhawenimana11

Doc Date: 3/20/2023

Codes Applied: Suggestions to improve SRH during puberty and adolescence by boys

Excerpt Creator: tcuhawenimana11

Excerpt Created On: 3/21/2023

Excerpt Range: 3712-4331

Bishobora kumutera ingaruka nanone zo kuba ikirara kuko hari byinshi mu ubushobozi bwa ababyeyi bacu tubasaba bakagira icyo birengagiza ugasanga havuyemwo gutorongera ugiye kuba ku umuhanda ubaye ikirara, ibindi harimwo ingaruka zo kwishora mu ingeso z ubusambanyi nk imibonano mpuzabitsina ugasanga uvanyemwo sida cyangwa se uvanyemwo n izindi ndwara, kimwe yuko ushobora nko gukuramwo urupfu bitewe nuko ibyo ubona byinshi uwemera kubiguha hari ni icyo nawe agusaba nawe ukagendera kuri byabindi ngo ntamyaka 100, koko iryo jambo ntabwo ari ryiza kuko imyaka 100 nayo irahari kandi hari na benshi bayimaze, murakoze.

Title: Mukarange Boys Trnscribed.docx

Doc Creator: tcuhawenimana11

Doc Date: 3/20/2023

Codes Applied: SRH issues young boys and girls might might not be aware of during their puberty

Excerpt Creator: tcuhawenimana11

Excerpt Created On: 3/21/2023

Excerpt Range: 4473-4602

Oya hari gukoresha wenda nki ibikoresho bikomeretsa cyane cyane nku urwembe ushobora kuba warukoresha nabwo mukaba mwakanduzanya.

Title: Mukarange Boys Trnscribed.docx

Doc Creator: tcuhawenimana11

Doc Date: 3/20/2023

Codes Applied: Precautions to take during puberty for boys

Excerpt Creator: tcuhawenimana11

Excerpt Created On: 3/21/2023

Excerpt Range: 4902-5096

imibonano mpuzabitsina yose idakingiye yatera uburwayi kubera yuko nta protection cyangwa se kwirinda kuba kuri hagati aho ngaho kandi iyo habuzemwo kwirinda niho dukuramwo indwara zo kwandura.

Title: Mukarange Boys Trnscribed.docx

Doc Creator: tcuhawenimana11

Doc Date: 3/20/2023

Codes Applied: Boys and girls know about the causes of the causes leading to the physiological changes during puberty

Excerpt Creator: tcuhawenimana11

Excerpt Created On: 3/21/2023

Excerpt Range: 5292-5917

njye nkuko mbitekereza impinduka ikiba cyaziteye, umubiri w umwana w umuhungu ugira nk imisemburo nk icyo nakwita nki imisemburo, imisemburo wenda buriya ni karemano iba iri mu muntu iba ishinzwe wenda gukora icyo gikorwa runaka iba yakoze reka tuvugeko nayo iba yakoze rero iyo ugeze muri icyo gihe umuhungu aba afite ubushobozi bwo kuba yatera inda ntekereza ko ari cyo gituma aribwo utangira kubona yamihindagurikire twabonye kuba wabyutse ugasanga wiroteyeho, kuryama ukabyuka mugitondo cyakare ugasanga igitsina cyawe yahagaze tubyita gushyukwa muri make, njyewe nibyo mbona byaba bitera iriya mihindagurikire, murakoze.

Title: Mukarange Boys Trnscribed.docx

Doc Creator: tcuhawenimana11

Doc Date: 3/20/2023

Codes Applied: Boys and girls know about the causes of the causes leading to the physiological changes during puberty

Excerpt Creator: tcuhawenimana11

Excerpt Created On: 3/21/2023

Excerpt Range: 6037-6649

Murakoze, ubundi iyo umuntu avutse hari imisemburo cyangwa se ama arumone (harmones) yego umuntu avukana zikaba arizo zigenda zitera guhindagurika kuko urabona nkuko umuntu akura niko nayo igenda ikura, uko ikura nayo niko igenda igira ibindi bintu igutegeka gukora utarutuye ukora, iyo misemburo yo mumubiri haba ukuntu nabyita njyewe ninkuko umuntu yavuga ngo amabya aba yakuze kubera yuko niyo atangira guproducinga intanga ngabo, izo ntanga ngabo rero ukumva mu mubiri wawe zimeze nkiziri kugukirigita cyangwa zirakubangamiye ukumva waharanira yuko zisohoka zikagenda, ndumva ari nkuko umusemburo waba umeze.

Title: Mukarange Boys Trnscribed.docx

Doc Creator: tcuhawenimana11

Doc Date: 3/20/2023

Codes Applied: Physiological changes occuring during puberty-Girls

Excerpt Creator: tcuhawenimana11

Excerpt Created On: 3/21/2023

Excerpt Range: 6826-7025

Okay murakoze, ndi nimero 2, impinduka zigaragara ku mwangavu harimwo kumera amabere, kwaguka kwi imyanya myibarukiro yiwe nayo iriyongera, kugira akajwi kameze nka kagenda slowly kiyungurura ukuntu,

Title: Mukarange Boys Trnscribed.docx

Doc Creator: tcuhawenimana11

Doc Date: 3/20/2023

Codes Applied: Psychological changes occuring during puberty-Girls

Excerpt Creator: tcuhawenimana11

Excerpt Created On: 3/21/2023

Excerpt Range: 7026-7145

umwangavu atangira kugira isoni zo kuba wenda yakwiyambika ubusa abantu bakabona imyanya myibarukiro yiwe, nibyo numva.

Title: Mukarange Boys Trnscribed.docx

Doc Creator: tcuhawenimana11

Doc Date: 3/20/2023

Codes Applied: Physiological changes occuring during puberty-Girls

Excerpt Creator: tcuhawenimana11

Excerpt Created On: 3/21/2023

Excerpt Range: 7232-7377

icyo numva ko umukobwa yageze mugihe cyu ubwa adolescence impinduka zibaho nuko aragenda agakura akagira ikibuno kinini, akazana amabere manini,

Title: Mukarange Boys Trnscribed.docx

Doc Creator: tcuhawenimana11

Doc Date: 3/20/2023

Codes Applied: Psychological changes occuring during puberty-Girls

Excerpt Creator: tcuhawenimana11

Excerpt Created On: 3/21/2023

Excerpt Range: 7378-7474

agatangira kwitinya eeh agatangira no kwigirira isuku nuko atarasanwe akora, eeh nibyo murakoze.

Title: Mukarange Boys Trnscribed.docx

Doc Creator: tcuhawenimana11

Doc Date: 3/20/2023

Codes Applied: Physiological changes occuring during puberty-Girls

Excerpt Creator: tcuhawenimana11

Excerpt Created On: 3/21/2023

Excerpt Range: 7475-7744

Eeh nk impinduka umukobwa ashobora kuba yagira ageze mu ikegero tugezemwo hari kuba yamera amabere, kuba yamera nawe isya, kuba imyanya myibarukiro ye yaba migari kurusha uko yarameze akiri umwana, eeh no kugira nyine ijwi rimeze ukuntu nyine rimeze neza, eeh murakoze.

Title: Mukarange Boys Trnscribed.docx

Doc Creator: tcuhawenimana11

Doc Date: 3/20/2023

Codes Applied: Physiological changes occuring during puberty-Girls

Excerpt Creator: tcuhawenimana11

Excerpt Created On: 3/21/2023

Excerpt Range: 7745-7878

Ikindi nabunganira na none gishobora kujyaho, iyo atangiye gukura ageze muri iyo myaka ajya no mu mihango, atangira kujya mu mihango.

Title: Mukarange Boys Trnscribed.docx

Doc Creator: tcuhawenimana11

Doc Date: 3/20/2023

Codes Applied: Psychological changes occuring during puberty-Girls

Excerpt Creator: tcuhawenimana11

Excerpt Created On: 3/21/2023

Excerpt Range: 7903-8074

ikindi kintu umukobwa kiyongeraho kuri we iyo ageze mu igihe cy ubwangavu atangira gukunda abahungu cyane akumva aho ari hose akeneye wenda kuba yaba ari kumwe n abahungu

Title: Mukarange Boys Trnscribed.docx

Doc Creator: tcuhawenimana11

Doc Date: 3/20/2023

Codes Applied: Psychological changes occuring during puberty-Girls

Excerpt Creator: tcuhawenimana11

Excerpt Created On: 3/21/2023

Excerpt Range: 8102-8549

Eeh ni abakobwa bose bageze mu igihe cyu ubwangavu ni uko hari abihagararaho ariko bibabaho, umukobwa agatangira kugira icyo bita hips, eeh umukobwa akamera amabere, ubundi akamera n isya ku igitsina cye, cyane cyane ku mukobwa ukuntu nyine aba ateye imico ye igenda ihinduka, niba yari umukobwa wenda wa agasazi cyane mu ubwana bwe iyo amaze kugera mu igihe cy ubukure aratangira agahinduka agatangira kwitonda akagira isoni, eeeh nibyo murakoze.

Title: Mukarange Boys Trnscribed.docx

Doc Creator: tcuhawenimana11

Doc Date: 3/20/2023

Codes Applied: Physiological changes occuring during puberty-Girls

Excerpt Creator: tcuhawenimana11

Excerpt Created On: 3/21/2023

Excerpt Range: 8582-8728

noneho impinduka umukobwa yagira atangiye kugera mu ikigero cy ubwangavu, ashobora kumera incakwaha, akamera amabere cyangwa akajya no mu mihango.

Title: Mukarange Boys Trnscribed.docx

Doc Creator: tcuhawenimana11

Doc Date: 3/20/2023

Codes Applied: Boys and girls know about the causes of the causes leading to the physiological changes during puberty

Excerpt Creator: tcuhawenimana11

Excerpt Created On: 3/21/2023

Excerpt Range: 8840-10089

umukobwa igitera izo mpinduka ziwe nawe ni iyo misemburo ijyanjye nu umubiri we ku imihindagurikire ye iba igenda, tuvuge nkiyo agiye mumihango hari nkicyo twakwita nk igi cyangwa se intanga ngore iba yakuze ikaza aho nakwita muri nyababyeyi ishaka ko wenda yahahurira ni ingabo ngo bigire ikindi kintu bireme, bireme wenda umwana ubwo, noneho iyo ije ikayibura kuriya ajya mu mihango akava ariya amaraso niya iba yabaye expired yashwanyutse yaje ikayibura ikaba perishable nyine ubwo ikaba ngombwa ko imyanda isohoka, murakoze.

Nanjye uko mbitekereza nkuko wenda k umuhungu twe ibyacu tuba tubyita sperms kubakobwa bo tubona nkuko abivuze ni nk igi riba riri aho twakwita nko muri ovary, ariho ritangirira gukurira nko mu iminsi wenda nko kuva k umunsi umwe kugera nko kuminsi wenda nka 24 aho rivamwo rikimuka rikanjya aho twakwita nko muri ovudagiti aho ngaho iyo rigiyemwo riba ryiteguye kuba ryarema n umwana riba rikuze, niho riba rigiye kuba ryategereza intanga ngabo bibaye ngombwa, nibwo rero iyo umukobwa atabashije gukora imibonano mpuzabitsina ngo yantanga ngabo ngo iboneke nibwo ntekerezako aribwo ryagi ubwo riba rirengeje igihe ukwezi kwabo kuba kuzuye then rigashwanyuka nibwo tubona yagiye mu mihango ya myanda igasohoka, murakoze.

Title: Mukarange Boys Trnscribed.docx

Doc Creator: tcuhawenimana11

Doc Date: 3/20/2023

Codes Applied: Boys and girls know about the causes of the causes leading to the physiological changes during puberty

Excerpt Creator: tcuhawenimana11

Excerpt Created On: 3/21/2023

Excerpt Range: 10141-10523

Eeh n imisemburo iba nyine yahindaguritse muri we eeh igatuma nyine ariyo, agirigira icyo gikorwa cyo kuba nyine yamera izo sya, n imisemburo nyine ye iba yaje nyine igategereza uburyo bitewe nyine n ukuntu ubuzima bwe nyine bumeze niba wenda afite igikuriro bikaba ngombwa nyine ko iyo misemburo iyo ibaye myinshi mu mubiri bituma nyine yahita aba yazana yamera izo isya, murakoze.

Title: Mukarange Boys Trnscribed.docx

Doc Creator: tcuhawenimana11

Doc Date: 3/20/2023

Codes Applied: Boys and girls know about the causes of the causes leading to the physiological changes during puberty

Excerpt Creator: tcuhawenimana11

Excerpt Created On: 3/21/2023

Excerpt Range: 10581-10700

Biba byatewe n imisemburo igaruka iza iyo yakuze, eeh iyo yageze mu bwangavu iyo misemburo ituma ibyo bintu byose biza.

Title: Mukarange Boys Trnscribed.docx

Doc Creator: tcuhawenimana11

Doc Date: 3/20/2023

Codes Applied: Boys and girls know about the causes of the causes leading to the physiological changes during puberty

Excerpt Creator: tcuhawenimana11

Excerpt Created On: 3/21/2023

Excerpt Range: 10701-10763

Hari nk izina ry umusemburo n umwe wambwira?

Oya ntabwo nyizi.

Title: Mukarange Boys Trnscribed.docx

Doc Creator: tcuhawenimana11

Doc Date: 3/20/2023

Codes Applied: Precautions to take during puberty for boys

Excerpt Creator: tcuhawenimana11

Excerpt Created On: 3/21/2023

Excerpt Range: 10975-11076

Uko tubyitwaramwo ntakundi mbere na mbere ubundi n ukwitwararika nyine, okay nukwirinda peer pressure

Title: Mukarange Boys Trnscribed.docx

Doc Creator: tcuhawenimana11

Doc Date: 3/20/2023

Codes Applied: Precautions to take during puberty for boys

Excerpt Creator: tcuhawenimana11

Excerpt Created On: 3/21/2023

Excerpt Range: 11096-11196

Nibwo bukiza, urumva ndacyashaka ntabwo nabireka gusa ariko nyine ngomba kwirinda na peer pressure.

Title: Mukarange Boys Trnscribed.docx

Doc Creator: tcuhawenimana11

Doc Date: 3/20/2023

Codes Applied: Precautions to take during puberty for boys

Excerpt Creator: tcuhawenimana11

Excerpt Created On: 3/21/2023

Excerpt Range: 11268-11696

Oya, irakonta cyane ko ishobora yo hari n igihe igushuka ukumvako nyine warenze uri undi wundi, kuba warameze ubwanwa ntabwo bivuze ko wabaye umugabo, ibingibi n ibimenyetso bya mbere biza ku muntu w umuhungu, noneho uko abyitwaramwo cyangwa se kwitwararika ntakundi umuntu aba agomba kugira uko agenda, ukirinda iyo peer pressure, ukirinda agakungu, ukirinda gukora imibonano mpuzabitsina idakingiye, eeh ndakeka ar ibyongibyo.

Title: Mukarange Boys Trnscribed.docx

Doc Creator: tcuhawenimana11

Doc Date: 3/20/2023

Codes Applied: Precautions to take during puberty for boys

Excerpt Creator: tcuhawenimana11

Excerpt Created On: 3/21/2023

Excerpt Range: 11707-12311

kubijyanye nk icyo ngicyo cy uburyo twitwara muri icyo bintu ugerageza kumva yuko ibibaye umubiri wawe nubwo byagenda gute utemerewe kugutegeka, kuko ikintu cyose kiva mu bwonko kandi ubwonko butatekereje na none ibintu ngo ubuhakanire n umutima wawe nama bwagushuka, kuko bwazajya butekereza ngo ariko buriya kwica ni byiza ugahita ufatiraho ukabikora ariko ugomba kugisha umutima wawe inama ukamenya ngo icyo gukora ni kiza, nkanjye iyo ngerageje gutekereza ibyo bintu mpita mfata umwanzuro nkavuga ati oya hari ibidakwiye kuko hari byinshi ntarageraho kuko ntabwo navuga ngo ngiye gukora iyo mibonano.

Title: Mukarange Boys Trnscribed.docx

Doc Creator: tcuhawenimana11

Doc Date: 3/20/2023

Codes Applied: Precautions to take during puberty for boys

Excerpt Creator: tcuhawenimana11

Excerpt Created On: 3/21/2023

Excerpt Range: 11708-12311

ubijyanye nk icyo ngicyo cy uburyo twitwara muri icyo bintu ugerageza kumva yuko ibibaye umubiri wawe nubwo byagenda gute utemerewe kugutegeka, kuko ikintu cyose kiva mu bwonko kandi ubwonko butatekereje na none ibintu ngo ubuhakanire n umutima wawe nama bwagushuka, kuko bwazajya butekereza ngo ariko buriya kwica ni byiza ugahita ufatiraho ukabikora ariko ugomba kugisha umutima wawe inama ukamenya ngo icyo gukora ni kiza, nkanjye iyo ngerageje gutekereza ibyo bintu mpita mfata umwanzuro nkavuga ati oya hari ibidakwiye kuko hari byinshi ntarageraho kuko ntabwo navuga ngo ngiye gukora iyo mibonano.

Title: Mukarange Boys Trnscribed.docx

Doc Creator: tcuhawenimana11

Doc Date: 3/20/2023

Codes Applied: How to take care of themselves when phsyiological changes occur for boys

Excerpt Creator: tcuhawenimana11

Excerpt Created On: 3/21/2023

Excerpt Range: 12368-12701

Nagize ubushyuhe, yego mpita numva yuko ari ibintu bisanzwe ari nk uko naba ntanabufite nkahita njya muri douche nkakaraba, biragabanuka iyo ukarabye, naho ku bijyanye n imibonano mpuzabitsina iyo byanze, hari ukuntu bigeraho bikanga bya burundu wifashisha agakingirizo mu rwego rwo kugira ngo utagira ibyo wangiza nibyo ubangamira.

Title: Mukarange Boys Trnscribed.docx

Doc Creator: tcuhawenimana11

Doc Date: 3/20/2023

Codes Applied: How to take care of themselves when phsyiological changes occur for boys

Excerpt Creator: tcuhawenimana11

Excerpt Created On: 3/21/2023

Excerpt Range: 12745-12881

njyewe uko byifatamwo wenda nkuko umuntu abyuka agasanga bimeze nabi, nanjye ngerageza kwitwararika kuko akundi wakora n imyaka ibikora,

Title: Mukarange Boys Trnscribed.docx

Doc Creator: tcuhawenimana11

Doc Date: 3/20/2023

Codes Applied: Precautions to take during puberty for girls

Excerpt Creator: tcuhawenimana11

Excerpt Created On: 3/21/2023

Excerpt Range: 13001-13297

nibyo koko uko twese turi hano ntabwo twabura incuti z abakobwa kuko wenda ntabwo wajya kwandika ibaruwa ubundi wakoresha nk enterinete cyagwa ukandika sms, icyo gihe iyo byakunaniye wumva kwifata byanze ubundi ukoresha agakingirizo, kuko ufite nko kugenda wenda byanze agakingirizo ntugakoreshe,

Title: Mukarange Boys Trnscribed.docx

Doc Creator: tcuhawenimana11

Doc Date: 3/20/2023

Codes Applied: Precautions to take during puberty for boys

Excerpt Creator: tcuhawenimana11

Excerpt Created On: 3/21/2023

Excerpt Range: 13501-13576

Ubwo wagutegetse ntayandi mahitamwo waba ufite ubwo wakoresha agakingirizo.

Title: Mukarange Boys Trnscribed.docx

Doc Creator: tcuhawenimana11

Doc Date: 3/20/2023

Codes Applied: Precautions to take during puberty for boys

Excerpt Creator: tcuhawenimana11

Excerpt Created On: 3/21/2023

Excerpt Range: 13698-13899

Eeh nanjye ubwanjye ndi nimero 6, eeh jyewe natwe turi abantu ugeregeza kwirinda nyine wenda ukagerageza gutegeka ibyo umubiri wawe ushaka kugutegeka ukabiyobora ariko iyo byanze ukoresha agakingirizo.

Title: Mukarange Boys Trnscribed.docx

Doc Creator: tcuhawenimana11

Doc Date: 3/20/2023

Codes Applied: Precautions to take during puberty for boys

Excerpt Creator: tcuhawenimana11

Excerpt Created On: 3/21/2023

Excerpt Range: 14005-14126

Okay ubonye utagafite ugerageza uburyo ki wikontorora ukagerageza buryo ki ukuntu watekereje waba utagafite ukabyihorera.

Title: Mukarange Boys Trnscribed.docx

Doc Creator: tcuhawenimana11

Doc Date: 3/20/2023

Codes Applied: How boys experience physiological changes that occured to them during puberty

Excerpt Creator: tcuhawenimana11

Excerpt Created On: 3/21/2023

Excerpt Range: 14251-15337

hari igihe utekereza imibonano mpuzabitsina uryamye cyangwa se mu gitondo igitsina cyawe cyanze pe cyahagaze nk igiti ugahamagara cher wawe kuri fone uba wumva nawe akibyuka cyangwa akiryamye wenda nka saa kumi nimwe gutyo saa kumi n igice ukamubwira uti byanze ni ukuri ariko n abakobwa nabo ndabizi ibyo biganiro barabikunda iyo muganishije kuriyo ngingo babyumva vuba ariko aba afite amatsiko ati ese urumva bimeze bite se? Ngo ubwose ingana ute? Ati shawu byihorere ntabwo uzi ukuntu meze ngo nonese ko ntaza basi ngo nze mpite nkutabara ati shaka uburyo unyeranjamwo ndebe ko narangiza wenda birahita birangira ngo basi koraho wimagina ko arinjye uri gukoraho ati mera gutya ndimajina ko arinjye uri kubikora ukaza gushiduka umeze nkaho uri gusambana n umuntu nubundi kuberako intekerezo wazimushizeho ibyo akorera kuri fone ukabikora wowe wimagina ko ariwe uri kubikora bitewe nuko mubuzima bwose muba musanganywe niyo muri kumwe uba umukorakoraho ati kora kwibere ndahita nibuka ko cyagihe narikozeho cyangwa se ndatekerezako ngiye kubikora byose bigakora igikorwa bita kwinisha.

Title: Mukarange Boys Trnscribed.docx

Doc Creator: tcuhawenimana11

Doc Date: 3/20/2023

Codes Applied: How boys experience physiological changes that occured to them during puberty

Excerpt Creator: tcuhawenimana11

Excerpt Created On: 3/21/2023

Excerpt Range: 15373-15408

Kirakemuka urarangiza eeh murakoze.

Title: Mukarange Boys Trnscribed.docx

Doc Creator: tcuhawenimana11

Doc Date: 3/20/2023

Codes Applied: How to take care of themselves when physiological changes occur-girls

Excerpt Creator: tcuhawenimana11

Excerpt Created On: 3/21/2023

Excerpt Range: 15566-16008

amakuru aba akenewe ni uko wowe ugiye kuyamubwira mugomba kuba muri inshuti, mukaganira amakuru yose ukamubwira ntabwoba nawe akakubwira ntabwoba nibwo mubasha kubikemura ariko iyo ugiye umucengacenga sumuganirize nawe ntacyo akubwira ugasanga byamubayeho wenda aje gutwara inda idateguwe cyangwa yanduriyemwo izindi ndwara, ariko iyo mubaye inshuti wenda umubyeyi akaba inshuti n umwana we ibyo byose bakaganira ntakintu kijya kiba murakoze.

Title: Mukarange Boys Trnscribed.docx

Doc Creator: tcuhawenimana11

Doc Date: 3/20/2023

Codes Applied: How to take care of themselves when physiological changes occur-girls Precautions to take during puberty for girls

Excerpt Creator: tcuhawenimana11

Excerpt Created On: 3/21/2023

Excerpt Range: 16081-16527

amakuru umukobwa akeneye amakuru ya mbere na mbere n ukuganirizwa, akagirwa inama, ukamuganiriza kubijyanye n imihindagurikire y umubiri we, ukamwumvisha ko nubwo arimwo arahindagurika mu mubiri hari ibyo umubiri umusaba gukora we yagerageza akabikwepa byananga agahitamwo yuko yaganira nuwo bagiye kugira icyo bakorana uburyo bakwirinda mwo kuko nubwo uba ugeze mu imyaka ya adolescence uba utaragera ku imyaka leta y Urwanda yemereraho gushaka.

Title: Mukarange Boys Trnscribed.docx

Doc Creator: tcuhawenimana11

Doc Date: 3/20/2023

Codes Applied: How girls experience changes occuring to them during puberty

Excerpt Creator: tcuhawenimana11

Excerpt Created On: 3/21/2023

Excerpt Range: 16586-17054

Kubera ko umukobwa akenshi ugeze muri adolescence ubundi aba atinya kubaza hari ibintu byinshi aba agikwepa kwepa ariko umuhungu we ugeze muri adolescence atangira kumva we yuko aricyo gihe ngo amenye buri kimwe cyose njye nkiyo abimbwiye ngerangeza kumubwira ati bimwe ntabyo nzi ibindi ndabizi, ibyo nzi nkabimusobanurira noneho ibyo ntazi nkamubwira nti impa umwanya ndazakubaza nk abandi bantu bakuze basubize nibansubiza igisubizo bari bunsubize ndagusubiza nawe.

Title: Mukarange Boys Trnscribed.docx

Doc Creator: tcuhawenimana11

Doc Date: 3/20/2023

Codes Applied: How to take care of themselves when physiological changes occur-girls

Excerpt Creator: tcuhawenimana11

Excerpt Created On: 3/21/2023

Excerpt Range: 17096-17481

Akenshi akunda kumbaza nk ibibazo bijyanye no gukora mbonano mpuzabitsina akenshi wenda nk iyo twabikoze aba arimwo arambaza ngo ubundi uba wumva ufitinga gute? Uba wumva umeze muri wowe gute? Ese nk ubungubu uragutse ugize gutya bikaba ngombwa ko untera inda wakora iki? Nkamubwirango wenda ku imyaka yacu ntabwo turageza igihe cyo kubana reka tujye twihangana dukoreshe agakingirizo.

Title: Mukarange Boys Trnscribed.docx

Doc Creator: tcuhawenimana11

Doc Date: 3/20/2023

Codes Applied: Precautions to take during puberty for boys Precautions to take during puberty for girls

Excerpt Creator: tcuhawenimana11

Excerpt Created On: 3/21/2023

Excerpt Range: 17599-17889

Utunwa? Utunwa nyine umuntu aradufata nitwo ahubwo utunwa niyo mbarutso yo kugirango munakore imibonano mpuzabitsina, kuko ntabwo wakwifata ngo umuntu aturutse murugo iwabo nawe uri iwanyu araje neza ngo uhite uvuga ngo urarambitse ntabwo waba uri itungo ubanza kugira uburyo umuryoshyamwo.

Title: Mukarange Boys Trnscribed.docx

Doc Creator: tcuhawenimana11

Doc Date: 3/20/2023

Codes Applied: Precautions to take during puberty for girls

Excerpt Creator: tcuhawenimana11

Excerpt Created On: 3/21/2023

Excerpt Range: 18091-19061

njyewe amakuru ndemera ko umukobwa aba agomba kuganirizwa yaba n ababyeyi cyangwa se n inshuti ze cyangwa n inzego z umutekano kugirango ya mihindagurikire nimubaho atangira ngo n ikindi kibazo yagize amenye ko ari ibintu byamubayeho yaragambwe aziko bizamubaho bimutegereje ari ibintu bisanzwe ataniheba, muri make umukobwa utanamuganirije ko niyo condom ibaho ashobora no kuba ataziko inabaho yanayibonana umuhungu akaba atanamenya uko banayambara cyangwa n umuhungu yamushuka abahungu bo ubundi condom we nta nubwo ajya anayitekerezaho cyane birenze, aho bimufatiye we nubundi umuhaye ntiyabyanga ngo nuko ntayo afite ariko umukobwa we ugomba kumuganiriza akamenya ko mu igihe bizaba ngombwa ko yabikora byanze umuhungu yamunaniye akamenya ko ubwe we niyo condom yakabaye anayifitiye akaba yamubwira ngo cheri rero banza wambare iyi condom bityo rero urumva ko byaba ari byiza ko umukobwa agomba kuganirizwa ibyo byose akaba abizi nihagire ikimutera ubwoba, murakoze.

Title: Mukarange Boys Trnscribed.docx

Doc Creator: tcuhawenimana11

Doc Date: 3/20/2023

Codes Applied: How to take care of themselves when physiological changes occur-girls Received information is misleading and risky

Excerpt Creator: tcuhawenimana11

Excerpt Created On: 3/21/2023

Excerpt Range: 19134-19757

hari ukuntu nk umuhungu amushuka iyo bakundana akamubwira ati rero iyo tubikoze ntanikintu twaba nta n agakingirizo bituma utarwara ibishishi cyangwa ukazana taye rero iyo aramutse ataraganiriye n ababyeyi be ngo bamusobanurire wenda nibyo bintu byamuviramwo nk ibibazo cg indwara cyangwa kubura ejo heza he intego afite yahita irangirira aho, iyo batamusobanuriye ngo bamubwirengo iyo uko yakuramwo inda nizangaruka niho usanga yemeye nibyo umuhungu yamubwiye kuko ntabumenyi abifiteho, aakameyako niba bimunaniye agomba gukoresha ako gakingirizo ahubwo bakazibuka kumugira inama bamuha ubwo bumenyi aruko ingaruka zaje.

Title: Mukarange Boys Trnscribed.docx

Doc Creator: tcuhawenimana11

Doc Date: 3/20/2023

Codes Applied: Received information is misleading and risky

Excerpt Creator: tcuhawenimana11

Excerpt Created On: 3/21/2023

Excerpt Range: 19947-20723

Aba abizi KO ari kumushuka kuko we icyo aba akeneye wenda aba akeneye ni umuti we uribumuvure.

Okay ndi nimero 9 nanjye, impamvu mvuga yukoi byo by umuhungu ashobora kuba afite ubumenyi cg se atabumenye ntabumenyi abifiteho biterwa n muhungu uburyo ari shapu, hari umuhungu uba ari shapu akaba yabasha kubaza umubyeyi ariko nk abo bana bababatuye akenshi bibana bataraganiriye cyane n ababyeyi, we aba yumva yuko ibyo bintu ataribyo cg se akumvako aribyo, njyewe ku giti cyanjye usibye ko nazashaka nk umwanya nkegera muganga nanjye ako kantu najyaga nkibazaho gusa nanubu wenda murwego rwo kwitabara namubwira ati ndakuvura ariko nyie nta makuru menshi tuba tubifiteho, yego.

Ibyo ngibyo mba numva ataribyo, mba numva ibyo ngibyo ubivuga uba umeze nkuri kwitabara umubeshya.

Title: Mukarange Boys Trnscribed.docx

Doc Creator: tcuhawenimana11

Doc Date: 3/20/2023

Codes Applied: Types of information provided to boys and girls during puberty

Excerpt Creator: tcuhawenimana11

Excerpt Created On: 3/21/2023

Excerpt Range: 20067-20206

impamvu mvuga yukoi byo by umuhungu ashobora kuba afite ubumenyi cg se atabumenye ntabumenyi abifiteho biterwa n muhungu uburyo ari shapu

Title: Mukarange Boys Trnscribed.docx

Doc Creator: tcuhawenimana11

Doc Date: 3/20/2023

Codes Applied: Sources of information received during puberty

Excerpt Creator: tcuhawenimana11

Excerpt Created On: 3/21/2023

Excerpt Range: 20207-20341

hari umuhungu uba ari shapu akaba yabasha kubaza umubyeyi ariko nk abo bana bababatuye akenshi bibana bataraganiriye cyane n ababyeyi

Title: Mukarange Boys Trnscribed.docx

Doc Creator: tcuhawenimana11

Doc Date: 3/20/2023

Codes Applied: Received information is misleading and risky

Excerpt Creator: tcuhawenimana11

Excerpt Created On: 3/21/2023

Excerpt Range: 20811-21364

impamvu nemezako ibyo bintu byo kubeshya umukobwa mugihe uri kumusaba ko mukora imibonano mpuzabitsina nuko nimba tubyibuka twese abantu bisiramuje iyo ukiva kwisiramuza har igihe bakubwirango kugirango icyo bita nk inzembe cyangwa se buriya buryaryate nyuma yuko bagukuramwo indodo buvemo wagombye gusaba umukobwa mugakora imibonano mpuzabitsina ariko njyewe nisiramuza ntacyo nigeze nkora kandi ubwo buryaryate ntabwo nigeze numva, niyo mpamvu mvuga ko byose ari ibinyoma byo kugira ngo bashe kugwa mur ibyo bishuko gusa mukore imibonano mpuzabitsina.

Title: Mukarange Boys Trnscribed.docx

Doc Creator: tcuhawenimana11

Doc Date: 3/20/2023

Codes Applied: Precautions to take during puberty for girls

Excerpt Creator: tcuhawenimana11

Excerpt Created On: 3/21/2023

Excerpt Range: 21375-21688

N andi makuru umukobwa akeneye kuba yamenya hari ukumwereka yuko ibyo umuhungu aba akora uba uzi ibyaribyo uba ushaka kwivura ariko umukobwa we umenya yuko ibyo wamuhaga atari ukuvura ari uko ushoje ibyo ukora ariko n ubundi umukobwa ugasanga umuteye ikibazo ari wowe umuhungu uba wivura ariko we umutera indwara.

Title: Mukarange Boys Trnscribed.docx

Doc Creator: tcuhawenimana11

Doc Date: 3/20/2023

Codes Applied: Types of information provided to boys and girls during puberty

Excerpt Creator: tcuhawenimana11

Excerpt Created On: 3/21/2023

Excerpt Range: 21793-22041

Eeh buriya usanga kenshi na kenshi ari abahungu kuko nibo bakunda gutinyuka kuba babaza yeah abakobwa bo bakunda kuba bitinyira ariko abahungu bakunda kuba bisanzura bakaba bavuga ibintu byose ntasoni bafite ntaki ariko abakobwa bo baritinya cyane.

Title: Mukarange Boys Trnscribed.docx

Doc Creator: tcuhawenimana11

Doc Date: 3/20/2023

Codes Applied: Boys have much information about SRH

Excerpt Creator: tcuhawenimana11

Excerpt Created On: 3/21/2023

Excerpt Range: 21793-22796

Eeh buriya usanga kenshi na kenshi ari abahungu kuko nibo bakunda gutinyuka kuba babaza yeah abakobwa bo bakunda kuba bitinyira ariko abahungu bakunda kuba bisanzura bakaba bavuga ibintu byose ntasoni bafite ntaki ariko abakobwa bo baritinya cyane.

Impamvu navuga ko ari abahungu cyane cyane abahungu bageze mur icyo kigero iyo afite nk inshuti ze akenshi bo bakunda kubwirana ibintu byabo byinshi, ashobora no kumubaza bitewe nuko abyumva undi akamuha ingero z uko abyumva n undi akamuha ize, bakagira amakuru menshi, ariko umukobwa we ashobora no kujya nko mumihango ahubwo akumva yaciye igikuba agatinya no kuba yanabibwira mugenzi we ko ari kuyijyamwo, kwakundi kwitinya kwanga gutanga amakuru yabo, ugasanga bafie amakuru makeya ahubwo abahungu aribo bafite menshi.

Njyewe numva ko abakobwa n abahungu bose banganya gutinyuka kuko bose abahungu bagira igihe bahura bakaganira kuri ibyo n abakombwa kandi barabigira kuko nta kuntu umukobwa yabaho bataganira n abandi k ubuzima bw imyororokere bwabo.

Title: Mukarange Boys Trnscribed.docx

Doc Creator: tcuhawenimana11

Doc Date: 3/20/2023

Codes Applied: Perceptions about the use of condom during sex

Excerpt Creator: tcuhawenimana11

Excerpt Created On: 3/21/2023

Excerpt Range: 22994-23692

iyo bigenze gutyo ugakoresha agakingirizo aho ngaho ntabwo aba ari amaburakindi, ahubwo ni ukugirango wirinde nawe yirinde kuko iyo mwageze kuri iyo tap akenshi n kenshi umuhungu bakunda kwisama basandaye cyangwa se abakobwa ntabwo cheri wawe mwaba mukundana aribwo mutangiye gukundana ngo uhite wihutira kumubwira ngo mwipimishe ushobora kubikora nkanjye urugero rwihuse ntabwo twaba dukundana nanjye nko mpite nkubwira ngo twipimishe ahubwo mu rwego rwo kugira ngo nirinde nawe nkurinde, wenda njyewe sinatwita ariko wowe watwita, na none njyewe nakwandura cyangwa se nkanakwanduza ariko mpita nkoresha agakingirizo m urwego rwo kugira ngo twirinde twese dukomeze kandi dukundanye tumeranye neza.

Title: Mukarange Boys Trnscribed.docx

Doc Creator: tcuhawenimana11

Doc Date: 3/20/2023

Codes Applied: How boys experience physiological changes that occured to them during puberty

Excerpt Creator: tcuhawenimana11

Excerpt Created On: 3/21/2023

Excerpt Range: 23812-24160

Ku imyaka yacu usanga umuhungu kur iki gihe usigaye ageza 15,16,17 akumva arashaka imibonano mpuzabitsina kandi iyo myaka abahungu benshi baba baba iwabo kandi ari wa wundi ukirerwa, na none ntabwo wavuga ngo ugiye gukorera ahongaho uharanire ko mwabyara nawe ubwawe utarabasha kwirera ngo wiyumve yuko wafata inshingano zo kurera umugore n umwana.

Title: Mukarange Boys Trnscribed.docx

Doc Creator: tcuhawenimana11

Doc Date: 3/20/2023

Codes Applied: Perceptions about the use of condom during sex

Excerpt Creator: tcuhawenimana11

Excerpt Created On: 3/21/2023

Excerpt Range: 24363-25072

Yego yaripimishije, akubwirako mwaganiriye byose ati ntakibazo narikingiye sinatwita?

Nanone bitewe n uko abakobwa akenshi na kenshi baba bafite abahungu benshi babatereta, ntabwo namwizera kimwe n uko n abahungu baba bafite abakobwa benshi batereta, kuko usanga umukobwa umwe ateretwa n abahungu barenze icumi ugasanga umuhungu umwe nawe aratereta abarenga icumi, yego mu kigero cyacu, mu rwego rwo kuvuga ngo nanyanga nzahite mbona undi dukundana bitangoye cyangwa ngo bintere ikibazo muri njyewe, rero ntabwo namwizera, ningombwa ngo dukoreshe kagakingirizo abyanze ubwongubwo ntitaye k umuriro naba mfite cyangwa se uburyo naba merewemwo mpita niyumvisha ko harikindi kintu ashaka kunshyiramwo ntarimfite.

Title: Mukarange Boys Trnscribed.docx

Doc Creator: tcuhawenimana11

Doc Date: 3/20/2023

Codes Applied: Purpose of friendship between a boy and a girl during adolescence

Excerpt Creator: tcuhawenimana11

Excerpt Created On: 3/21/2023

Excerpt Range: 25257-25585

Kuri ik igihe cyacu mu myaka yanjye, urugero nkanjye mfite imyaka 17, intego yo gukundana sinakubeshya kuko ntabwo navuga ngo mfite imyaka 17 ngo mu imyaka 25 nibwo nzaba mvuye nko murugo ngo nagire gupanga ubuzima bwanjye bushyashya ngo mpite numvako nazanamwo umugore ntaragira icyo ngeraho nubundi ntayindi ntego n imibonano.

Title: Mukarange Boys Trnscribed.docx

Doc Creator: tcuhawenimana11

Doc Date: 3/20/2023

Codes Applied: Precautions to take during puberty for boys

Excerpt Creator: tcuhawenimana11

Excerpt Created On: 3/21/2023

Excerpt Range: 26139-26281

Ingaruka z imibonano mpuzabitsina, nizo tuba tuzi har iziba ari nyeya cyane ko umuntu kubwe ntabwo aba yakabaye succesfied mubyo we atekereza.

Title: Mukarange Boys Trnscribed.docx

Doc Creator: tcuhawenimana11

Doc Date: 3/20/2023

Codes Applied: Suggestions to improve SRH during puberty and adolescence by boys

Excerpt Creator: tcuhawenimana11

Excerpt Created On: 3/21/2023

Excerpt Range: 26305-27488

nk amakuru mba mbona umuntu w umuhungu aba agomba gukenera, wenda nshobora nko kwibaza ikibazo niba ryamanye n uwo mukobwa mba ngomba kumenya ngo ese n ryari uwo mukobwa ashobora kuba yatwara inda cyangwa se niryari umukobwa ashobora kuba ari mugihe cyo kuba atatwara inda kuko tujya twigako hari nk iminsi umukobwa ashobora kuba yamara avuye m igihe cye k imihango, bakatubwirako icy gihe umukobwa kuberako igi riba riri kucyuri rishobora kuba riba ritagakuze, uwo mukobwa mushobora kuryamana akaba atatwara inda rero urumvako ibyo byose tuba tugomba kubimenya, tukamenyako ushobora no kubaza umukobwa wenda igihe amaze avuye m mihango, ukaba wamenyako igihe cyo gusama inda byoroshye kegereje ukaba wakihangana agakingirizo ntugakoreshe kuko n ubundi agakingirizo kugakoresha n akibanze ariko ntabwo bivuze ko utamutera inda kuko agakingirizo amahirwe yako ninka 50 k ijana byo kuba wamutera inda, ntekerezako unshobora no kuba ukambaye wenda ukaba wanakambaye nabi cyangwa se kakaba kanapfumuka ntubimenye wowe ukizerango wakambaye kandi ubwo wabirangije, rero uba ugomba kumenya igihe cyose n uwo mukobwa mugiye kuba mwabikorana agezemwo kugirango umenyeko wakwirinda, murakoze.

Title: Mukarange Boys Trnscribed.docx

Doc Creator: tcuhawenimana11

Doc Date: 3/20/2023

Codes Applied: Information that boys need

Excerpt Creator: tcuhawenimana11

Excerpt Created On: 3/21/2023

Excerpt Range: 27550-27621

n ukwibukako iyo agiye muri icyo gikorwa agomba gukoresha agakingirizo,

Title: Mukarange Boys Trnscribed.docx

Doc Creator: tcuhawenimana11

Doc Date: 3/20/2023

Codes Applied: Information that boys need

Excerpt Creator: tcuhawenimana11

Excerpt Created On: 3/21/2023

Excerpt Range: 27726-28037

Amakuru nkeneye nuko mu igihe naba ngiye kujya mur icyogikorwa ngomba kumenyako agakingirizo gakenewe cyangwa kuko iyo utagakoresheje uba uri kwishyira m ibibazo niy nshuti yawe namwe muba mukekako ushobora kumutera inda, ugasanga havutsemwo uwo mwana kandi namwe muba mukirerwa, nayo makuru numva naba nyeneye.

Title: Mukarange Boys Trnscribed.docx

Doc Creator: tcuhawenimana11

Doc Date: 3/20/2023

Codes Applied: Information that boys need

Excerpt Creator: tcuhawenimana11

Excerpt Created On: 3/21/2023

Excerpt Range: 28233-28323

Yego, n ukumenya uko wagakoresha, wowe ushobora kujya muri icyo gikorwa ukagakoresha nabi.

Title: Mukarange Boys Trnscribed.docx

Doc Creator: tcuhawenimana11

Doc Date: 3/20/2023

Codes Applied: Information that boys need

Excerpt Creator: tcuhawenimana11

Excerpt Created On: 3/21/2023

Excerpt Range: 28349-28821

ikindi numva namenya kur ibyo ng ibyo nuko wenda hari uko bavuga ngo umuntu ashobora gukora imibonano mpuzabitsina akajya kwa muganga hataracamwo hatarashira amasaha 72 bakaba bamuha imiti, ese njyewe ubundi ndayikoze nibyo wenda sinzi ngo n uwo muntu yanduye, andi makuru namenya nuko wenda harimwo bamwe umunt abibwira ukumva we ntanubwo yabyiyumvisha, ese wenda wampa ibini bituma ntasama cyangwa ntandura virusi itera sida? Noneho aho ngaho nyine ugasobanurirwa neza,

Title: Mukarange Boys Trnscribed.docx

Doc Creator: tcuhawenimana11

Doc Date: 3/20/2023

Codes Applied: Information that boys need

Excerpt Creator: tcuhawenimana11

Excerpt Created On: 3/21/2023

Excerpt Range: 29056-29677

Amakuru numva kuri uwo ng uwo utangiye kwinjira m ubugimbi twamuha, icyambere n mbere n inama nyinshi cyane zijyanye n kwirinda n uburyo binamunaniye kwirinda yabigenza, ukamurinda nanone ukamugira inama zijyanye no kwirinda ibigare by abahungu bakuze kuko abahungu bakuze usanga akenshi aribo bararura abakiri batoya, yego ukamugira inama nanone kubijyanye no kwirinda abakobwa kuko ntabwo wakwirinda abo bahungu bakuze ngo umukobwa we abure kugushuka kuko usanga nk abo ngabo aba aribwo bari kwinjira m ubugimbi abakobwa bakuze babashuka bakabafatirana bakabashora mur iyo mibonano mpuzabitsina itanakingiye, murakoze.

Title: Mukarange Boys Trnscribed.docx

Doc Creator: tcuhawenimana11

Doc Date: 3/20/2023

Codes Applied: Information that boys need

Excerpt Creator: tcuhawenimana11

Excerpt Created On: 3/21/2023

Excerpt Range: 29679-30303

Nibyo nyine har ukuntu nk umukobwa ashobora kukubona akumva aragukunze arakwifuje kuko uko umuhungunyakwifuza umukobwa ninako n umukobwa yakwifuza umuhungu wenda nuko ashobora kugira ubwoba akabitinya, benshi babitnya kuba banabivuga ariko nawe ashobora kugushuka, nka kuriya wenda usanga umwana wamusize murugo agejeje imyaka 13,14 aho ngaho wenda yasigaranye n umukozi ufite imyaka makumyabiri ningahe ashobora kumushuka bitewe nawe n ikibazo yahuye nacyo, birashoboka ko ashobora kumushuka ugasanga amushoye muriyo mibonano mpuzabitsina kandi ntan ikintu abiziho ngo wenda ngo abashe kuba yakwicyingira nuko njye mbyumva.

Title: Mukarange Boys Trnscribed.docx

Doc Creator: tcuhawenimana11

Doc Date: 3/20/2023

Codes Applied: Obstacles and risks experienced by boys during the sexual and reproductive health changes occuring during puberty and adolescence

Excerpt Creator: tcuhawenimana11

Excerpt Created On: 3/21/2023

Excerpt Range: 30396-30464

Bibaho cyane kenshi ahubwo nta nubwo kaba ari gakeya ntabwo byabura.

Title: Mukarange Boys Trnscribed.docx

Doc Creator: tcuhawenimana11

Doc Date: 3/20/2023

Codes Applied: Obstacles and risks experienced by boys during the sexual and reproductive health changes occuring during puberty and adolescence

Excerpt Creator: tcuhawenimana11

Excerpt Created On: 3/21/2023

Excerpt Range: 30618-30730

Nabyo bibaho cyane, uko wenda nk umukobwa mwaba muri nko mukigero kimwe yagushuka cyangwa umuhungu se yagushuka.

Title: Mukarange Boys Trnscribed.docx

Doc Creator: tcuhawenimana11

Doc Date: 3/20/2023

Codes Applied: Obstacles and risks experienced by boys during the sexual and reproductive health changes occuring during puberty and adolescence

Excerpt Creator: tcuhawenimana11

Excerpt Created On: 3/21/2023

Excerpt Range: 30783-31681

Murakoze, ukuntu bibaho, bibaho ko umuhungu har igihe ashobora gushuka umuhungu mugenzi we, wenda twebwe nyine abahungu bameze gutyo tubita ngo n abaGe abatinganyi bashobora kugushuka bitewe nuko we muriwe ntabwo nyine akunda abakobwa pee hanyuma akaza ubonako ar umuhungu mukagenda kuberako ar iniga yawe mukagenda mukajya gusangira wenda niba anyway nkizo nzoga cyangwa atazinywa mukajya gusangira ugasanga m inzoga cyawe cyangwa icyokunywa cyawe niyo yaba ari niyo jui hari ibindi bintu ashyizemwo, ibyo bintu ashyizemwo bikaba byagutera wenda nk ikibazo ukaba wenda wasinzira cyangwa wahwera agahita agufatirana nk uko nguko kuberako we bitewe nk ukuntu ameze, ntabwo uko ameze akunda abakobwa nyine kuberako ar umuhungu mugenzi wawe nawe aba ashaka umuhungu mugenzi we kugirango aryamane, we yagushka kuberako we abikubwiye ntabwo wabyemera, we yagushuka rero birashoboka ibyo binabaho cyane.

Title: Mukarange Boys Trnscribed.docx

Doc Creator: tcuhawenimana11

Doc Date: 3/20/2023

Codes Applied: Precautions to take during puberty for boys Received information is not detailed (mixed)

Excerpt Creator: tcuhawenimana11

Excerpt Created On: 3/21/2023

Excerpt Range: 31909-32606

Eeeh njyewe reka mvuge kuri njyewe amakuru ntabwo nigeze wenda nyahabwa n umubyeyi kuko ntabwo nagiye ngo mbwire mama ngo mubwire ahubwo nayakuye mubasore bagenzi banjye cyangwa abahungu bagenzi banjye cyangwa n abagabo banduta, nibo bagiye bayampa bakambwira ngo ati niba ugiye kuryamana n ako kana koresha ubwirinzi cyangwa akakubdwira wenda umuhungu mugenzi wawe akakubwira ati aho kugirango muryamane gutyo, gutyo nyine amakuru nk ayo ngayo tuba twarahawe har igihe wenda akubwiye umuntu w umuhungu mugenzi wawe akubwiye ngo kugirango utazamutera inda wenda uzabanze umenye igihe agiye mumihangon ighe ayivuyemwo kugirango wowe uzafatirane hagati aho ngaho kugirango utazagira ikibazo uteza.

Title: Mukarange Boys Trnscribed.docx

Doc Creator: tcuhawenimana11

Doc Date: 3/20/2023

Codes Applied: Sources of information received during puberty

Excerpt Creator: tcuhawenimana11

Excerpt Created On: 3/21/2023

Excerpt Range: 32621-32744

amakuru njyewe mfite nagiye nyakura m inshuti zanjye abandi bahungu bagenzi banjye nisanzuraho dukunda kuganira buri munsi.

Title: Mukarange Boys Trnscribed.docx

Doc Creator: tcuhawenimana11

Doc Date: 3/20/2023

Codes Applied: Sources of information received during puberty

Excerpt Creator: tcuhawenimana11

Excerpt Created On: 3/21/2023

Excerpt Range: 32878-32973

amakuru mfite k ubuzima bw imyororokere amwe nayakuye k umubyeyi wanjye andi nyakura mu ishuri.

Title: Mukarange Boys Trnscribed.docx

Doc Creator: tcuhawenimana11

Doc Date: 3/20/2023

Codes Applied: Sources of information received during puberty

Excerpt Creator: tcuhawenimana11

Excerpt Created On: 3/21/2023

Excerpt Range: 32974-33283

Nk amakuru mfite nagiye nyakura kuri bakuru banjye kuko mfite bakuru banjye bakuru bari gusoza n amashuri, har ukuntu aza akakubwira ati rero murumuna wanjye ndabona uri kumwe n udukobwwa inshuro nyinshi cyane, agatangira kugenda akugiramwo inama ugasanga amakuru niho uyakuye kuko aba afite byinshi akurusha.

Title: Mukarange Boys Trnscribed.docx

Doc Creator: tcuhawenimana11

Doc Date: 3/20/2023

Codes Applied: Precautions to take during puberty for boys

Excerpt Creator: tcuhawenimana11

Excerpt Created On: 3/21/2023

Excerpt Range: 33857-35267

Inama z abahungu bagenzi bawe cyangwa inshuti zawe zo zirayikuira cyane zikakubwira ngo kariya kana nimba muri inshuti genda ugafatirane hakiri kare, bakakugiramwo inama rwose bakakubwira ati katazagucika cyangwa nka mukuru wanjye nkanjye eeh nawe inama nkizo yagiye azingira nk izo ngizo.

Murakoze ndi nimero 4, njye amakuru menshi ahantu nagiye nyakura ku ikigo cyacu, nk iki kigo dukunda gusurwa cyane uko nyeka ntabwo hacaho ukwezi tutagize abashyitsi bo kutwigisha ku ibintu by imyororokere tugakora essemble twese hamwe, byaba umuyobozi w ikigo nawe ubwe afata igihe akatwigisha n abalimu bakadukoramwo amatsinda nyine abahungu, abalimu b abagabo bagatwara twe abahungu, ab abamama bagatwara abakobwa nyine, twese tukiga bakatubwira, twavayo n ubundi ariko nihahandi turongera tukihuza tukaganira kubyo batumbwiye kuko buri muntu aba afite amatsiko yo kumenya ngo uriya ko bamubwiye ngo ajye ukwe nanjye jye ukwanjye baganiriye ibiki? Ubwo nyine umukobwa akakubwira ibyo bamubwiye wenda wasanga harih ahantu bakubangamiye ukavuga uti oya hariya babangamye, natwe rero har ibyo baba batubwiye nkatwe wenda tukamubwira ati dore natwe n uku batubwiye, aho niho genda nkura amakuru nyine nk abayobozi basanzwe nka disipi ajya akunda kuza bakaganira, akadukoresha inama, har ubwo tuganira twese turi hamwe hakaba n ubwo tuganira tutari hamwe bitewe ubwo n ingingo turi buganireho, murakoze njye naho nyakura.

Title: Mukarange Boys Trnscribed.docx

Doc Creator: tcuhawenimana11

Doc Date: 3/20/2023

Codes Applied: Sources of information received during puberty

Excerpt Creator: tcuhawenimana11

Excerpt Created On: 3/21/2023

Excerpt Range: 35305-35539

amakuru njyewe ayambere nayabwiwe n bakuru banjye, eeh ahandi umuntu ayakura ni kubitangazamakuru, kuri radiyo birirwa babyigisha n tv birirwa babyigisha ahandi n hano ku ishuri aribyo uyu muvandimwe yavugaga tugira ibiganiro byinshi…

Title: Mukarange Boys Trnscribed.docx

Doc Creator: tcuhawenimana11

Doc Date: 3/20/2023

Codes Applied: Sources of information received during puberty

Excerpt Creator: tcuhawenimana11

Excerpt Created On: 3/21/2023

Excerpt Range: 35760-35839

Ubundi abaduha amakuru, umuntu ajya kuguha amakuru bitewe n ukuntu aba akubona.

Title: Mukarange Boys Trnscribed.docx

Doc Creator: tcuhawenimana11

Doc Date: 3/20/2023

Codes Applied: How to take care of themselves when phsyiological changes occur for boys

Excerpt Creator: tcuhawenimana11

Excerpt Created On: 3/21/2023

Excerpt Range: 35922-36311

Iyo wumvise biryoshye urayumva cyane nko kuri radiyo cyangwa television niho uba wumvako amakuru bashobora kuguha ariyo y ukuri, ahandi ni hano ku ishuri narindimwo mvuga cyane ko ibyo biganiro batubwira batugira inama badushyira muri group amaclub atandukanye niho umuntu agenda akura amakuru no mubitangazamakuru nyine na youtube umuntu ashobora kuyakurayo n izindi mbuga nkoranyambaga.

Title: Mukarange Boys Trnscribed.docx

Doc Creator: tcuhawenimana11

Doc Date: 3/20/2023

Codes Applied: Sources of information received during puberty

Excerpt Creator: tcuhawenimana11

Excerpt Created On: 3/21/2023

Excerpt Range: 36342-36690

amakuru njyewe nayabonye impande zombie, k uruhande runshuka n urugira inama kuko nanone iyo batagushutse ngo uve m inzira y ukuru ntabwo ubasha kugaruka muri yanzira, akarusho nka bagenzi banjye kuko har igihe akanshi bagenzi banjye twakunze kugendana nabo n abantu nyine bakunda kunywera inzoga cyane eeh b abajama n amagenge nyine muri make yego

Title: Mukarange Boys Trnscribed.docx

Doc Creator: tcuhawenimana11

Doc Date: 3/20/2023

Codes Applied: Received information is misleading and risky

Excerpt Creator: tcuhawenimana11

Excerpt Created On: 3/21/2023

Excerpt Range: 36692-37532

arakubwira ati sha kurya umwana nibyiza biruhura m umutwe bigabanya stress akakumvisha ibyiza byabyo ntakumvishe ibibi, ariko nanone iyo ugeze k umubyeyi cyangwa se undi muntu akumvisha ibibi bya byabindi bakubwiye noneho nawe waguma mugihirahiro ukabura icyo ufata n icyo ureka nibwo ufata umwanzuro ukajya kuri wawundi ukoshya kuko ntabwo wahita wihutira kujya kuri wawundi ukubwiza ukuri kuko akenshi na kenshi dukunda kubanza kumva ikinyoma kurenza kumva ukuru, iyo uvuye muri byabinyoma wenda tuvuge nk umuntu wenda ubikoze bwambere ntabwo yashobora kumva ibyiza byabyo cyangwa se ibibi byabyo ariko iyo avuyeyo amaze kubikora ashobora kuza noneho agatega amatwi wawundi umugira inama nziza kurenza wawundi umugira inama mbi so nyine dukwiye kubanza kumenyako ko akenshi ukuri ariko kwambere nanone kandi kumva ibintu by abantu bakuru.

Title: Mukarange Boys Trnscribed.docx

Doc Creator: tcuhawenimana11

Doc Date: 3/20/2023

Codes Applied: Sources of information received during puberty

Excerpt Creator: tcuhawenimana11

Excerpt Created On: 3/21/2023

Excerpt Range: 37608-37681

noneho njye amakuru nayakuye murugo ndetse no ku nshuti zanjye tugendana.

Title: Mukarange Boys Trnscribed.docx

Doc Creator: tcuhawenimana11

Doc Date: 3/20/2023

Codes Applied: Sources of information received during puberty

Excerpt Creator: tcuhawenimana11

Excerpt Created On: 3/21/2023

Excerpt Range: 37714-37858

Ni mama njyewe yampaye amakuru noneho amakuru ampaye nayasanisha nayo nakuye mu nshuti ngasanga si bimwe noneho njyewe nkajyenda ngatega amatwi.

Title: Mukarange Boys Trnscribed.docx

Doc Creator: tcuhawenimana11

Doc Date: 3/20/2023

Codes Applied: Received information is right and provides sufficient information helping boys and girls during puberty

Excerpt Creator: tcuhawenimana11

Excerpt Created On: 3/21/2023

Excerpt Range: 37908-37998

Njyewe ubundi yambwiye nakwirinda gukora imibonano mpuzabitsina ko nakwanduriramo indwara.

Title: Mukarange Boys Trnscribed.docx

Doc Creator: tcuhawenimana11

Doc Date: 3/20/2023

Codes Applied: Received information is right and provides sufficient information helping boys and girls during puberty

Excerpt Creator: tcuhawenimana11

Excerpt Created On: 3/21/2023

Excerpt Range: 38013-38433

Ambwira n ukundi nyine biriya bagenda bavuga wenda ngo iyo muryamanye ngo akira ibishishi akambwira ati ibyo baragushuka wowe rindira igihe, noneho mu nshuti zanjye bakambwira bati uriya mwana woe umurebe neza noneho iyo bakubwiye ibyo hari igihe bo baba barabikoze bikabagiraho ingaruka noneho nabo bagashaka kukwanduza ibiri mu mitwe yabo bagashaka kubigushyiramo bakagushora nyine muri izo ngeso murakoze.

Title: Mukarange Boys Trnscribed.docx

Doc Creator: tcuhawenimana11

Doc Date: 3/20/2023

Codes Applied: Sources of information received during puberty

Excerpt Creator: tcuhawenimana11

Excerpt Created On: 3/21/2023

Excerpt Range: 38458-38586

amakuru njyewe nayakuye m inshuti zanjye no ku ishuri, m inshuti zanjye hari abambwiraga amakuru meza abandi bakambwira atariyo.

Title: Mukarange Boys Trnscribed.docx

Doc Creator: tcuhawenimana11

Doc Date: 3/20/2023

Codes Applied: Received information is misleading and risky

Excerpt Creator: tcuhawenimana11

Excerpt Created On: 3/21/2023

Excerpt Range: 38695-39194

Bambwiraga ubundi ngo gukora imibonano mpuzabitsina, bakakubwira ati uriya mukobwa nyine ngo aragukunda ariko ngo nuko wowe wigira ipamba akakumbwira ati nyine ngo ufata ihene ngo ayifata igihebeba, akakubwira ati rero rya umwana ibindi uzaruburana ugarutse ariko usaganga nabandi bakakubwira ati iyo mico ntabwo ari myiza wowe niba ukunze nk umuntu nyine sumushyire muriyo mibonano mpuzabitsina no ku ishuri bakakubwira ati ubundi ngo niba bikunaniye ngo koresha agakingirizo cyangwa ngo ubyirinde.

Title: Mukarange Boys Trnscribed.docx

Doc Creator: tcuhawenimana11

Doc Date: 3/20/2023

Codes Applied: Sources of information received during puberty

Excerpt Creator: tcuhawenimana11

Excerpt Created On: 3/21/2023

Excerpt Range: 39353-39376

Ku ikigo cy urubyiruko.

Title: Mukarange Boys Trnscribed.docx

Doc Creator: tcuhawenimana11

Doc Date: 3/20/2023

Codes Applied: Sources of information received during puberty

Excerpt Creator: tcuhawenimana11

Excerpt Created On: 3/21/2023

Excerpt Range: 39673-39686

No ku ishuri.

Title: Mukarange Boys Trnscribed.docx

Doc Creator: tcuhawenimana11

Doc Date: 3/20/2023

Codes Applied: Sources of information received during puberty

Excerpt Creator: tcuhawenimana11

Excerpt Created On: 3/21/2023

Excerpt Range: 39715-39768

ahantu nakuye amakuru n umubyeyi wayampaye w umumama

Title: Mukarange Boys Trnscribed.docx

Doc Creator: tcuhawenimana11

Doc Date: 3/20/2023

Codes Applied: Received information is right and provides sufficient information helping boys and girls during puberty

Excerpt Creator: tcuhawenimana11

Excerpt Created On: 3/21/2023

Excerpt Range: 39716-40185

ahantu nakuye amakuru n umubyeyi wayampaye w umumama, yarambwiye nti ushobora gukora imibonano mpuzabitsina ukiri muto nawe utarakura nyine nkikurera ugasanga wenda wanduriyemwo indwara cyangwa ubyaye umwana bikaba ngombwa ko uturushya kandi nawe iyo ubonye wateye umukobwa inda wumva mumutwe nawe utazi ukuntu umeze wumva utikontorora neza, ikindi baravuze ati ushobora gukora imibonano mpuzabitsina ukaba wanafungwa kandi ubona ukiri mutoya ukaba wiyangirije ubuzima.

Title: Mukarange Boys Trnscribed.docx

Doc Creator: tcuhawenimana11

Doc Date: 3/20/2023

Codes Applied: Received information is right and provides sufficient information helping boys and girls during puberty

Excerpt Creator: tcuhawenimana11

Excerpt Created On: 3/21/2023

Excerpt Range: 40373-40564

Mama erega nyine niwe uba wisanzuraho kuko papa har igihe aba adahari aza nijoro ukaba utabona umwanya wo kumuganiriza ariko mama kuberako mwirindwana uba wanamuganiriza mukaba inshuti cyane.

Title: Mukarange Boys Trnscribed.docx

Doc Creator: tcuhawenimana11

Doc Date: 3/20/2023

Codes Applied: Sources of information received during puberty

Excerpt Creator: tcuhawenimana11

Excerpt Created On: 3/21/2023

Excerpt Range: 40565-40798

Impamvu umubyeyi w umumama ariwe ukunda ubundi kutugira inama har igihe nk umubyeyi w umupapa ubimubwira akakuka inabi noneho wowe ukagira ubwoba ukamutinya kubimubaza kandi umubyeyi w umumama iyo ubimubajije ntakibazo akubwira neza.

Title: Mukarange Boys Trnscribed.docx

Doc Creator: tcuhawenimana11

Doc Date: 3/20/2023

Codes Applied: Sources of information received during puberty

Excerpt Creator: tcuhawenimana11

Excerpt Created On: 3/21/2023

Excerpt Range: 40799-41417

Murakoze, ndi nimero 9 akenshi na kenshi impamvu abana b abahungu bisanzura kubabyeyi ba abamama nuko mama wawe nibyose aho kugirango ubure mama wawe, wowe wakwibona urimwo uribura kuko umubyeyi w umumama ntabwo watekereza umuntu wagutwise amezi 9 akugendana mu inda wavuka akakumesera winereye winyariye akakwitaho atakutse inabi ngo uzavuge yuko wamwanga, naho akenshi ba papa bacu wowe unamubajije yanagushuka, impamvu mvuga yuko yanagushuka kuko har igihe ababyeyi nanone babana batarabanje gukundanaho, ukazajya uhora wumva papa wawe arimo yuka inabi mama wawe ibyo rero bigatuma wamwana w umuhungu yanga papa we.

Title: Mukarange Boys Trnscribed.docx

Doc Creator: tcuhawenimana11

Doc Date: 3/20/2023

Codes Applied: Sources of information received during puberty

Excerpt Creator: tcuhawenimana11

Excerpt Created On: 3/21/2023

Excerpt Range: 41491-41947

Nk ubu nkanjye papa ntanubwo nanatinyuka sinzi nibaa nanatinyuka n kugira ikintu mubaza, njyewe mvugana na mama akenshi, yego ababyeyi b abamama umunsi k uwundi nibo bakurikirana abana babo b abahungu kimwe nuko ababyeyi b abagabo nabo umunsi k umunsi akurikirana umukobwa we yego akenshi usanga n iyo umubyeyi w umugabo abyaye umuhungu n umukobwa umuntu ufata care nyinshi cyane aba ari umukobwa sinzi ahubwo muzadukorera ubuvugizi murebe uburyo byagenda.

Title: Mukarange Boys Trnscribed.docx

Doc Creator: tcuhawenimana11

Doc Date: 3/20/2023

Codes Applied: Received information is right and provides sufficient information helping boys and girls during puberty

Excerpt Creator: tcuhawenimana11

Excerpt Created On: 3/21/2023

Excerpt Range: 41964-42900

impamvu tujya ku babyeyi b abadamu nka mama wawe papa wawe akunda wenda nimba yagiye mu kazi akaza atinze ,akaza nka nimugoroba kandi we aba atinyitse usanga ari wa muntu udashaka kwamenyerwa n abana cyane akavuga ati uyu mwana ibintu atangiye kuzana ni n imico mibi , ku buryo ushobora no kubivuga ushaka kubimubaza ugasanga uriye nk inkoni zirenga nyinhi cyane ukavuga ati rero mama njya mubwira wenda agashiduka akancyaha wenda n ubwo atahita ambwira nabi cyane wenda ashobora no kungira inama akavuga ati reka mbimubazeho, wabimubaza ho nibwo nibwo ushobora kumvaho gacyeya agahita agira ubwoba akavuga ati umwana wanjye atazagwa mu bishuko nk ibyo ngibyo agahita yihutira kubigusobanurira hakiri kare mu gihe wenda nk umubyeyi w umu papa ashobora kereba ati uyum mwana nimbimubwira ashobora kuzagira ikindi gihe akagenda agutinza akavuga ngo uracyari umwana kandi yagakwiye kubikubwira hakiri kare kugira ngo ukure ubizi.

Title: Mukarange Boys Trnscribed.docx

Doc Creator: tcuhawenimana11

Doc Date: 3/20/2023

Codes Applied: Sources of information received during puberty

Excerpt Creator: tcuhawenimana11

Excerpt Created On: 3/21/2023

Excerpt Range: 42924-43048

njyewe har icyo wenda namera nkaho nopozinga bagenzi banjye, ababyeyi bombi birashoboka ko baguha amakuru kuko tuvuge wenda.

Title: Mukarange Boys Trnscribed.docx

Doc Creator: tcuhawenimana11

Doc Date: 3/20/2023

Codes Applied: Sources of information received during puberty

Excerpt Creator: tcuhawenimana11

Excerpt Created On: 3/21/2023

Excerpt Range: 43074-43524

Kuri njyewe impamvu navuze ko amakuru nayahawe na mama, nageze igihe cyo kuyakenera papa adahari, kuberako birashoboka ko hari undi mugenzi wanjye nawe waba warabuze nyine cyangwa se yarakeneye amakuru akayahabwa na se bitewe n urukundo amufitiye, ariko impamvu njye mvuga ko umubyeyi w umumama ariwe wafashe iyambere ambwira ibyo ngomba kwirinda uko nitwara, nuko namenye ubwenge ariwe mbona, uwo data sinari muzi wenda ngo abe yampereza ayo makuru.

Title: Mukarange Boys Trnscribed.docx

Doc Creator: tcuhawenimana11

Doc Date: 3/20/2023

Codes Applied: Sources of information received during puberty

Excerpt Creator: tcuhawenimana11

Excerpt Created On: 3/21/2023

Excerpt Range: 43548-44409

amakuru njyewe akenshi na kenshi nayakuraga kuri radio no kumubyeyi wanjye mama, we yambwiye nyine ukuntu ushobora kuba wakwirinda wenda imibonano mpuzabitsina, usaganga wenda ugiye nko mu inshuti zawe zikwigishije ukuntu wabonamwo umukobwa wenda mwiza bakakubwirango gutya na gutya ngo fata ahubwo uri ikigoryi ati urimwo uratinda, ariko kenshi na kenshi njyewe iyo ndi murungano rwanjye nkumva barimwo baragira inama ngo akenshi na kenshi zo kuba nanjya bashora nyine m ibintu by abakobwa, njyewe mbasha kuba naba nava muri icyo kigare nubundi akenshi na kenshi n abantu bakure njye nkunda gutega amatwi nkanumva ni kuri radio nikuri youtube na mama gutyo kuko papa ashobora kumpa amakuru asanzwe nyine izo ngeso azigira agasanga aguhaye nk amakuru wenda atariyo, kenshi na kenshi bitewe n igihe aba yaragutwise nk umwanya aba aguha bituma nyine muhuza cyane.

Title: Mukarange Boys Trnscribed.docx

Doc Creator: tcuhawenimana11

Doc Date: 3/20/2023

Codes Applied: Obstacles and risks experienced by boys during the sexual and reproductive health changes occuring during puberty and adolescence

Excerpt Creator: tcuhawenimana11

Excerpt Created On: 3/21/2023

Excerpt Range: 44610-44748

Ingaruka yambere n ugushukwa.

Ndi ngaruka cyangwa se ingorane ushobora guhura nazo ni ukubura mobilayizasiyo y ubwo bukangura mbaga nyine.

Title: Mukarange Boys Trnscribed.docx

Doc Creator: tcuhawenimana11

Doc Date: 3/20/2023

Codes Applied: Obstacles and risks experienced by boys during the sexual and reproductive health changes occuring during puberty and adolescence

Excerpt Creator: tcuhawenimana11

Excerpt Created On: 3/21/2023

Excerpt Range: 44790-45075

Ku ubuzima bw imyororokere, kuba ubwo bukangurambaga bushobora kutubera indi mbogamizi.

Ku abahungu ingaruka ziba mbere na mbere iyo utabyitwayemwo neza ushiduka wakuyemwo indwara zidakira ukaba wanavanamwo cyangwa se n urupfu kuko har igihe nk umuntu aguhemukira wanjya ubitekerezaho…

Title: Mukarange Boys Trnscribed.docx

Doc Creator: tcuhawenimana11

Doc Date: 3/20/2023

Codes Applied: Obstacles and risks experienced by boys during the sexual and reproductive health changes occuring during puberty and adolescence

Excerpt Creator: tcuhawenimana11

Excerpt Created On: 3/21/2023

Excerpt Range: 45131-46270

Yego, urugero nka sida,yego cyangwa se ukanakuramwo urupfu kuko ntabwo naba mporana intimba wenda, hari nk ukuntu bakureresha muri family ugasanga umwe muba member wa family niwe ukwifatiye, noneho iyo muhora muhura kenshi na kenshi yantimba irazuka bikaba byanakuviramwo y uko wamutima ukoshya wakubwirango iyahure cyangwa se wabiganirira ku inshuti yawe akenshi abahungu turaganira kandi abahungu ntitugira ibitekerezo bimwe, dushobora kuganira njyewe nkaba nakugira inama undi twaganira nanone njyewe nkamushuka, shobora kumushuka nkamubwira ati niba byaragenze gutyo genda kuri police umurege noneho nakumvako ngiye gusebya umuryango nkavuga nti reka nkemure ikibazo njyewe mveho, ugasanga njyewe ndiyahuye cyangwa se niyikishije imiti, ariko nyine ingaruka nyinshi cyane cyane harimwo no kubyara imburagihe ukabyara igihe cyawe kitaragera kuko nanone iyo uhuye nabagenzi bawe bakakubwirango ntukarire bombo mu ishashi ushiduka ya bombo yavuyemwo shikarete kandi iyo ivuyemwo shikarete, shikarete iragukwedukana, ugasanga yanda rero igukurikiranye ukiri mutoya barakujyanye baragufunze ubuzima bwawe ububayeho uri mubwigunge, murakoze.

Title: Mukarange Boys Trnscribed.docx

Doc Creator: tcuhawenimana11

Doc Date: 3/20/2023

Codes Applied: Obstacles and risks experienced by boys during the sexual and reproductive health changes occuring during puberty and adolescence

Excerpt Creator: tcuhawenimana11

Excerpt Created On: 3/21/2023

Excerpt Range: 46294-46560

ubundi ingauka abahungu bahura nazo, harimwo kwandura nka virus itera sida, indi ngaruka umuhungu ashobora kuba yakoze imibonano mpuzabitsina wenda n umugore umuruta cyangwa se umukobwa umuruta yamushutse noneho akamutera izo ndwara wa muhungu we agashaka kwiyahura.

Title: Mukarange Boys Trnscribed.docx

Doc Creator: tcuhawenimana11

Doc Date: 3/20/2023

Codes Applied: Obstacles and risks experienced by boys during the sexual and reproductive health changes occuring during puberty and adolescence

Excerpt Creator: tcuhawenimana11

Excerpt Created On: 3/21/2023

Excerpt Range: 46580-46795

izindi ngaruka abahungu dushobora guhura nazo n ukwiheba, noneho muri uko kwiheba bidushora mu iki mu biyobyabwenge, tukajya kunywa ibyo biyobyabwenge kugirango twiyibagize n akogahinda twagize twatewe n ibyo byose.

Title: Mukarange Boys Trnscribed.docx

Doc Creator: tcuhawenimana11

Doc Date: 3/20/2023

Codes Applied: Obstacles and risks experienced by boys during the sexual and reproductive health changes occuring during puberty and adolescence

Excerpt Creator: tcuhawenimana11

Excerpt Created On: 3/21/2023

Excerpt Range: 46806-47103

indi ngaruka yahura nayo n ukwiyicira ejo hazaza hawe, har igihe wanjya muri izo ngeso ugasanga uteye umukobwa inda, ugasanga y amashuri wigaga urayasubitse ubwo ugiye gufata iyindi ngero yo gutunga umugore n umwana kandi namwe mukiri abana, urumva ejo hawe hazaza ba uhishe hakiri kare, murakoze.

Title: Mukarange Boys Trnscribed.docx

Doc Creator: tcuhawenimana11

Doc Date: 3/20/2023

Codes Applied: Obstacles and risks experienced by girls during sexual and reproductive health changes occuring during puberty and adolescence

Excerpt Creator: tcuhawenimana11

Excerpt Created On: 3/21/2023

Excerpt Range: 47104-47354

Indi ngaruka n ukubyara utarageza igihe nyine ugatangira kurera umwana kandi nawe ukiri umwana.

Murakoze ndi nimero 6, ingaruka ziriho ushobora gukura mu mibonano mpuzabitsina zeze ubu ngubu, ni ugufuga kuko leta imibonano idakingiye leta ntibyemera.

Title: Mukarange Boys Trnscribed.docx

Doc Creator: tcuhawenimana11

Doc Date: 3/20/2023

Codes Applied: Obstacles and risks experienced by girls during sexual and reproductive health changes occuring during puberty and adolescence

Excerpt Creator: tcuhawenimana11

Excerpt Created On: 3/21/2023

Excerpt Range: 47597-47974

Ingorane bahura nazo, ni nko gushukwa, gukoreshwa ibyo batari bateganije, bishobora no kubaviramo ingaruka zabicira ejo hazaza. Gukoreshwa ibyo atateganije ni ukuntu wenda umukobwa ashobora nko kujya gusaba akazi ahantu manager akamubwira ati kugira ngo nguhe akazi turabanza turyamane. Bitewe na profit ari kumva imbere akabikora bya mwikize kugira ngo abashe kubona ako kazi.

Title: Mukarange Boys Trnscribed.docx

Doc Creator: tcuhawenimana11

Doc Date: 3/20/2023

Codes Applied: Obstacles and risks experienced by girls during sexual and reproductive health changes occuring during puberty and adolescence

Excerpt Creator: tcuhawenimana11

Excerpt Created On: 3/21/2023

Excerpt Range: 47975-48524

Ingora ne ahura nazo, iya mbere ni ukubyara imbura gihe kandi muri uko kubyara imburagihe, just nawe urabyumva umwana wawe aba abyaye imbura gihe nta kintu uba ukimukorera uba wumva ari nk ikinani nyine kiri aho ngaho cyananiranye nk ikirara cyabuze aho kijya ashobora no kumubwira ati mvira aha ngaha nawe ujye niba wahisemo kubyara ushoboye gutunga urugo. So iyo umukuye iwawe akajyenda ajya ku muhanda kandi kujya kuri uwo muhanda ashobora kuba indara muri uko kuba indara aghinduka indaya ni kwa kundi nyine police imufata ikamujyana muri kasho.

Title: Mukarange Boys Trnscribed.docx

Doc Creator: tcuhawenimana11

Doc Date: 3/20/2023

Codes Applied: Obstacles and risks experienced by girls during sexual and reproductive health changes occuring during puberty and adolescence

Excerpt Creator: tcuhawenimana11

Excerpt Created On: 3/21/2023

Excerpt Range: 48541-48805

ok ndi nimero 9 izindi ngaruka abana b abakoba bahura nazo zimwe ni uko aba atakaje ejo hazaza hiwe heza ikindi bimutera kwiheba ikindi kandi n igihugu nacyo kiba gihombye umuyobozi w ejo cyangwa se undi muntu ukomeye uzagirira igihugu akamaro ejo hazaza murakoze.

Title: Mukarange Boys Trnscribed.docx

Doc Creator: tcuhawenimana11

Doc Date: 3/20/2023

Codes Applied: Obstacles and risks experienced by girls during sexual and reproductive health changes occuring during puberty and adolescence

Excerpt Creator: tcuhawenimana11

Excerpt Created On: 3/21/2023

Excerpt Range: 48806-49131

Indi ngaruka ubundi umukobwa bashobora gusama inda itateganijwe akabihisha ubundi ugasanga bimuviriyemo n urupfu. Ashobora kubihishaubwo nyine yarabihishe ni iwabo yarabibahishe, noneho igihe cyo kubyara cyagera we ugasanga atinye no kujya kwa muganga wenda apfuye abyara cyangwa se bamubwiye ibindi bintu by imiti bikamwica.

Title: Mukarange Boys Trnscribed.docx

Doc Creator: tcuhawenimana11

Doc Date: 3/20/2023

Codes Applied: Obstacles and risks experienced by girls during sexual and reproductive health changes occuring during puberty and adolescence

Excerpt Creator: tcuhawenimana11

Excerpt Created On: 3/21/2023

Excerpt Range: 49142-49334

izindi ngaruka umukobwa ahura nazo iyo atwaye inda mburagihe hari igihe aba atwite umwana ugasanga abyaye umwana upfuye cyangwa nawe agapfa ari kumubyara cyangwa akarwamo za fisitire murakoze.

Title: Mukarange Boys Trnscribed.docx

Doc Creator: tcuhawenimana11

Doc Date: 3/20/2023

Codes Applied: Obstacles and risks experienced by girls during sexual and reproductive health changes occuring during puberty and adolescence

Excerpt Creator: tcuhawenimana11

Excerpt Created On: 3/21/2023

Excerpt Range: 49335-49660

Ingaruka umukobwa ahura nazo umukobwa iyo atwaye inda bituma anasaza vuba mu bitekerezo kuko nta gihe aba yari yageza cyo gutunga umwana.

Indi ngaruka ashobora gukora imibonano mpuzabitsina yajya kwipimisha agasanga yaranduye kandiiwabo bamukanira cyane agakurizamo gutinda kubibabwira ahubwo akajya kwiyahura.

Yaranduye iki?

Title: Mukarange Boys Trnscribed.docx

Doc Creator: tcuhawenimana11

Doc Date: 3/20/2023

Codes Applied: Obstacles and risks experienced by girls during sexual and reproductive health changes occuring during puberty and adolescence

Excerpt Creator: tcuhawenimana11

Excerpt Created On: 3/21/2023

Excerpt Range: 49661-49678

Virusi itera sida

Title: Mukarange Boys Trnscribed.docx

Doc Creator: tcuhawenimana11

Doc Date: 3/20/2023

Codes Applied: Obstacles and risks experienced by girls during sexual and reproductive health changes occuring during puberty and adolescence

Excerpt Creator: tcuhawenimana11

Excerpt Created On: 3/21/2023

Excerpt Range: 49701-50025

indi ngaruka numva baba batongeye kuvuga iyo nakongera ho umukobwa ashobora guhura nazo ni uko ashobora no kunyereza nimba umura ukaba wakwiyubika bityo bigatuma n ubundi nta rubyaro azagira, indi cyangwa se ugasanga akuyemo inda kdi akiri muto ugasanga arakuze abuze urubyaro njye ni izo ngizo numva yahura nazo murakoze.

Title: Mukarange Boys Trnscribed.docx

Doc Creator: tcuhawenimana11

Doc Date: 3/20/2023

Codes Applied: Obstacles and risks experienced by girls during sexual and reproductive health changes occuring during puberty and adolescence

Excerpt Creator: tcuhawenimana11

Excerpt Created On: 3/21/2023

Excerpt Range: 50252-50480

none inzitiza za mbere ni ukugira ababyeyi kuba ababyeyi batagira abana inama bageze mu gihe cy ubwangavu izindi nzitizi na none za mbere ni ukugira ni ukudahura n abanyu benshi ngo batugire inama ni ukutabona amahugurwa yego.

Title: Mukarange Boys Trnscribed.docx

Doc Creator: tcuhawenimana11

Doc Date: 3/20/2023

Codes Applied: Obstacles and risks experienced by boys during the sexual and reproductive health changes occuring during puberty and adolescence

Excerpt Creator: tcuhawenimana11

Excerpt Created On: 3/21/2023

Excerpt Range: 50481-50725

Indi barriers cyangwa se challenge ashobora guhura nazo ni ukubura ubukangurambaga ku myororokere nyine y ingimbi n abangavu ikindi akabura aho akura amakuru nk uko bahora bavuga ngo icyambere ni amakuru iyo nta makuru nyine nta kintu wageraho.

Title: Mukarange Boys Trnscribed.docx

Doc Creator: tcuhawenimana11

Doc Date: 3/20/2023

Codes Applied: Obstacles and risks experienced by boys during the sexual and reproductive health changes occuring during puberty and adolescence Obstacles and risks experienced by girls during sexual and reproductive health changes occuring during puberty and adolescence

Excerpt Creator: tcuhawenimana11

Excerpt Created On: 3/21/2023

Excerpt Range: 50749-50934

indi nzitizi wenda umukobwa cyangwa twebwe twahura nazo harimo wenda nko kuba wowe ubwawe wumva nabi utinda kumva wowe ukumva y uko ibintu byose ubizi ibyo bakubwira atari byo murakoze.

Title: Mukarange Boys Trnscribed.docx

Doc Creator: tcuhawenimana11

Doc Date: 3/20/2023

Codes Applied: Obstacles and risks experienced by girls during sexual and reproductive health changes occuring during puberty and adolescence

Excerpt Creator: tcuhawenimana11

Excerpt Created On: 3/21/2023

Excerpt Range: 50749-50934

indi nzitizi wenda umukobwa cyangwa twebwe twahura nazo harimo wenda nko kuba wowe ubwawe wumva nabi utinda kumva wowe ukumva y uko ibintu byose ubizi ibyo bakubwira atari byo murakoze.

Title: Mukarange Boys Trnscribed.docx

Doc Creator: tcuhawenimana11

Doc Date: 3/20/2023

Codes Applied: Obstacles and risks experienced by boys during the sexual and reproductive health changes occuring during puberty and adolescence

Excerpt Creator: tcuhawenimana11

Excerpt Created On: 3/21/2023

Excerpt Range: 50964-51655

Izindi nzitizi ni ukuba utaragize ubushake bwo gushaka amakuru inzitizi zo kuba utaragize ubushake bwo gushaka amakuru ku buzima bw imyororokere.

Murakoze ndi nimero 2 izindi nzitizi duhura nazo ni uburyo wenda amakuru yatugeraho mu buryo bwihuse wenda bakaba bakoresha imbuga nkoranyambaga bikabasha kuduhugura ku buryo bwose tudafashe urugendo cyangwa se wenda nko ku byapa bakabimarkingaho cyane ku buryo buri wese nutabashije kuba yakora urugendo ajya aho izo meetings zabaye ziri kubivugaho yaca nka ahantu akavuga ati ariko kiriya cyapa nk isomo runaka rivuga rishaka kugira icyo rimpindurahomu buzima bwanjye mu buzima ngezemu mu gihe cy imyororokere bw ubusore n ingimbi murakoze.

Title: Mukarange Boys Trnscribed.docx

Doc Creator: tcuhawenimana11

Doc Date: 3/20/2023

Codes Applied: Suggestions to improve SRH during puberty and adolescence by boys

Excerpt Creator: tcuhawenimana11

Excerpt Created On: 3/21/2023

Excerpt Range: 51785-52239

none icyo navuga ni nko nk uburyo mwadufasha mukajya mukora ibiganiro henshi hatandukanye mukaganiriza abana bageze mu gihe cy ubugimbi cyangwa cy ubwangavu abadolescents kuko akenshiduhura n ibibazo tutabyiteguye tutanabizi kubera ko nta makuru. Nkurugero mukagenda mutanga nk amakuru n amahugurwa henshi cyane hagiye hatandukanye kugira ngo ejo ahazaza hacu habe heza kandi n igihugu kizatubyaze umusaruro tunagikorere tubashe kugiteza imbere murakoze.

Title: Munyiginya GS boy transcrib.docx

Doc Creator: tcuhawenimana11

Doc Date: 3/20/2023

Codes Applied: Physiological changes occuring during puberty-Boys

Excerpt Creator: tcuhawenimana11

Excerpt Created On: 3/22/2023

Excerpt Range: 276-496

nzimwe mu mpinduka umwana w’umuhungu agira igihe ageze mu gihe cy’ubugimbi, impinduka yambere nu Kuninga ijwi, iya kabiri Kwiroteraho, iya gatatu Bamwe na bamwe wenda bagira Ibiheri mu maso ndetse ba kamera n’ubwanwa.

Title: Munyiginya GS boy transcrib.docx

Doc Creator: tcuhawenimana11

Doc Date: 3/20/2023

Codes Applied: Boys and girls know about the causes of the causes leading to the physiological changes during puberty

Excerpt Creator: tcuhawenimana11

Excerpt Created On: 3/22/2023

Excerpt Range: 576-664

Impamvu ituma izo mpinduka ziba Ndacyeka ari Imisemburo y’umuntu kugiti cye iba mumubiri

Title: Munyiginya GS boy transcrib.docx

Doc Creator: tcuhawenimana11

Doc Date: 3/20/2023

Codes Applied: Physiological changes occuring during puberty-Boys

Excerpt Creator: tcuhawenimana11

Excerpt Created On: 3/22/2023

Excerpt Range: 865-1050

numva ko zimwe mumpiduka umwana w’umuhungungu ugeze mucyiciro cy’ubugimbi, agaragaza harimo kuzana ibiheri mumaso, kuniga ijwi, kwiroteraho ndetse no kuba yazana amatuza rimwe na rimwe.

Title: Munyiginya GS boy transcrib.docx

Doc Creator: tcuhawenimana11

Doc Date: 3/20/2023

Codes Applied: Boys and girls know about the causes of the causes leading to the physiological changes during puberty

Excerpt Creator: tcuhawenimana11

Excerpt Created On: 3/22/2023

Excerpt Range: 1051-1119

Nkaba numva byaba biterwa n’imisemburo yaba yifitemo imbere muri we.

Title: Munyiginya GS boy transcrib.docx

Doc Creator: tcuhawenimana11

Doc Date: 3/20/2023

Codes Applied: Boys and girls know about the causes of the causes leading to the physiological changes during puberty

Excerpt Creator: tcuhawenimana11

Excerpt Created On: 3/22/2023

Excerpt Range: 1269-1402

kuricyo kibazo warubajije nkunganiyeho ntabwo nahindura ibyo yaravuze nibyo gusa kuricyo cy’umusemburo biterwa n’igihe umuntu arimo.

Title: Munyiginya GS boy transcrib.docx

Doc Creator: tcuhawenimana11

Doc Date: 3/20/2023

Codes Applied: Boys and girls know about the causes of the causes leading to the physiological changes during puberty

Excerpt Creator: tcuhawenimana11

Excerpt Created On: 3/22/2023

Excerpt Range: 1449-1650

Harihihe umuntu aba ari mukuru ariko aho umuntu atandukanira n’uwundi, igihe umuntu amereyemo incakwaha n’ubwo bwanwa bitandukana n’icyundi amereramo ibyongibyo ibyo niho ibihe bitandukanira by’abantu.

Title: Munyiginya GS boy transcrib.docx

Doc Creator: tcuhawenimana11

Doc Date: 3/20/2023

Codes Applied: Physiological changes occuring during puberty-Boys

Excerpt Creator: tcuhawenimana11

Excerpt Created On: 3/22/2023

Excerpt Range: 1807-2018

ikintu numva gishobora cyateza kubaho izo mpinduka nanjye ntabwo narenza kuri bagenzi banjye ibyo bavuze ahubwo ikindi ushobora no kuba wanamera n’insya ndetse ukazana n’ubwo bucakwaha bitewe n’imisemburo ufite.

Title: Munyiginya GS boy transcrib.docx

Doc Creator: tcuhawenimana11

Doc Date: 3/20/2023

Codes Applied: Boys and girls know about the causes of the causes leading to the physiological changes during puberty

Excerpt Creator: tcuhawenimana11

Excerpt Created On: 3/22/2023

Excerpt Range: 1807-2018

ikintu numva gishobora cyateza kubaho izo mpinduka nanjye ntabwo narenza kuri bagenzi banjye ibyo bavuze ahubwo ikindi ushobora no kuba wanamera n’insya ndetse ukazana n’ubwo bucakwaha bitewe n’imisemburo ufite.

Title: Munyiginya GS boy transcrib.docx

Doc Creator: tcuhawenimana11

Doc Date: 3/20/2023

Codes Applied: Physiological changes occuring during puberty-Boys

Excerpt Creator: tcuhawenimana11

Excerpt Created On: 3/22/2023

Excerpt Range: 2479-2668

ikintu numva gishobora kuba gituma umuntu uri mubugimbi agira zimwe mumpinduka kumubiri we, harimo kumera insya, kumera ubwanwa ndetse akaba yananiga ijwi, akaba yanagira n’amatuza manini,

Title: Munyiginya GS boy transcrib.docx

Doc Creator: tcuhawenimana11

Doc Date: 3/20/2023

Codes Applied: Boys and girls know about the causes of the causes leading to the physiological changes during puberty

Excerpt Creator: tcuhawenimana11

Excerpt Created On: 3/22/2023

Excerpt Range: 2669-2772

nkaba numva ikintu gishobora kuba kibitera ari ubuzima yabayemo ndetse n’imisemburo yifitemo mumubiri.

Title: Munyiginya GS boy transcrib.docx

Doc Creator: tcuhawenimana11

Doc Date: 3/20/2023

Codes Applied: Physiological changes occuring during puberty-Girls

Excerpt Creator: tcuhawenimana11

Excerpt Created On: 3/22/2023

Excerpt Range: 3058-3259

Ye, kumukobwa ugeze mugihe cy’ubwangavu, impinduka nyinshi ni izigaragara tubona, harimo kuzana amabuno manini, gupfundura amabere, ndetse twumva yuko bajya no mugihe cy’ukwezi aricyo gihe k’imihango.

Title: Munyiginya GS boy transcrib.docx

Doc Creator: tcuhawenimana11

Doc Date: 3/20/2023

Codes Applied: Boys and girls know about the causes of the causes leading to the physiological changes during puberty

Excerpt Creator: tcuhawenimana11

Excerpt Created On: 3/22/2023

Excerpt Range: 3260-3387

cyo gihe nka ntekereza ko biterwa n’ imisemburo bafite ndetse n’ubuzima babayemo ndetse n’ubiryo babayeho muburyo butandukanye.

Title: Munyiginya GS boy transcrib.docx

Doc Creator: tcuhawenimana11

Doc Date: 3/20/2023

Codes Applied: Physiological changes occuring during puberty-Girls

Excerpt Creator: tcuhawenimana11

Excerpt Created On: 3/22/2023

Excerpt Range: 3654-3845

impinduka zindi numva ziba kumukobwa, nongeyeho kubwo mugenzi wanjye avuze, umukobwa nawe ashobora kugira ibiheri nkibyo umuhungu yagira, akamera n’izo ncakwaha akaba yamera n’izo nsya nawe.

Title: Munyiginya GS boy transcrib.docx

Doc Creator: tcuhawenimana11

Doc Date: 3/20/2023

Codes Applied: Physiological changes occuring during puberty-Girls

Excerpt Creator: tcuhawenimana11

Excerpt Created On: 3/22/2023

Excerpt Range: 3910-4017

Ibyo bagenzi banjye bavuze nibyo, nanone abakobwa hari ubwo bagira baamataye manini bitehe n’igihe arimo,

Title: Munyiginya GS boy transcrib.docx

Doc Creator: tcuhawenimana11

Doc Date: 3/20/2023

Codes Applied: Boys and girls know about the causes of the causes leading to the physiological changes during puberty

Excerpt Creator: tcuhawenimana11

Excerpt Created On: 3/22/2023

Excerpt Range: 4017-4106

nanone ikibitera hari igihe avukira ahantu heza akarya neza bigatuma ibyo byose bimubaho.

Title: Munyiginya GS boy transcrib.docx

Doc Creator: tcuhawenimana11

Doc Date: 3/20/2023

Codes Applied: Boys and girls know about the causes of the causes leading to the physiological changes during puberty

Excerpt Creator: tcuhawenimana11

Excerpt Created On: 3/22/2023

Excerpt Range: 4222-4433

twese tuziko uburere buruta ubuvuke, avutse ntarye neza, ntabeho neza yego niyo yaba mumuryango ukennye ariko akabona iyo ndyo yuzuye kuko wawundi utabonye indyo yuzuye ntabwo yagira ubuzima bwiza nk’uwayibonye.

Title: Munyiginya GS boy transcrib.docx

Doc Creator: tcuhawenimana11

Doc Date: 3/20/2023

Codes Applied: Physiological changes occuring during puberty-Girls

Excerpt Creator: tcuhawenimana11

Excerpt Created On: 3/22/2023

Excerpt Range: 4721-4844

izo mpinduka harimo kuzana amabuno, kumera amabere, kumera insya, no kumer n’ubucakwaha cyangwa bagasesa n’ibiheri mumaso.

Title: Munyiginya GS boy transcrib.docx

Doc Creator: tcuhawenimana11

Doc Date: 3/20/2023

Codes Applied: Physiological changes occuring during puberty-Girls

Excerpt Creator: tcuhawenimana11

Excerpt Created On: 3/22/2023

Excerpt Range: 4977-5229

Umukobwa wageze mugihe cy’ubwangavu bishobora kutagaragara ngo azane ayo mabuno, cyanwa azane nayo mabere ariko nyine umukobwa wageze mugihe cy’ubwangavu, aba ageze igihe nawe aba ashobora kujya ku isoko nawe agakundana atangira kwikoraho akimeza neza.

Title: Munyiginya GS boy transcrib.docx

Doc Creator: tcuhawenimana11

Doc Date: 3/20/2023

Codes Applied: Psychological changes occuring during puberty-Girls

Excerpt Creator: tcuhawenimana11

Excerpt Created On: 3/22/2023

Excerpt Range: 5132-5228

aba ageze igihe nawe aba ashobora kujya ku isoko nawe agakundana atangira kwikoraho akimeza neza

Title: Munyiginya GS boy transcrib.docx

Doc Creator: tcuhawenimana11

Doc Date: 3/20/2023

Codes Applied: Psychological changes occuring during puberty-Girls

Excerpt Creator: tcuhawenimana11

Excerpt Created On: 3/22/2023

Excerpt Range: 5334-5470

Iyo umukobwa amaze kwimeza neza, iyo ageze mugihe cy’ubwangavu, atangira kugira aba boyifurendi ariko iyo akiri mutoya ntabwo yamugira.

Title: Munyiginya GS boy transcrib.docx

Doc Creator: tcuhawenimana11

Doc Date: 3/20/2023

Codes Applied: Boys and girls know about the causes of the causes leading to the physiological changes during puberty

Excerpt Creator: tcuhawenimana11

Excerpt Created On: 3/22/2023

Excerpt Range: 5546-5728

ikindi gishobora kugaragaza mukobwa ugeze mugihe cy’ubwangavu, kidashobora kugaragara kumubiri we ariko abantu bashobora kubona cyangwa bakumva, uwo mukobwa atangira guhindura ijwi,

Title: Munyiginya GS boy transcrib.docx

Doc Creator: tcuhawenimana11

Doc Date: 3/20/2023

Codes Applied: Physiological changes occuring during puberty-Girls

Excerpt Creator: tcuhawenimana11

Excerpt Created On: 3/22/2023

Excerpt Range: 5546-5728

ikindi gishobora kugaragaza mukobwa ugeze mugihe cy’ubwangavu, kidashobora kugaragara kumubiri we ariko abantu bashobora kubona cyangwa bakumva, uwo mukobwa atangira guhindura ijwi,

Title: Munyiginya GS boy transcrib.docx

Doc Creator: tcuhawenimana11

Doc Date: 3/20/2023

Codes Applied: Physiological changes occuring during puberty-Girls

Excerpt Creator: tcuhawenimana11

Excerpt Created On: 3/22/2023

Excerpt Range: 5751-5873

Atangira kugira akajwi kayongobeye ukuntu gatandukanye n’ijwi rinini ry’abahungu cg akabana ukumva afite akajwi kari soft.

Title: Munyiginya GS boy transcrib.docx

Doc Creator: tcuhawenimana11

Doc Date: 3/20/2023

Codes Applied: Psychological changes occuring during puberty-Girls

Excerpt Creator: tcuhawenimana11

Excerpt Created On: 3/22/2023

Excerpt Range: 5916-6055

Ikindi nakongeraho, imyitwarire irahinduka akamera bitandukanye nuko yari ameze. Waramuherukaga akiri umwana mushobora guhura ukamuyoberwa.

Title: Munyiginya GS boy transcrib.docx

Doc Creator: tcuhawenimana11

Doc Date: 3/20/2023

Codes Applied: Psychological changes occuring during puberty-Girls

Excerpt Creator: tcuhawenimana11

Excerpt Created On: 3/22/2023

Excerpt Range: 6081-6166

Imyitwarire niba ari wamuntu wasangaga ajagaraye, usanga ibintu byose biri kumurongo.

Title: Munyiginya GS boy transcrib.docx

Doc Creator: tcuhawenimana11

Doc Date: 3/20/2023

Codes Applied: How boys experience physiological changes that occured to them during puberty

Excerpt Creator: tcuhawenimana11

Excerpt Created On: 3/22/2023

Excerpt Range: 6460-6729

Njyewe impinduka zambayeho, nanize ijwi, nzana n’ibiheri mumaso, ubaza; muri make suko wavugaga? Ntabwo aruko navugaga, uhhh, namera insya, naniroteraho, ukuntu nabifashe nkuko mumasomo twagendaga twiga ntabwo nabyakiriye nkibishyashya

nabyakiriye nyine nkuko nabyize.

Title: Munyiginya GS boy transcrib.docx

Doc Creator: tcuhawenimana11

Doc Date: 3/20/2023

Codes Applied: How boys experience physiological changes that occured to them during puberty

Excerpt Creator: tcuhawenimana11

Excerpt Created On: 3/22/2023

Excerpt Range: 6762-7023

Ibyo bintu biba rero ntabwi biza ngo bihite bikubaho byose. Uragenda niba ari ubucakwaha haza gake gake, niba ari ubwanwa haza gake gake nibwo ugenda ubyakira. Yenda bije icyarimo nibwo wamera nk’uhungabanyemo ariko uko bigenda biza gake gakeya ugenda wiyakira.

Title: Munyiginya GS boy transcrib.docx

Doc Creator: tcuhawenimana11

Doc Date: 3/20/2023

Codes Applied: How boys experience physiological changes that occured to them during puberty

Excerpt Creator: tcuhawenimana11

Excerpt Created On: 3/22/2023

Excerpt Range: 7173-7217

Niroteyeho, nzana insya, mera n’ubucakwaha;

Title: Munyiginya GS boy transcrib.docx

Doc Creator: tcuhawenimana11

Doc Date: 3/20/2023

Codes Applied: How boys experience physiological changes that occured to them during puberty

Excerpt Creator: tcuhawenimana11

Excerpt Created On: 3/22/2023

Excerpt Range: 7251-7314

narabyakiriye nyine nkuko twabyize mbifata ukonguko nyine biri.

Title: Munyiginya GS boy transcrib.docx

Doc Creator: tcuhawenimana11

Doc Date: 3/20/2023

Codes Applied: How boys experience physiological changes that occured to them during puberty

Excerpt Creator: tcuhawenimana11

Excerpt Created On: 3/22/2023

Excerpt Range: 7343-7722

impinduka nagize nkigitangira kwinjira mugihe cy’ubugimbi, natangiye niga ijwi ndetse nzana n’amatuza manini. Nyuma yahoo nibwo naje kubona ibintu bias nkaho bitunguranye ubundi najyaga numva bavuga nanjye ubwanjye binyiberaho aribyo bita kwiroteraho, bikimbaho umwanya umwe narikanze uwundi ariko numva ndishimye kuko bavugaga ko umuhungu muzima ibyo bimenyetso bigomba kumubaho

Title: Munyiginya GS boy transcrib.docx

Doc Creator: tcuhawenimana11

Doc Date: 3/20/2023

Codes Applied: How boys experience physiological changes that occured to them during puberty

Excerpt Creator: tcuhawenimana11

Excerpt Created On: 3/22/2023

Excerpt Range: 7776-7833

Yego nibwo nanjye nahise menya ko mbaye muzima. Murakoze.

Title: Munyiginya GS boy transcrib.docx

Doc Creator: tcuhawenimana11

Doc Date: 3/20/2023

Codes Applied: How boys experience physiological changes that occured to them during puberty

Excerpt Creator: tcuhawenimana11

Excerpt Created On: 3/22/2023

Excerpt Range: 7930-8045

Najyaga numva barimo kubivuga nkavuga nti njye bizambaho ryari? Bimbayeho nibwo nanjye numvise mbaye umuntu muzima.

Title: Munyiginya GS boy transcrib.docx

Doc Creator: tcuhawenimana11

Doc Date: 3/20/2023

Codes Applied: How boys experience physiological changes that occured to them during puberty

Excerpt Creator: tcuhawenimana11

Excerpt Created On: 3/22/2023

Excerpt Range: 8083-8547

Murakoze, njye ibintu byaje, najyaga numva nkabona abandi bavuga kandi njye bitaraza, nameze insya, nanjye nabonye zije numva nishimye numva mbaye umuntu mukuru nk’abandi. Nkumva abandi bavuga ngo bamee ubucakwaha kandi njye ntabwo mfite nkumva njye sindakura ariko bwaraje nyine numva ko nanjye natangiye gukura. Nize ijwi, nkumva abandi baravuga bitandukanye nanjye kubera ko bamaze kugera mugihe cy’ubugimbi, nize ijwi nanjye numva ko nageze mugihe cy’ubugimbi.

Title: Munyiginya GS boy transcrib.docx

Doc Creator: tcuhawenimana11

Doc Date: 3/20/2023

Codes Applied: How boys experience physiological changes that occured to them during puberty

Excerpt Creator: tcuhawenimana11

Excerpt Created On: 3/22/2023

Excerpt Range: 8692-8856

Ubundi, ukuntu inginmbi nyine nkanjye ukuntu nihereyeho, nanjye maze kumva ko maze gukura numva ko nanjye ngomba kwimeza neza kuburyo nanjye nabona uwo girlfriend.

Title: Munyiginya GS boy transcrib.docx

Doc Creator: tcuhawenimana11

Doc Date: 3/20/2023

Codes Applied: How boys experience physiological changes that occured to them during puberty

Excerpt Creator: tcuhawenimana11

Excerpt Created On: 3/22/2023

Excerpt Range: 8939-9402

imyitwarire y’umuntu ugeze hariya iyo atabereyemo umuntu mwiza usanga abereyemo umuntu mubi, kuberako umuntu wese arebye abona ntacyo amurusha kuberako niho havukiramo agasuzuguro imyitwarire mibi, wahura na papa wawe munzu wabona afite ubwanwa nawe ukabina urabufite, yabukoramo nawe ukavukoramo, hhhh, ariko iyo wabereyemo mwiza niho bavuga ngo igiti kigorororwa kikiri gitoya iyo waherewemo uburere bwiza, ariko iyo utarezwe neza biragoye gutsinda icyo kizame.

Title: Munyiginya GS boy transcrib.docx

Doc Creator: tcuhawenimana11

Doc Date: 3/20/2023

Codes Applied: How boys experience physiological changes that occured to them during puberty

Excerpt Creator: tcuhawenimana11

Excerpt Created On: 3/22/2023

Excerpt Range: 9476-9583

Kuriyo myitwarire y’ingimbi nubundi yose bayivuzeho, buri muhungu wese ibyo bimenyetso bimubaho biba bimwe.

Title: Munyiginya GS boy transcrib.docx

Doc Creator: tcuhawenimana11

Doc Date: 3/20/2023

Codes Applied: How boys experience physiological changes that occured to them during puberty

Excerpt Creator: tcuhawenimana11

Excerpt Created On: 3/22/2023

Excerpt Range: 9664-9906

Ariko nyine kuburyo witwara iyo ubibonye nkatwe nk’abantu bize, usanga hose guhera mumashuri abanza twaragiye tubyiga ntabwo bikubaho ngo bigutungure, ahubwo nkuko mugenzi wanjye yabivuze iyo bikubayeho urishima ukumva ko ubaye umuntu mukuru.

Title: Munyiginya GS boy transcrib.docx

Doc Creator: tcuhawenimana11

Doc Date: 3/20/2023

Codes Applied: Psychological changes occuring during puberty-Boys

Excerpt Creator: tcuhawenimana11

Excerpt Created On: 3/22/2023

Excerpt Range: 10256-10453

urumva nyine nkanjye, nkikiri umwana wasangaga ndi mumuhanda usanga ndimo ndakina ibifuniko mumuhanda, ariko nkubungubu nanjye ntabwo nabikora kuko mba mfite ubwoba bw’umuntu wambona ko yananseka.

Title: Munyiginya GS boy transcrib.docx

Doc Creator: tcuhawenimana11

Doc Date: 3/20/2023

Codes Applied: Psychological changes occuring during puberty-Boys

Excerpt Creator: tcuhawenimana11

Excerpt Created On: 3/22/2023

Excerpt Range: 10510-10538

utangira kugira impungenge,

Title: Munyiginya GS boy transcrib.docx

Doc Creator: tcuhawenimana11

Doc Date: 3/20/2023

Codes Applied: Psychological changes occuring during puberty-Boys How boys experience physiological changes that occured to them during puberty

Excerpt Creator: tcuhawenimana11

Excerpt Created On: 3/22/2023

Excerpt Range: 10569-11098

Njyewe impinduka zambayeho, nuko ibikorwa bimwe bibi nakoraga, nkimanara kuba ingimbi, mbere y’uko mba ingimbi hari ibikorwa bibi nakoraga bitaribyo, wenda nk’umubyeyi akambwira ati kora iki, kora iki ariko ubu mbikora batambwirije mbona ko bikwiye, ikindi nkumuntu utaraba ingimbi ibintu byo gukundana ntabwo ujya ubijyamo ariko iyo umaze kuba ingimbi, utangira kuvuga uti reka ntangire jye kucyangwe, umukobwa nambona basi abone ko hari ikintu cyahindutse basi abone ko hari ikintu cyahindutse tari nko mubwana,eeee ko ucyeye.

Title: Munyiginya GS boy transcrib.docx

Doc Creator: tcuhawenimana11

Doc Date: 3/20/2023

Codes Applied: How boys experience physiological changes that occured to them during puberty

Excerpt Creator: tcuhawenimana11

Excerpt Created On: 3/22/2023

Excerpt Range: 11487-11725

Iyo myitwarire mibi njye nubwo itarambaho, ariko bagenzi banjye tuganira bamwira ko aricyo gihe bageramo bakumva bashaka gukora sekisi, bakumv ko batabikoze ntabuzima baba bafite, eee akenshi ni iyo myitwarire ikunda kugaragara. Murakoze.

Title: Munyiginya GS boy transcrib.docx

Doc Creator: tcuhawenimana11

Doc Date: 3/20/2023

Codes Applied: Psychological changes occuring during puberty-Boys

Excerpt Creator: tcuhawenimana11

Excerpt Created On: 3/22/2023

Excerpt Range: 11487-11725

Iyo myitwarire mibi njye nubwo itarambaho, ariko bagenzi banjye tuganira bamwira ko aricyo gihe bageramo bakumva bashaka gukora sekisi, bakumv ko batabikoze ntabuzima baba bafite, eee akenshi ni iyo myitwarire ikunda kugaragara. Murakoze.

Title: Munyiginya GS boy transcrib.docx

Doc Creator: tcuhawenimana11

Doc Date: 3/20/2023

Codes Applied: Psychological changes occuring during puberty-Boys

Excerpt Creator: tcuhawenimana11

Excerpt Created On: 3/22/2023

Excerpt Range: 11802-11893

babikora murwego rwo kwishimisha ariko iyo babikoze ni naho bamekinga shuwa ko ari bazima.

Title: Munyiginya GS boy transcrib.docx

Doc Creator: tcuhawenimana11

Doc Date: 3/20/2023

Codes Applied: How boys experience physiological changes that occured to them during puberty

Excerpt Creator: tcuhawenimana11

Excerpt Created On: 3/22/2023

Excerpt Range: 12269-12360

njye naragerageje biranga, hahahah. Twari tugiye kubikora birangira badufashe tutarabikora.

Title: Munyiginya GS boy transcrib.docx

Doc Creator: tcuhawenimana11

Doc Date: 3/20/2023

Codes Applied: How boys experience physiological changes that occured to them during puberty

Excerpt Creator: tcuhawenimana11

Excerpt Created On: 3/22/2023

Excerpt Range: 12522-12979

murakoze, kuri icyo kijyanye nuko umusore ugeze mugihe cy’ubugimbi abenshi umubiri ukund gua naho uturushije imbaraga ukatujyanayo, noneho uko umubiri ubidusaba cyane bitewe nuko tureba babakobwa bari mugihe cy’ubwangavu tukumva turi kubifuza cyane tukumva turi gushaka kumenya nabo uko bateye. Noneho kubijyane, niho umuhungu atangira kuvuga ngo ko numva ngo barabikora, abahungu benshi tubikora, kugira ngo tujye kumva uko abandi babikora uko biba bimeze.

Title: Munyiginya GS boy transcrib.docx

Doc Creator: tcuhawenimana11

Doc Date: 3/20/2023

Codes Applied: Psychological changes occuring during puberty-Boys

Excerpt Creator: tcuhawenimana11

Excerpt Created On: 3/22/2023

Excerpt Range: 12522-12979

murakoze, kuri icyo kijyanye nuko umusore ugeze mugihe cy’ubugimbi abenshi umubiri ukund gua naho uturushije imbaraga ukatujyanayo, noneho uko umubiri ubidusaba cyane bitewe nuko tureba babakobwa bari mugihe cy’ubwangavu tukumva turi kubifuza cyane tukumva turi gushaka kumenya nabo uko bateye. Noneho kubijyane, niho umuhungu atangira kuvuga ngo ko numva ngo barabikora, abahungu benshi tubikora, kugira ngo tujye kumva uko abandi babikora uko biba bimeze.

Title: Munyiginya GS boy transcrib.docx

Doc Creator: tcuhawenimana11

Doc Date: 3/20/2023

Codes Applied: How boys experience physiological changes that occured to them during puberty

Excerpt Creator: tcuhawenimana11

Excerpt Created On: 3/22/2023

Excerpt Range: 13034-13114

yego, nigeze kubigeragezaho rimwe kugira ngo numve koko ibyo ngibyo baba bavuga.

Title: Munyiginya GS boy transcrib.docx

Doc Creator: tcuhawenimana11

Doc Date: 3/20/2023

Codes Applied: Information that girls need

Excerpt Creator: tcuhawenimana11

Excerpt Created On: 3/22/2023

Excerpt Range: 13956-14307

Amakuru numva cyane cyane umukobwa aba akeneye ni ugusobanukirwa n’igihe agezemo. Akigishwa kuba yakwirinda, cyane cyane gukora nkizo segisi kuko ayikoze bishobora kumuviramo ingaruka zo guhita asama bitateganijwe akiri mutoya. Rero nkaba numva inama ndetse n’amahugurwa uwo mukobwa yaba akeneye ari ugusobanukirwa buryo ki umuntu asama uburyo bicamo.

Title: Munyiginya GS boy transcrib.docx

Doc Creator: tcuhawenimana11

Doc Date: 3/20/2023

Codes Applied: Information that girls need

Excerpt Creator: tcuhawenimana11

Excerpt Created On: 3/22/2023

Excerpt Range: 14453-14695

njye ndumva umukobwa ugeze mugihe cy’ubwangavu, aba akeneye n’amakuru y’uburyo yakwitwar mugihe akeneye imihango. Kuko akenshi na kenshi usanga iyo abonye imihango bimutunguye igihe atabashije kwigirira isuku abantu benshi anyuzeho bamuseka.

Title: Munyiginya GS boy transcrib.docx

Doc Creator: tcuhawenimana11

Doc Date: 3/20/2023

Codes Applied: Information that girls need

Excerpt Creator: tcuhawenimana11

Excerpt Created On: 3/22/2023

Excerpt Range: 14807-15104

Amakuru umukobwa ashobora kuba akenera ni ukwigirira isuku kugira ngo ataba ikibazo muri bagenzi be mukwiyanduza wenda mugihe cy’imihango. Wenda ikindi ni ukugira ababyeyi bamuhora hafi bamugira inama z’impinduka, n’uburyo yakwitwara mugihe umubiri wanze ukamusunika mugukora ibindi bitari byiza.

Title: Munyiginya GS boy transcrib.docx

Doc Creator: tcuhawenimana11

Doc Date: 3/20/2023

Codes Applied: Information that boys need

Excerpt Creator: tcuhawenimana11

Excerpt Created On: 3/22/2023

Excerpt Range: 15328-15761

Amakuru umuhungu akenera, aba agomba kwigishwa n’umubyeyi we nka papa we n’urugero. Akaba yamwigisha kuko aba yaraciye muri icyo kigero akaba yamwigisha uburyo yakwitwaramo neza kuko turabizi iyo umuntu yiroteyeho ushobora no gusambana, no kuryamana n’umukobwa badakoresheje agakingirizo akaba yamutera inda rero amakuru aba akeneye nuko yakwigishwa akitabwaho n’umubyeyi we kuko aba yarabiciyemo akaba yamwigisha uburyo yakwirinda.

Title: Munyiginya GS boy transcrib.docx

Doc Creator: tcuhawenimana11

Doc Date: 3/20/2023

Codes Applied: Suggestions to improve SRH during puberty and adolescence by boys

Excerpt Creator: tcuhawenimana11

Excerpt Created On: 3/22/2023

Excerpt Range: 15329-15761

Amakuru umuhungu akenera, aba agomba kwigishwa n’umubyeyi we nka papa we n’urugero. Akaba yamwigisha kuko aba yaraciye muri icyo kigero akaba yamwigisha uburyo yakwitwaramo neza kuko turabizi iyo umuntu yiroteyeho ushobora no gusambana, no kuryamana n’umukobwa badakoresheje agakingirizo akaba yamutera inda rero amakuru aba akeneye nuko yakwigishwa akitabwaho n’umubyeyi we kuko aba yarabiciyemo akaba yamwigisha uburyo yakwirinda.

Title: Munyiginya GS boy transcrib.docx

Doc Creator: tcuhawenimana11

Doc Date: 3/20/2023

Codes Applied: Sources of information received during puberty

Excerpt Creator: tcuhawenimana11

Excerpt Created On: 3/22/2023

Excerpt Range: 16097-16150

Njye amakuru nahawe, icyogihe nayigishijwe na barimu

Title: Munyiginya GS boy transcrib.docx

Doc Creator: tcuhawenimana11

Doc Date: 3/20/2023

Codes Applied: Types of information provided to boys and girls during puberty Received information is right and provides sufficient information helping boys and girls during puberty

Excerpt Creator: tcuhawenimana11

Excerpt Created On: 3/22/2023

Excerpt Range: 16150-16476

batubwira buryo ki n’umuhungu mbere yo kwiroteraho cg se muricyo gihe kugira ngo abamfite ubuzima buzira umuze agomba kuba ashyukwa. Batubwira ko uko ushyukwa ari uguhaguruka kw’igitsina cy’umugabo kikaba cyafata umurego kigahindura ingano we bwite atabigizemo uruhare ahubwo ari ibitekerezo n’imisemburo irimbere mumubiri we.

Title: Munyiginya GS boy transcrib.docx

Doc Creator: tcuhawenimana11

Doc Date: 3/20/2023

Codes Applied: Received information is right and provides sufficient information helping boys and girls during puberty

Excerpt Creator: tcuhawenimana11

Excerpt Created On: 3/22/2023

Excerpt Range: 16534-16642

Andi makuru baduhaye batubwiye ko muri icyo gihe umukobwa aba agezemo cy’imihango muryamanye ashobora gusama

Title: Munyiginya GS boy transcrib.docx

Doc Creator: tcuhawenimana11

Doc Date: 3/20/2023

Codes Applied: Sources of information received during puberty

Excerpt Creator: tcuhawenimana11

Excerpt Created On: 3/22/2023

Excerpt Range: 16845-16920

amakuru nyine numvise ni ayo nakuye hano ku ishuri nkuku turimo turabyiga.

Title: Munyiginya GS boy transcrib.docx

Doc Creator: tcuhawenimana11

Doc Date: 3/20/2023

Codes Applied: Received information is not detailed (mixed) Types of information provided to boys and girls during puberty

Excerpt Creator: tcuhawenimana11

Excerpt Created On: 3/22/2023

Excerpt Range: 16921-17045

Ni uko natwe uramutse uryamanye n’umukobwa ugeze mugihe cy’ubwangavu mudakoresheje agakingirizo ushobora kuba wamutera inda.

Title: Munyiginya GS boy transcrib.docx

Doc Creator: tcuhawenimana11

Doc Date: 3/20/2023

Codes Applied: Sources of information received during puberty

Excerpt Creator: tcuhawenimana11

Excerpt Created On: 3/22/2023

Excerpt Range: 17237-17426

njyewe mama yambwiye ko iyo umuhungu ageze mugihe cy’ubugimbi, iyo atushije akaryamana n’umukobwa aba ashobora kumutera inda cyangwa akaba yagira izindi indwara yandurira muri icyo gikorwa.

Title: Munyiginya GS boy transcrib.docx

Doc Creator: tcuhawenimana11

Doc Date: 3/20/2023

Codes Applied: Types of information provided to boys and girls during puberty Received information is right and provides sufficient information helping boys and girls during puberty

Excerpt Creator: tcuhawenimana11

Excerpt Created On: 3/22/2023

Excerpt Range: 17300-17426

iyo atushije akaryamana n’umukobwa aba ashobora kumutera inda cyangwa akaba yagira izindi indwara yandurira muri icyo gikorwa.

Title: Munyiginya GS boy transcrib.docx

Doc Creator: tcuhawenimana11

Doc Date: 3/20/2023

Codes Applied: Sources of information received during puberty

Excerpt Creator: tcuhawenimana11

Excerpt Created On: 3/22/2023

Excerpt Range: 17693-17821

n’insuti zaijya zibivuga hanze kumuhanda nyine dutembera, kimwe nuko bagenzi banjye bashobora kuba barateye amada bakiri batoya

Title: Munyiginya GS boy transcrib.docx

Doc Creator: tcuhawenimana11

Doc Date: 3/20/2023

Codes Applied: Received information is right and provides sufficient information helping boys and girls during puberty

Excerpt Creator: tcuhawenimana11

Excerpt Created On: 3/22/2023

Excerpt Range: 17954-18162

jyewe papa wanjye yambwiye yuko umuntu iyo agejeje igihe aba yariroteyeho ashobora kuryamana n’umukobwa akamutera inda cyangwa se atanamutera inda akaba yanamwanduza indwara zitandukanye niyo makuru yampaye.

Title: Munyiginya GS boy transcrib.docx

Doc Creator: tcuhawenimana11

Doc Date: 3/20/2023

Codes Applied: Sources of information received during puberty

Excerpt Creator: tcuhawenimana11

Excerpt Created On: 3/22/2023

Excerpt Range: 17955-18162

jyewe papa wanjye yambwiye yuko umuntu iyo agejeje igihe aba yariroteyeho ashobora kuryamana n’umukobwa akamutera inda cyangwa se atanamutera inda akaba yanamwanduza indwara zitandukanye niyo makuru yampaye.

Title: Munyiginya GS boy transcrib.docx

Doc Creator: tcuhawenimana11

Doc Date: 3/20/2023

Codes Applied: Types of information provided to boys and girls during puberty

Excerpt Creator: tcuhawenimana11

Excerpt Created On: 3/22/2023

Excerpt Range: 17955-18162

jyewe papa wanjye yambwiye yuko umuntu iyo agejeje igihe aba yariroteyeho ashobora kuryamana n’umukobwa akamutera inda cyangwa se atanamutera inda akaba yanamwanduza indwara zitandukanye niyo makuru yampaye.

Title: Munyiginya GS boy transcrib.docx

Doc Creator: tcuhawenimana11

Doc Date: 3/20/2023

Codes Applied: Sources of information received during puberty

Excerpt Creator: tcuhawenimana11

Excerpt Created On: 3/22/2023

Excerpt Range: 18333-18461

njyewe mama yambwiye ko iyo umuhungu atangiye kugera mugihe cy’ubugimbi atangira kwiyumva nyine akumva ko afite imbaraga nyine.

Title: Munyiginya GS boy transcrib.docx

Doc Creator: tcuhawenimana11

Doc Date: 3/20/2023

Codes Applied: Types of information provided to boys and girls during puberty

Excerpt Creator: tcuhawenimana11

Excerpt Created On: 3/22/2023

Excerpt Range: 18334-18461

njyewe mama yambwiye ko iyo umuhungu atangiye kugera mugihe cy’ubugimbi atangira kwiyumva nyine akumva ko afite imbaraga nyine.

Title: Munyiginya GS boy transcrib.docx

Doc Creator: tcuhawenimana11

Doc Date: 3/20/2023

Codes Applied: Sources of information received during puberty

Excerpt Creator: tcuhawenimana11

Excerpt Created On: 3/22/2023

Excerpt Range: 18569-18645

Amenshi nyumvira munshuti zanjye n’abantu dukorana mubuzima bwa buri munsi.

Title: Munyiginya GS boy transcrib.docx

Doc Creator: tcuhawenimana11

Doc Date: 3/20/2023

Codes Applied: Received information is misleading and risky

Excerpt Creator: tcuhawenimana11

Excerpt Created On: 3/22/2023

Excerpt Range: 18646-18961

Gusa amwe ayo bafite usanga ari ibihuha kuko abenshi ntibaba baragize n’amahirwe yo kugera mu ishuri. Aho usanga bavuga ko umuntu ufite ubuzima bwiza ari uwuba yarasambanye. Ugahita ubona ingaruka zo gusambana harimo abo baba barafunze, harimo ababa bafite ingo nabo ubwabo batishoboye bakajya gushyiraho n’abandi.

Title: Munyiginya GS boy transcrib.docx

Doc Creator: tcuhawenimana11

Doc Date: 3/20/2023

Codes Applied: Types of information provided to boys and girls during puberty

Excerpt Creator: tcuhawenimana11

Excerpt Created On: 3/22/2023

Excerpt Range: 18748-18820

Aho usanga bavuga ko umuntu ufite ubuzima bwiza ari uwuba yarasambanye.

Title: Munyiginya GS boy transcrib.docx

Doc Creator: tcuhawenimana11

Doc Date: 3/20/2023

Codes Applied: Types of information provided to boys and girls during puberty Received information is right and provides sufficient information helping boys and girls during puberty Precautions to take during puberty for boys

Excerpt Creator: tcuhawenimana11

Excerpt Created On: 3/22/2023

Excerpt Range: 18961-19633

Gusa icyo gihe njyewe nagerageje kuganira n’umubyeyi, mama yambwiye yuko igihe ngezemo ngomba kwitonda, cyane cyane agirinama yuko bakobwa benshi nabo umubiri ujya ubarusha imbaraga bakaba bagira ubushake bwo kuba bakwihutira kwishakira umuhungu ambwira buryi ki snhobora kwitara abakobwa bakomeje kunyegera cyane kuburyo bashobora kuba banyangiriza umubiri. Yambwiye ko nshobora kubabwira ko ibyongibyo bashaka ko dukora ntagahunda mfitanye nabo. Ndetse n’ibyongibyo bashak ko dukora nta gahunda mfite. Ambwira ko nzihanganira amazina bazagenda banyita harimo kunyita ko ndi ikiremba, ndi imbwa ntacyo nshoboye, ambwira ko igihe kizagera bakabona ko ibyo byose bibeshyaga

Title: Munyiginya GS boy transcrib.docx

Doc Creator: tcuhawenimana11

Doc Date: 3/20/2023

Codes Applied: How boys experience physiological changes that occured to them during puberty

Excerpt Creator: tcuhawenimana11

Excerpt Created On: 3/22/2023

Excerpt Range: 19975-20143

iyo ugeze mugihe cy’ubugimbi utangira kumva ko ufite imbaraga, ushobora kwibeshya ko ufite imbaraga ukaba wakora imitimo ivunanye ugasanga ikwangirije ejo hawe hazaza.

Title: Munyiginya GS boy transcrib.docx

Doc Creator: tcuhawenimana11

Doc Date: 3/20/2023

Codes Applied: Psychological changes occuring during puberty-Boys

Excerpt Creator: tcuhawenimana11

Excerpt Created On: 3/22/2023

Excerpt Range: 19975-20143

iyo ugeze mugihe cy’ubugimbi utangira kumva ko ufite imbaraga, ushobora kwibeshya ko ufite imbaraga ukaba wakora imitimo ivunanye ugasanga ikwangirije ejo hawe hazaza.

Title: Munyiginya GS boy transcrib.docx

Doc Creator: tcuhawenimana11

Doc Date: 3/20/2023

Codes Applied: How boys experience physiological changes that occured to them during puberty

Excerpt Creator: tcuhawenimana11

Excerpt Created On: 3/22/2023

Excerpt Range: 20434-20586

Ahangaha ikindi navugaho nuko navuga ko ushobora kuba wibeshya ko wakuze ukaba watera umukobwa w’abandi inda rimwe na rimwe bikaba byakuviramo gufungwa.

Title: Munyiginya GS boy transcrib.docx

Doc Creator: tcuhawenimana11

Doc Date: 3/20/2023

Codes Applied: Obstacles and risks experienced by boys during the sexual and reproductive health changes occuring during puberty and adolescence

Excerpt Creator: tcuhawenimana11

Excerpt Created On: 3/22/2023

Excerpt Range: 20740-20845

Ikindi na none, iyo utifshe bikuvirambo gufata inshingano ukiri muto utaranageza igihe.

UBAZA: Nka gute?

Title: Munyiginya GS boy transcrib.docx

Doc Creator: tcuhawenimana11

Doc Date: 3/20/2023

Codes Applied: Obstacles and risks experienced by boys during the sexual and reproductive health changes occuring during puberty and adolescence

Excerpt Creator: tcuhawenimana11

Excerpt Created On: 3/22/2023

Excerpt Range: 20855-20997

Kuba watangira nko gutunga urugo ukiri muto. Nko kwita kubana, kwita kuruwo mugore wateye inda bikakuviramo gufata izo nshingano utazishoboye.

Title: Munyiginya GS boy transcrib.docx

Doc Creator: tcuhawenimana11

Doc Date: 3/20/2023

Codes Applied: Obstacles and risks experienced by boys during the sexual and reproductive health changes occuring during puberty and adolescence

Excerpt Creator: tcuhawenimana11

Excerpt Created On: 3/22/2023

Excerpt Range: 21124-21313

Urabona iyo wameze insya ukamera n’izo ncakwaha, uhita ukenera ibikoresho byo gukoresha ukuraho iyo myanda y’incakwaha n’insya. Bityo bikaba ingorane rimwe na rimwe uba udafite ubushobozi.

Title: Munyiginya GS boy transcrib.docx

Doc Creator: tcuhawenimana11

Doc Date: 3/20/2023

Codes Applied: How boys experience physiological changes that occured to them during puberty

Excerpt Creator: tcuhawenimana11

Excerpt Created On: 3/22/2023

Excerpt Range: 21531-21753

Iyo ugeze mugihe cy’ubugimbi, utangira kwigira ikigomeke ugatangira guta iwanyu ukajya kwibera ahandi hatari iwanyu, ukajya kunywa amatabi, amayoga nyine nk’ibyo, naho ugiye nyine ugashaka kunanirana ukiba ni ibyo nyine.

Title: Munyiginya GS boy transcrib.docx

Doc Creator: tcuhawenimana11

Doc Date: 3/20/2023

Codes Applied: Obstacles and risks experienced by boys during the sexual and reproductive health changes occuring during puberty and adolescence

Excerpt Creator: tcuhawenimana11

Excerpt Created On: 3/22/2023

Excerpt Range: 21793-22161

njyewe, ingorane zambere mbona umuhungu ugeze mugihe cy’ubugimbi agenda ahura nazo, ikintu cya 1 kigorana ni inshuti, abantu ubana nabo, baragushuka bitewe nukuntu nawe urimo kwiyumva ko wabaye umusore, bakaba bakujyana mungeso zimwe na zimwe bakora n’bantu bakuru nubwo ziba Atari nziza ukaba ushaka kuzikuriramo zihgahita zikwicira ubuzima.

UBAZA: Waduha nk’ingero?

Title: Munyiginya GS boy transcrib.docx

Doc Creator: tcuhawenimana11

Doc Date: 3/20/2023

Codes Applied: How to take care of themselves when phsyiological changes occur for boys Received information is misleading and risky

Excerpt Creator: tcuhawenimana11

Excerpt Created On: 3/22/2023

Excerpt Range: 22171-22685

eeee, urugero, batangira kukwereka ko ugeze mugihe cyo gukenera amafaranga bakakwereka n’uburyo ushobora kuyabonamo noneho bakakujyana mumirimo igoranye bakakubwira ko ugomba kuva mu ishuri, wenda ukajya nko kuragira inka z’abaturage kugira ngo ubashe kuba wabona amafaranga yo kwikoraho kuko nawe uba umaze kumva ko ukeneye amafaranga wakwigira icyo gihe rero uragenda iryo shuri ugasanga urariretse nibyo wakoraga bikaba birapfuye nako kazi kagahagarara kubera bwabwana wajyanye biba bigoye kugarura icyo kizere.

Title: Munyiginya GS boy transcrib.docx

Doc Creator: tcuhawenimana11

Doc Date: 3/20/2023

Codes Applied: Obstacles and risks experienced by girls during sexual and reproductive health changes occuring during puberty and adolescence

Excerpt Creator: tcuhawenimana11

Excerpt Created On: 3/22/2023

Excerpt Range: 22777-23008

Ingorane bahura nazo, urabona iyo umukobwa yatangiye kugira ibyo biheri, aba yumva kuba afite ibyo biheri bashobora kuba batamwemera. Agashaka kuba yashaka amavuta meza yo kuba yakiza ibyo biheri kandi akiri umwana atarayashakaga.

Title: Munyiginya GS boy transcrib.docx

Doc Creator: tcuhawenimana11

Doc Date: 3/20/2023

Codes Applied: Obstacles and risks experienced by girls during sexual and reproductive health changes occuring during puberty and adolescence

Excerpt Creator: tcuhawenimana11

Excerpt Created On: 3/22/2023

Excerpt Range: 23050-23347

wenda shobora gukora saye kuko aba yiga atayabona, kandi ari ahantu hakennye ugasanga abandi bakobwa bagenzi be nka bariya b’indaya ugasanga baramushutse bamubwiye uko yabonamo amafaranga akoze imibonano mpuzabitsinda n’umuhungu ashobora kuba yamuha amafaranga akaba yakwigurira ayo mavuta ashaka.

Title: Munyiginya GS boy transcrib.docx

Doc Creator: tcuhawenimana11

Doc Date: 3/20/2023

Codes Applied: Obstacles and risks experienced by girls during sexual and reproductive health changes occuring during puberty and adolescence

Excerpt Creator: tcuhawenimana11

Excerpt Created On: 3/22/2023

Excerpt Range: 23529-23606

umukobwa yumva ko akuze akajya gushaka abagabo kandi atakuze akajysa gushaka.

Title: Munyiginya GS boy transcrib.docx

Doc Creator: tcuhawenimana11

Doc Date: 3/20/2023

Codes Applied: Obstacles and risks experienced by girls during sexual and reproductive health changes occuring during puberty and adolescence

Excerpt Creator: tcuhawenimana11

Excerpt Created On: 3/22/2023

Excerpt Range: 23731-23874

ingorane kubakobwa, abakobwa bashobora kwishora mubiyobyabwenge, ashobora kwishora mungorane zo kubyara ighe kitageze, bikamwicira ejo hazaza.

Title: Munyiginya GS boy transcrib.docx

Doc Creator: tcuhawenimana11

Doc Date: 3/20/2023

Codes Applied: Obstacles and risks experienced by girls during sexual and reproductive health changes occuring during puberty and adolescence

Excerpt Creator: tcuhawenimana11

Excerpt Created On: 3/22/2023

Excerpt Range: 23924-24169

Njye ingorane mbona abana b’abakobwa bahura nazo, harimo kuba batwara inda zitateganijwe. Iyo bageze muri icyo gihe umubiri wabo ubakururira gukorera imibonano rimwe na rimwe badakoresheje agakingirizo bikaba byabaviramo gutwara inda imburagihe.

Title: Munyiginya GS boy transcrib.docx

Doc Creator: tcuhawenimana11

Doc Date: 3/20/2023

Codes Applied: Obstacles and risks experienced by girls during sexual and reproductive health changes occuring during puberty and adolescence

Excerpt Creator: tcuhawenimana11

Excerpt Created On: 3/22/2023

Excerpt Range: 24214-24463

Ikintu mbona imbogamizi ni ikigero abakobwa baba bagezemo bakagira intege nke cyane, umuntu wese iki kigero aba yabasha kukimufatiramo akamushuka akamujyana mubyo yishakira. Ubwo rero iyo adafite inyigisho zihagije biragora kunyura muri icyo kiciro

Title: Munyiginya GS boy transcrib.docx

Doc Creator: tcuhawenimana11

Doc Date: 3/20/2023

Codes Applied: Obstacles and risks experienced by girls during sexual and reproductive health changes occuring during puberty and adolescence

Excerpt Creator: tcuhawenimana11

Excerpt Created On: 3/22/2023

Excerpt Range: 24543-24602

Icyo kigero yari akigezemo arashukwa birangira atewe inda.

Title: Munyiginya GS boy transcrib.docx

Doc Creator: tcuhawenimana11

Doc Date: 3/20/2023

Codes Applied: Obstacles and risks experienced by boys during the sexual and reproductive health changes occuring during puberty and adolescence

Excerpt Creator: tcuhawenimana11

Excerpt Created On: 3/22/2023

Excerpt Range: 24914-25161

inzitizi njye mbona haba kubakobwa cg abahungu bahura nazo, mbere na mbere ni ukutamenya makuru. Kutamenya imiterere y’imibiri yabo noneho bigatuma n’ubikoze ntabwenge asyiramo ntanakoreshe agakingirizo ugasanga bimuviriyemo no kwandura izo ndwara

Title: Munyiginya GS boy transcrib.docx

Doc Creator: tcuhawenimana11

Doc Date: 3/20/2023

Codes Applied: Obstacles and risks experienced by girls during sexual and reproductive health changes occuring during puberty and adolescence

Excerpt Creator: tcuhawenimana11

Excerpt Created On: 3/22/2023

Excerpt Range: 24914-25163

inzitizi njye mbona haba kubakobwa cg abahungu bahura nazo, mbere na mbere ni ukutamenya makuru. Kutamenya imiterere y’imibiri yabo noneho bigatuma n’ubikoze ntabwenge asyiramo ntanakoreshe agakingirizo ugasanga bimuviriyemo no kwandura izo ndwara.

Title: Munyiginya GS boy transcrib.docx

Doc Creator: tcuhawenimana11

Doc Date: 3/20/2023

Codes Applied: Obstacles and risks experienced by girls during sexual and reproductive health changes occuring during puberty and adolescence Obstacles and risks experienced by boys during the sexual and reproductive health changes occuring during puberty and adolescence

Excerpt Creator: tcuhawenimana11

Excerpt Created On: 3/22/2023

Excerpt Range: 25246-25547

inzitizi njyewe mbona akenshi ziterwa n’amakimbirane aba mumuryango. Kuko iyo mumuryango harimo amakimbirane, bituma wa mwana ababyeyi be batamuganiriza bityo bigatuma yifatira imyanzuro bikaba rimwe na rimwe byamuviramo no kuba kumukobwa yatwara inda imburagihe cg kumuhungu yatera inda imbura gihe.

Title: Munyiginya GS boy transcrib.docx

Doc Creator: tcuhawenimana11

Doc Date: 3/20/2023

Codes Applied: Obstacles and risks experienced by girls during sexual and reproductive health changes occuring during puberty and adolescence

Excerpt Creator: tcuhawenimana11

Excerpt Created On: 3/22/2023

Excerpt Range: 25586-25820

inzitizi mbona muri icyo gihe akenshi abantu baba bafite isoni zo kuvuga ibyababayeho no kuba yabwira cyangwa agishe inama umuntu mukuru ahubwo agashaka inshuti ye nayo ikabayamuha amakuru atizeye we ubwe akaba yakwishora mungeso mbi.

Title: Munyiginya GS boy transcrib.docx

Doc Creator: tcuhawenimana11

Doc Date: 3/20/2023

Codes Applied: Obstacles and risks experienced by boys during the sexual and reproductive health changes occuring during puberty and adolescence Obstacles and risks experienced by girls during sexual and reproductive health changes occuring during puberty and adolescence

Excerpt Creator: tcuhawenimana11

Excerpt Created On: 3/22/2023

Excerpt Range: 25916-26305

Urabona iyo umuryango urimo amakimbirane umwangavu cyangwa ingimbi, iyo ari umuhungu w’ingimbi akabona nkiwabo bahora mumakimbirane runaka, we ahita atangira gupanga buryo ki yajya gushakira ahandi ubuzima ni hamwe usanga iyo umuntu iyo agiye gushaka akazi akakabura ahita aba umujura, ugasanga cyangwa anyweye ibiyobyabwenge akumva ko ari byo byakiza ayo makimbirane no kwigunga muri we.

Title: Munyiginya GS boy transcrib.docx

Doc Creator: tcuhawenimana11

Doc Date: 3/20/2023

Codes Applied: Obstacles and risks experienced by girls during sexual and reproductive health changes occuring during puberty and adolescence

Excerpt Creator: tcuhawenimana11

Excerpt Created On: 3/22/2023

Excerpt Range: 26351-26587

Inzitizi nyine, kwakundi amakimbirane y’imiryango hahandi umukobwa w’ingimbi iyo nyina yumvise atangiye gukura ahita ajya gushaka akazi kwakundi aba yabonye inshuti ye imurangira akazi akajya kugakora agahura wenda n’abahungu bamushuka.

Title: Munyiginya GS boy transcrib.docx

Doc Creator: tcuhawenimana11

Doc Date: 3/20/2023

Codes Applied: Obstacles and risks experienced by boys during the sexual and reproductive health changes occuring during puberty and adolescence

Excerpt Creator: tcuhawenimana11

Excerpt Created On: 3/22/2023

Excerpt Range: 26640-26686

Umuhungu inzitizi ahura nazo Ni kwakunanirana.

Title: Munyiginya GS boy transcrib.docx

Doc Creator: tcuhawenimana11

Doc Date: 3/20/2023

Codes Applied: Obstacles and risks experienced by boys during the sexual and reproductive health changes occuring during puberty and adolescence

Excerpt Creator: tcuhawenimana11

Excerpt Created On: 3/22/2023

Excerpt Range: 26706-27309

njye inzitizi ya mbere mbona ishobora kubaho Ni ubujiji, icya kabiri akaba akwibeshya we ubwe bwite akaba yakwibwira ibitari byo ndetse yanabaza agashakira amakuru ahatariho ugasanga urwo ruhurirane rw’ubujiji rwose ruri kumujyahooo rugize ingaruk nyinshi kubuzima bwe. Ikindi cya 2 mbona cy’imbogamizi ikunze no kugora benshi Ni ubukene, ugasanga mubukene umuntu abayemo buramufatiranye nundi muntu wakagize icyo yamufashije wenda ngo abashe kubaa yamufasha ngo asohoke muri ubwo bukene ahubwo ugasanga amufatiraniyemo amushoye mungeso mbi amubeshya ko ashobora kuba yamukura muri ubwo bukene murakoze.

Title: Munyiginya GS boy transcrib.docx

Doc Creator: tcuhawenimana11

Doc Date: 3/20/2023

Codes Applied: Received information is misleading and risky

Excerpt Creator: tcuhawenimana11

Excerpt Created On: 3/22/2023

Excerpt Range: 27558-28143

Murakoze, twatangiye tuganira, njye kuri ibyongibyo akomeje kugiraho ikibazo, mwagarutse kubintu byo gukora sex, mugaruka no kubintu bijyanye no gukoresha udukingirizo, kandi hari amakuru menshi agenda agaruka ngo ukoresha agakingirizo ngo ariya mavuta aba ari mugakingirizo ngo nayo iyo ukomeje kugakoresha inshuro nyinshi nyo ngo agutera indwara, ibyo muratubwira koo niba ariya mavuta ashobora gutera umuntu indwara?

Ikindi cya kabiri ngo agakingirizo ngo ushobora kugakoresha kakajya mugitsina cy’umukobwa kakamubyimbiramo ngo agaturika? Ibyo nabyo muratubwira niba koko ari ukuri?

Title: Munyiginya GS boy transcrib.docx

Doc Creator: tcuhawenimana11

Doc Date: 3/20/2023

Codes Applied: Suggestions to improve SRH during puberty and adolescence by boys

Excerpt Creator: tcuhawenimana11

Excerpt Created On: 3/22/2023

Excerpt Range: 28818-29089

Njye ntabwo ari ikibazo narimfite igitekerezo, wenda nkuko abenshi murubyiruko batamenya imyororokere y’umubiri wabo wenda babaha nko mucyaro wenda bazana ikigo gishinzwe gutanga amakuru kurubyiruko kugira ngo batazajya bashukwa kandi wenda kandi abakabahaye amakuru meza

Title: Munyiginya GS boy transcrib.docx

Doc Creator: tcuhawenimana11

Doc Date: 3/20/2023

Codes Applied: Suggestions to improve SRH during puberty and adolescence by boys

Excerpt Creator: tcuhawenimana11

Excerpt Created On: 3/22/2023

Excerpt Range: 29303-29586

Njyewe ikindi kintu numvaga ni inyunganizi nagiraga ngo ntanjye kubantu bageze muri iki gihe, kunzobere abantu bakwegera ababyeyi bakabaganiriza umubyeyi usanga aganiriza umwana ariko umwana ntamubwire byose ahubwo umubyeyi ajye yegera inshuti zuwo mwana ziba zifite amakuru kurenza.

Title: Mulinga boys.docx

Doc Creator: tcuhawenimana11

Doc Date: 3/20/2023

Codes Applied: Physiological changes occuring during puberty-Boys

Excerpt Creator: tcuhawenimana11

Excerpt Created On: 3/22/2023

Excerpt Range: 897-1046

Harimo kuzana ibiheri mu maso, kumera insya ni ncakwaha, kuniga ijwi no kwiroteraho zikaba ziterwa n’imisemburo y’umuntu bitewe n’igihe aba agezemo.

Title: Mulinga boys.docx

Doc Creator: tcuhawenimana11

Doc Date: 3/20/2023

Codes Applied: Physiological changes occuring during puberty-Boys

Excerpt Creator: tcuhawenimana11

Excerpt Created On: 3/22/2023

Excerpt Range: 1100-1191

umuntu azana ibiheri mu maso, akaniga ijwi, akazana ibituza, akiroteraho eee ndumva aribyo.

Title: Mulinga boys.docx

Doc Creator: tcuhawenimana11

Doc Date: 3/20/2023

Codes Applied: Boys and girls know about the causes of the causes leading to the physiological changes during puberty

Excerpt Creator: tcuhawenimana11

Excerpt Created On: 3/22/2023

Excerpt Range: 1212-1301

Biterwa n’imyaka afite hari n’igihe biba biterwa n’imisemburo afite wenda yo mu muryango.

Title: Mulinga boys.docx

Doc Creator: tcuhawenimana11

Doc Date: 3/20/2023

Codes Applied: Physiological changes occuring during puberty-Boys

Excerpt Creator: tcuhawenimana11

Excerpt Created On: 3/22/2023

Excerpt Range: 1455-1554

Ukuntu umuntu nyine ahinduka aniga ijwi, akazana amatuza, akamera incakwaha ni nsya akaniroteraho.

Title: Mulinga boys.docx

Doc Creator: tcuhawenimana11

Doc Date: 3/20/2023

Codes Applied: Boys and girls know about the causes of the causes leading to the physiological changes during puberty

Excerpt Creator: tcuhawenimana11

Excerpt Created On: 3/22/2023

Excerpt Range: 1580-1630

Ikintu kibitera nimisemburo ibi imurimo mumubiri.

Title: Mulinga boys.docx

Doc Creator: tcuhawenimana11

Doc Date: 3/20/2023

Codes Applied: Physiological changes occuring during puberty-Boys

Excerpt Creator: tcuhawenimana11

Excerpt Created On: 3/22/2023

Excerpt Range: 1735-1822

Impinduka nzi nukumera incakwaha, akazana ibiheri mu maso agahinduka no mu bitekerezo.

Title: Mulinga boys.docx

Doc Creator: tcuhawenimana11

Doc Date: 3/20/2023

Codes Applied: Boys and girls know about the causes of the causes leading to the physiological changes during puberty

Excerpt Creator: tcuhawenimana11

Excerpt Created On: 3/22/2023

Excerpt Range: 1842-1892

Rimwe biterwa n’imyaka agezemo cyangwa imisemburo.

Title: Mulinga boys.docx

Doc Creator: tcuhawenimana11

Doc Date: 3/20/2023

Codes Applied: Physiological changes occuring during puberty-Boys

Excerpt Creator: tcuhawenimana11

Excerpt Created On: 3/22/2023

Excerpt Range: 1959-2062

Noneho impinduka harimo kumera insya, kuzana ibiheri mu maso, hakazamo gushyuha cyane no kuzana amatuza

Title: Mulinga boys.docx

Doc Creator: tcuhawenimana11

Doc Date: 3/20/2023

Codes Applied: Boys and girls know about the causes of the causes leading to the physiological changes during puberty

Excerpt Creator: tcuhawenimana11

Excerpt Created On: 3/22/2023

Excerpt Range: 2063-2096

bigaterwa n’imisemburo y’umubiri.

Title: Mulinga boys.docx

Doc Creator: tcuhawenimana11

Doc Date: 3/20/2023

Codes Applied: Physiological changes occuring during puberty-Boys

Excerpt Creator: tcuhawenimana11

Excerpt Created On: 3/22/2023

Excerpt Range: 2176-2283

Impinduka zigaragara ku mwana w’ingimbi nuko amera insya n’incakwaha, akaniga ijwi’ akazana ibiheri mumaso.

Title: Mulinga boys.docx

Doc Creator: tcuhawenimana11

Doc Date: 3/20/2023

Codes Applied: Boys and girls know about the causes of the causes leading to the physiological changes during puberty

Excerpt Creator: tcuhawenimana11

Excerpt Created On: 3/22/2023

Excerpt Range: 2284-2357

Ikibitera byaterwa n’igihe agezemo n’imyaka ye n’umusemburo w’umubiri we.

Title: Mulinga boys.docx

Doc Creator: tcuhawenimana11

Doc Date: 3/20/2023

Codes Applied: Physiological changes occuring during puberty-Girls

Excerpt Creator: tcuhawenimana11

Excerpt Created On: 3/22/2023

Excerpt Range: 2793-2941

Izo mpinduka rero atangira kumera amabere, akajya mu mihango, agatangira kumera amabuno akamera n’insya akenshi n’abakobwa bakunda kumera ubucakwaha

Title: Mulinga boys.docx

Doc Creator: tcuhawenimana11

Doc Date: 3/20/2023

Codes Applied: Boys and girls know about the causes of the causes leading to the physiological changes during puberty

Excerpt Creator: tcuhawenimana11

Excerpt Created On: 3/22/2023

Excerpt Range: 2942-2986

ibyo bishobora guterwa n’imisemburo myinshi.

Title: Mulinga boys.docx

Doc Creator: tcuhawenimana11

Doc Date: 3/20/2023

Codes Applied: Physiological changes occuring during puberty-Girls

Excerpt Creator: tcuhawenimana11

Excerpt Created On: 3/22/2023

Excerpt Range: 3066-3216

Impinduka zibaho ku mukobwa nuko amera amabere, akazana ibishishi mu maso, akazana amabuno, akazana n’insya, noneho uko ibintu bigenda ajya mu mihango

Title: Mulinga boys.docx

Doc Creator: tcuhawenimana11

Doc Date: 3/20/2023

Codes Applied: Boys and girls know about the causes of the causes leading to the physiological changes during puberty

Excerpt Creator: tcuhawenimana11

Excerpt Created On: 3/22/2023

Excerpt Range: 3217-3283

noneho bigaterwa n’imyaka aba afite kugirango agire iyo misemburo.

Title: Mulinga boys.docx

Doc Creator: tcuhawenimana11

Doc Date: 3/20/2023

Codes Applied: Physiological changes occuring during puberty-Girls

Excerpt Creator: tcuhawenimana11

Excerpt Created On: 3/22/2023

Excerpt Range: 3363-3500

Impinduka rero umwana w’umukobwa ugeze mu gihe cy’ubwangavu agira azana amabere noneho agatangira kugira ijwi ryoroheje, akazana n’insya

Title: Mulinga boys.docx

Doc Creator: tcuhawenimana11

Doc Date: 3/20/2023

Codes Applied: Boys and girls know about the causes of the causes leading to the physiological changes during puberty

Excerpt Creator: tcuhawenimana11

Excerpt Created On: 3/22/2023

Excerpt Range: 3500-3542

bigaterwa n’imisemburo afite mu mubiri we.

Title: Mulinga boys.docx

Doc Creator: tcuhawenimana11

Doc Date: 3/20/2023

Codes Applied: Physiological changes occuring during puberty-Girls

Excerpt Creator: tcuhawenimana11

Excerpt Created On: 3/22/2023

Excerpt Range: 3641-3834

Impinduka umwana w’umukobwa zimugaragaraho ageze mu gihe cy’ubwangavu n’ukumera amabere, insya n’incakwaha, akajya mu mihango, akazana amabuno, akagira ijwi ryoroheje nibishishi mu maso hanyuma

Title: Mulinga boys.docx

Doc Creator: tcuhawenimana11

Doc Date: 3/20/2023

Codes Applied: Boys and girls know about the causes of the causes leading to the physiological changes during puberty

Excerpt Creator: tcuhawenimana11

Excerpt Created On: 3/22/2023

Excerpt Range: 3835-3904

ikibitera n’imisemburo ye yaba ivubura cyane cyangwa ikora vuba vuba.

Title: Mulinga boys.docx

Doc Creator: tcuhawenimana11

Doc Date: 3/20/2023

Codes Applied: How boys experience physiological changes that occured to them during puberty

Excerpt Creator: tcuhawenimana11

Excerpt Created On: 3/22/2023

Excerpt Range: 4099-4346

Ngewe ibyambayeho n’ukwirotera uko nabyitwayemo najyaga numva abandi bari bakuze kunduta bakavuga ngo iyo umuntu yiroteye aba yakuze cyangwa ageze mu gihe cy’ubugimbi nange mbifata gutyo ko ngeze mu gihe cy’ubugimbi nyine numva yuko nange nakuze

Title: Mulinga boys.docx

Doc Creator: tcuhawenimana11

Doc Date: 3/20/2023

Codes Applied: How boys experience physiological changes that occured to them during puberty

Excerpt Creator: tcuhawenimana11

Excerpt Created On: 3/22/2023

Excerpt Range: 4464-4556

Impinduka zambayeho niroteyeho mbibonye ndeba umu mama ndabimubaza aransobanurira, murakoze.

Title: Mulinga boys.docx

Doc Creator: tcuhawenimana11

Doc Date: 3/20/2023

Codes Applied: Sources of information received during puberty

Excerpt Creator: tcuhawenimana11

Excerpt Created On: 3/22/2023

Excerpt Range: 4504-4556

ndeba umu mama ndabimubaza aransobanurira, murakoze.

Title: Mulinga boys.docx

Doc Creator: tcuhawenimana11

Doc Date: 3/20/2023

Codes Applied: How boys experience physiological changes that occured to them during puberty

Excerpt Creator: tcuhawenimana11

Excerpt Created On: 3/22/2023

Excerpt Range: 4624-4898

Impinduka zambayeho muri cya gihe cy’ubugimbi nameze insya mbanza kugira ubwoba ndibaza ni ko nameze umusatsi kandi umusatsi uba kumutwe hanyuma nza kubaza bakuru bange baza kubwira ko umuntu wakuze amera insya bambwira ko byitwa insya numva ndatuje numva ntakibazo binteye.

Title: Mulinga boys.docx

Doc Creator: tcuhawenimana11

Doc Date: 3/20/2023

Codes Applied: Sources of information received during puberty

Excerpt Creator: tcuhawenimana11

Excerpt Created On: 3/22/2023

Excerpt Range: 4761-4792

hanyuma nza kubaza bakuru bange

Title: Mulinga boys.docx

Doc Creator: tcuhawenimana11

Doc Date: 3/20/2023

Codes Applied: How boys experience physiological changes that occured to them during puberty

Excerpt Creator: tcuhawenimana11

Excerpt Created On: 3/22/2023

Excerpt Range: 4990-5190

Impinduka zambayeho nukwirotera ngirango n’ibintu bikaze mu gitondo mbyutse mbaza abantu bagenzi bange barambwira ngo iyo umuntu yatangiye gukura yageze mu gihe cy’ubugimbi nibwo atangira kwiroteraho.

Title: Mulinga boys.docx

Doc Creator: tcuhawenimana11

Doc Date: 3/20/2023

Codes Applied: Sources of information received during puberty

Excerpt Creator: tcuhawenimana11

Excerpt Created On: 3/22/2023

Excerpt Range: 5066-5092

mbaza abantu bagenzi bange

Title: Mulinga boys.docx

Doc Creator: tcuhawenimana11

Doc Date: 3/20/2023

Codes Applied: How boys experience physiological changes that occured to them during puberty

Excerpt Creator: tcuhawenimana11

Excerpt Created On: 3/22/2023

Excerpt Range: 5350-5520

Ngewe nange nameze insya mbaza bakuru bange n’inshuti barambwira bati ubu nawe wageze mu gihe cy’ubugimbi mbifata gutyo nyine ndavuga ni ubwo nange nakuze nagiye mu bandi

Title: Mulinga boys.docx

Doc Creator: tcuhawenimana11

Doc Date: 3/20/2023

Codes Applied: Sources of information received during puberty

Excerpt Creator: tcuhawenimana11

Excerpt Created On: 3/22/2023

Excerpt Range: 5375-5403

mbaza bakuru bange n’inshuti

Title: Mulinga boys.docx

Doc Creator: tcuhawenimana11

Doc Date: 3/20/2023

Codes Applied: How boys experience physiological changes that occured to them during puberty

Excerpt Creator: tcuhawenimana11

Excerpt Created On: 3/22/2023

Excerpt Range: 5586-5719

Ngewe ibyambayeho nukuniga ijwi ariko nagizengo n’uburwayi abantu bakuru nibo bajyaga bambwira ngo nakuze numva ko ntakibazo kibirimo

Title: Mulinga boys.docx

Doc Creator: tcuhawenimana11

Doc Date: 3/20/2023

Codes Applied: Sources of information received during puberty

Excerpt Creator: tcuhawenimana11

Excerpt Created On: 3/22/2023

Excerpt Range: 5645-5658

abantu bakuru

Title: Mulinga boys.docx

Doc Creator: tcuhawenimana11

Doc Date: 3/20/2023

Codes Applied: How boys experience physiological changes that occured to them during puberty

Excerpt Creator: tcuhawenimana11

Excerpt Created On: 3/22/2023

Excerpt Range: 5782-6117

Nange ikibazo cyambayeho nukwiroteraho nararyamye mugitondo mbyutse mbona amazi mu buriri ngirango nanyaye kubuririri mbaza murumuna wange nti se wigeze ubona nyara kuburiri? Ngo oya ngo ibyo ni ibintu bisanzwe ngo wiroteyeho ndamubaza ngo ubuse nakwitwara gute arambwira ngo ubwo wakuze ngo nukujya witwara neza ntukagire ihungabana

Title: Mulinga boys.docx

Doc Creator: tcuhawenimana11

Doc Date: 3/20/2023

Codes Applied: Sources of information received during puberty

Excerpt Creator: tcuhawenimana11

Excerpt Created On: 3/22/2023

Excerpt Range: 5902-5923

mbaza murumuna wange

Title: Mulinga boys.docx

Doc Creator: tcuhawenimana11

Doc Date: 3/20/2023

Codes Applied: How boys experience physiological changes that occured to them during puberty

Excerpt Creator: tcuhawenimana11

Excerpt Created On: 3/22/2023

Excerpt Range: 6184-6283

Ibyambayeho ngewe nukwiroteraho ariko kwiroteraho bakaba barabimbwiye rero ntakibazo nigeze ngira.

Title: Mulinga boys.docx

Doc Creator: tcuhawenimana11

Doc Date: 3/20/2023

Codes Applied: Information that girls need

Excerpt Creator: tcuhawenimana11

Excerpt Created On: 3/22/2023

Excerpt Range: 6576-6648

Amakuru yahabwa yagisha inama abantu bakuru bakamugira inama akababaza,

Title: Mulinga boys.docx

Doc Creator: tcuhawenimana11

Doc Date: 3/20/2023

Codes Applied: Information that girls need

Excerpt Creator: tcuhawenimana11

Excerpt Created On: 3/22/2023

Excerpt Range: 6752-6862

Amakuru yahabwa kubera ibi bintu yaba abibonye nk’ibimutunguye bayamuha neza bakamubwira n’uburyo yabyirindamo

Title: Mulinga boys.docx

Doc Creator: tcuhawenimana11

Doc Date: 3/20/2023

Codes Applied: Information that girls need

Excerpt Creator: tcuhawenimana11

Excerpt Created On: 3/22/2023

Excerpt Range: 7048-7341

Amakuru umwana wumukobwa yakenera nubujyanama ku babyeyi be cyangwa kugana ikigonderamubizama bakamugira inama kubyerekeranye n’ibyamubayeho nko kuzana ibiheri. Hari igihe bamwe bakubwira ngo ukore imibonanompuzabitsina ngo nibwo bigenda ariko nyine bakamugira inama bakamubwira yuko ataribyo.

Title: Mulinga boys.docx

Doc Creator: tcuhawenimana11

Doc Date: 3/20/2023

Codes Applied: Information that girls need

Excerpt Creator: tcuhawenimana11

Excerpt Created On: 3/22/2023

Excerpt Range: 7442-7681

Amakuru yakenera ashobora kuba ku kigo wenda yabaza n’abarimu bakamubwira ati mugihe wenda yaba yagiye nko mu mihanga akaba atabizi akaba wenda aziko yakomeretse ku kigo abarimu bashobora kubimubwira bakamuhugura akaza kubimenya atarabizi.

Title: Mulinga boys.docx

Doc Creator: tcuhawenimana11

Doc Date: 3/20/2023

Codes Applied: Information that girls need

Excerpt Creator: tcuhawenimana11

Excerpt Created On: 3/22/2023

Excerpt Range: 7820-8005

Ndumva amakuru yakenera ari ukumenya ko kumera amabere ntakibazo kibrimo. Atari uburwayi kandi no kumera insya ko nta kibazo kibirimo n’ibindi byose byamubaho ko ari igihe aba agezemo.

Title: Mulinga boys.docx

Doc Creator: tcuhawenimana11

Doc Date: 3/20/2023

Codes Applied: Information that boys need

Excerpt Creator: tcuhawenimana11

Excerpt Created On: 3/22/2023

Excerpt Range: 8092-8281

Umwana ugeze mu gihe cy’ubwangavu amakuru agomba guhabwa nuko agomba kumenya ko yakuze ntazongere gukina n’abana kuko bashobora kugira ibindi bakubagana ugasanga arasamye akaba yakwirinda.

Title: Mulinga boys.docx

Doc Creator: tcuhawenimana11

Doc Date: 3/20/2023

Codes Applied: Information that girls need

Excerpt Creator: tcuhawenimana11

Excerpt Created On: 3/22/2023

Excerpt Range: 8093-8281

Umwana ugeze mu gihe cy’ubwangavu amakuru agomba guhabwa nuko agomba kumenya ko yakuze ntazongere gukina n’abana kuko bashobora kugira ibindi bakubagana ugasanga arasamye akaba yakwirinda.

Title: Mulinga boys.docx

Doc Creator: tcuhawenimana11

Doc Date: 3/20/2023

Codes Applied: Information that girls need

Excerpt Creator: tcuhawenimana11

Excerpt Created On: 3/22/2023

Excerpt Range: 8426-8632

Amakuru umwana w’umukobwa ugeze mu gihe cy’ubwangavu yahabwa n’ukumenya ko iyo yageze mu mihango agomba kwisukura akiyitaho kuburyo ashobora no kumenya ko wenda atakomeretse akabasha kuba yakwikorera isuku.

Title: Mulinga boys.docx

Doc Creator: tcuhawenimana11

Doc Date: 3/20/2023

Codes Applied: Information that girls need

Excerpt Creator: tcuhawenimana11

Excerpt Created On: 3/22/2023

Excerpt Range: 9058-9184

amakuru numva yahabwa nuko yazajya nyine agirira isuku nko kumera insya akazogosha ndetse n’incakwaha ayo niyo makuru namuha.

Title: Mulinga boys.docx

Doc Creator: tcuhawenimana11

Doc Date: 3/20/2023

Codes Applied: Information that girls need

Excerpt Creator: tcuhawenimana11

Excerpt Created On: 3/22/2023

Excerpt Range: 9242-9434

Ngewe amakuru akeneye nuko mu gihe yiroteyeho atagomba kugira ipfunwe ryuko wenda ashobora kuba yanyaye ku buriri akamenya amakuru yuko ari ibintu bimubayeho kuko yakuze ko ari ibintu bisanzwe

Title: Mulinga boys.docx

Doc Creator: tcuhawenimana11

Doc Date: 3/20/2023

Codes Applied: Information that boys need

Excerpt Creator: tcuhawenimana11

Excerpt Created On: 3/22/2023

Excerpt Range: 9242-9434

Ngewe amakuru akeneye nuko mu gihe yiroteyeho atagomba kugira ipfunwe ryuko wenda ashobora kuba yanyaye ku buriri akamenya amakuru yuko ari ibintu bimubayeho kuko yakuze ko ari ibintu bisanzwe

Title: Mulinga boys.docx

Doc Creator: tcuhawenimana11

Doc Date: 3/20/2023

Codes Applied: Information that boys need

Excerpt Creator: tcuhawenimana11

Excerpt Created On: 3/22/2023

Excerpt Range: 9518-9685

Amakuru akeneye umwana ugeze mu gihe cy’ubugimbi agomba kumenya ko yakuze umuntu wiroteyeho atera inda akamenya yuko ari ukwirindaa ntihagire umwana wabandi yendereza.

Title: Mulinga boys.docx

Doc Creator: tcuhawenimana11

Doc Date: 3/20/2023

Codes Applied: Information that boys need

Excerpt Creator: tcuhawenimana11

Excerpt Created On: 3/22/2023

Excerpt Range: 9852-10000

Umuhungu amakuru akeneye n’ukumenya yuko yakuze yakirinda imibonano mpuzabitsia ko ashobora yahura n’imbogamizi nyinshi nk’indwara nyinshi zikabije,

Title: Mulinga boys.docx

Doc Creator: tcuhawenimana11

Doc Date: 3/20/2023

Codes Applied: How to take care of themselves when phsyiological changes occur for boys

Excerpt Creator: tcuhawenimana11

Excerpt Created On: 3/22/2023

Excerpt Range: 10384-10467

Amakuru naba narabwiwe nuko nakwigirira isuku ku mpinduka zangezeho zose zubugimbi,

Title: Mulinga boys.docx

Doc Creator: tcuhawenimana11

Doc Date: 3/20/2023

Codes Applied: Precautions to take during puberty for boys

Excerpt Creator: tcuhawenimana11

Excerpt Created On: 3/22/2023

Excerpt Range: 10589-10703

Inama nahawe n’ukwirinda nkareka gukubagana nkareka kugendana n’abana batoya kuko nakuze ntakiri mu rugero rwabo.

Title: Mulinga boys.docx

Doc Creator: tcuhawenimana11

Doc Date: 3/20/2023

Codes Applied: How to take care of themselves when phsyiological changes occur for boys

Excerpt Creator: tcuhawenimana11

Excerpt Created On: 3/22/2023

Excerpt Range: 10868-10934

Harimo kumbwira ko kuniga ijwi Atari ubrwayi kandi nkanagira isuku

Title: Mulinga boys.docx

Doc Creator: tcuhawenimana11

Doc Date: 3/20/2023

Codes Applied: Precautions to take during puberty for boys

Excerpt Creator: tcuhawenimana11

Excerpt Created On: 3/22/2023

Excerpt Range: 11022-11190

Amakuru nahawe nabwiwe yuko nakuze umuntu wiroteye ko iyo akoze imibonano n’umukobwa wagiye mu mihango atera inda bambwiye ko rero arukwirinda ibyo bifite igihe cyabyo.

Title: Mulinga boys.docx

Doc Creator: tcuhawenimana11

Doc Date: 3/20/2023

Codes Applied: Precautions to take during puberty for boys

Excerpt Creator: tcuhawenimana11

Excerpt Created On: 3/22/2023

Excerpt Range: 11297-11536

ahawe amakuru ko umwana wamaze kwiroteraho yakora imibonano mpuzabitsina n’umwana w’umukobwa nawe wageze mu gihe cyo kugera mu mihango ashobora kumutera inda bityo umwana w’umuhungu akifata kwifata byamunanira akaba yakoresha agakingirizo.

Title: Mulinga boys.docx

Doc Creator: tcuhawenimana11

Doc Date: 3/20/2023

Codes Applied: Precautions to take during puberty for boys

Excerpt Creator: tcuhawenimana11

Excerpt Created On: 3/22/2023

Excerpt Range: 11613-11728

Amakuru nahawe n’ukumenya kwirinda kuba nakora imibonano mpuzabitsinda nayikora nkayikora nkoresheje agakingirizo,

Title: Mulinga boys.docx

Doc Creator: tcuhawenimana11

Doc Date: 3/20/2023

Codes Applied: Precautions to take during puberty for boys

Excerpt Creator: tcuhawenimana11

Excerpt Created On: 3/22/2023

Excerpt Range: 11826-11932

Amakuru nahawe nuko umwana w’umuhungu wamaze kwiroteraho aba ageze mu gihe cy’ubugimbi aba atakiri umwana.

Title: Mulinga boys.docx

Doc Creator: tcuhawenimana11

Doc Date: 3/20/2023

Codes Applied: Precautions to take during puberty for boys

Excerpt Creator: tcuhawenimana11

Excerpt Created On: 3/22/2023

Excerpt Range: 12182-12421

Nge amakuru naba narahawe nuko umuhungu uba yariroteyeho aba yarakuze yakora imibonanompuzabitsina n’umukobwa wagiye mu mihango, idakingire bagaterana inda ariko amakuru nahawe nuko umuntu wameze insya aba yakuze n’umukobwa wameze amabere.

Title: Mulinga boys.docx

Doc Creator: tcuhawenimana11

Doc Date: 3/20/2023

Codes Applied: Sources of information received during puberty

Excerpt Creator: tcuhawenimana11

Excerpt Created On: 3/22/2023

Excerpt Range: 12814-12891

Ahantu twakuye amakuru twayakuye ku mashuri tubaza ababyeyi tubaza n’inshuti.

Title: Mulinga boys.docx

Doc Creator: tcuhawenimana11

Doc Date: 3/20/2023

Codes Applied: Sources of information received during puberty

Excerpt Creator: tcuhawenimana11

Excerpt Created On: 3/22/2023

Excerpt Range: 12961-13130

Ahantu nge naba narakuye amakuru ni ku babyeyi kuri bakuru bange ku nshuti zange cyangwa se no kuma radiyo hari ibiganiro bijya bicaho cyangwa se amakinamico nk’urunana.

Title: Mulinga boys.docx

Doc Creator: tcuhawenimana11

Doc Date: 3/20/2023

Codes Applied: Sources of information received during puberty

Excerpt Creator: tcuhawenimana11

Excerpt Created On: 3/22/2023

Excerpt Range: 13205-13327

Nge amakuru ahantu nayakuye ni muri club tubamo nyine mu mudugudu. Mu rugo abakuru ba club yacu nibo batubwira ayo makuru,

Title: Mulinga boys.docx

Doc Creator: tcuhawenimana11

Doc Date: 3/20/2023

Codes Applied: Sources of information received during puberty

Excerpt Creator: tcuhawenimana11

Excerpt Created On: 3/22/2023

Excerpt Range: 13398-13503

Ahantu amakuru nayakuye n’abarezi bo kw’ishuri bo club hari igihe batuganiriza ku buzima bw’imyororokere.

Title: Mulinga boys.docx

Doc Creator: tcuhawenimana11

Doc Date: 3/20/2023

Codes Applied: Sources of information received during puberty

Excerpt Creator: tcuhawenimana11

Excerpt Created On: 3/22/2023

Excerpt Range: 13577-13762

gewe aho nakuye amakuru hari abantu nyine bavaga kubigonderabuzima bakaza hano kw’ishuri bakayatubwira rimwe na rimwe nkayakura mu babyeyi no muri bakuru bange aho niho naba narayakuye.

Title: Mulinga boys.docx

Doc Creator: tcuhawenimana11

Doc Date: 3/20/2023

Codes Applied: Sources of information received during puberty

Excerpt Creator: tcuhawenimana11

Excerpt Created On: 3/22/2023

Excerpt Range: 13838-13946

Ngewe aho nakuraga amakuru ni mu babyeyi mu barimu no kumaradiyo, kumakinamico nkayumva nkumvamo ayo makuru.

Title: Mulinga boys.docx

Doc Creator: tcuhawenimana11

Doc Date: 3/20/2023

Codes Applied: Sources of information received during puberty

Excerpt Creator: tcuhawenimana11

Excerpt Created On: 3/22/2023

Excerpt Range: 14029-14125

Nge ubwa mbere niroteraho bwarakeye njya kumujyanama w’ubuzima mu mudugudu niwe wampaye amakuru.

Title: Mulinga boys.docx

Doc Creator: tcuhawenimana11

Doc Date: 3/20/2023

Codes Applied: Obstacles and risks experienced by boys during the sexual and reproductive health changes occuring during puberty and adolescence

Excerpt Creator: tcuhawenimana11

Excerpt Created On: 3/22/2023

Excerpt Range: 14548-14876

Ingorane umwana w’umuhungu ashobora guhura nazo ageze mu gihe cy’ubugimbi hari igihe ashobora kugira amarari y’ubusambanyi akaba yasambana n’umukobwa nawe ugeze mu gihe cy’ubwangavu bagakora imobonanompuzabitsina idakingiye cyangwa badakoresheje agakingirizo umukobwa ugasanga arasamye umuhungu bikamuviramo guhanishwa gufungwa.

Title: Mulinga boys.docx

Doc Creator: tcuhawenimana11

Doc Date: 3/20/2023

Codes Applied: Obstacles and risks experienced by boys during the sexual and reproductive health changes occuring during puberty and adolescence

Excerpt Creator: tcuhawenimana11

Excerpt Created On: 3/22/2023

Excerpt Range: 14922-15171

Ingorane umwana w’umuhungu yahura nazo ashobora kuba yarahuye na group mbi abantu bamwoshya mu biyobyabwenge bikaba byamutera gusambana agakora imibonanompuzabitsina idakingiye akaba yatera umukobwa inda cyangwa akandura indwara zigiye zitandukanye.

Title: Mulinga boys.docx

Doc Creator: tcuhawenimana11

Doc Date: 3/20/2023

Codes Applied: Obstacles and risks experienced by boys during the sexual and reproductive health changes occuring during puberty and adolescence

Excerpt Creator: tcuhawenimana11

Excerpt Created On: 3/22/2023

Excerpt Range: 15240-15380

Umwana w’umuhungu ingorane ashobora kuba yahura nazo nuko group abamo cyangwa ni nk’udutego yagwano ugasanga ateye inda mu gihe cy’ubugimbi.

Title: Mulinga boys.docx

Doc Creator: tcuhawenimana11

Doc Date: 3/20/2023

Codes Applied: Obstacles and risks experienced by boys during the sexual and reproductive health changes occuring during puberty and adolescence

Excerpt Creator: tcuhawenimana11

Excerpt Created On: 3/22/2023

Excerpt Range: 15457-15819

Umwana wumuhungu imbogamizi yahura nazo ashobora kugenda yahura n’umukobwa ari chr we hanyuma agatangira akamutereta maze ubundi bakajya gukora imibonanompuzabitsina umukobwa akaba yibagiwe ko bakoresha agakingirizo bose babyibagiwe bakaba bakora imibonanompuzabitsina idakingiye umukobwa akaba yasama agasama inda hanyuma umuhungu nawe agafungwa bitari ngombwa.

Title: Mulinga boys.docx

Doc Creator: tcuhawenimana11

Doc Date: 3/20/2023

Codes Applied: Obstacles and risks experienced by boys during the sexual and reproductive health changes occuring during puberty and adolescence

Excerpt Creator: tcuhawenimana11

Excerpt Created On: 3/22/2023

Excerpt Range: 15901-16066

Imbogamizi umuhungu yahura nazo ni nko kwishora mu biyobyabwenge n’ubusambanyi akaba yareka inshuri cyangwa se bakamubeshya ko yakuze akareka ishuri akava no murugo.

Title: Mulinga boys.docx

Doc Creator: tcuhawenimana11

Doc Date: 3/20/2023

Codes Applied: Obstacles and risks experienced by boys during the sexual and reproductive health changes occuring during puberty and adolescence

Excerpt Creator: tcuhawenimana11

Excerpt Created On: 3/22/2023

Excerpt Range: 16150-16456

mwana w’umuhungu ugeze mu gihe cy’ubugimbi aba yumva yakuze agatangira kuzana amaraso ya gisore niho biva ugasanga umuntu w’umwana aramwendereje aramukubise ubwo burya atangira kumva ko yakuze atangira gutereta ugasanga bimuviriyemo gutera inda noneho agatangira gushakishwa nikigo gishinzwe ubugenzacyaha.

Title: Mulinga boys.docx

Doc Creator: tcuhawenimana11

Doc Date: 3/20/2023

Codes Applied: Obstacles and risks experienced by girls during sexual and reproductive health changes occuring during puberty and adolescence

Excerpt Creator: tcuhawenimana11

Excerpt Created On: 3/22/2023

Excerpt Range: 16770-16992

nkaba numva ingorane umwana w’umukobwa ugeze mu gihe cy’ubwangavu yagira ari nkuko yaba yagiye mu mihango yahita ahura n’umuhungu bagakora imibonanompuzabitsina yahita asama bikamuviramo ingaruka mu babyeyi no mu bayobozi,

Title: Mulinga boys.docx

Doc Creator: tcuhawenimana11

Doc Date: 3/20/2023

Codes Applied: Obstacles and risks experienced by girls during sexual and reproductive health changes occuring during puberty and adolescence

Excerpt Creator: tcuhawenimana11

Excerpt Created On: 3/22/2023

Excerpt Range: 17117-17317

Ingaruka umukobwa yahura nazo ashobora kujya mu mihango akabyibagirwa yahura n’umusore akamutera inda bikamuvira kureka amashuri kwiga akabireka ntiyibuke ko yateye inda cyangwa bikamuviramo n’urupfu,

Title: Mulinga boys.docx

Doc Creator: tcuhawenimana11

Doc Date: 3/20/2023

Codes Applied: Obstacles and risks experienced by girls during sexual and reproductive health changes occuring during puberty and adolescence

Excerpt Creator: tcuhawenimana11

Excerpt Created On: 3/22/2023

Excerpt Range: 17374-17547

Ingorane umukobwa yagira nuko yaba yaragiye mu mihango yahuye na cher we barakundanye yarangiza akamushuka bagakora imibonanompuzabitsina bayikora idakingiye akamutera inda.

Title: Mulinga boys.docx

Doc Creator: tcuhawenimana11

Doc Date: 3/20/2023

Codes Applied: Obstacles and risks experienced by girls during sexual and reproductive health changes occuring during puberty and adolescence

Excerpt Creator: tcuhawenimana11

Excerpt Created On: 3/22/2023

Excerpt Range: 17600-17831

Ingorane umwana w’umukobwa ugeze mu gihe cy’ubwangavu yahura nazo harimo kujya mu bishuko akajya mu kigare kibi agatangira akajya murubwo burayi akajya mu myuga nyine itamuhesha ishema akaba ariho biva rero basenya abana babakobwa.

Title: Mulinga boys.docx

Doc Creator: tcuhawenimana11

Doc Date: 3/20/2023

Codes Applied: Obstacles and risks experienced by girls during sexual and reproductive health changes occuring during puberty and adolescence

Excerpt Creator: tcuhawenimana11

Excerpt Created On: 3/22/2023

Excerpt Range: 18166-18338

inzitizi umukobwa yagira ni nkuko yaba afite ababyeyi ntiyicarane nabo ngo bamugire inama cyangwa ntagire inshuti zimugira inama ngo ave mu ngeso mbi bikamugirira ingaruka.

Title: Mulinga boys.docx

Doc Creator: tcuhawenimana11

Doc Date: 3/20/2023

Codes Applied: SRH challenges girls meet during their puberty

Excerpt Creator: tcuhawenimana11

Excerpt Created On: 3/22/2023

Excerpt Range: 18405-18533

Inzitizi umwana w’umukobwa cyamgwa w’umuhungu ashobora guhura nazo n’ukuba atabona abantu bamuganiriza ku buzima bwimyororokere.

Title: Mulinga boys.docx

Doc Creator: tcuhawenimana11

Doc Date: 3/20/2023

Codes Applied: SRH challenges girls meet during their puberty

Excerpt Creator: tcuhawenimana11

Excerpt Created On: 3/22/2023

Excerpt Range: 18620-18794

Inzitizi yahura nazo nuko ashobora gukurira mu miryango atabana nababyeyi be ntibamuhe amakuru ajyanye n’imyororokere bigatuma ajya mu ngeso mbi kubera ko atabanye nababyeyi,

Title: Mulinga boys.docx

Doc Creator: tcuhawenimana11

Doc Date: 3/20/2023

Codes Applied: SRH challenges girls meet during their puberty

Excerpt Creator: tcuhawenimana11

Excerpt Created On: 3/22/2023

Excerpt Range: 18882-19190

Inzitizi umwana w’umuhungu cyangwa umukobwa yahura nazo nuko yaba yaravukiye mu miryango ikennye akabura ubushobozi bwo kubonana n’ababyeyi be bakajya baganira cyangwa nawe kuba yatera ikirenge ngo abe yagera kukigonderabuzima aganire n’abajyanama bityo akabura amakuru kubijyanye nubuzima bw’imyirorokerere.

Title: Mulinga boys.docx

Doc Creator: tcuhawenimana11

Doc Date: 3/20/2023

Codes Applied: SRH challenges girls meet during their puberty

Excerpt Creator: tcuhawenimana11

Excerpt Created On: 3/22/2023

Excerpt Range: 19272-19534

Inzitizi ingimbi cyangwa abangavu bahura nazo nukutamenya amakuru ku buzima bw’imyororokere cyangwa se ngo babisobanukirwe neza ko gukora imibonanompuzabitsina idakingiye ushobora gukuramo inda zitateganyijwe cyangwa se indwara zandurira mu mibonanompuzabitsina.

Title: Mulinga boys.docx

Doc Creator: tcuhawenimana11

Doc Date: 3/20/2023

Codes Applied: Suggestions to improve SRH during puberty and adolescence by boys

Excerpt Creator: tcuhawenimana11

Excerpt Created On: 3/22/2023

Excerpt Range: 19721-19876

Ngewe ikintu numva navuga nuko ama club yaba menshi mu midugudu abana bakabona ahantu bajya bajya gufata inyigisho ku bijyanye no kubuzima bw’imyororokere,

Title: Mulinga boys.docx

Doc Creator: tcuhawenimana11

Doc Date: 3/20/2023

Codes Applied: Suggestions to improve SRH during puberty and adolescence by boys

Excerpt Creator: tcuhawenimana11

Excerpt Created On: 3/22/2023

Excerpt Range: 19968-20156

Ikintu navugaho umuhungu cyangwa umukobwa bageze mu gihe cy’ubwangavu cyangwa ubugimbi yazajya yegera abantu bakuru bamuruseho cyangwa abarezi bose bakamugira inama kubyo nyine aba arimo.

Title: Mulinga boys.docx

Doc Creator: tcuhawenimana11

Doc Date: 3/20/2023

Codes Applied: Suggestions to improve SRH during puberty and adolescence by boys

Excerpt Creator: tcuhawenimana11

Excerpt Created On: 3/22/2023

Excerpt Range: 20235-20331

Icyo numva nabivugaho nuko bakongera ibiganiro kuri radiyo zose bivuga ku buzima bw’imyororokere

Title: Mulinga boys.docx

Doc Creator: tcuhawenimana11

Doc Date: 3/20/2023

Codes Applied: Suggestions to improve SRH during puberty and adolescence by boys

Excerpt Creator: tcuhawenimana11

Excerpt Created On: 3/22/2023

Excerpt Range: 20432-20623

Nge icyo nabivugaho nugushishikariza ababyeyi, abarezi nabakuru kuganira n’abana babo bakababwira nabo ko hari ibihe baciyemo kandi nabo bakababwira ko bazabicamo bakabaha amakuru abanogeye.

Title: Mulinga boys.docx

Doc Creator: tcuhawenimana11

Doc Date: 3/20/2023

Codes Applied: Suggestions to improve SRH during puberty and adolescence by boys

Excerpt Creator: tcuhawenimana11

Excerpt Created On: 3/22/2023

Excerpt Range: 20724-20878

Nge inama nagira abakobwa n’abahungu bageze mu gihe cy’ubwangavu n’ubugimbi n’ukwegera ababyeyi bakababwira ukuntu ibintu biba bigenda barangiza bakifata.

Title: G S KAYONZA BOYS Transcri.docx

Doc Creator: tcuhawenimana11

Doc Date: 3/20/2023

Codes Applied: Physiological changes occuring during puberty-Boys

Excerpt Creator: tcuhawenimana11

Excerpt Created On: 3/21/2023

Excerpt Range: 673-866

impinduka abona nukumera incakwaha, kumera insya kwiroteraho nibindi bigeye bitandukanye nko kuniga ijwi kwaguka mu gatuza nyine ukabona we yarahindutse, kumera ibishishi nibyo ngibyo murakoze.

Title: G S KAYONZA BOYS Transcri.docx

Doc Creator: tcuhawenimana11

Doc Date: 3/20/2023

Codes Applied: Psychological changes occuring during puberty-Boys

Excerpt Creator: tcuhawenimana11

Excerpt Created On: 3/21/2023

Excerpt Range: 881-1185

Izindi mpinduka umuntu agira ageze mu gihe cy’ubugimbi iyo ubonye umukobwa nyine hari igihe umwifuza ukumva ushaka gukora imobonanompuzabitsina, atangira no kugenda yaguka mu bitekerezo uko atekereza biba bitandukanye nuko yatekerezaga akiri umwana, agatangira kugenda avuga ngo uriya mukobwa ateye neza.

Title: G S KAYONZA BOYS Transcri.docx

Doc Creator: tcuhawenimana11

Doc Date: 3/20/2023

Codes Applied: Physiological changes occuring during puberty-Boys

Excerpt Creator: tcuhawenimana11

Excerpt Created On: 3/21/2023

Excerpt Range: 1259-1295

Nukumera ubwanwa ariko ntibirambaho.

Title: G S KAYONZA BOYS Transcri.docx

Doc Creator: tcuhawenimana11

Doc Date: 3/20/2023

Codes Applied: Physiological changes occuring during puberty-Boys

Excerpt Creator: tcuhawenimana11

Excerpt Created On: 3/21/2023

Excerpt Range: 1358-1445

Ni ibihe bimenyetso bikugaragariza ko wageze muri adolescence? Kumera incakwaha ninsya

Title: G S KAYONZA BOYS Transcri.docx

Doc Creator: tcuhawenimana11

Doc Date: 3/20/2023

Codes Applied: Psychological changes occuring during puberty-Boys

Excerpt Creator: tcuhawenimana11

Excerpt Created On: 3/21/2023

Excerpt Range: 1604-1664

Ntago uko narimeze nyine ariko nkimeze ubu, byarahindutse.

Title: G S KAYONZA BOYS Transcri.docx

Doc Creator: tcuhawenimana11

Doc Date: 3/20/2023

Codes Applied: Psychological changes occuring during puberty-Boys

Excerpt Creator: tcuhawenimana11

Excerpt Created On: 3/21/2023

Excerpt Range: 1754-1795

uko nitwaraga mbere ntago ariko nkitwara.

Title: G S KAYONZA BOYS Transcri.docx

Doc Creator: tcuhawenimana11

Doc Date: 3/20/2023

Codes Applied: Precautions to take during puberty for boys

Excerpt Creator: tcuhawenimana11

Excerpt Created On: 3/21/2023

Excerpt Range: 1956-2485

Mpereye kucyo yarariho kwigengesera n’ukwigengesera muri buri kintu cyose ugiyemo cyangwa se ugiye gukora kugirango abakuri inyuma cyangwa abakubona, ababyeyi bawe baterwe ishema nuko wavuye mu kigero cyo kwitwa umwana ukagera mu kigero kwitwa ingimbi. Kuba uri ingimbi bisaba yuko na barumuna bawe bakuri inyuma bazajya bavuga ngo mukuru wacu twabonaga yitwara gutya, yasubizaga gutya ababyeyi, yagendaga gutya mubyo yajyaga gukora byose niyo mpamvu kuba uvuye mu kigero cyo kwitwa umwana ukitwa ingimbi bisaba ubuhanga bwinshi.

Title: G S KAYONZA BOYS Transcri.docx

Doc Creator: tcuhawenimana11

Doc Date: 3/20/2023

Codes Applied: Physiological changes occuring during puberty-Boys

Excerpt Creator: tcuhawenimana11

Excerpt Created On: 3/21/2023

Excerpt Range: 2604-2672

Icyambere uhindura ijwi n’ibitekerezo bikaguka no mu maso hakaguka,

Title: G S KAYONZA BOYS Transcri.docx

Doc Creator: tcuhawenimana11

Doc Date: 3/20/2023

Codes Applied: Psychological changes occuring during puberty-Boys

Excerpt Creator: tcuhawenimana11

Excerpt Created On: 3/21/2023

Excerpt Range: 2739-2978

Ikintu cya mbere umuhungu abanza kugira iyo ageze muricyo kigero aba yumva yuko buri mukobwa wese yamurongora, icya kabiri aba yumva ko buri kintu cyose yagishobora kubera yuko nicyo gihe aba yumva afite imbaraga kurusha cya gihe cyubwana.

Title: G S KAYONZA BOYS Transcri.docx

Doc Creator: tcuhawenimana11

Doc Date: 3/20/2023

Codes Applied: Psychological changes occuring during puberty-Boys

Excerpt Creator: tcuhawenimana11

Excerpt Created On: 3/21/2023

Excerpt Range: 3137-3383

Imyitwarire yabahungu bamaze kugera mu gihe cy’ubugimbi intekerezo ze zirahinduka niyo yabona nk’umukobwa mwiza cyangwa bavugana ugasanga araye amutekereza avuga ati uriya mukobwa namubona gute cyangwa ugasanga arimo kwifuza nabandi bakobwa bose.

Title: G S KAYONZA BOYS Transcri.docx

Doc Creator: tcuhawenimana11

Doc Date: 3/20/2023

Codes Applied: Psychological changes occuring during puberty-Boys

Excerpt Creator: tcuhawenimana11

Excerpt Created On: 3/21/2023

Excerpt Range: 3572-3689

Iyo atamubonye nawe ashaka uburyo amutereta icyo gihe nawe kugucika sinzi cyangwa ukihangana ugapfira muri nyagasani.

Title: G S KAYONZA BOYS Transcri.docx

Doc Creator: tcuhawenimana11

Doc Date: 3/20/2023

Codes Applied: Received information is right and provides sufficient information helping boys and girls during puberty

Excerpt Creator: tcuhawenimana11

Excerpt Created On: 3/21/2023

Excerpt Range: 4186-4339

Iri somo twizemo, twiga nk’umukobwa uburyo mwakora imibonanomuzabitsina ariko ntumutere inda cyangwa se ntugire indwara wandura bitewe nuburyo wifashije.

Title: G S KAYONZA BOYS Transcri.docx

Doc Creator: tcuhawenimana11

Doc Date: 3/20/2023

Codes Applied: How to take care of themselves when phsyiological changes occur for boys

Excerpt Creator: tcuhawenimana11

Excerpt Created On: 3/21/2023

Excerpt Range: 4413-4489

Twize amazina y’imyanya y’igitsina cy’abakobwa twiga nuko twayikorera isuku.

Title: G S KAYONZA BOYS Transcri.docx

Doc Creator: tcuhawenimana11

Doc Date: 3/20/2023

Codes Applied: Precautions to take during puberty for boys

Excerpt Creator: tcuhawenimana11

Excerpt Created On: 3/21/2023

Excerpt Range: 4508-4647

Twize wenda n’ukuntu ugiye gukora imibonanomuzabitsina ukuntu wakwirinda kumutera inda cyanwa ukirinda indwara dukoresheje nkagakingirizo.

Title: G S KAYONZA BOYS Transcri.docx

Doc Creator: tcuhawenimana11

Doc Date: 3/20/2023

Codes Applied: Received information is right and provides sufficient information helping boys and girls during puberty

Excerpt Creator: tcuhawenimana11

Excerpt Created On: 3/21/2023

Excerpt Range: 4740-4792

P6 twizemo indwara zandurira mu mibonanomuzabitsina.

Title: G S KAYONZA BOYS Transcri.docx

Doc Creator: tcuhawenimana11

Doc Date: 3/20/2023

Codes Applied: Precautions to take during puberty for girls

Excerpt Creator: tcuhawenimana11

Excerpt Created On: 3/21/2023

Excerpt Range: 4844-5225

Twebwe twizemo twize cyane cyane imyororokere y’abakobwa kubera ko guhera mu minsi 25 ya mbere umukobwa cyangwa igitsina gore cyose kigomba kuba cyageze mu gihe bita icyo kujya mu mihango cyangwa gusohora imihango. Guhera kw’itariki yambere y’ukwezi kwa cyenda urugero kugeza kwitariki makumyabiri igitsina gore cyose kigomba kuba cyarageze mu gihe cyo kuba cya producinze imihango

Title: G S KAYONZA BOYS Transcri.docx

Doc Creator: tcuhawenimana11

Doc Date: 3/20/2023

Codes Applied: Boys and girls know about the causes of the causes leading to the physiological changes during puberty

Excerpt Creator: tcuhawenimana11

Excerpt Created On: 3/21/2023

Excerpt Range: 5380-5535

Ntakindi muri bwongereho nikigero mwari mugezemo tu! Imana yararebye ibona aba bana bageze muri imyaka yayindi ihita ivuga ngo aba bana nibamere ubwanwa?

Title: G S KAYONZA BOYS Transcri.docx

Doc Creator: tcuhawenimana11

Doc Date: 3/20/2023

Codes Applied: Physiological changes occuring during puberty-Girls

Excerpt Creator: tcuhawenimana11

Excerpt Created On: 3/21/2023

Excerpt Range: 5670-5872

Murakoze, umwana w’umukobwa we atangira kumera amabere, agatangira kugira size nini cyangwa se ama hips Manini akazana buriya nayita iki ra? Mu Kinyarwanda atangira kuzana amataye ndumva ariko babyita.

Title: G S KAYONZA BOYS Transcri.docx

Doc Creator: tcuhawenimana11

Doc Date: 3/20/2023

Codes Applied: Psychological changes occuring during puberty-Girls

Excerpt Creator: tcuhawenimana11

Excerpt Created On: 3/21/2023

Excerpt Range: 5872-6018

Atangira nyine kubona nawe ashaka abahungu cyane adashaka kuguma mu bakobwa bagenzi be iyo ageze mu kigero cy’imyaka cumi n’itanu cumi n’itandatu.

Title: G S KAYONZA BOYS Transcri.docx

Doc Creator: tcuhawenimana11

Doc Date: 3/20/2023

Codes Applied: Physiological changes occuring during puberty-Girls

Excerpt Creator: tcuhawenimana11

Excerpt Created On: 3/21/2023

Excerpt Range: 6042-6184

Nawe atangira kuzana incakwaha, insya agatangira no kugenda abyibuha muri size akanagabanuka ijwi rigenda rimeze nkaho ari gato gato. Akajwi

Title: G S KAYONZA BOYS Transcri.docx

Doc Creator: tcuhawenimana11

Doc Date: 3/20/2023

Codes Applied: Physiological changes occuring during puberty-Girls

Excerpt Creator: tcuhawenimana11

Excerpt Created On: 3/21/2023

Excerpt Range: 6259-6291

Ikindi ashobora no gusama inda.

Title: G S KAYONZA BOYS Transcri.docx

Doc Creator: tcuhawenimana11

Doc Date: 3/20/2023

Codes Applied: Physiological changes occuring during puberty-Girls

Excerpt Creator: tcuhawenimana11

Excerpt Created On: 3/21/2023

Excerpt Range: 6306-6385

We akoze imibonanomuzabitsina ari mu mihago cyangwa amaze iminsi mike ayivuyemo

Title: G S KAYONZA BOYS Transcri.docx

Doc Creator: tcuhawenimana11

Doc Date: 3/20/2023

Codes Applied: Physiological changes occuring during puberty-Girls

Excerpt Creator: tcuhawenimana11

Excerpt Created On: 3/21/2023

Excerpt Range: 6444-6577

Ikindi kiyongera nge nakongeraho ku minduka ziba ku mukobwa yaba mu minduka ziri physical, cyangwa agatangira kuba agira isuku cyane.

Title: G S KAYONZA BOYS Transcri.docx

Doc Creator: tcuhawenimana11

Doc Date: 3/20/2023

Codes Applied: Psychological changes occuring during puberty-Boys

Excerpt Creator: tcuhawenimana11

Excerpt Created On: 3/21/2023

Excerpt Range: 6749-7051

Aba ashaka kujya ahantu hari abahungu benshi yumva muri we akunze abahungu. Buriya se kuki aba yumva abakunze? Nukubera ikigero cy’imyaka aba agezemo. Nonese ko hari abakobwa bagera muri icyo gihe ntibashake kuba bari aho abahungu bari? Ariko nawe mu ntekerezo nawe buriya aba afite ukuntu abatekereza.

Title: G S KAYONZA BOYS Transcri.docx

Doc Creator: tcuhawenimana11

Doc Date: 3/20/2023

Codes Applied: Psychological changes occuring during puberty-Girls

Excerpt Creator: tcuhawenimana11

Excerpt Created On: 3/21/2023

Excerpt Range: 7200-7263

Umukobwa ikintu aba yumva ashaka gukora imibonanompuzabitsina.

Title: G S KAYONZA BOYS Transcri.docx

Doc Creator: tcuhawenimana11

Doc Date: 3/20/2023

Codes Applied: Boys and girls know about the causes of the causes leading to the physiological changes during puberty

Excerpt Creator: tcuhawenimana11

Excerpt Created On: 3/21/2023

Excerpt Range: 7289-7319

Biba biterwa nikigero agezemo.

Title: G S KAYONZA BOYS Transcri.docx

Doc Creator: tcuhawenimana11

Doc Date: 3/20/2023

Codes Applied: How boys experience physiological changes that occured to them during puberty

Excerpt Creator: tcuhawenimana11

Excerpt Created On: 3/21/2023

Excerpt Range: 8074-8199

Ngewe kubera ko twari tumaze nigihe tubyiga nahise mbifata nkibisanzwe kubera ko twari twarabyize narimbizi ntago byanangaje.

Title: G S KAYONZA BOYS Transcri.docx

Doc Creator: tcuhawenimana11

Doc Date: 3/20/2023

Codes Applied: How boys experience physiological changes that occured to them during puberty

Excerpt Creator: tcuhawenimana11

Excerpt Created On: 3/21/2023

Excerpt Range: 8291-8397

nahise numva ko nyine navuye mu gihe cy’abana nagiye mu gihe cy’abakuru ahaa uvuze ikintu kitwe cyabakuru.

Title: G S KAYONZA BOYS Transcri.docx

Doc Creator: tcuhawenimana11

Doc Date: 3/20/2023

Codes Applied: How boys experience physiological changes that occured to them during puberty

Excerpt Creator: tcuhawenimana11

Excerpt Created On: 3/21/2023

Excerpt Range: 8774-8904

Nge byaranunguye usibye kontanumuntu nigeze mbaza ariko nyine nahisembifata nkibisanzwe numva ko ari ibintu bibaho kukiremwamuntu.

Title: G S KAYONZA BOYS Transcri.docx

Doc Creator: tcuhawenimana11

Doc Date: 3/20/2023

Codes Applied: How boys experience physiological changes that occured to them during puberty

Excerpt Creator: tcuhawenimana11

Excerpt Created On: 3/21/2023

Excerpt Range: 9019-9297

Ntago byashoboka ko ntahantu waba warabyumvishe ntago twese tugenda mubana, harigehe wagendaga mu basore ukumva baravuze ngo man uziko uyu munsi nogoshe insya ariko ziri kurya? So, rero ukaza gucengera ukabaza insya zibahe bakagusobanurira niyo mpamvu nge nabifashe nkibisanzwe.

Title: G S KAYONZA BOYS Transcri.docx

Doc Creator: tcuhawenimana11

Doc Date: 3/20/2023

Codes Applied: How boys experience physiological changes that occured to them during puberty

Excerpt Creator: tcuhawenimana11

Excerpt Created On: 3/21/2023

Excerpt Range: 9348-9443

Yego hafi ya yose narinyafite kuburyo ntahantu byigeze bingiraho shoke ya cyane murakoze cyane.

Title: G S KAYONZA BOYS Transcri.docx

Doc Creator: tcuhawenimana11

Doc Date: 3/20/2023

Codes Applied: How boys experience physiological changes that occured to them during puberty

Excerpt Creator: tcuhawenimana11

Excerpt Created On: 3/21/2023

Excerpt Range: 9515-9992

Nge bimbaho numvishe nshaka gukora imibonanompizabitsina. Byakubayeho wumva nyine wowe urashaka gukora imibonanomuzabitsina. Urabona nyine uraryama nyine uba warameze nkinsya ukabyuka wiroteyeho ukumva za nzozi wagize ni nziza ukavuga uti icyabingezaho. wabyitwayemo ute? Nyine icyo gihe bitewe nuburyo mwishuri twari twarabyize ubitekerezaho ukavuga uti uwatereta nkumukobwa ubundi tugakora imibonanompuzabitsina ko numva mbikeneye ugatangira kugenda ubeshya utwana twabandi.

Title: G S KAYONZA BOYS Transcri.docx

Doc Creator: tcuhawenimana11

Doc Date: 3/20/2023

Codes Applied: How boys experience physiological changes that occured to them during puberty

Excerpt Creator: tcuhawenimana11

Excerpt Created On: 3/21/2023

Excerpt Range: 10075-10156

Oya nyine nange icyo nsatse kuvuga nashatse kubigeragezaho gusa harukuntu nyine.

Title: G S KAYONZA BOYS Transcri.docx

Doc Creator: tcuhawenimana11

Doc Date: 3/20/2023

Codes Applied: How boys experience physiological changes that occured to them during puberty

Excerpt Creator: tcuhawenimana11

Excerpt Created On: 3/21/2023

Excerpt Range: 10181-10316

Oya ntago yanteye indobo nge twari twanemeranyije hari ukuntu uvugira nko kuri fone, hanyuma mwarimu mugisha aba arahageze arabatesha.

Title: G S KAYONZA BOYS Transcri.docx

Doc Creator: tcuhawenimana11

Doc Date: 3/20/2023

Codes Applied: Precautions to take during puberty for boys

Excerpt Creator: tcuhawenimana11

Excerpt Created On: 3/21/2023

Excerpt Range: 10359-10569

Ego njya ryumva ariko navugiye kuri fone mukuru wange aba aranyumvishe arabwira ngo ushobora kwandura ibi sida nage mbitekerejeho ndavuga nti nangiritse nkiri umwana nti ka byihorere nihangane negereze umogore.

Title: G S KAYONZA BOYS Transcri.docx

Doc Creator: tcuhawenimana11

Doc Date: 3/20/2023

Codes Applied: Received information is right and provides sufficient information helping boys and girls during puberty

Excerpt Creator: tcuhawenimana11

Excerpt Created On: 3/21/2023

Excerpt Range: 10359-10569

Ego njya ryumva ariko navugiye kuri fone mukuru wange aba aranyumvishe arabwira ngo ushobora kwandura ibi sida nage mbitekerejeho ndavuga nti nangiritse nkiri umwana nti ka byihorere nihangane negereze umogore.

Title: G S KAYONZA BOYS Transcri.docx

Doc Creator: tcuhawenimana11

Doc Date: 3/20/2023

Codes Applied: Sources of information received during puberty

Excerpt Creator: tcuhawenimana11

Excerpt Created On: 3/21/2023

Excerpt Range: 10400-10412

mukuru wange

Title: G S KAYONZA BOYS Transcri.docx

Doc Creator: tcuhawenimana11

Doc Date: 3/20/2023

Codes Applied: How boys experience physiological changes that occured to them during puberty

Excerpt Creator: tcuhawenimana11

Excerpt Created On: 3/21/2023

Excerpt Range: 10646-11062

Nge bimbaho nyine, ntago nahise niyumvisha ko nkuze ahubwo nshaka uko nzajya nirinda ibintu nkibyongibyo wakumva bikujemo ugashaka uko uhindura ibitekerezo ukabyikuramo. Byapfuye kukuzamo gutyo ngo ufate ingamba nkizongizo zikakaye kandi nawe uri umwana ugomba kurya show. Nyine twarabyize ngendeye kuko twabyize twakwirinda maze nange nshaka uko nazajya byirinda. Muri make nyine uhagaze bwuma, yee ntago ndabikora.

Title: G S KAYONZA BOYS Transcri.docx

Doc Creator: tcuhawenimana11

Doc Date: 3/20/2023

Codes Applied: How to take care of themselves when phsyiological changes occur for boys

Excerpt Creator: tcuhawenimana11

Excerpt Created On: 3/21/2023

Excerpt Range: 11291-11415

Amakuru agomba kumenya n’ukuntu agomba kwitwara muri icyo gihe cyimyaka agezemo nukuntu yajya yigirira isuku ubwe ku mubiri.

Title: G S KAYONZA BOYS Transcri.docx

Doc Creator: tcuhawenimana11

Doc Date: 3/20/2023

Codes Applied: Psychological changes occuring during puberty-Girls

Excerpt Creator: tcuhawenimana11

Excerpt Created On: 3/22/2023

Excerpt Range: 12212-13250

Umukobwa cyangwa se abakobwa bageze muri icyo kigero cy’imyaka cumi n’ibiri,n’itatu,n’ine n’itanu, kugeza kuri cumi n’imunani icyo gihe aba ataramenya kwifatira icyemezo cyicyo yakora amakuru aba akeneye ku buzima bwe ni ukumenya ukuntu azitwar mu gihe atereswe n’umuhungu nawe uri muru icyo kigero kuko usanga iyo umukobwa ageze muri icyi kigero aba afite icyo bita ubushyuhe kuberako araryama akarota ari kumwe n’umuhungu umuhungu nawe akaryama akarota ari kumwe n’umukobwa mbese ni icyo kigero tuba turimo ariko iyo abashije kubona amakuru akabona umuntu umubwira ati rero mwana wange iyo ugeze muri iyi myaka, umukobwa yirinda cyane umuhungu yirinda cyane kuganira ku.. cyane cyane ku myaka tuba tugezemo kuri icyo gihe usanga aricyo kenshi duterura cyane kuruta kuganira ku masomo, kuruta kuganira mu rugo tukaganira ku bijyanye n’imibonano mpuzabitsina kubera ko aribyo kenshi tuba turarikiye ariko iyo ababyeyi batubwiye ati rero bana bacu nibajya babaganiriza ibyongibyo Atari mwarimu ubibabwiye, mujye muva muri icyo kiganiro pe!

Title: G S KAYONZA BOYS Transcri.docx

Doc Creator: tcuhawenimana11

Doc Date: 3/20/2023

Codes Applied: Received information is misleading and risky

Excerpt Creator: tcuhawenimana11

Excerpt Created On: 3/22/2023

Excerpt Range: 13567-14057

Yego, arahari kuko natije mubyara wange fone, nyine kwakundi ujya kuru youtube uga searching ndayimuha ngo a searchinge arebe ibyo ashaka mugurira mega za 500 ndavuga ati sinkudabagije uno munsi? Ndamureka ngiye kureba ibyo ya searchinze yisearchingiraga uko umokobwa anyara igihe ari gukora imibonano mpuzabitsina, nyine ukareba ugasanga ibintu nkibyo nibyo yashakaga kwirebera, ashaka kwirebera za prono kandi kuri youtube ntibazitanga gusa ubimubwiye yarakara nayo makuru mfite yonyine.

Title: G S KAYONZA BOYS Transcri.docx

Doc Creator: tcuhawenimana11

Doc Date: 3/20/2023

Codes Applied: Sources of information received during puberty

Excerpt Creator: tcuhawenimana11

Excerpt Created On: 3/22/2023

Excerpt Range: 13612-13645

nyine kwakundi ujya kuru youtube

Title: G S KAYONZA BOYS Transcri.docx

Doc Creator: tcuhawenimana11

Doc Date: 3/20/2023

Codes Applied: Information that girls need

Excerpt Creator: tcuhawenimana11

Excerpt Created On: 3/22/2023

Excerpt Range: 14300-14476

Amakuru yose arayakeneye ariko akamenya uburyo abyitwaramo mugihe afita ayo makuru. Amakuru yingenzi akeneye kurubu, ayo akeneye ni agendany nimibonanaompuzabitsina uko ikorwa.

Title: G S KAYONZA BOYS Transcri.docx

Doc Creator: tcuhawenimana11

Doc Date: 3/20/2023

Codes Applied: Information that girls need

Excerpt Creator: tcuhawenimana11

Excerpt Created On: 3/22/2023

Excerpt Range: 14757-15077

Nkumugira inama. Nkumugura inama, ntago wamubuza nubundi kumenya ayo makuru wamureka akayamenya ariko mugihe amenye ayo makuru aritwara ate? Kuko hari nkumuntu w’umukobwa cyangwa w’umuhungu kwifata binanira ukamubwira ati niba byanze akira ubwirinzi ubikore ukamera nkumuhaye rugari kuko hari abantu badashobora kwifata.

Title: G S KAYONZA BOYS Transcri.docx

Doc Creator: tcuhawenimana11

Doc Date: 3/20/2023

Codes Applied: Information that girls need

Excerpt Creator: tcuhawenimana11

Excerpt Created On: 3/22/2023

Excerpt Range: 15208-15314

Amakuru aba akeneye ni nkukuntu yakwigirira isuku, akirinda abamushuka, bamushora mu mibonanompuzabitsina.

Title: G S KAYONZA BOYS Transcri.docx

Doc Creator: tcuhawenimana11

Doc Date: 3/20/2023

Codes Applied: Information that girls need

Excerpt Creator: tcuhawenimana11

Excerpt Created On: 3/22/2023

Excerpt Range: 15350-15600

Icyo nakongeraho nuko bakwigirira isuku bakirinda nababashuka. Ayo niyo makuru bajya babona? Wapi bakamenya no kwifata n’uko bibananiye babigenza niba bakoresha nudukingirizo kugira ngo badatwara inda cyanwa indwara zandurira mu mibonanompuzabitsina.

Title: G S KAYONZA BOYS Transcri.docx

Doc Creator: tcuhawenimana11

Doc Date: 3/20/2023

Codes Applied: Information that boys need

Excerpt Creator: tcuhawenimana11

Excerpt Created On: 3/22/2023

Excerpt Range: 15777-15840

Kwigirira isuku, no kwirinda kwishora mu mibonanompuzabitsina.

Title: G S KAYONZA BOYS Transcri.docx

Doc Creator: tcuhawenimana11

Doc Date: 3/20/2023

Codes Applied: Information that boys need

Excerpt Creator: tcuhawenimana11

Excerpt Created On: 3/22/2023

Excerpt Range: 15881-16094

Icyo navuga amakuru umuhungu aba akeneye ni ukuntu yakitwara muri icyo kigero agezemo akirinda wenda nubwo yaba ashaka gukura imibonanompuzabitsina wenda byanze kwifata, agashaka agakingirizo akaba ariko akoresha.

Title: G S KAYONZA BOYS Transcri.docx

Doc Creator: tcuhawenimana11

Doc Date: 3/20/2023

Codes Applied: Information that boys need

Excerpt Creator: tcuhawenimana11

Excerpt Created On: 3/22/2023

Excerpt Range: 16280-16518

Ukuntu nakwigirira isuku ku mubiri wange kandi nkaba nakwirinda gutera inda umukobwa kandi bikaba byiza ko umuhungu wese aba asiramuye. Kuri wowe rero amakuru waba ukeneye ni ukuntu wakwirinda gutera umukobwa inda ukoresheje agakingirizo.

Title: G S KAYONZA BOYS Transcri.docx

Doc Creator: tcuhawenimana11

Doc Date: 3/20/2023

Codes Applied: Boys and girls know about the causes of the causes leading to the physiological changes during puberty

Excerpt Creator: tcuhawenimana11

Excerpt Created On: 3/22/2023

Excerpt Range: 16720-16889

uburyo umuhungu ntago bisaba amasaha cyangwa se umunota, ubundi umwana w’umuhungu cyangwa se ingimbi atangira gutera inda afite imyaka cumi n’itanu n’itandatu kuzamura.

Title: G S KAYONZA BOYS Transcri.docx

Doc Creator: tcuhawenimana11

Doc Date: 3/20/2023

Codes Applied: Boys and girls know about the causes of the causes leading to the physiological changes during puberty

Excerpt Creator: tcuhawenimana11

Excerpt Created On: 3/22/2023

Excerpt Range: 16921-16970

Yahuye numukobwa bagakora imibonana mpuzabitsina.

Title: G S KAYONZA BOYS Transcri.docx

Doc Creator: tcuhawenimana11

Doc Date: 3/20/2023

Codes Applied: Obstacles and risks experienced by girls during sexual and reproductive health changes occuring during puberty and adolescence

Excerpt Creator: tcuhawenimana11

Excerpt Created On: 3/22/2023

Excerpt Range: 17040-17262

Yee atayirinze cyangwa umukobwa nawe atazi igihe ari mu gihe cyukwezi kwe nibyo yayitwita kubera ko inanga ngabo irindira igi ry’umukobwa iminsi itanu, hanyuma inanga ngore zo zikayirindira iminsi itatu zikaba zabaye lost.

Title: G S KAYONZA BOYS Transcri.docx

Doc Creator: tcuhawenimana11

Doc Date: 3/20/2023

Codes Applied: Received information is not detailed (mixed)

Excerpt Creator: tcuhawenimana11

Excerpt Created On: 3/22/2023

Excerpt Range: 17386-17980

Umwana w’umuhungu igiha yatera inda urabona nkumukobwa avuye mu mihango, numuhungu barumvikanye hari ukuntu umukobwa aba atazi kubara neza iminsi umuhungu umukobwa akamubwira ngo nge nzi kubara iminsi yange biyizera hari ukuntu uvuga ati turizeranye ati ntarwara urwaye nange ntayo ndwaye tukizerana sibyo? Ugasanga nkumukobwa ajijwe mo nkumunsi umwe noneho wowe uragiye uhise ukorera aho ugasanga havuyemo nko kumutera inda.ese buriya ni nko muyihe minsi buriya umuhungu yatera inda? Mu minsi yukwezi kumukobwa ni iyihe minsi umuhungo yatera inda umukobwa? Iyabakobwa yo ntago napfa kuyimenya.

Title: G S KAYONZA BOYS Transcri.docx

Doc Creator: tcuhawenimana11

Doc Date: 3/20/2023

Codes Applied: Received information is not detailed (mixed)

Excerpt Creator: tcuhawenimana11

Excerpt Created On: 3/22/2023

Excerpt Range: 17993-18121

Nange ntago mbizi. Ubu ntago uzi igihe waterera inda? Ngewe igihe cyose umuhungu yayitera ariko umukobwa biterwa nigihe arimo.

Title: G S KAYONZA BOYS Transcri.docx

Doc Creator: tcuhawenimana11

Doc Date: 3/20/2023

Codes Applied: Received information is misleading and risky

Excerpt Creator: tcuhawenimana11

Excerpt Created On: 3/22/2023

Excerpt Range: 18121-18329

Ni mu bihe rero umukobwa mwakora imibonanompuzabitsina ukamutera inda? Avuye nko mu kwezi kwe ataramaara nkiminsi myinshi. Wansobanuriraho gakeya? Wenda hashize nkiminsi 2 nyine cyangwa umwe abonye imihango.

Title: G S KAYONZA BOYS Transcri.docx

Doc Creator: tcuhawenimana11

Doc Date: 3/20/2023

Codes Applied: Information that boys need

Excerpt Creator: tcuhawenimana11

Excerpt Created On: 3/22/2023

Excerpt Range: 18469-18862

Murakoze, ngewe amakuru numva nkeneye nukumenya uburyo umuntu yakwifata cyangwa se nge nakwifata kubera ko akenshi ntabwo uhorana numubyeyi cyangwa se aho ugiye hose ngo umubyeyi mube muri kumwe ahubwo bwa mbere iyo umenye uburyo wakwifata nibyo byaba byiza kurusha ukundi ikindi umubyeyi nyine akagushishikariza cyangwa se akakuganirirza ku buzima bwimyororokere cyane kuruta kuganira ibindi.

Title: G S KAYONZA BOYS Transcri.docx

Doc Creator: tcuhawenimana11

Doc Date: 3/20/2023

Codes Applied: Information that boys need

Excerpt Creator: tcuhawenimana11

Excerpt Created On: 3/22/2023

Excerpt Range: 18930-20469

Ababyeyi biyi minsi ntabwo baba babyitayeho cyane niyo mpamvu usanga dufite ibibazo twebwe urubyiruko kubera ko umunyarwanda yaravuze ngo umwana apfira mwiterura niyo mpamvu usinga rero twebwe bitugiraho uruhare kubera ko ababyeyi biyi minsi bita ku kazi kuruta kwita ku bana niyo mpamvu rero usanga tujya mu nzira twe twita ko ari nziza ako societe cyangwa bakuru bacu nababyeyi bacu bakazita mbi ariko twebwe tuba tuwumva ko ariyo right yacu kd umusaruro tuzasaruramo ni rwa rupfu nubundi kubera ko iyo ugiye mu nzira mbi ugahura nuwo mukobwa yarahuye nundi mu tipe wavukanye izindi ndwara yavukanye akenshi abana benshi dusigaye tuvukana sida ataruko twahuye nundi wayirwaye. Dusigaye tuvukana sida kubera ko mu babyeyi bacu cyangwa se mu gihe twavutsemo twavutse nta biganiro bigeze bakorana nababyeyi babo niyo mpamvu rero natwe tubigenderamo. Twebwe turi inzirakarengane zababyeyi bacu so niyo mpamvu muriyi minsi kugirango uzabone umubyeyi wakwicaje akakubwira ati mwana wange iyi myaka ugezemo ni myiza arko kuyindi nzira ni mbi. Urugero ugeze mu myaka 15, 16, 17 iyi myaka niyo myaka utererwamo inda cyangwa se ugasanga wiswe papa utarageza igihe cyo kwitwa umubyeyi ugasanga ni hahandi urwariyemo sida umwana uzabyara nawe azayirwara ayivukanye kuko hari igihe umukobwa wateye kubera ko ari kwihisha atajya kwipimisha hakiri kare ngo bakurikira iyo nzira karengane iri mu nda ye niyo mpamvu akenshi muri iyi myaka yacu tuzajya no gushaka ugasanga dushatse ibisasu kubera ko ntabwo twigeze twitabwaho nkuko bikwiye murakoze cyane!

Title: G S KAYONZA BOYS Transcri.docx

Doc Creator: tcuhawenimana11

Doc Date: 3/20/2023

Codes Applied: Information that boys need

Excerpt Creator: tcuhawenimana11

Excerpt Created On: 3/22/2023

Excerpt Range: 20586-20672

Ngewe amakuru navuga ko nkeneye namakuru yubwirinzi kuko abenshi kwifata biratunanira.

Title: G S KAYONZA BOYS Transcri.docx

Doc Creator: tcuhawenimana11

Doc Date: 3/20/2023

Codes Applied: Information that boys need

Excerpt Creator: tcuhawenimana11

Excerpt Created On: 3/22/2023

Excerpt Range: 20780-21391

Wakaguze se uzi uko kambarwa? Yego tuba twarabyize. Kuburyo wagakoresha uri confident ntakibazo? Ntakibazo rwose. None se amakuru y’ubwirinzi ko numva uyazi andi wumva uzabona arenze kurayo n’ayahe? Oya hari ukuntu, sukuvuga ngo umukobwa wese muryamanye aba afite uburwayi cyangwa se umuhungu, hari ukuntu umukobwa aba ari muri cya gihe bita cyuko wa mutera inda ukavuga ati nubundi nimbikora nta bwirinzi nshobora kumutera inda. Muri makeya mbikuvugiye mukeneye kumenya amakuru ku bijyanye nubuzima bwumukobwa kugirango muzajye mujya kugirana ubushuti mubizi neza ibihe barimo nubwo mwagiye mubitsinda. Cyane.

Title: G S KAYONZA BOYS Transcri.docx

Doc Creator: tcuhawenimana11

Doc Date: 3/20/2023

Codes Applied: Information that boys need

Excerpt Creator: tcuhawenimana11

Excerpt Created On: 3/22/2023

Excerpt Range: 21544-21647

Amakuru nakenera nuko nazajya ngirira isuku igitsina cyange cyangwa uko nakwirinda nyine ziriya ndwara.

Title: G S KAYONZA BOYS Transcri.docx

Doc Creator: tcuhawenimana11

Doc Date: 3/20/2023

Codes Applied: Information that boys need

Excerpt Creator: tcuhawenimana11

Excerpt Created On: 3/22/2023

Excerpt Range: 21719-21766

Yego.. ubwo ni bumwe mu buryo bwo kugira isuku.

Title: G S KAYONZA BOYS Transcri.docx

Doc Creator: tcuhawenimana11

Doc Date: 3/20/2023

Codes Applied: Information that boys need

Excerpt Creator: tcuhawenimana11

Excerpt Created On: 3/22/2023

Excerpt Range: 21943-22018

Uko nakwirinda gukora imibonanompuzabitsinda nkatera umukobwa wabandi inda.

Title: G S KAYONZA BOYS Transcri.docx

Doc Creator: tcuhawenimana11

Doc Date: 3/20/2023

Codes Applied: Information that boys need

Excerpt Creator: tcuhawenimana11

Excerpt Created On: 3/22/2023

Excerpt Range: 22285-22467

Kuberako baca umugani ngo utaganiriye na se ntamenya icyo sekuru yasize avuze, mu gihe cyacu dushobora kumva ko byose tubizi ariko hari ibindi tutaramenya. Kuri ngewe ntago mbizi 100

Title: G S KAYONZA BOYS Transcri.docx

Doc Creator: tcuhawenimana11

Doc Date: 3/20/2023

Codes Applied: Obstacles and risks experienced by boys during the sexual and reproductive health changes occuring during puberty and adolescence

Excerpt Creator: tcuhawenimana11

Excerpt Created On: 3/22/2023

Excerpt Range: 23104-23383

Ntabwo ibyo bintu bitworohera kuko nkiyo uzanye nkicyo kiganiro ahita atangira kuvuga ati wabaye indaya. Uvuga ko wabaye indaya aba arinde? Aba ari papa cyangwa mama? Nyine we ahita atangira kuvuga ati watangiye kujya mu mico mibi (bose). Ubivuga cyane ninde? Aba ari umukecuru.

Title: G S KAYONZA BOYS Transcri.docx

Doc Creator: tcuhawenimana11

Doc Date: 3/20/2023

Codes Applied: Obstacles and risks experienced by boys during the sexual and reproductive health changes occuring during puberty and adolescence

Excerpt Creator: tcuhawenimana11

Excerpt Created On: 3/22/2023

Excerpt Range: 23384-23812

Ari papa cyangwa mama ninde bikorohera kuba waganira nawe kubijyanye nubuzima bwimyororokere wumva arinde? Hari ukuntu uba ufite papa wawe ariko ukagiramo akantu ko kumutinya, hari nukuntu uba ufite mama wawe ariko nyine hari ababyeyi baba batarize neza. Kuri wowe nko ku giti cyawe uwo byakorohera niba warigeze kubigerageza ninde? Uwo byakorohera kubiganira yaba ari mama wawe ariko iyo ubizanye ahita avuga ngo warananiranye.

Title: G S KAYONZA BOYS Transcri.docx

Doc Creator: tcuhawenimana11

Doc Date: 3/20/2023

Codes Applied: Obstacles and risks experienced by boys during the sexual and reproductive health changes occuring during puberty and adolescence

Excerpt Creator: tcuhawenimana11

Excerpt Created On: 3/22/2023

Excerpt Range: 23813-24040

Wowe ninde bikorohera kuganira nawe hagati ya mama wawe cyangwa papa wawe niba ubafite bombi? Mama wange. Naho papa? Papa ntabwo tujya tubiganiraho cyane kuko nyine ntago muba munavugana cyane nkuko mama wawe aguhora I ruhande.

Title: G S KAYONZA BOYS Transcri.docx

Doc Creator: tcuhawenimana11

Doc Date: 3/20/2023

Codes Applied: Obstacles and risks experienced by boys during the sexual and reproductive health changes occuring during puberty and adolescence

Excerpt Creator: tcuhawenimana11

Excerpt Created On: 3/22/2023

Excerpt Range: 24072-24440

Kuki papa mutabiganira? Ntago dukunda guhura cyane.

Nange nabiganira na mama kuko akenshi na kenshi papa ntabwo aba ari mu rugo, usanga yagiye ku kazi yaza akaza nka saa yine naryamye agahita aryama yabyuka akongera agahita agenda. Aramutse aza kare se akabona umwanya wamwisanzuraho ukamuganiriza kuriyo ngingo? Oya. Kubera? Mama niwe nisanzuraho cyane kurusha papa.

Title: G S KAYONZA BOYS Transcri.docx

Doc Creator: tcuhawenimana11

Doc Date: 3/20/2023

Codes Applied: Obstacles and risks experienced by boys during the sexual and reproductive health changes occuring during puberty and adolescence

Excerpt Creator: tcuhawenimana11

Excerpt Created On: 3/22/2023

Excerpt Range: 24447-24847

Ibyaribyo byose ndabyimenyera kubera ko akenshi biba biterwa nubuzima umuntu aba yaraciyemo kera, bigaterwa rero nahantu umuntu aba yarakuriye. Ufite ababyeyi? Yego ndabafite ariko nababonye mu gihe ntarimbakeneye reka abe ariko mbivuga kuko mu gihe narimbakeneye sinigeze mbabona. Nicyo mbasabye nkibasaba kuko mba mbona ntakundi nabigenza nta yindi choice mfite kandi ntago nkunda kubiganira cyane.

Title: G S KAYONZA BOYS Transcri.docx

Doc Creator: tcuhawenimana11

Doc Date: 3/20/2023

Codes Applied: Sources of information received during puberty

Excerpt Creator: tcuhawenimana11

Excerpt Created On: 3/22/2023

Excerpt Range: 25014-25083

Nge numva abantu babivuga cyangwa nabanyamakuru cyangwa kuri radiyo.

Title: G S KAYONZA BOYS Transcri.docx

Doc Creator: tcuhawenimana11

Doc Date: 3/20/2023

Codes Applied: Physiological changes occuring during puberty-Boys

Excerpt Creator: tcuhawenimana11

Excerpt Created On: 3/22/2023

Excerpt Range: 25186-25280

Numvaga bavuga nyine ngo iyo wakuze uniga ijwi ukamera nincakwaha nta bindi birenze bavugaga.

Title: G S KAYONZA BOYS Transcri.docx

Doc Creator: tcuhawenimana11

Doc Date: 3/20/2023

Codes Applied: Sources of information received during puberty

Excerpt Creator: tcuhawenimana11

Excerpt Created On: 3/22/2023

Excerpt Range: 25335-25418

Numvaga abantu babivuga muri karitsiye gutyo.

Akenshi na kenshi twabyize mw’ishuri.

Title: G S KAYONZA BOYS Transcri.docx

Doc Creator: tcuhawenimana11

Doc Date: 3/20/2023

Codes Applied: How to take care of themselves when phsyiological changes occur for boys

Excerpt Creator: tcuhawenimana11

Excerpt Created On: 3/22/2023

Excerpt Range: 25621-25703

Ntacyo, icyo bambwira gusa nukujya nigirira isuku noga buri munsi munsi byonyine.

Title: G S KAYONZA BOYS Transcri.docx

Doc Creator: tcuhawenimana11

Doc Date: 3/20/2023

Codes Applied: Sources of information received during puberty

Excerpt Creator: tcuhawenimana11

Excerpt Created On: 3/22/2023

Excerpt Range: 25704-25881

Okay nange ntabwo nigeze mbiganira n’ababyeyi cyane akenshi ubundi twebwe tubikura ku mashuri niho akenshi tubikura cyane kuruta no mu rugo, kuruta no mu giturage kuko akenshi.

Title: G S KAYONZA BOYS Transcri.docx

Doc Creator: tcuhawenimana11

Doc Date: 3/20/2023

Codes Applied: Sources of information received during puberty

Excerpt Creator: tcuhawenimana11

Excerpt Created On: 3/22/2023

Excerpt Range: 26030-26110

Oya akenshi biba byaraturutse ku barimu iyo tugeze kuri topic bita reproduction.

Title: G S KAYONZA BOYS Transcri.docx

Doc Creator: tcuhawenimana11

Doc Date: 3/20/2023

Codes Applied: Received information is misleading and risky

Excerpt Creator: tcuhawenimana11

Excerpt Created On: 3/22/2023

Excerpt Range: 26153-26587

Akenshi ibyo ngibyo tubiganira duhana ama passe ahubwo tuba tuganira ahantu turi buze kubarira cyangwa se tuza guhurira nimba ari abandi banywa inzoga tukavuga ngo noneho turahurira kuri 5 abe ariho dusengerera noneho nibigera muga saa yine dusohokemo tujye kubarya inyuma nyine nibyo tuba tuganira ntabwo tuba tuganira mu burere twagahawe cyangwa se twakabonye ku babyeyi bacu, tuba tubica hejuru nkuca hejuru yamazi kubera ikiraro.

Title: G S KAYONZA BOYS Transcri.docx

Doc Creator: tcuhawenimana11

Doc Date: 3/20/2023

Codes Applied: Received information is misleading and risky

Excerpt Creator: tcuhawenimana11

Excerpt Created On: 3/22/2023

Excerpt Range: 26687-26775

Ikiganiro twembwe nyine dutera ni ikiganiro kigamije uburyo twebwe turi busambane nyine.

Title: G S KAYONZA BOYS Transcri.docx

Doc Creator: tcuhawenimana11

Doc Date: 3/20/2023

Codes Applied: Sources of information received during puberty

Excerpt Creator: tcuhawenimana11

Excerpt Created On: 3/22/2023

Excerpt Range: 26776-27144

Nibyo kuko akenshi iyo ushaka nkayo makuru hari igihe uyakura kuri bakuru bawe, nka mukuru wawe hari igihe wumva abivuga ukavuga ati ahaaa, no mwishuri nyine hari ibyo mwarimu akwigisha ariko iyo amaze akwigisha ishuri ntiribura abantu bakuru nyine mugaterana hari ukuntu mukora nkaka groupe mugashyiramo agakayi hagati ngo ubwo muri kwiga kumbi ikiganiro cyaryoshye.

Title: G S KAYONZA BOYS Transcri.docx

Doc Creator: tcuhawenimana11

Doc Date: 3/20/2023

Codes Applied: Sources of information received during puberty

Excerpt Creator: tcuhawenimana11

Excerpt Created On: 3/22/2023

Excerpt Range: 27578-27786

Hari ukuntu nyine mwishuri mwiga unit ya reproduction mukayigeraho mukabyigaho ako mbere yuko nyine mubyigaho hari ukuntu wiga ukuze ubundi icyo kigero ukakigeramo kandi nubundi uba ufite bakuru bawe mubana.

Title: G S KAYONZA BOYS Transcri.docx

Doc Creator: tcuhawenimana11

Doc Date: 3/20/2023

Codes Applied: Sources of information received during puberty

Excerpt Creator: tcuhawenimana11

Excerpt Created On: 3/22/2023

Excerpt Range: 27862-28065

Nyine urabona hari igihe uba ufite bakuru bawe baba ari benshi nyine baraganiraga nyine hari umuhungu winshuti yabo yari yaje kubasura nkumva bari kubinoza neza bavuga ukuntu agakobwa ari igikara nyine.

Title: G S KAYONZA BOYS Transcri.docx

Doc Creator: tcuhawenimana11

Doc Date: 3/20/2023

Codes Applied: Sources of information received during puberty

Excerpt Creator: tcuhawenimana11

Excerpt Created On: 3/22/2023

Excerpt Range: 28286-28335

Ngewe akenshi nayakuye kwishuri no muri sosiyete.

Title: G S KAYONZA BOYS Transcri.docx

Doc Creator: tcuhawenimana11

Doc Date: 3/20/2023

Codes Applied: Sources of information received during puberty

Excerpt Creator: tcuhawenimana11

Excerpt Created On: 3/22/2023

Excerpt Range: 28468-28622

Nyine kwa kundi uba uri kumwe ninshuti zawe muri kuganira ugasanga nyine ikiganiro bahise bakerekeza ku bintu nkibyo. Ubwo nawe ugaporofitiramo ukabyumva.

Title: G S KAYONZA BOYS Transcri.docx

Doc Creator: tcuhawenimana11

Doc Date: 3/20/2023

Codes Applied: Received information is misleading and risky

Excerpt Creator: tcuhawenimana11

Excerpt Created On: 3/22/2023

Excerpt Range: 28779-28953

Ugasanga bari kuganira nko ku mukobwa ngo uyu mukobwa ni mwiza cyangwa ugasanga bari kumuvugaho ibibi, ugasanga bari kuvuga ngo ni indaya siyitwara neza afite imico iyi niyi.

Title: G S KAYONZA BOYS Transcri.docx

Doc Creator: tcuhawenimana11

Doc Date: 3/20/2023

Codes Applied: Sources of information received during puberty

Excerpt Creator: tcuhawenimana11

Excerpt Created On: 3/22/2023

Excerpt Range: 29142-29451

Kwishuri ntago birabaho ako ukuntu mvuga bibaho ni muri sosiyete rusange, hariya hepfo hari nkigihe usanga bubatse nkama tent ngo buri muntu ajye kureba uko ahagaze ugasanga bari kubivugaho babiganiriza abantu kandi babivuga mu buryo bwa rusange kuburyo uhaciye ukeneye ayo makuru urategereza nyine ukayumva.

Title: G S KAYONZA BOYS Transcri.docx

Doc Creator: tcuhawenimana11

Doc Date: 3/20/2023

Codes Applied: Sources of information received during puberty

Excerpt Creator: tcuhawenimana11

Excerpt Created On: 3/22/2023

Excerpt Range: 29710-29744

Yego ku kigo cy’urubyiruko bibaho.

Title: G S KAYONZA BOYS Transcri.docx

Doc Creator: tcuhawenimana11

Doc Date: 3/20/2023

Codes Applied: Received information is right and provides sufficient information helping boys and girls during puberty

Excerpt Creator: tcuhawenimana11

Excerpt Created On: 3/22/2023

Excerpt Range: 30062-30199

Yego bijya bibaho cyane kunzu yurubyiruko iyo ushaka amakuru yimyororokere ujya hariya kunzu yurubyiruko bakakugira inama yuko wakitwara.

Title: G S KAYONZA BOYS Transcri.docx

Doc Creator: tcuhawenimana11

Doc Date: 3/20/2023

Codes Applied: Obstacles and risks experienced by boys during the sexual and reproductive health changes occuring during puberty and adolescence

Excerpt Creator: tcuhawenimana11

Excerpt Created On: 3/22/2023

Excerpt Range: 30836-31074

Iyo udasobanukiwe neza amakuru yo kubuzima bwimyororokere ushobora kugenda ukikundishwa nko ku mukobwa kubera ko utazi ukuntu wakirinda indwara cyangwa kumutera inda ugasanga mukoze imibonanopuzabitsina umuteye inda kubera amakuru makeya.

Title: G S KAYONZA BOYS Transcri.docx

Doc Creator: tcuhawenimana11

Doc Date: 3/20/2023

Codes Applied: Obstacles and risks experienced by boys during the sexual and reproductive health changes occuring during puberty and adolescence

Excerpt Creator: tcuhawenimana11

Excerpt Created On: 3/22/2023

Excerpt Range: 31161-31215

Utangira kwitwa papa kandi warumwana ufite imyaka 16.

Title: G S KAYONZA BOYS Transcri.docx

Doc Creator: tcuhawenimana11

Doc Date: 3/20/2023

Codes Applied: Obstacles and risks experienced by boys during the sexual and reproductive health changes occuring during puberty and adolescence

Excerpt Creator: tcuhawenimana11

Excerpt Created On: 3/22/2023

Excerpt Range: 31391-32068

Ingorane tugira twembwe nkabana babahungu cyangwa se urubyiruko nuko tutaba dufite amakuru ahagije ku buizima bwimyororokere, urabajije ngo ni ikihe kibazo wagira mu gihe waba wateye inda kandi uba wakuze? Kubera ko uba ugikeneye kurerwa ugatera iyo nda icyo gihe ntago wakitwa papa. witwa papa kubera ko ibyo uba wakoze utaba wabimenye ariko nawe uba ugikenewe kurerwa niyo mpamvu ndumva yuko ingorane duhura nazo akenshi aruko tutaba dufite amakuru ahagije kubuzima bwimyororokere ariyo mpamvu abakobwa ndetse nabahungu twabyaye mu gihe tutabiteganyije aribyo bya bindi twumva ngo twabyaye kuri mpa numveho. Akenshi muriki gihe abana benshi dusigaye tuvuka kuri mpa numveho.

Title: G S KAYONZA BOYS Transcri.docx

Doc Creator: tcuhawenimana11

Doc Date: 3/20/2023

Codes Applied: Obstacles and risks experienced by boys during the sexual and reproductive health changes occuring during puberty and adolescence

Excerpt Creator: tcuhawenimana11

Excerpt Created On: 3/22/2023

Excerpt Range: 32163-32285

Ntago Babura kubera ko kayonza bazi ko ari igicumbi cyindaya bakaba bazi ko ari igicumbi cyinda zitateguwe ntago byabura.

Title: G S KAYONZA BOYS Transcri.docx

Doc Creator: tcuhawenimana11

Doc Date: 3/20/2023

Codes Applied: Obstacles and risks experienced by boys during the sexual and reproductive health changes occuring during puberty and adolescence

Excerpt Creator: tcuhawenimana11

Excerpt Created On: 3/22/2023

Excerpt Range: 32397-32475

Iyo utabifiteho amakuru ushobora kwigirira isuku nkeya bikakuviramo uburwayi.

Title: G S KAYONZA BOYS Transcri.docx

Doc Creator: tcuhawenimana11

Doc Date: 3/20/2023

Codes Applied: Obstacles and risks experienced by boys during the sexual and reproductive health changes occuring during puberty and adolescence

Excerpt Creator: tcuhawenimana11

Excerpt Created On: 3/22/2023

Excerpt Range: 32667-32751

Ingorane wahura nayo nuko ushobora gutera inda ukiri muto cyangwa ugakuramo indwara.

Title: G S KAYONZA BOYS Transcri.docx

Doc Creator: tcuhawenimana11

Doc Date: 3/20/2023

Codes Applied: Obstacles and risks experienced by boys during the sexual and reproductive health changes occuring during puberty and adolescence

Excerpt Creator: tcuhawenimana11

Excerpt Created On: 3/22/2023

Excerpt Range: 32826-32851

Sida, imitezi, mburugu.

Title: G S KAYONZA BOYS Transcri.docx

Doc Creator: tcuhawenimana11

Doc Date: 3/20/2023

Codes Applied: Obstacles and risks experienced by boys during the sexual and reproductive health changes occuring during puberty and adolescence

Excerpt Creator: tcuhawenimana11

Excerpt Created On: 3/22/2023

Excerpt Range: 33079-33376

Ingorane yahura nazo nuko agira ubushake bwo gutera akabariro yabikora akabikora ntamakuru abifiteho ugasanga wenda ateye nkinda, ugasanga akuyemo sida nuwo mwana uvutse icyo gihe nawe azavukana ubwo burwayo kuko mubikoze mukiri bato mutinya kubibwira iwanyu nubundi umwana kwitabwaho biragorana.

Title: G S KAYONZA BOYS Transcri.docx

Doc Creator: tcuhawenimana11

Doc Date: 3/20/2023

Codes Applied: Obstacles and risks experienced by boys during the sexual and reproductive health changes occuring during puberty and adolescence

Excerpt Creator: tcuhawenimana11

Excerpt Created On: 3/22/2023

Excerpt Range: 33435-33626

Ingorane uhura nazo nugupfa mu mutwe. Gute rero nicyo nshaka kumenya? Kuko nyine hari ukuntu nyine ubishaka ukabibura ariko wowe ugahora igitekerezo ari uwabimpa. Ntagitekerezo kizima wagira.

Title: G S KAYONZA BOYS Transcri.docx

Doc Creator: tcuhawenimana11

Doc Date: 3/20/2023

Codes Applied: Obstacles and risks experienced by boys during the sexual and reproductive health changes occuring during puberty and adolescence

Excerpt Creator: tcuhawenimana11

Excerpt Created On: 3/22/2023

Excerpt Range: 33723-34022

Ingaruka uhura nazo mu gihe wapfuye mu bitekerezo nta kindi ushobora gutekereza cyangwa gukora udakoze iyo mibonanompuzabitsina ntakindi pe. Hari nigihe ingaruka zibivamo ujya no gufata ibiyobyabwenge kugirango ube ubyibagiweho gato niyo mpamvu rero ubuzima bwimyororokere tubikeneye kurusha ibindi.

Title: G S KAYONZA BOYS Transcri.docx

Doc Creator: tcuhawenimana11

Doc Date: 3/20/2023

Codes Applied: Obstacles and risks experienced by girls during sexual and reproductive health changes occuring during puberty and adolescence

Excerpt Creator: tcuhawenimana11

Excerpt Created On: 3/22/2023

Excerpt Range: 34203-34409

Umukobwa aratwita ugasanga avuye mwishuri akanagira nizindi ndwara arwara. Ni ibyo byonyine 3 uvuze?

Ingaruka nanone umukobwa yagira kuba abitekereza ariko atabikora nanone kwikinisha yagira ingaruka nawe.

Title: G S KAYONZA BOYS Transcri.docx

Doc Creator: tcuhawenimana11

Doc Date: 3/20/2023

Codes Applied: Obstacles and risks experienced by girls during sexual and reproductive health changes occuring during puberty and adolescence

Excerpt Creator: tcuhawenimana11

Excerpt Created On: 3/22/2023

Excerpt Range: 34492-34735

Ashobora kwishora mu mibonanompuzabitsina. Yishoye mu mibonanompuzabitsina akabikora rimwe hari ikibazo? Ashobora gukuramo indwara. Yabikoze rimwe gusa? Yego. Indwara yakuramo yabikoze rimwe gusa ninkizihe se ubwo? Ni nka sida cyangwa imitezi.

Title: G S KAYONZA BOYS Transcri.docx

Doc Creator: tcuhawenimana11

Doc Date: 3/20/2023

Codes Applied: Obstacles and risks experienced by girls during sexual and reproductive health changes occuring during puberty and adolescence

Excerpt Creator: tcuhawenimana11

Excerpt Created On: 3/22/2023

Excerpt Range: 34736-35138

Ese umuntu ashobora kurwara sida cyangwa indwara zandurira mumibonanompuzabitsina igihe cyose akoze iyo mibonanompuzabitsina? Oya. Bigenda bite? Wenda nko gutiza ibikoresho bikomeretsa wenda nabyo bishobora kukwanduza. Igihe cyose umukobwa ashobora kwandura sida cyangwa izo ndwara? Igihe yakoze imibonanompuzabitsina ayikoranye nuwanduye cyangwa idakingiye. Idakingiye? Yego. Ni icyo nashakaga kumva.

Title: G S KAYONZA BOYS Transcri.docx

Doc Creator: tcuhawenimana11

Doc Date: 3/20/2023

Codes Applied: Obstacles and risks experienced by girls during sexual and reproductive health changes occuring during puberty and adolescence

Excerpt Creator: tcuhawenimana11

Excerpt Created On: 3/22/2023

Excerpt Range: 35268-35366

Yandura indwara akareka nishuri ugasanga umukobwa niyo yasamye agize igisebo mu bandi bantu bose.

Title: G S KAYONZA BOYS Transcri.docx

Doc Creator: tcuhawenimana11

Doc Date: 3/20/2023

Codes Applied: Obstacles and risks experienced by girls during sexual and reproductive health changes occuring during puberty and adolescence

Excerpt Creator: tcuhawenimana11

Excerpt Created On: 3/22/2023

Excerpt Range: 35367-35604

Ese abakobwa bose iyo basamye barabigaragaza? Oya. Hari ababihisha, iyo babihishe se hari icyo baba? Hari igihe bibaviramo kuyikuramo. Iyo ayikuyemo se hari ingaruka zindi byagira? Baba bavuga ngo ashobora kuyikuramo ntazongere kubyara.

Title: G S KAYONZA BOYS Transcri.docx

Doc Creator: tcuhawenimana11

Doc Date: 3/20/2023

Codes Applied: Obstacles and risks experienced by girls during sexual and reproductive health changes occuring during puberty and adolescence

Excerpt Creator: tcuhawenimana11

Excerpt Created On: 3/22/2023

Excerpt Range: 35628-35669

Hari nigihe yayikuramo ugasanga arapfuye.

Title: G S KAYONZA BOYS Transcri.docx

Doc Creator: tcuhawenimana11

Doc Date: 3/20/2023

Codes Applied: Obstacles and risks experienced by girls during sexual and reproductive health changes occuring during puberty and adolescence

Excerpt Creator: tcuhawenimana11

Excerpt Created On: 3/22/2023

Excerpt Range: 35742-35791

Ntago nyine abona uko akurikirana umwana we neza.

Title: G S KAYONZA BOYS Transcri.docx

Doc Creator: tcuhawenimana11

Doc Date: 3/20/2023

Codes Applied: Obstacles and risks experienced by girls during sexual and reproductive health changes occuring during puberty and adolescence

Excerpt Creator: tcuhawenimana11

Excerpt Created On: 3/22/2023

Excerpt Range: 35792-35983

Agiye kubyara kwa muganga ataragiye ajya kwipimisha mu gihe yaratwite ni izihe ngaruka ashobora guhura nazo?

Nyine ntago amenya igihe cy’umwana we. Hari nigiye yabyara nabi ugasanga arapfuye.

Title: G S KAYONZA BOYS Transcri.docx

Doc Creator: tcuhawenimana11

Doc Date: 3/20/2023

Codes Applied: Obstacles and risks experienced by girls during sexual and reproductive health changes occuring during puberty and adolescence

Excerpt Creator: tcuhawenimana11

Excerpt Created On: 3/22/2023

Excerpt Range: 36171-36232

Nukutegera umuruta wenda ngo baganire cyangwa se ababyeyi be.

Title: G S KAYONZA BOYS Transcri.docx

Doc Creator: tcuhawenimana11

Doc Date: 3/20/2023

Codes Applied: Obstacles and risks experienced by girls during sexual and reproductive health changes occuring during puberty and adolescence

Excerpt Creator: tcuhawenimana11

Excerpt Created On: 3/22/2023

Excerpt Range: 36247-36356

Iyo atabyize ngo anabikurikiranye amenye uko azitwara nazo ziba ari inzitizi. Ubwo biba arukutagira amakuru.

Title: G S KAYONZA BOYS Transcri.docx

Doc Creator: tcuhawenimana11

Doc Date: 3/20/2023

Codes Applied: Obstacles and risks experienced by boys during the sexual and reproductive health changes occuring during puberty and adolescence

Excerpt Creator: tcuhawenimana11

Excerpt Created On: 3/22/2023

Excerpt Range: 36433-36550

Nabyo bishobora kuba inzitizi arko iyo ushaka kumenya ikintu uranabaza ntago ari ngombwa ngo ababyeyi bakuganiririze.

Title: G S RWAMAGANA A BOYS.docx

Doc Creator: tcuhawenimana11

Doc Date: 3/20/2023

Codes Applied: Physiological changes occuring during puberty-Boys

Excerpt Creator: tcuhawenimana11

Excerpt Created On: 3/22/2023

Excerpt Range: 198-462

Impinduka ziba ku mwana ugeze mu gihe cyubugimbi atangira guhinduka ku mubiri akaniga ijwi umubiri nyine ugatangira guhinduka. Ugahinduka gute? Uko umubiri waruri ntago ariko uba umeze atangira kumera insya, incakwaha, akaniga ijwi aba atandukanye numwana mutoya.

Title: G S RWAMAGANA A BOYS.docx

Doc Creator: tcuhawenimana11

Doc Date: 3/20/2023

Codes Applied: Physiological changes occuring during puberty-Boys

Excerpt Creator: tcuhawenimana11

Excerpt Created On: 3/22/2023

Excerpt Range: 551-1003

Zimwe mu mpinduka zigaragara ku mwana wumuhungu uri mu gihe cyingimbi aniga ijwi, rimwe na rimwe usanga nimyumvire ye igenda ihinduka nkumubyeyi we yamubwira ikintu akumva ntago babyumva kimwe akumva niwe uri mu kuri, ikindi nacyo ashobora kuzana ubwanwa bitewe nubuzima abamo, ashobora no kwaguka nko mu maso ashobora no kurwara ibiheri bityo rero bigatuma nisura ye ihinduka uko yagaragaraga ko ari umwana ugatangira kubona ko yabaye umuntu mukuru.

Title: G S RWAMAGANA A BOYS.docx

Doc Creator: tcuhawenimana11

Doc Date: 3/20/2023

Codes Applied: Received information is misleading and risky

Excerpt Creator: tcuhawenimana11

Excerpt Created On: 3/22/2023

Excerpt Range: 1031-1349

Izo mpinduka zishobora guterwa nimibereho ubayemo, ushobora nko kuba urya neza bugatuma uzana nkubwanwa, ukaniga ijwi cyangwa se ufite inshuti zitari nziza ushobora kugera muri iyo myaka zikakujyana nko kunywa ibiyobyabwenge zikubwira ko ari byiza bitewe na sosiyete urimo bigatuma uhinduka gutyo ukagira nimico mibi.

Title: G S RWAMAGANA A BOYS.docx

Doc Creator: tcuhawenimana11

Doc Date: 3/20/2023

Codes Applied: Boys and girls know about the causes of the causes leading to the physiological changes during puberty

Excerpt Creator: tcuhawenimana11

Excerpt Created On: 3/22/2023

Excerpt Range: 1031-1349

Izo mpinduka zishobora guterwa nimibereho ubayemo, ushobora nko kuba urya neza bugatuma uzana nkubwanwa, ukaniga ijwi cyangwa se ufite inshuti zitari nziza ushobora kugera muri iyo myaka zikakujyana nko kunywa ibiyobyabwenge zikubwira ko ari byiza bitewe na sosiyete urimo bigatuma uhinduka gutyo ukagira nimico mibi.

Title: G S RWAMAGANA A BOYS.docx

Doc Creator: tcuhawenimana11

Doc Date: 3/20/2023

Codes Applied: Psychological changes occuring during puberty-Boys

Excerpt Creator: tcuhawenimana11

Excerpt Created On: 3/22/2023

Excerpt Range: 1401-1966

Ngewe impinduka numva hari ukuntu batangira kugira irari ryubusambanyi iyo batangiye kugera mu gihe cyubugimbi kd ikindi usanga umwana atangira kwigira nkumuntu ufite imbaraga kwisi ko ntamuntu ushobora kuba yamukubita, nimero ni iya kabiri imyaka 16 ishuri niga muwa 2 secondary, ibyo nibyo bimenyetso nshobora kuba nzi. Nukuvuga ngo iyo ageze muricyo kigero akaba yasambana atitaye ku ngaruka zamubaho apfa kuba yumva gusa umubiri ubishaka kurugero ruri hejuru. Byaba byarakubayeho? Yego byambayeho arko nyine byahise birekera. Ariko icyo gikorwa cyabaye? Yeeee.

Title: G S RWAMAGANA A BOYS.docx

Doc Creator: tcuhawenimana11

Doc Date: 3/20/2023

Codes Applied: Physiological changes occuring during puberty-Girls

Excerpt Creator: tcuhawenimana11

Excerpt Created On: 3/22/2023

Excerpt Range: 2157-2301

Impinduka umukobwa agira mu gihe ageze mu gihe cyubwangavu harimo kuza kwimihango, gutangira kumera incakwaha, amabere ye agatangira kwiyongera.

Title: G S RWAMAGANA A BOYS.docx

Doc Creator: tcuhawenimana11

Doc Date: 3/20/2023

Codes Applied: Boys and girls know about the causes of the causes leading to the physiological changes during puberty

Excerpt Creator: tcuhawenimana11

Excerpt Created On: 3/22/2023

Excerpt Range: 2348-2469

iterwa nimisemburo yumubiri nindyo arya niba arya indyo yuzuye impinduka ziza hakiri kare mbere yuko urya nabi byamubaho.

Title: G S RWAMAGANA A BOYS.docx

Doc Creator: tcuhawenimana11

Doc Date: 3/20/2023

Codes Applied: Psychological changes occuring during puberty-Girls

Excerpt Creator: tcuhawenimana11

Excerpt Created On: 3/22/2023

Excerpt Range: 2586-2868

Impinduka ziba ku mwana wumukobwa mu gihe ageze mu gihe cyubwangavu usanga atangiye kugira isuku, ugasanga atangiye gukora neza imirimo cyangwa no kumvira ababyeyi akurikije nabandi agendana nabo agatangira kumva ko yakuze yagira inshuti nyinshi zabahungu ndetse akaba yanakwigenga,

Title: G S RWAMAGANA A BOYS.docx

Doc Creator: tcuhawenimana11

Doc Date: 3/20/2023

Codes Applied: Boys and girls know about the causes of the causes leading to the physiological changes during puberty

Excerpt Creator: tcuhawenimana11

Excerpt Created On: 3/22/2023

Excerpt Range: 2908-3185

Ikibitera ni imyaka aba ageze cyangwa se abo agendana nabo bashobora kuba agendana nabantu bakuru nawe bigatuma yigira mukuru nkabo, ndetse ikindi cyabitera ni imisemburo yo mumubriri nkibyo arya nimba ari byiza bigatuma yaba mukuru akiri mutoya akajya mu cyiciro cyubwangavu,

Title: G S RWAMAGANA A BOYS.docx

Doc Creator: tcuhawenimana11

Doc Date: 3/20/2023

Codes Applied: How boys experience physiological changes that occured to them during puberty

Excerpt Creator: tcuhawenimana11

Excerpt Created On: 3/22/2023

Excerpt Range: 3398-3942

Izo mpinduka mu zaba zarambayeho icyambere nanize ijwi icya kabiri nukumera incakwaha nicyagatatu nuko nigitsina cyange cyariyongereye nikindi cya kane ninsya zaraje hari nigihe umubiri ugushuka ukumva wakora imibonanompuzabitsina bitewe ningaruka zabyo wagiye wigaho nawe ukarwanya uwo mubiri ukuntu uri kugushuka, murakoze. Izo mpinduka nzibonye ntabwo nagiye mbibwira buri muntu wese, barambazaga ati ese ugira insya nkababwira ato oya ngewe ntansya ngira nge ndacyari umwana bigatuma nyine mpisha ikintu mfite numubyeyi nkumva ntabimubwira.

Title: G S RWAMAGANA A BOYS.docx

Doc Creator: tcuhawenimana11

Doc Date: 3/20/2023

Codes Applied: Sources of information received during puberty

Excerpt Creator: tcuhawenimana11

Excerpt Created On: 3/22/2023

Excerpt Range: 4152-4260

Yego. Mu babyeyi bawe se uwo wumva wakwisanzuraho yaba papa cyangwa mama? Kubera ko papa ntawe ubwo ni mama.

Title: G S RWAMAGANA A BOYS.docx

Doc Creator: tcuhawenimana11

Doc Date: 3/20/2023

Codes Applied: How boys experience physiological changes that occured to them during puberty

Excerpt Creator: tcuhawenimana11

Excerpt Created On: 3/22/2023

Excerpt Range: 4403-4543

Impinduka zaba zarambayeho ni nko kwiyongera mu bwenge nyine ugakora ibintu nkibyabantu bakuru cyangwa igitsina kikiyongera ndumva ntabindi.

Title: G S RWAMAGANA A BOYS.docx

Doc Creator: tcuhawenimana11

Doc Date: 3/20/2023

Codes Applied: How boys experience physiological changes that occured to them during puberty

Excerpt Creator: tcuhawenimana11

Excerpt Created On: 3/22/2023

Excerpt Range: 4623-4833

Wabyitwayemo ute; ntabwo nagizengo nuburwayi nyine kuko narimfite amakuru ako nanone ntakubibwira umuntu uwariwe wese wenda nkumubyeyi yakuganiriza akwigisha ku bintu bijyanye nimyororokere mukaganira murakoze.

Title: G S RWAMAGANA A BOYS.docx

Doc Creator: tcuhawenimana11

Doc Date: 3/20/2023

Codes Applied: How boys experience physiological changes that occured to them during puberty

Excerpt Creator: tcuhawenimana11

Excerpt Created On: 3/22/2023

Excerpt Range: 4907-5450

Impinduka zambayeho nukumera insya no kumera incakwaha ndumva arizo zonyine. Ari ubucakwaha ninsya se niki cyabanje kuza? Ninsya. Warazishimiye se, wumvishe ari ibintu bigutunguye? Ngewe rero narabyangaga kubera ko najyaga numva ngo ni indwara ibizanaho maze ubundi bikantera ubwoba kuko niko abana twari duturanye bambwiraga. Ugize izo mpungenge se hari uwo wigeze uzisangiza? Wapi kuko nubungubu usibye hano mbivugiye ntawundi muntu ubizi. Nonese uracyafite izo mpungenge ko byaba biterwa nuburwayi? Oya mwishuri narabyize ko atari uburwayi.

Title: G S RWAMAGANA A BOYS.docx

Doc Creator: tcuhawenimana11

Doc Date: 3/20/2023

Codes Applied: How boys experience physiological changes that occured to them during puberty

Excerpt Creator: tcuhawenimana11

Excerpt Created On: 3/22/2023

Excerpt Range: 5560-6427

Impinduka zaba zarambayeho nuko natangiye kumva nshaka amafaranga nkumva nangiye gukenera nkibyo mbona mugenzi wange mukuru afite nkumva nange nshaka inkweto zihenze nkiizo mbona abandi bafite, natangiye kujya nkunda abakobwa ndetse nkatangira kujya ngenda mbasaba urukundo, murakoze. Uko nabyitwayemo nasanze yuko nubwo naba nkeneye amafaranga bitavuze kujya mubujura cyangwa se nge muzindi ngeso mbi zatuma mbona amafaranga ahubwo nange nayakorera ndetse nkakora ibyo nshoboye bitamvunnye nkabasha kugera kucyo nifuza cyangwa nkabisangiza ababyeyi bange bakajya bamfasha muribyo byifuzo byange murakoze. Uko nabyitwayemo kuba numva nshaka abakobwa bivuze ko numvaga nshaka ko twakora imibonanompuzabitsina ahubwo numvaga ko nagira inshuti zabakobwa nange abandi tugenda bambwira ngi mfite chr nange nkababwira ngo ndamufite atarukuvuga ngo nuko twajya mu ngeso mbi

Title: G S RWAMAGANA A BOYS.docx

Doc Creator: tcuhawenimana11

Doc Date: 3/20/2023

Codes Applied: How boys experience physiological changes that occured to them during puberty

Excerpt Creator: tcuhawenimana11

Excerpt Created On: 3/22/2023

Excerpt Range: 6550-6823

Impinduka zaba zarambayeho nuko natangiye kumera insya, nkazana nincakwaha, ijwi naryo ritangiye kujya riza gake gake. Ntakindi kimenyetso? Amabya yange yariyongereye. Wabyitwayemo ute? Nabibwiye ababyeyi barabwira ngo ibyo ni ibikubaho nyine iyo utangiye kujya mubugimbi.

Title: G S RWAMAGANA A BOYS.docx

Doc Creator: tcuhawenimana11

Doc Date: 3/20/2023

Codes Applied: How boys experience physiological changes that occured to them during puberty

Excerpt Creator: tcuhawenimana11

Excerpt Created On: 3/22/2023

Excerpt Range: 6937-7052

Ngewe natangiye kuniga ijwi mera ninsya, nkahora numva nshaka abakobwa imbere yange nkabona nigitsina kiri gukura.

Title: G S RWAMAGANA A BOYS.docx

Doc Creator: tcuhawenimana11

Doc Date: 3/20/2023

Codes Applied: How boys experience physiological changes that occured to them during puberty

Excerpt Creator: tcuhawenimana11

Excerpt Created On: 3/22/2023

Excerpt Range: 7101-7365

wiroteraho byaje nka 3 kumera nincakwaha. Wabyitwayeho utese wiroteyeho cyangwa ibindi bimenyetso? Nabibwiye mukuru wange arambwiye ngo ugomba kubibwira mama akakugira inama noneho ndabimubwira arambwira ngo watangiye gukura ntukagendere mu kigare cyabantu bakuru.

Title: G S RWAMAGANA A BOYS.docx

Doc Creator: tcuhawenimana11

Doc Date: 3/20/2023

Codes Applied: Information that girls need

Excerpt Creator: tcuhawenimana11

Excerpt Created On: 3/22/2023

Excerpt Range: 7696-8437

Mba numva umwana wumukobwa ugeze muriyo myaka amakuru yakenera nkababyeyi cyangwa kwishuri rye bashobora kumuganiriza bakamubwira buryo ki yabyitwaramo aramutse agiye mu mihango sibyo kuko hari nkumukobwa ari nkubwa mbere aba agiye mu mihango ugasanga nyine yabyitwayemo mu buryo butari bwiza cyangwa se akaba agiye mu mihango inshuti ze akabizibwira zigatangira kumubeshya bitewe nuko zimuruta zikamubwira ati niba ujya mi mihango ukababara bisaba yuko wakora imibonanompuzabitsina, ariko iyo wize usanga akenshi baba bakubeshya niyo mpamvu abarimu cyangwa ababyeyi baba bagomba kwegera abo bangavu bakabaha amakuru bakababwira bati bigenda gutya na gutya kandi ugomba kwirinda inshuti mbi kandi ugomba kongera isuku ndumva ari ibyo ngibyo.

Title: G S RWAMAGANA A BOYS.docx

Doc Creator: tcuhawenimana11

Doc Date: 3/20/2023

Codes Applied: Information that girls need

Excerpt Creator: tcuhawenimana11

Excerpt Created On: 3/22/2023

Excerpt Range: 8541-8665

Icyo gihe umukobwa wumwangavu amakuru yakenera nuko yajya kukigonderabuzima bakamugira inama yizo mpinduka zagiye zimubaho.

Title: G S RWAMAGANA A BOYS.docx

Doc Creator: tcuhawenimana11

Doc Date: 3/20/2023

Codes Applied: Information that girls need

Excerpt Creator: tcuhawenimana11

Excerpt Created On: 3/22/2023

Excerpt Range: 8750-9368

Nuko yakenera amakuru yukuntu umubiri we ugenda uhindagurika akagenda abaza nabantu bamuruta, hari nigihe atangira kuzana ibiheri hanyuma bagenzi be bakamugira inama yukuntu yaryamana numuhungu kugirango ibyo biheri bigende ariko akenshi na kenshi usanga izo nshuti zikugiraiyo nama atari inshuti nyanshuti. Icyiza nuko wajya kukigo nderabuzima wagize nkibyo bimenyetso akumva yakora iyo mibonanompuzabitsina yamushuka ako kanya akumva yabikora ariko akenshi na kenshi usanga ataribyo kuko ashobora no gutwara inda itateguwe. Mbese akeneye kumenya ayo makuru kuburyo niyo izo ngaruka zamubaho kugirango atware iyo nda.

Title: G S RWAMAGANA A BOYS.docx

Doc Creator: tcuhawenimana11

Doc Date: 3/20/2023

Codes Applied: Received information is not detailed (mixed)

Excerpt Creator: tcuhawenimana11

Excerpt Created On: 3/22/2023

Excerpt Range: 9587-9830

Mu gihe ari mu mihango, mu gihe yayivuyemo cyangwa mu gihe atarayijyamo wumva ari ryari yatwara inda? Hari nigihe inda uyifata ukiri mu mihango, imihano yawe rero igeraho igahagarara ntago ihita iza icyo uhita ukora wegera abaganga ukababaza.

Title: G S RWAMAGANA A BOYS.docx

Doc Creator: tcuhawenimana11

Doc Date: 3/20/2023

Codes Applied: Information that boys need

Excerpt Creator: tcuhawenimana11

Excerpt Created On: 3/22/2023

Excerpt Range: 10248-10841

makuru umwana wumuhungu yagira mu gihe ari mu gihe cyingimbi hari ukugani numubyeyi we cyangwa se bakuru be bakamubwira ibijyanye nubuzima bwimyororokere yababwira ko wenda yiroteyeho amakuru yahabwa bashobora kumubwira ngo ujye wirnda cyane inshuti zagushyira mu byago kuko akenshi na kenshi nizo zitera ubusambanyi bwa hato na hato bigatuma byakwicira ubuzima ubwo ngubwo bakabikubuza cyangwa bakakugira inama zo kujya ugendana nabantu mungana cyangwa se wakumva abafite ikigare ukabavamo hakiri kare bataragutera ibyo bigare bafite cyangwa izo ngeso mbi bafite zikagutera ikibazo murakoze.

Title: G S RWAMAGANA A BOYS.docx

Doc Creator: tcuhawenimana11

Doc Date: 3/20/2023

Codes Applied: Types of information provided to boys and girls during puberty

Excerpt Creator: tcuhawenimana11

Excerpt Created On: 3/22/2023

Excerpt Range: 11371-11735

Amakuru naba narahawe nuko iyo umuntu ageze mu bugimbi icyambere numvise nuko ngo igitsina cye cyiyongera kandi akagira ibintu byirari rirenze kubuzima bwe arahinduka amajwi agahinduka ndetse nawe ubwawe ukumva ko waremereye uri hejuru cyane, hari nibindi bintu bigenda biza kumubiri harimo insya, harimo kumera incakwaha numubri we mbega ukabona yuko wahindutse.

Title: G S RWAMAGANA A BOYS.docx

Doc Creator: tcuhawenimana11

Doc Date: 3/20/2023

Codes Applied: Types of information provided to boys and girls during puberty

Excerpt Creator: tcuhawenimana11

Excerpt Created On: 3/22/2023

Excerpt Range: 11905-12002

Nge amakuru bampaye barambwiye ngo uzana ibiheri mu maso ugatangira kuzana irari n’ibyo bambwiye.

Title: G S RWAMAGANA A BOYS.docx

Doc Creator: tcuhawenimana11

Doc Date: 3/20/2023

Codes Applied: Received information is right and provides sufficient information helping boys and girls during puberty

Excerpt Creator: tcuhawenimana11

Excerpt Created On: 3/22/2023

Excerpt Range: 11905-12002

Nge amakuru bampaye barambwiye ngo uzana ibiheri mu maso ugatangira kuzana irari n’ibyo bambwiye.

Title: G S RWAMAGANA A BOYS.docx

Doc Creator: tcuhawenimana11

Doc Date: 3/20/2023

Codes Applied: Obstacles and risks experienced by boys during the sexual and reproductive health changes occuring during puberty and adolescence

Excerpt Creator: tcuhawenimana11

Excerpt Created On: 3/22/2023

Excerpt Range: 12209-12470

Nyine icyo kibazo iyo kije hari igihe uba uri umuntu wize waciye mwishuri ariko ugasanga hari nabantu babyishoramo kubera ko batigeze biga ariko iyo wize riragufata nyine rikakuganza ariko ukavuga ati mu mutwe wange mubyukuri hakagira akantu kazamo karikurinda.

Title: G S RWAMAGANA A BOYS.docx

Doc Creator: tcuhawenimana11

Doc Date: 3/20/2023

Codes Applied: How boys experience physiological changes that occured to them during puberty

Excerpt Creator: tcuhawenimana11

Excerpt Created On: 3/22/2023

Excerpt Range: 12909-13221

Nyine iyo kwifata byanze ubundi iyo mumubiri wawe bihindutse urabireka nibintu bisanzwe bihita birekera ni iminota mikeya bigashira ugomba kwi controller nyine umubiri wawe ukawuturisha ntago ari ngombwa ko uhita ujya kwishora mu mibonanompuzabitsina igihe umubiri wawe wahindutse ugomba kwitwara neza murakoze.

Title: G S RWAMAGANA A BOYS.docx

Doc Creator: tcuhawenimana11

Doc Date: 3/20/2023

Codes Applied: How boys experience physiological changes that occured to them during puberty

Excerpt Creator: tcuhawenimana11

Excerpt Created On: 3/22/2023

Excerpt Range: 13455-13621

Amakuru naba narahawe ku bijyanye nubuzima bwimyororokere nuko bambwiye ko iyo ugeze mu gihe cyubugimbi ugomba kumera insya, incakwaha ndetse nigitsina cyikiyongera.

Title: G S RWAMAGANA A BOYS.docx

Doc Creator: tcuhawenimana11

Doc Date: 3/20/2023

Codes Applied: Sources of information received during puberty

Excerpt Creator: tcuhawenimana11

Excerpt Created On: 3/22/2023

Excerpt Range: 13639-13665

Ni mukuru wange wabimbwiye

Title: G S RWAMAGANA A BOYS.docx

Doc Creator: tcuhawenimana11

Doc Date: 3/20/2023

Codes Applied: How to take care of themselves when phsyiological changes occur for boys How to take care of themselves when physiological changes occur-girls

Excerpt Creator: tcuhawenimana11

Excerpt Created On: 3/22/2023

Excerpt Range: 13807-14773

Amakuru ngewe naba narahawe nuko umuntu ugeze nuriyo myaka yubugimbi cyangwa se ubwangavu nko ku muhungu ashobora kuzana ubwanwa, ashobora kuzana insya ashobora gutangira kumva ko arenze akiyemera akumva afite imbaraga cyangwa nanone andi makuru ajyanye nibyo ngibyo bashobora kukubwira ati nugeza imyaka nka 18 cyangwa 19 utariroteraho na rimwe cyangwa ukaba utarashaka gutereta umukunzi icyo ngo ushobora kuba urwaye cyangwa se ukagira iyo mwaka 18 cyangwa 19 utaraniga ijwi, nyine utaragira ibimenyetso byerekana ko uri ingimbi icyo gihe ngo ushobora kuba urwaye ushobora kuba wajya kwa muganga ukamenya uko bimeze cyangwa se nanone kumwangavu amakuru mbona numva mbaziho nuko babwirwa yuko ikintu cya mbere babanza kubigisha nukmenya kubara iminsi yabo igihe bagira mu kwezi kwabo igihe bagira mu mihango akamenya igihe bashobora kumuterera inda akamenya igihe yayisamira ndetse bakamwigisha uburyo yanabyitwaramo mu gihe byabaye mu gihe imihango yaje murakoze.

Title: G S RWAMAGANA A BOYS.docx

Doc Creator: tcuhawenimana11

Doc Date: 3/20/2023

Codes Applied: Psychological changes occuring during puberty-Boys

Excerpt Creator: tcuhawenimana11

Excerpt Created On: 3/22/2023

Excerpt Range: 14845-15125

Amakuru naba nzi ku ngimbi nuko umuhungu atangira kwifuza umukobwa, atangira kugira ikigare agenderamo nyine abantu agendana nabo ndetse atangira kumva ko afite imbaraga agatangira kuba yasuzugura ababyeyi ndetse icyo ashaka ntabe yakongera ku cyaka ababyeyi akakishakira we ubwe.

Title: G S RWAMAGANA A BOYS.docx

Doc Creator: tcuhawenimana11

Doc Date: 3/20/2023

Codes Applied: Information that girls need

Excerpt Creator: tcuhawenimana11

Excerpt Created On: 3/22/2023

Excerpt Range: 15126-15427

Noneho kumwangavu we amakuru naba nzi nuko agirwa inama yo kujya kwiteresha urushinge rwinkondi yumura, atangira kuba yatangira kugendana ibikoresho yakenera mu gihe cyimihango yakwambara cyangwa se akagirwa inama yo kuvuga oya mu gihe ahuye numuntu umushuka ngo bakore imibonanompuzabitsina murakoze.

Title: G S RWAMAGANA A BOYS.docx

Doc Creator: tcuhawenimana11

Doc Date: 3/20/2023

Codes Applied: Information that boys need

Excerpt Creator: tcuhawenimana11

Excerpt Created On: 3/22/2023

Excerpt Range: 15463-15639

Inama agirwa agirwa inama yo kwirinda kugendera mu kigare cyabantu bafite ingeso mbi, agirwa inama yo kwirinda kwiyahuza ibiyobyabwenge cyangwa kwishora mu ngeso sitari nziza.

Title: G S RWAMAGANA A BOYS.docx

Doc Creator: tcuhawenimana11

Doc Date: 3/20/2023

Codes Applied: Obstacles and risks experienced by boys during the sexual and reproductive health changes occuring during puberty and adolescence

Excerpt Creator: tcuhawenimana11

Excerpt Created On: 3/22/2023

Excerpt Range: 15640-15836

Ese muricyo kigare nkingeso mbi zishobora kubamo nkinkizihe? Ingeso mbi zishobora kubamo ni nko kunywa ibiyobyabwenge, kwiba, gufata abana babandi kungufu ndetse nizindi ngeso nyine zitari nziza.

Title: G S RWAMAGANA A BOYS.docx

Doc Creator: tcuhawenimana11

Doc Date: 3/20/2023

Codes Applied: Sources of information received during puberty

Excerpt Creator: tcuhawenimana11

Excerpt Created On: 3/22/2023

Excerpt Range: 16182-16367

Aho naba narakue ayo makuru mu gihe byakubayeho ubaza nyine umuntu mukuru ubona ko nawe yabiciyemo akagusobanurira. Ubwo ni nka bakuru bawe cyangwa abagukuriye mu myaka yo hejuru? Yego.

Title: G S RWAMAGANA A BOYS.docx

Doc Creator: tcuhawenimana11

Doc Date: 3/20/2023

Codes Applied: Sources of information received during puberty

Excerpt Creator: tcuhawenimana11

Excerpt Created On: 3/22/2023

Excerpt Range: 16435-16556

Amakuru ahantu hambere nayavanye nagiye mbyumva, ahantu ha kabiri ni kukigonderabuzima no ku babyeyi nkabavanamo amakuru.

Title: G S RWAMAGANA A BOYS.docx

Doc Creator: tcuhawenimana11

Doc Date: 3/20/2023

Codes Applied: Sources of information received during puberty

Excerpt Creator: tcuhawenimana11

Excerpt Created On: 3/22/2023

Excerpt Range: 16910-17112

Nyine ahantu amakuru yose aba ari menshi ni kukigonderabuzima, nababyeyi erega hari igihe ubaza bataraciye mwishuri bakakubwira ayo bazo ariko kukigonderabuzima haba hari abaganga babyigiye bose babizi.

Title: G S RWAMAGANA A BOYS.docx

Doc Creator: tcuhawenimana11

Doc Date: 3/20/2023

Codes Applied: Sources of information received during puberty

Excerpt Creator: tcuhawenimana11

Excerpt Created On: 3/22/2023

Excerpt Range: 17198-17617

Amakuru menshi nagiye nyakura kuri bagenzi bange kwakundi mwicarana nabana muri karitsiye cyangwa nkiri umu petit mutoya nkijya gusenya nkumva abandi babivuga cyangwa se nanyura nkahantu igihe waba uri gukorana nabantu mu kazi nkatwe dukunda gukora akazi abantu bubaka bo biba bibari mu bwonko. Murakora bakabivuga nawe ugutwi wakubanguye bagenzi bawe nabo barabivuga mbega hari ahantu henshi umuntu aba agenda abikura.

Title: G S RWAMAGANA A BOYS.docx

Doc Creator: tcuhawenimana11

Doc Date: 3/20/2023

Codes Applied: Received information is misleading and risky

Excerpt Creator: tcuhawenimana11

Excerpt Created On: 3/22/2023

Excerpt Range: 17732-18001

Amakuru yo ku mashatsiye ntakubeshye baba bavuga ibintu byukuri kuko baba barabikoze, cyane cyane ibintu bakunda kuvuga ni ibintu byubsambanyi cyane ariko nyine ntabwo uba urabikra ngo ubimenye ako ibintu baba bavuga uba wumva ari ibintu byukuri ariko ntanama bakugira.

Title: G S RWAMAGANA A BOYS.docx

Doc Creator: tcuhawenimana11

Doc Date: 3/20/2023

Codes Applied: Obstacles and risks experienced by boys during the sexual and reproductive health changes occuring during puberty and adolescence

Excerpt Creator: tcuhawenimana11

Excerpt Created On: 3/22/2023

Excerpt Range: 18422-18870

Ingorane ingimbi zahura nazo harimo nko kuvuga ko wenda bameze nkinsya bikabatera isoni mu bandi bantu bagenzi babo bikabatera ipfunwe cyangwa se bakumva ngo yameze incakwaha bagatangira kuguserereza bavuga ngo urashaje, ngo uri mukuru ngo uraturuta ngo rero ujya kuba uri kumwe nabo bantu munga ngo twebwe uraturuta. Hari ahantu uvuze ngo wenda yarameze nkinsya none se murerekanana? Oya wenda hari igihe muba muri kuganira akakubwira ko azifite.

Title: G S RWAMAGANA A BOYS.docx

Doc Creator: tcuhawenimana11

Doc Date: 3/20/2023

Codes Applied: Obstacles and risks experienced by boys during the sexual and reproductive health changes occuring during puberty and adolescence

Excerpt Creator: tcuhawenimana11

Excerpt Created On: 3/22/2023

Excerpt Range: 19037-20058

Ingorane akenshi tujya dukunda guhura nazo iyo ugeze muricyo gihe utangira kwironda ukavuga ati wabona nkumuntu ugasanga umurusha nkumwaka umwe ariko ukavuga ati uyunguyu sinagendana nawe, bakabigukora cyangwa se wowe ukabibakora urumva ko iyongiyo ari ingorane, nanone ushobora kumva ko wakuze urenze abandi wabona ko uriya hari ibintu yagezeho nawe ukumva ushatse kubigeraho ugasanga agukoresheje amafuti we afite imbaraga akora akazi, abashaka kugura icyo akeneye kuko ntambaraga zibikora ufite ugasanga ubashije nko kujya kwiba ugasanga uhuye ningorane nkiyongiyo ukiba bakagufunga cyangwa se ushobora guhinduka mu mubiri wawe ukumva urashaka ngo gusambana kwifata bikakunanira ugasanga ukoze icyo cyaha cyo gusambana nyine niyo ni ingorane kuko ushobora gukura nkindwara cyagwa ugasanga uteye nkinda kuko iyo wageze muri iyo myaka biba bishoboka ko waba watera inda urumva ko iyo ari ingorane ushobora kuba wahura nayo ikakubera mbi cyane kuba watera inda cyangwa ukaba wakwandura butewe niyo myaka ugezemo murakoze.

Title: G S RWAMAGANA A BOYS.docx

Doc Creator: tcuhawenimana11

Doc Date: 3/20/2023

Codes Applied: Obstacles and risks experienced by boys during the sexual and reproductive health changes occuring during puberty and adolescence

Excerpt Creator: tcuhawenimana11

Excerpt Created On: 3/22/2023

Excerpt Range: 20257-20782

Uwo twaba tuzi, ntabwo habura umuntu waba uzi byabayeho ushobora nko gusanga umuntu afite nkimyaka nka 17-18 ugasanga yamaze kwiyumvisha yuko atakundana numuntu ufite iyo myaka nawe ugasanga we arashaka gukundana numuntu ufite imyaka nka 25 gutyo amuruta imyaka myinshi ifatika cyangwa se biba bishoboka kuba ku muntu cyane nkubu umuntu mukuru ukamukurikiraho amafaranga se bikarangira nyine agushoye nko mu mico mibi yubusambanyi nuko agushukishije amafaranga urabyemeye ukandura indwara ugasanga ubuzima bwawe burangiritse.

Title: G S RWAMAGANA A BOYS.docx

Doc Creator: tcuhawenimana11

Doc Date: 3/20/2023

Codes Applied: Obstacles and risks experienced by boys during the sexual and reproductive health changes occuring during puberty and adolescence

Excerpt Creator: tcuhawenimana11

Excerpt Created On: 3/22/2023

Excerpt Range: 20894-21168

Ingorane ushobora guhura nazo ushobora guhura nabandi bagushuka ugatangira guta umurongo imico warufite igahinduka, ugatangira gutaha utinze usuzugura ababyeyi udakora imirimo ngewe numva izo arizo ngorane ushobora guhura nazo bitewe naho uri cyangwa nabo muhura bagushuka.

Title: G S RWAMAGANA A BOYS.docx

Doc Creator: tcuhawenimana11

Doc Date: 3/20/2023

Codes Applied: Physiological changes occuring during puberty-Boys

Excerpt Creator: tcuhawenimana11

Excerpt Created On: 3/22/2023

Excerpt Range: 21305-21523

Ubundi iyo ugeze mu gihe cyingimbi ibiryo waryaga ushobora kubikuba 2 bitewe nuko umeze, hari igihe uba wumva ibiryo baguhaye ari nkabikeya ukumva ushaka kurya cyane nyine hari igihe apetit yiyongera bitewe nuko umeze.

Title: G S RWAMAGANA A BOYS.docx

Doc Creator: tcuhawenimana11

Doc Date: 3/20/2023

Codes Applied: Obstacles and risks experienced by boys during the sexual and reproductive health changes occuring during puberty and adolescence

Excerpt Creator: tcuhawenimana11

Excerpt Created On: 3/22/2023

Excerpt Range: 21563-21730

Ingorane bishobora kuyitera bitewe nuko uri kwishuri ushobora kugira ubusambo ugasanga utangiye ingeso mbi zo kurwanira nabagenzi bawe ibiryo ugasanga ntago ari byiza.

Title: G S RWAMAGANA A BOYS.docx

Doc Creator: tcuhawenimana11

Doc Date: 3/20/2023

Codes Applied: Obstacles and risks experienced by girls during sexual and reproductive health changes occuring during puberty and adolescence

Excerpt Creator: tcuhawenimana11

Excerpt Created On: 3/22/2023

Excerpt Range: 21881-22217

Ingorane numva numva ko wenda igihe abakobwa bageze mu mihango hari amakuru wenda babaha Atari meza bakababwira ngo niba ujya mihango ukababara wenda ngo kugirango bikire nuko uryamana numuhungu ubwo baryamana numuhungu bagakuramo ingaruka zo kuba bakandura indwara zandurira mu mibonanompuzabitsina ninda zitateguwe eeee ndumva aribyo.

Title: G S RWAMAGANA A BOYS.docx

Doc Creator: tcuhawenimana11

Doc Date: 3/20/2023

Codes Applied: Obstacles and risks experienced by girls during sexual and reproductive health changes occuring during puberty and adolescence

Excerpt Creator: tcuhawenimana11

Excerpt Created On: 3/22/2023

Excerpt Range: 22481-22730

nzitizi bagira hari nkigihe nyine nkumubyeyi abona umwana we ari kwitwara mu bintu bitari byiza akamwihorera ugasanga nyine ni ababyeyi nabo bakeneye inyigisho ariko nyine kubera ko habayeyo ibigo nderabuzima ukajyayo bakakugira inama ntubyishoremo.

Title: G S RWAMAGANA A BOYS.docx

Doc Creator: tcuhawenimana11

Doc Date: 3/20/2023

Codes Applied: Obstacles and risks experienced by girls during sexual and reproductive health changes occuring during puberty and adolescence

Excerpt Creator: tcuhawenimana11

Excerpt Created On: 3/22/2023

Excerpt Range: 22932-23086

Inzitizi abari bahura nazo nuko bashobora gutwita bamara gutwita bakumva bakuramo iyo nda kandi niyo nda bagiye kuvanamo bashobora kuvutswa ubuzima bwabo.

Title: G S RWAMAGANA A BOYS.docx

Doc Creator: tcuhawenimana11

Doc Date: 3/20/2023

Codes Applied: Obstacles and risks experienced by girls during sexual and reproductive health changes occuring during puberty and adolescence

Excerpt Creator: tcuhawenimana11

Excerpt Created On: 3/22/2023

Excerpt Range: 23351-23510

Inzitizi numva ari nko kugira ababyeyi babi babasinzi ushobora wenda nkumuturanyi muturanye ukabona afite abana bafite imico myiza bakajya babegera bakamubaza.

Title: G S RWAMAGANA A BOYS.docx

Doc Creator: tcuhawenimana11

Doc Date: 3/20/2023

Codes Applied: Obstacles and risks experienced by girls during sexual and reproductive health changes occuring during puberty and adolescence

Excerpt Creator: tcuhawenimana11

Excerpt Created On: 3/22/2023

Excerpt Range: 23581-23749

Inzitizi numva ni nko kugirwa inama mbi ubajije inama cyangwa se nkuko mugenzi wange yarabivuze kugira ababyeyi babi no kugira ikigare kibi cyabagenzi bawe ugenda nabo.

Title: G S RWAMAGANA A BOYS.docx

Doc Creator: tcuhawenimana11

Doc Date: 3/20/2023

Codes Applied: Obstacles and risks experienced by boys during the sexual and reproductive health changes occuring during puberty and adolescence

Excerpt Creator: tcuhawenimana11

Excerpt Created On: 3/22/2023

Excerpt Range: 23879-24667

Inzitizi bashobora guhura nazo nuko bashobora no kwiroteraho bakabona ibimenyetso bigaragaza ko batangiye gukura nko kumera insya, incakwaha no kumera amabere bakifuza kubibwira ababyeyi babo cyangwa kujya kukigonderabuzima hanyuma bakwegera yenda nabo babyeyi babo, abo babyeyi babo ntibifuze kubaha amakuru yuzuye ahubwo bakababwira ati ntabwo murakura kuburyo mwabaza ibyo bintu mbese bakababwira ngo muracyari abana hanyuma bakubwira ngo nawe uracyari umwana ukumva ucitse intege ukumva utakongera no kubabaza, noneho hari nigihe ugendera mu kigare cyiza abo bagenzi bawe ugendana nabo bakakubwira ati wicika intege jya kukigonderabuzima ubwo iyo ugiye kukigonderabuzima bo baguha amakuru meza ntihagire inzitizi uhura nazo ariko hari nigihe baguha amakuru ukumva nawe suyizeye neza.

Title: G S RWAMAGANA A BOYS.docx

Doc Creator: tcuhawenimana11

Doc Date: 3/20/2023

Codes Applied: Suggestions to improve SRH during puberty and adolescence by boys

Excerpt Creator: tcuhawenimana11

Excerpt Created On: 3/22/2023

Excerpt Range: 25048-25284

Icyo numva navuga nuko ababyeyi bacu batugira inama ku gihe ndetse bakadufasha kutwereka inzira nziza twanyuramo, abantu twagendana nabo ndetse tukaba twabafatira umwanya tukajya hamwe tukaba twabiganiraho nabandi tukagira imico myiza.

Title: G S RWAMAGANA A BOYS.docx

Doc Creator: tcuhawenimana11

Doc Date: 3/20/2023

Codes Applied: Suggestions to improve SRH during puberty and adolescence for girls

Excerpt Creator: tcuhawenimana11

Excerpt Created On: 3/22/2023

Excerpt Range: 25320-25534

Numva ngewe ngirango kurubyiruko rubashe kubona amakuru yukuri habaho nkubukangurambanga mu rubyiruko no mu bantu bakuru kugirango babantu bakuru batabizi batinyuke baganirize abana babo na babana batinyuke kubaza

Title: G S RWAMAGANA A BOYS.docx

Doc Creator: tcuhawenimana11

Doc Date: 3/20/2023

Codes Applied: Suggestions to improve SRH during puberty and adolescence by boys

Excerpt Creator: tcuhawenimana11

Excerpt Created On: 3/22/2023

Excerpt Range: 25320-25534

Numva ngewe ngirango kurubyiruko rubashe kubona amakuru yukuri habaho nkubukangurambanga mu rubyiruko no mu bantu bakuru kugirango babantu bakuru batabizi batinyuke baganirize abana babo na babana batinyuke kubaza

Title: Focus group discussion-Kiziguro boys Transc.docx

Doc Creator: tcuhawenimana11

Doc Date: 3/20/2023

Codes Applied: Physiological changes occuring during puberty-Boys

Excerpt Creator: tcuhawenimana11

Excerpt Created On: 3/21/2023

Excerpt Range: 398-482

impinduka ziba ku muhungu nuko iyo amaze gukura yiroteraho bigaterwa n’imisemburo ye

Title: Focus group discussion-Kiziguro boys Transc.docx

Doc Creator: tcuhawenimana11

Doc Date: 3/20/2023

Codes Applied: Age

Excerpt Creator: tcuhawenimana11

Excerpt Created On: 3/21/2023

Excerpt Range: 678-694

Mfite imyaka 18

Title: Focus group discussion-Kiziguro boys Transc.docx

Doc Creator: tcuhawenimana11

Doc Date: 3/20/2023

Codes Applied: Physiological changes occuring during puberty-Boys

Excerpt Creator: tcuhawenimana11

Excerpt Created On: 3/21/2023

Excerpt Range: 714-949

Impinduka ziba ku mwana w’umuhungu mu gihe amaze kugera mu gihe cy’ubugimbi ni nko kuniga ijwi. Iyo anize ijwi ni ikimenyetso cy’uko aba avuye mu kiciro kimwe agiye mu kindi. Atangira kwiroteraho ibyo nabyo bikaba biterwa n’imisemburo.

Title: Focus group discussion-Kiziguro boys Transc.docx

Doc Creator: tcuhawenimana11

Doc Date: 3/20/2023

Codes Applied: Psychological changes occuring during puberty-Boys

Excerpt Creator: tcuhawenimana11

Excerpt Created On: 3/21/2023

Excerpt Range: 950-1088

Ni igihe kiba kimwereka yuko aramutse akoranye imibonano mpuzabitsina n’umukobwa ashobora kuba yamutera inda mu gihe babikoze batikingiye.

Title: Focus group discussion-Kiziguro boys Transc.docx

Doc Creator: tcuhawenimana11

Doc Date: 3/20/2023

Codes Applied: Psychological changes occuring during puberty-Boys

Excerpt Creator: tcuhawenimana11

Excerpt Created On: 3/21/2023

Excerpt Range: 1241-1360

Njyewe impinduka nzi nuko iyo umuhungu ageze mu gihe cy’ubugimbi atangira kuzana ibiheri mu maso akanamera inshakwaha.

Title: Focus group discussion-Kiziguro boys Transc.docx

Doc Creator: tcuhawenimana11

Doc Date: 3/20/2023

Codes Applied: Physiological changes occuring during puberty-Boys

Excerpt Creator: tcuhawenimana11

Excerpt Created On: 3/21/2023

Excerpt Range: 1369-1474

Impinduka ziba ku mwana w’umuhungu ugeze mu gihe cy’ubugimbi ashobora kuzana igituza akanamera impwempwe.

Title: Focus group discussion-Kiziguro boys Transc.docx

Doc Creator: tcuhawenimana11

Doc Date: 3/20/2023

Codes Applied: Physiological changes occuring during puberty-Boys

Excerpt Creator: tcuhawenimana11

Excerpt Created On: 3/21/2023

Excerpt Range: 1520-1591

Iyo umuhungu atangiye gukora azana insya n’ibindi bigaragaza ko yakuze

Title: Focus group discussion-Kiziguro boys Transc.docx

Doc Creator: tcuhawenimana11

Doc Date: 3/20/2023

Codes Applied: Physiological changes occuring during puberty-Boys

Excerpt Creator: tcuhawenimana11

Excerpt Created On: 3/21/2023

Excerpt Range: 1619-1641

Nk’inshakwaha n’insya.

Title: Focus group discussion-Kiziguro boys Transc.docx

Doc Creator: tcuhawenimana11

Doc Date: 3/20/2023

Codes Applied: Physiological changes occuring during puberty-Boys

Excerpt Creator: tcuhawenimana11

Excerpt Created On: 3/21/2023

Excerpt Range: 1724-1806

Impinduka ziba ku mwana w’umuhungu ugeze mu gihe cy’ubugimbi atangira kuniga ijwi.

Title: Focus group discussion-Kiziguro boys Transc.docx

Doc Creator: tcuhawenimana11

Doc Date: 3/20/2023

Codes Applied: Boys and girls know about the causes of the causes leading to the physiological changes during puberty

Excerpt Creator: tcuhawenimana11

Excerpt Created On: 3/21/2023

Excerpt Range: 1845-1860

Ni imisemburo.

Title: Focus group discussion-Kiziguro boys Transc.docx

Doc Creator: tcuhawenimana11

Doc Date: 3/20/2023

Codes Applied: Age

Excerpt Creator: tcuhawenimana11

Excerpt Created On: 3/21/2023

Excerpt Range: 1896-1916

Imyaka yanjye ni 19

Title: Focus group discussion-Kiziguro boys Transc.docx

Doc Creator: tcuhawenimana11

Doc Date: 3/20/2023

Codes Applied: Physiological changes occuring during puberty-Boys

Excerpt Creator: tcuhawenimana11

Excerpt Created On: 3/21/2023

Excerpt Range: 1957-2041

Njyewe uko mbibona mba mbona biterwa n’igihe umuntu aba agezemo akaba yahindura ijwi

Title: Focus group discussion-Kiziguro boys Transc.docx

Doc Creator: tcuhawenimana11

Doc Date: 3/20/2023

Codes Applied: Psychological changes occuring during puberty-Boys

Excerpt Creator: tcuhawenimana11

Excerpt Created On: 3/21/2023

Excerpt Range: 2042-2193

noneho akumva ko ari umusore kurusha abandi, nyine akumva ni umusore ku rwego rwo hejuru, noneho akumva yatangira kuvugisha uwo abonye nyine yishimiye.

Title: Focus group discussion-Kiziguro boys Transc.docx

Doc Creator: tcuhawenimana11

Doc Date: 3/20/2023

Codes Applied: Psychological changes occuring during puberty-Boys

Excerpt Creator: tcuhawenimana11

Excerpt Created On: 3/21/2023

Excerpt Range: 2233-2338

Oya ntabwo ari uwo ari we wese amarangamutima ye nyine amwerekeza ku mukobwa kandi no ku bakobwa bibaho.

Title: Focus group discussion-Kiziguro boys Transc.docx

Doc Creator: tcuhawenimana11

Doc Date: 3/20/2023

Codes Applied: Age

Excerpt Creator: tcuhawenimana11

Excerpt Created On: 3/21/2023

Excerpt Range: 2484-2500

Mfite imyaka 18.

Title: Focus group discussion-Kiziguro boys Transc.docx

Doc Creator: tcuhawenimana11

Doc Date: 3/20/2023

Codes Applied: Physiological changes occuring during puberty-Boys

Excerpt Creator: tcuhawenimana11

Excerpt Created On: 3/21/2023

Excerpt Range: 2501-2665

Njyewe ikintu mbona ku mpinduka ku muhungu utangiye kugera mu gihe cy’ubugimbi nkuko bagenzi banjye babivuze ijwi rirahinduka, igihagararo mu gihagararo physically

Title: Focus group discussion-Kiziguro boys Transc.docx

Doc Creator: tcuhawenimana11

Doc Date: 3/20/2023

Codes Applied: Psychological changes occuring during puberty-Boys

Excerpt Creator: tcuhawenimana11

Excerpt Created On: 3/21/2023

Excerpt Range: 2665-2795

atangira kwiyumva yakuze yabaye umuntu w’umugabo kandi yumva ageze mu gihe cyo kwifatira imyanzuro eee no kugira inama bagenzi be.

Title: Focus group discussion-Kiziguro boys Transc.docx

Doc Creator: tcuhawenimana11

Doc Date: 3/20/2023

Codes Applied: Psychological changes occuring during puberty-Boys

Excerpt Creator: tcuhawenimana11

Excerpt Created On: 3/21/2023

Excerpt Range: 2886-3006

Numva ko mu gihe cy’ubugimbi umuhungu we yifatira umwanzuro mu gihe yamaze kumva ko yakuze mbese aba yumva ntawamubasha.

Title: Focus group discussion-Kiziguro boys Transc.docx

Doc Creator: tcuhawenimana11

Doc Date: 3/20/2023

Codes Applied: Physiological changes occuring during puberty-Boys

Excerpt Creator: tcuhawenimana11

Excerpt Created On: 3/21/2023

Excerpt Range: 3091-3165

Njyewe impinduka numva ingimbi zikunze kugiraho nuko batangira kwiroteraho

Title: Focus group discussion-Kiziguro boys Transc.docx

Doc Creator: tcuhawenimana11

Doc Date: 3/20/2023

Codes Applied: Psychological changes occuring during puberty-Boys

Excerpt Creator: tcuhawenimana11

Excerpt Created On: 3/21/2023

Excerpt Range: 3166-3247

yarangiza nyine akumva ashaka kubonana n’abakobwa cyangwa se bamwe bakanikinisha.

Title: Focus group discussion-Kiziguro boys Transc.docx

Doc Creator: tcuhawenimana11

Doc Date: 3/20/2023

Codes Applied: Boys and girls know about the causes of the causes leading to the physiological changes during puberty

Excerpt Creator: tcuhawenimana11

Excerpt Created On: 3/21/2023

Excerpt Range: 3273-3531

Njyewe uko mbibona iyo atangiye kwiroteraho biterwa nuko umusemburo we umuryanamo bigarwa n’ibiryo arya kuko umwana urira ku gihe hakaba n’utarira ku gihe ntabwo bakwiroteraho kimwe. Hari uwiroteraho igihe gitoya hakaba uwiroteraho afite igihagararo gitoya …

Title: Focus group discussion-Kiziguro boys Transc.docx

Doc Creator: tcuhawenimana11

Doc Date: 3/20/2023

Codes Applied: Psychological changes occuring during puberty-Boys

Excerpt Creator: tcuhawenimana11

Excerpt Created On: 3/21/2023

Excerpt Range: 3651-3775

Nabyo bibaho niba wenda nk’umuhungu yatahaga sa mboya sambili agatangira kongera amasaha, agatangira kutumvikana n’ababyeyi.

Title: Focus group discussion-Kiziguro boys Transc.docx

Doc Creator: tcuhawenimana11

Doc Date: 3/20/2023

Codes Applied: Psychological changes occuring during puberty-Boys

Excerpt Creator: tcuhawenimana11

Excerpt Created On: 3/21/2023

Excerpt Range: 3869-4145

Murakoze njye icyo nakongeraho igihe umuhungu atangiye kugera mu gihe cy’ubugimbi akenshi nk’iyo abana n’ababyeyi usanga akenshi yifuza ko ataba mu rugo, akumva ari we mugabo mu rugo, akenshi iyo abona se ahari nawe ahari aba abona bangana bikarangira ubona yisanzuye kuri se.

Title: Focus group discussion-Kiziguro boys Transc.docx

Doc Creator: tcuhawenimana11

Doc Date: 3/20/2023

Codes Applied: Physiological changes occuring during puberty-Girls

Excerpt Creator: tcuhawenimana11

Excerpt Created On: 3/21/2023

Excerpt Range: 4323-4432

Muri bashiki bacu impinduka bagira ni nko kumera amebere, bakazana n’incakwaha ndetse bakagira n’igihagararo.

Title: Focus group discussion-Kiziguro boys Transc.docx

Doc Creator: tcuhawenimana11

Doc Date: 3/20/2023

Codes Applied: Psychological changes occuring during puberty-Girls

Excerpt Creator: tcuhawenimana11

Excerpt Created On: 3/21/2023

Excerpt Range: 4473-4533

Ikindi ni ikintu cyo kwigirira isuku bakishyira ku cyangwe.

Title: Focus group discussion-Kiziguro boys Transc.docx

Doc Creator: tcuhawenimana11

Doc Date: 3/20/2023

Codes Applied: Physiological changes occuring during puberty-Girls

Excerpt Creator: tcuhawenimana11

Excerpt Created On: 3/21/2023

Excerpt Range: 4555-4620

Indi mpinduka iba kuri bashiki bacu bagira nuko bajya mu mihango.

Title: Focus group discussion-Kiziguro boys Transc.docx

Doc Creator: tcuhawenimana11

Doc Date: 3/20/2023

Codes Applied: Psychological changes occuring during puberty-Girls

Excerpt Creator: tcuhawenimana11

Excerpt Created On: 3/21/2023

Excerpt Range: 4694-4792

Njyewe icyo mbaziho iyo bageze mu myaka y’ubwangavu batangira guhindura imyitwarire mu myambarire.

Title: Focus group discussion-Kiziguro boys Transc.docx

Doc Creator: tcuhawenimana11

Doc Date: 3/20/2023

Codes Applied: Psychological changes occuring during puberty-Girls

Excerpt Creator: tcuhawenimana11

Excerpt Created On: 3/21/2023

Excerpt Range: 4840-4938

Muri bashiki bacu iyo bageze mu gihe cy’ubwangavu batangira kumva bahura n’umuhungu cyane cyane.

Title: Focus group discussion-Kiziguro boys Transc.docx

Doc Creator: tcuhawenimana11

Doc Date: 3/20/2023

Codes Applied: Physiological changes occuring during puberty-Girls

Excerpt Creator: tcuhawenimana11

Excerpt Created On: 3/21/2023

Excerpt Range: 4970-5035

Kuri bashiki bacu iyo bageze mu gihe cy’ubwangavu bamera amabere.

Title: Focus group discussion-Kiziguro boys Transc.docx

Doc Creator: tcuhawenimana11

Doc Date: 3/20/2023

Codes Applied: Physiological changes occuring during puberty-Girls

Excerpt Creator: tcuhawenimana11

Excerpt Created On: 3/21/2023

Excerpt Range: 5087-5162

Batangira guhindura ijwi ukumva bafite akajwi kanyororotse mbese kari soft.

Title: Focus group discussion-Kiziguro boys Transc.docx

Doc Creator: tcuhawenimana11

Doc Date: 3/20/2023

Codes Applied: Psychological changes occuring during puberty-Girls

Excerpt Creator: tcuhawenimana11

Excerpt Created On: 3/21/2023

Excerpt Range: 5207-5432

Njyewe mbona hahinduka n’ingendo mbese uko bagendaga bakiri abana ukabona byahindutse hari ikindi cyiyongereyeho. Ikindi kintu mbona umukobwa ugeze mu gihe cy’ubwangavu ntashobora gukarabira aho abantu bamubonera agira isoni.

Title: Focus group discussion-Kiziguro boys Transc.docx

Doc Creator: tcuhawenimana11

Doc Date: 3/20/2023

Codes Applied: How boys experience physiological changes that occured to them during puberty

Excerpt Creator: tcuhawenimana11

Excerpt Created On: 3/21/2023

Excerpt Range: 5864-6220

Murakoze njyewe nagize ikimenyetso cyo kwiroteraho. Nari narategereje igihe kirekire hanyuma umunsi umwe nza kurota nakoze sex n’umukobwa hanyuma mbyutse nsanga mu mashuka hari sperms ndibaza nti byagenze gute. Byari ku wa gatandatu ntangira kwibaza nti ninsohora amashuka nkajya kuyafura mu rugo baratangira kumbaza ngo byagenze gute habaye ikihe kibazo.

Title: Focus group discussion-Kiziguro boys Transc.docx

Doc Creator: tcuhawenimana11

Doc Date: 3/20/2023

Codes Applied: Precautions to take during puberty for boys Sources of information received during puberty

Excerpt Creator: tcuhawenimana11

Excerpt Created On: 3/21/2023

Excerpt Range: 6220-6828

Ubwo birangira nigiriye inama yo kujya kwegera mere wanjye kuko mere wanjye dusanzwe tuganira ubwo ndamubaza nti gutya na gutya nuko arambwira ati ni ibintu bisanzwe bibaho mu buzima, arambwira ati genda ufure turaza kuganira nyuma. Ndafura nsoje ndagenda turaganira arambwira ati rero ibyakubayeho ni ikimenyetso kigaragaza ko wakuze utazibeshya ngo ushake gukorana imibonano n’umukobwa kuko ni ikimenyetso kerekana ko ushobora kuba watera inda ati umuntu wese mushobora kuba mwaryamana utitaye kukuvuga ngo sindi mukuru cyane cyangwa yaba mutoya ariko ageze mu gihe cyo kuba nawe yasama inda wamutera inda.

Title: Focus group discussion-Kiziguro boys Transc.docx

Doc Creator: tcuhawenimana11

Doc Date: 3/20/2023

Codes Applied: Received information is right and provides sufficient information helping boys and girls during puberty

Excerpt Creator: tcuhawenimana11

Excerpt Created On: 3/21/2023

Excerpt Range: 6453-6828

Ndafura nsoje ndagenda turaganira arambwira ati rero ibyakubayeho ni ikimenyetso kigaragaza ko wakuze utazibeshya ngo ushake gukorana imibonano n’umukobwa kuko ni ikimenyetso kerekana ko ushobora kuba watera inda ati umuntu wese mushobora kuba mwaryamana utitaye kukuvuga ngo sindi mukuru cyane cyangwa yaba mutoya ariko ageze mu gihe cyo kuba nawe yasama inda wamutera inda.

Title: Focus group discussion-Kiziguro boys Transc.docx

Doc Creator: tcuhawenimana11

Doc Date: 3/20/2023

Codes Applied: How boys experience physiological changes that occured to them during puberty

Excerpt Creator: tcuhawenimana11

Excerpt Created On: 3/21/2023

Excerpt Range: 6935-7001

Nanjye nameze ubushakwaha njye ntakindi nakoze nabibwiye ababyeyi.

Title: Focus group discussion-Kiziguro boys Transc.docx

Doc Creator: tcuhawenimana11

Doc Date: 3/20/2023

Codes Applied: How to take care of themselves when phsyiological changes occur for boys

Excerpt Creator: tcuhawenimana11

Excerpt Created On: 3/21/2023

Excerpt Range: 7052-7207

Nabibabwiye bombi ubwo bambwira ko umwanzuro ntawundi ari ukuzogosha kugira ngo nirinde umwana. Ubwo babimfashamo iyo mikasi barayingurira nkajya nzogosha.

Title: Focus group discussion-Kiziguro boys Transc.docx

Doc Creator: tcuhawenimana11

Doc Date: 3/20/2023

Codes Applied: How boys experience physiological changes that occured to them during puberty

Excerpt Creator: tcuhawenimana11

Excerpt Created On: 3/21/2023

Excerpt Range: 7269-7411

Njyewe nyine nabonye nireteyeho mbibwira umubyeyi hanyuma arambwira ibyambaho hanyuma agatangira kumbuza kuzajya nkora imibonano mpuzabitsina.

Title: Focus group discussion-Kiziguro boys Transc.docx

Doc Creator: tcuhawenimana11

Doc Date: 3/20/2023

Codes Applied: Sources of information received during puberty

Excerpt Creator: tcuhawenimana11

Excerpt Created On: 3/21/2023

Excerpt Range: 7488-7500

Nabwiye mama

Title: Focus group discussion-Kiziguro boys Transc.docx

Doc Creator: tcuhawenimana11

Doc Date: 3/20/2023

Codes Applied: How boys experience physiological changes that occured to them during puberty

Excerpt Creator: tcuhawenimana11

Excerpt Created On: 3/21/2023

Excerpt Range: 7608-7791

Njyewe byatangiye niyumva mu rugo ntamuntu wankoraho nkazajya numva mfite imbaraga ariko ababyeyi banjye bangira inama bambwira ko nazaza nitwara neza kugira ngo ntazahura n’ingorane.

Title: Focus group discussion-Kiziguro boys Transc.docx

Doc Creator: tcuhawenimana11

Doc Date: 3/20/2023

Codes Applied: Precautions to take during puberty for boys

Excerpt Creator: tcuhawenimana11

Excerpt Created On: 3/21/2023

Excerpt Range: 7697-7791

ababyeyi banjye bangira inama bambwira ko nazaza nitwara neza kugira ngo ntazahura n’ingorane.

Title: Focus group discussion-Kiziguro boys Transc.docx

Doc Creator: tcuhawenimana11

Doc Date: 3/20/2023

Codes Applied: How boys experience physiological changes that occured to them during puberty

Excerpt Creator: tcuhawenimana11

Excerpt Created On: 3/21/2023

Excerpt Range: 8057-8283

Njye natangiye kwiyumva ko ndi mukuru nkareba ibyo bashiki banjye bakora bimwe nkabona bidakwiye rimwe nkaba nabakubita. Gusa ababyeyi baje kungira inama bambwira ko nkwiye kureba amahane kandi ko ngomba kwitwara neza mu rugo.

Title: Focus group discussion-Kiziguro boys Transc.docx

Doc Creator: tcuhawenimana11

Doc Date: 3/20/2023

Codes Applied: Precautions to take during puberty for boys

Excerpt Creator: tcuhawenimana11

Excerpt Created On: 3/21/2023

Excerpt Range: 8178-8283

Gusa ababyeyi baje kungira inama bambwira ko nkwiye kureba amahane kandi ko ngomba kwitwara neza mu rugo.

Title: Focus group discussion-Kiziguro boys Transc.docx

Doc Creator: tcuhawenimana11

Doc Date: 3/20/2023

Codes Applied: How boys experience physiological changes that occured to them during puberty

Excerpt Creator: tcuhawenimana11

Excerpt Created On: 3/21/2023

Excerpt Range: 8338-8388

Njye rero nameze insya narihebye nziko ntazazimera

Title: Focus group discussion-Kiziguro boys Transc.docx

Doc Creator: tcuhawenimana11

Doc Date: 3/20/2023

Codes Applied: How boys experience physiological changes that occured to them during puberty

Excerpt Creator: tcuhawenimana11

Excerpt Created On: 3/21/2023

Excerpt Range: 8442-8502

Urumva naguze akantu ka Makasi nyine ntangira kujya nogosha.

Title: Focus group discussion-Kiziguro boys Transc.docx

Doc Creator: tcuhawenimana11

Doc Date: 3/20/2023

Codes Applied: How boys experience physiological changes that occured to them during puberty

Excerpt Creator: tcuhawenimana11

Excerpt Created On: 3/21/2023

Excerpt Range: 8608-8695

Oya urumva yatangiye guhinduka nkumva mfite confidence nkumva mfite n’imbaraga nyinshi.

Title: Focus group discussion-Kiziguro boys Transc.docx

Doc Creator: tcuhawenimana11

Doc Date: 3/20/2023

Codes Applied: How boys experience physiological changes that occured to them during puberty

Excerpt Creator: tcuhawenimana11

Excerpt Created On: 3/21/2023

Excerpt Range: 8772-8981

Murakoze, njyewe bwa mbere niroteraho bijya kumbaho nta makuru kuri byo nari narigeze menya ngo mbisobanukirwe neza ariko nkimara kubibona ubwoba bwaranyishe ubwo bwarakeye nza kwishuri nirirwa hano meze neza

Title: Focus group discussion-Kiziguro boys Transc.docx

Doc Creator: tcuhawenimana11

Doc Date: 3/20/2023

Codes Applied: Precautions to take during puberty for boys Received information is right and provides sufficient information helping boys and girls during puberty

Excerpt Creator: tcuhawenimana11

Excerpt Created On: 3/21/2023

Excerpt Range: 8987-9485

hari umwarimu niyumvaho w’umugabo hanyuma tugiye muri pause ndamwegera mubwira ibyambayeho hanyuma aransobanurira anambwira uko nabyitwaramo, arambwira ati ugeze muri cya gihe ushobora gukora imibonano mpuzabitsina ukaba watera inda ndetse ukaba ari igihe ugezemo ukazajya wifuza umukobwa inshuro nyinshi ujye witwararika ku mukobwa cg umubyeyi cg se undi uwo ari we wese ushobora kugushora mu mibonano mpuzabitsina umwirinde kandi nunagerageza wenda ku bw’umubiri wakunaniye ukoreshe agakingirizo.

Title: Focus group discussion-Kiziguro boys Transc.docx

Doc Creator: tcuhawenimana11

Doc Date: 3/20/2023

Codes Applied: Sources of information received during puberty

Excerpt Creator: tcuhawenimana11

Excerpt Created On: 3/21/2023

Excerpt Range: 8987-9020

hari umwarimu niyumvaho w’umugabo

Title: Focus group discussion-Kiziguro boys Transc.docx

Doc Creator: tcuhawenimana11

Doc Date: 3/20/2023

Codes Applied: Received information is right and provides sufficient information helping boys and girls during puberty

Excerpt Creator: tcuhawenimana11

Excerpt Created On: 3/21/2023

Excerpt Range: 9542-9594

Ntabwo ndabikora ariko umunsi nabikoze nzagakoresha.

Title: Focus group discussion-Kiziguro boys Transc.docx

Doc Creator: tcuhawenimana11

Doc Date: 3/20/2023

Codes Applied: How boys experience physiological changes that occured to them during puberty

Excerpt Creator: tcuhawenimana11

Excerpt Created On: 3/21/2023

Excerpt Range: 9675-9930

Njye rero sindagakoresha kuko niyo mibonano sindayikora. Gusa mera insya cyane ko byaje byaratinze numvaga nameze insya nkumva ari igitangaza. hanyuma rero uko zigenda ziza zikura nkajya ngenda ngenda nzogosha cyane ko abandi bambwiraga ko aribwo zikora.

Title: Focus group discussion-Kiziguro boys Transc.docx

Doc Creator: tcuhawenimana11

Doc Date: 3/20/2023

Codes Applied: SRH issues young boys and girls might might not be aware of during their puberty

Excerpt Creator: tcuhawenimana11

Excerpt Created On: 3/21/2023

Excerpt Range: 10175-10393

Hari igihe umuntu aba atazi iby’izo mpinduka yarangiza ugasanga agiye mu ndaya ugasanga ahuye na accident kubera ko aba atari azi ibyo aribyo ubwo nyine byaba ari byiza ko igihe yabona umubiri uhindutse akagirwa inama.

Title: Focus group discussion-Kiziguro boys Transc.docx

Doc Creator: tcuhawenimana11

Doc Date: 3/20/2023

Codes Applied: Sources of information received during puberty

Excerpt Creator: tcuhawenimana11

Excerpt Created On: 3/21/2023

Excerpt Range: 10449-10476

Ni ukubaza nyine umubyeyi

Title: Focus group discussion-Kiziguro boys Transc.docx

Doc Creator: tcuhawenimana11

Doc Date: 3/20/2023

Codes Applied: Information that boys need

Excerpt Creator: tcuhawenimana11

Excerpt Created On: 3/21/2023

Excerpt Range: 10562-10646

Umwana w’umuhungu nk’urubyiruko mbona yakenera amakuru y’uko bakoresha agakingirizo.

Title: Focus group discussion-Kiziguro boys Transc.docx

Doc Creator: tcuhawenimana11

Doc Date: 3/20/2023

Codes Applied: Sources of information received during puberty

Excerpt Creator: tcuhawenimana11

Excerpt Created On: 3/21/2023

Excerpt Range: 10647-10755

Hano nyine hari ikigo cy’urubyiruko aho twakura amakuru kuko ibyo byose bijyanye n’imyororokere barabyigisha

Title: Focus group discussion-Kiziguro boys Transc.docx

Doc Creator: tcuhawenimana11

Doc Date: 3/20/2023

Codes Applied: Information that boys need

Excerpt Creator: tcuhawenimana11

Excerpt Created On: 3/21/2023

Excerpt Range: 10756-10869

mbese ibintu bijyanye no gukora imibonano mpuzabitsina hari byinshi batwigisha. Hari kwifata no kureka izo nzoga

Title: Focus group discussion-Kiziguro boys Transc.docx

Doc Creator: tcuhawenimana11

Doc Date: 3/20/2023

Codes Applied: Information that boys need

Excerpt Creator: tcuhawenimana11

Excerpt Created On: 3/21/2023

Excerpt Range: 10971-11150

Amakuru umwana w’umuhungu yakenera ageze mu gihe cy’ubugimbi nuko yajya aganirizwa ku bijyanye n’imibonano mpuzabitsina kugira ngo igihe nikigera atazatungurwa yisanze mu bibazo.

Title: Focus group discussion-Kiziguro boys Transc.docx

Doc Creator: tcuhawenimana11

Doc Date: 3/20/2023

Codes Applied: Information that boys need

Excerpt Creator: tcuhawenimana11

Excerpt Created On: 3/21/2023

Excerpt Range: 11207-11358

Njyewe ikindi nakongeraho, hashishikarizwa abahungu cyangwa abakobwa bakaba bajya mu nsengero bagakurikirana inyigisho z’abakuru kuko nazo zirabafasha.

Title: Focus group discussion-Kiziguro boys Transc.docx

Doc Creator: tcuhawenimana11

Doc Date: 3/20/2023

Codes Applied: Information that girls need

Excerpt Creator: tcuhawenimana11

Excerpt Created On: 3/21/2023

Excerpt Range: 11420-11700

Numva umukobwa w’umwangavu akeneye kumenya ko isaha n’isaha yakoze imibonano mpuzabitsina n’umuhungu ashobora kumutera inda akamwangiriza iterambere cg se umwana w’umuhungu ashobora gutera inda uw’umukobwa ugasanga Leta iri kumwirukaho ugasanga ejo heza he hakaba hasubiye inyuma.

Title: Focus group discussion-Kiziguro boys Transc.docx

Doc Creator: tcuhawenimana11

Doc Date: 3/20/2023

Codes Applied: Sources of information received during puberty

Excerpt Creator: tcuhawenimana11

Excerpt Created On: 3/21/2023

Excerpt Range: 11831-11933

Amakuru ajyanye n’ubuzima bw’imyororokere turayafite. Aho twayakuye ni mumashuri ahandi ni ku babyeyi.

Title: Focus group discussion-Kiziguro boys Transc.docx

Doc Creator: tcuhawenimana11

Doc Date: 3/20/2023

Codes Applied: Types of information provided to boys and girls during puberty

Excerpt Creator: tcuhawenimana11

Excerpt Created On: 3/21/2023

Excerpt Range: 12025-12357

Amakuru ajyanye n’imihindagurikire y’umubiri. Andi makuru mfite ni mu myitwarire ukamenya uko witwara iyo umaze kuba ingimbi mbese nk’umusore hari igihe uba utacyumvikana n’ababyeyi ariko ubwo nabashije kugirwa inama banyereka inzira y’ubuzima banandemamo icyizero cyejo hazaza kugira ngo nzabashe kugera ku ntego z’ubuzima bwanjye.

Title: Focus group discussion-Kiziguro boys Transc.docx

Doc Creator: tcuhawenimana11

Doc Date: 3/20/2023

Codes Applied: Sources of information received during puberty

Excerpt Creator: tcuhawenimana11

Excerpt Created On: 3/21/2023

Excerpt Range: 12458-12534

Ayo makuru mfite yose nayakuye ku babyeyi, mu ishuri aho ngaho haba hahagije

Title: Focus group discussion-Kiziguro boys Transc.docx

Doc Creator: tcuhawenimana11

Doc Date: 3/20/2023

Codes Applied: Sources of information received during puberty

Excerpt Creator: tcuhawenimana11

Excerpt Created On: 3/21/2023

Excerpt Range: 12669-12693

Njye nayakuye muri club.

Title: Focus group discussion-Kiziguro boys Transc.docx

Doc Creator: tcuhawenimana11

Doc Date: 3/20/2023

Codes Applied: Types of information provided to boys and girls during puberty

Excerpt Creator: tcuhawenimana11

Excerpt Created On: 3/21/2023

Excerpt Range: 12759-12969

Kumvira no kwitwararika. Urumva muganga yazaga ku ishuri akatubwira ku bijyanye n’ubuzima bw’imyororokere bityo tukabasha kumenya amakuru ajyanye nabyo. Muri club batubwiraga ibintu bigaragaza ko umuntu yakuze.

Title: Focus group discussion-Kiziguro boys Transc.docx

Doc Creator: tcuhawenimana11

Doc Date: 3/20/2023

Codes Applied: Sources of information received during puberty

Excerpt Creator: tcuhawenimana11

Excerpt Created On: 3/21/2023

Excerpt Range: 13035-13068

Amakuru mfite nayakuye ku ishuri

Title: Focus group discussion-Kiziguro boys Transc.docx

Doc Creator: tcuhawenimana11

Doc Date: 3/20/2023

Codes Applied: Sources of information received during puberty

Excerpt Creator: tcuhawenimana11

Excerpt Created On: 3/21/2023

Excerpt Range: 13174-13225

Nanjye rero amakuru ahantu nayakuye ni mu kiliziya.

Title: Focus group discussion-Kiziguro boys Transc.docx

Doc Creator: tcuhawenimana11

Doc Date: 3/20/2023

Codes Applied: Sources of information received during puberty

Excerpt Creator: tcuhawenimana11

Excerpt Created On: 3/21/2023

Excerpt Range: 13261-13309

Njye ni ku babyeyi. Hari bajyaga babituganiriza.

Title: Focus group discussion-Kiziguro boys Transc.docx

Doc Creator: tcuhawenimana11

Doc Date: 3/20/2023

Codes Applied: Sources of information received during puberty

Excerpt Creator: tcuhawenimana11

Excerpt Created On: 3/21/2023

Excerpt Range: 13372-13426

Njyewe amakuru aho nayakuye ni ku mbuga nkoranyambaga…

Title: Focus group discussion-Kiziguro boys Transc.docx

Doc Creator: tcuhawenimana11

Doc Date: 3/20/2023

Codes Applied: Sources of information received during puberty

Excerpt Creator: tcuhawenimana11

Excerpt Created On: 3/21/2023

Excerpt Range: 13458-13710

Nka youtube, n’izindi zose zikoreshwa aho basobanura ku bijyanye n’igihe cy’ubugimbi n’ibijyanye nabwo ndetse kubera ko bakenera gukora imibonano mpuzabitsina hari n’igihe bahohoterwa bakatubwira ko ufite inshingano zo kuvuga oya igihe ugiye kubikorwa.

Title: Focus group discussion-Kiziguro boys Transc.docx

Doc Creator: tcuhawenimana11

Doc Date: 3/20/2023

Codes Applied: Obstacles and risks experienced by boys during the sexual and reproductive health changes occuring during puberty and adolescence

Excerpt Creator: tcuhawenimana11

Excerpt Created On: 3/21/2023

Excerpt Range: 14093-14316

Ingora ya mbere abana b’abahungu bahura nazo ni nkuko bo ubwabo batigirira ikizere ngo bumve ko bagomba gutinyuka kubaza amakuru ajyanye n’ubuzima bw’imyororekere ahubwo ugasanga bishoye mu mibonano mpuzabitsina imburagihe.

Title: Focus group discussion-Kiziguro boys Transc.docx

Doc Creator: tcuhawenimana11

Doc Date: 3/20/2023

Codes Applied: Obstacles and risks experienced by boys during the sexual and reproductive health changes occuring during puberty and adolescence

Excerpt Creator: tcuhawenimana11

Excerpt Created On: 3/21/2023

Excerpt Range: 14417-14720

Ingorane abana b’abahungu bahura nazo mu gihe cy’ubugimbi hariho abatiyumvisha ko inama bari guhabwa arizo z’ukuri ahubwo bakumva ko kubera biyumvamo imbaraga inama bahabwa n’ababyeyi cyangwa abandi babaha inama atarizo ahubwo bo bakifuza gukora ibyo bumva bitekerereza bikarangira bibagushije mu cyaha.

Title: Focus group discussion-Kiziguro boys Transc.docx

Doc Creator: tcuhawenimana11

Doc Date: 3/20/2023

Codes Applied: Obstacles and risks experienced by boys during the sexual and reproductive health changes occuring during puberty and adolescence

Excerpt Creator: tcuhawenimana11

Excerpt Created On: 3/21/2023

Excerpt Range: 14761-14861

Urugero nk’icyaha cy’ubusambanyi, ugasanga yishoye mu biyobyabwenge bakabinwa kubera kutumvira inama

Title: Focus group discussion-Kiziguro boys Transc.docx

Doc Creator: tcuhawenimana11

Doc Date: 3/20/2023

Codes Applied: Obstacles and risks experienced by boys during the sexual and reproductive health changes occuring during puberty and adolescence

Excerpt Creator: tcuhawenimana11

Excerpt Created On: 3/21/2023

Excerpt Range: 14940-15146

Njyewe ingorane bahura nazo ashobora kwanga kumvira izo nama bamugira hanyuma akishora mu mibonana mpuzabitsina ugasanga ahanduriyemo indwara cyangwa se ugasanga ateye umukobwa inda ugasanga RIB iramufashe

Title: Focus group discussion-Kiziguro boys Transc.docx

Doc Creator: tcuhawenimana11

Doc Date: 3/20/2023

Codes Applied: How boys experience physiological changes that occured to them during puberty

Excerpt Creator: tcuhawenimana11

Excerpt Created On: 3/21/2023

Excerpt Range: 15330-15485

ngorane nahuye nazo njyewe ubwanjye nkimara kwiroteraho numvaga mbishaka cyane ariko nyine hari umuntu wangiriye inama hanyuma birangira gutyo ndabyihorera

Title: Focus group discussion-Kiziguro boys Transc.docx

Doc Creator: tcuhawenimana11

Doc Date: 3/20/2023

Codes Applied: Obstacles and risks experienced by boys during the sexual and reproductive health changes occuring during puberty and adolescence

Excerpt Creator: tcuhawenimana11

Excerpt Created On: 3/21/2023

Excerpt Range: 15534-15603

Iii, ni ingorane kuko ushobora kubishaka cyane bigatuma ugira stress.

Title: Focus group discussion-Kiziguro boys Transc.docx

Doc Creator: tcuhawenimana11

Doc Date: 3/20/2023

Codes Applied: Obstacles and risks experienced by boys during the sexual and reproductive health changes occuring during puberty and adolescence

Excerpt Creator: tcuhawenimana11

Excerpt Created On: 3/21/2023

Excerpt Range: 15664-15915

Njyewe ingorane nahuye nazo hari nkuko uba ubona abakobwa bose bashaka kukubeshya ikindi noneho badutesha umutwe cyane ikindi abanda ugasanga bashuka abanda bati wenda uteye neza babwira nk’umukobwa bityo bikaba byamukururira mu mibonano mpuzabitsina.

Title: Focus group discussion-Kiziguro boys Transc.docx

Doc Creator: tcuhawenimana11

Doc Date: 3/20/2023

Codes Applied: Obstacles and risks experienced by boys during the sexual and reproductive health changes occuring during puberty and adolescence

Excerpt Creator: tcuhawenimana11

Excerpt Created On: 3/21/2023

Excerpt Range: 15984-16228

Njyewe ingorane zambayeho ariko ntabwo zabaye gusa hari habuzeho gatoya. Urabona nyine cya gihe umuntu agera mu gihe cy’ubugimbi nyine akiroteraho hari igihe rero nari ndi kumwe n’umukobwa bimbaho nyoberwa uko byagenze habura gato ngo tubikore.

Title: Focus group discussion-Kiziguro boys Transc.docx

Doc Creator: tcuhawenimana11

Doc Date: 3/20/2023

Codes Applied: Obstacles and risks experienced by boys during the sexual and reproductive health changes occuring during puberty and adolescence

Excerpt Creator: tcuhawenimana11

Excerpt Created On: 3/21/2023

Excerpt Range: 16290-16450

Umuhungu ashobora kwishora mu mibonano mpuzabitsina ugasanga ateye inda akaba yacikishiriza amashuri cg akandura ibyo birwara bityo ubuzima bwe bukahangirikira.

Title: Focus group discussion-Kiziguro boys Transc.docx

Doc Creator: tcuhawenimana11

Doc Date: 3/20/2023

Codes Applied: Obstacles and risks experienced by boys during the sexual and reproductive health changes occuring during puberty and adolescence

Excerpt Creator: tcuhawenimana11

Excerpt Created On: 3/21/2023

Excerpt Range: 16643-16819

Nk’umuhungu akenshi atangira gutekereza kuri bya bintu byamubayeho agatangira gutsindwa mu ishuri bityo akaba yatangira kwishora mu biyobyabwenge kugira ngo bimufashe gutereta.

Title: Focus group discussion-Kiziguro boys Transc.docx

Doc Creator: tcuhawenimana11

Doc Date: 3/20/2023

Codes Applied: Received information is misleading and risky

Excerpt Creator: tcuhawenimana11

Excerpt Created On: 3/21/2023

Excerpt Range: 16865-17369

Ingorane zibaho ni uguhahabwa amakuru atizewe ibyo bintu bikakubaho noneho uwo ugiye kugisha inama akaba yaguha amakuru atizewe nk’urugero ukazana ibiheri mu maso noneho wajya kugisha inama uvuga uti ko mbona ibiheri binyishe bati wowe genda nusambana ibiheri bizakira nukora imibonano mpuzabitsina ibiheri bizakira. Icyo gihe iyo utagize ubushishozi cyangwa ngo ugire undi wundi ubaza icyo gihe ahita agushora muri izo ngorane zo gukora imibonano mpuzabitsina ejo ukaba wanatera umukobwa w’abandi inda.

Title: Focus group discussion-Kiziguro boys Transc.docx

Doc Creator: tcuhawenimana11

Doc Date: 3/20/2023

Codes Applied: Obstacles and risks experienced by boys during the sexual and reproductive health changes occuring during puberty and adolescence

Excerpt Creator: tcuhawenimana11

Excerpt Created On: 3/21/2023

Excerpt Range: 16866-17369

Ingorane zibaho ni uguhahabwa amakuru atizewe ibyo bintu bikakubaho noneho uwo ugiye kugisha inama akaba yaguha amakuru atizewe nk’urugero ukazana ibiheri mu maso noneho wajya kugisha inama uvuga uti ko mbona ibiheri binyishe bati wowe genda nusambana ibiheri bizakira nukora imibonano mpuzabitsina ibiheri bizakira. Icyo gihe iyo utagize ubushishozi cyangwa ngo ugire undi wundi ubaza icyo gihe ahita agushora muri izo ngorane zo gukora imibonano mpuzabitsina ejo ukaba wanatera umukobwa w’abandi inda.

Title: Focus group discussion-Kiziguro boys Transc.docx

Doc Creator: tcuhawenimana11

Doc Date: 3/20/2023

Codes Applied: Obstacles and risks experienced by boys during the sexual and reproductive health changes occuring during puberty and adolescence

Excerpt Creator: tcuhawenimana11

Excerpt Created On: 3/21/2023

Excerpt Range: 17456-17497

No gutwara inda cyangwa bakava mu ishuri.

Title: Focus group discussion-Kiziguro boys Transc.docx

Doc Creator: tcuhawenimana11

Doc Date: 3/20/2023

Codes Applied: Obstacles and risks experienced by girls during sexual and reproductive health changes occuring during puberty and adolescence Received information is misleading and risky

Excerpt Creator: tcuhawenimana11

Excerpt Created On: 3/21/2023

Excerpt Range: 17546-17700

Ingorane zabagaho nongeye kubyo avuze byabagaho umukobwa yaba yazanye ibiheri bakamubwira ngo asheke umuhungu bakorane sex ugasanga bivuyemo gutwara inda.

Title: Focus group discussion-Kiziguro boys Transc.docx

Doc Creator: tcuhawenimana11

Doc Date: 3/20/2023

Codes Applied: Obstacles and risks experienced by boys during the sexual and reproductive health changes occuring during puberty and adolescence

Excerpt Creator: tcuhawenimana11

Excerpt Created On: 3/21/2023

Excerpt Range: 17815-17914

Nuko iyo ahantu hose ageze batangira kumwanga bagatangira kumuvuga nabi bavuga ko yabaye icyomanzi.

Title: Focus group discussion-Kiziguro boys Transc.docx

Doc Creator: tcuhawenimana11

Doc Date: 3/20/2023

Codes Applied: Obstacles and risks experienced by girls during sexual and reproductive health changes occuring during puberty and adolescence

Excerpt Creator: tcuhawenimana11

Excerpt Created On: 3/21/2023

Excerpt Range: 17978-18448

Nko ku bakobwa ingorane bakunda guhura nazo iyo bamaze kugera mu gihe cy’ubwangavu atangira kumva yakuze kwa kundi yegeranye na bagenzi be haba ku mashuri cyangwa muri quartier aho dutuye hariho ababa baragize ubwoba bwo kugisha inama ababyeyi babo ugasanga aragiye mbese akumva yakuze nkuko bagenzi banjye babivuze ijipo igatangira kwigira harugura noneho bagatangira kwishora mu biyobyabwenge ugasanga umukobwa abaye nabi yandagaye ku muhanda ugasanga yabaye igicibwa.

Title: Focus group discussion-Kiziguro boys Transc.docx

Doc Creator: tcuhawenimana11

Doc Date: 3/20/2023

Codes Applied: Obstacles and risks experienced by girls during sexual and reproductive health changes occuring during puberty and adolescence

Excerpt Creator: tcuhawenimana11

Excerpt Created On: 3/21/2023

Excerpt Range: 18502-18707

Ingorane umukobwa ahura nazo hari nk’igihe ashaka kubaza iwabo ibijyanye n’imihindagurikire y’umubiri we ugasanga iwabo bavuga bati genda uri umwana bakamucira hanyuma ukazasanga yagiye kwishora mu buraya.

Title: Focus group discussion-Kiziguro boys Transc.docx

Doc Creator: tcuhawenimana11

Doc Date: 3/20/2023

Codes Applied: Obstacles and risks experienced by girls during sexual and reproductive health changes occuring during puberty and adolescence

Excerpt Creator: tcuhawenimana11

Excerpt Created On: 3/21/2023

Excerpt Range: 18957-19109

Urugero wenda nko ku bakobwa bashobora guhura na sugar daddy bitewe n’ubushobozi bukeya umukobwa afite bakaba bamushuka gutyo rero akumva ko yakora sex.

Title: Focus group discussion-Kiziguro boys Transc.docx

Doc Creator: tcuhawenimana11

Doc Date: 3/20/2023

Codes Applied: Obstacles and risks experienced by girls during sexual and reproductive health changes occuring during puberty and adolescence

Excerpt Creator: tcuhawenimana11

Excerpt Created On: 3/21/2023

Excerpt Range: 19131-19281

Nk’ikindi kintu navuga ni icyo kugira irari bakagira irari bigatuma nyine batagendera mu murongo mwiza bagatangira kwambara ibintu bigufi biyandarika.

Title: Focus group discussion-Kiziguro boys Transc.docx

Doc Creator: tcuhawenimana11

Doc Date: 3/20/2023

Codes Applied: Obstacles and risks experienced by girls during sexual and reproductive health changes occuring during puberty and adolescence

Excerpt Creator: tcuhawenimana11

Excerpt Created On: 3/21/2023

Excerpt Range: 19303-19569

Inzitizi bahura nazo ni ukwanga guha ababyeyi amakuru y’uko bageze muri icyo kugira ngo ababyeyi batangire kubamenyesha no kubaha buri kimwe cyose bakeneye. Bazabura buri kimwe cyose bakeneye bagatangira kubisaba ba basore baba bari hano hanze bakaba babatera inda.

Title: Focus group discussion-Kiziguro boys Transc.docx

Doc Creator: tcuhawenimana11

Doc Date: 3/20/2023

Codes Applied: Obstacles and risks experienced by girls during sexual and reproductive health changes occuring during puberty and adolescence

Excerpt Creator: tcuhawenimana11

Excerpt Created On: 3/21/2023

Excerpt Range: 19650-19841

Nanjye izo mbona ni kwa kundi wenda umukobwa aba yagiye nko mu mihango akumva nyine yaba akeneye nka fanta hanyuma nyine kubera kwa gusuzugura ababyeyi yavuye nko mu rugo akajya kuba umuyaya.

Title: Focus group discussion-Kiziguro boys Transc.docx

Doc Creator: tcuhawenimana11

Doc Date: 3/20/2023

Codes Applied: Obstacles and risks experienced by boys during the sexual and reproductive health changes occuring during puberty and adolescence

Excerpt Creator: tcuhawenimana11

Excerpt Created On: 3/21/2023

Excerpt Range: 19998-20119

Umuhungu ni ukuba yifata nk’umusore akumva nta muntu wamuvugaho bityo bigahera aho asuzugura bikazamuviramo kuba ikirara.

Title: Focus group discussion-Kiziguro boys Transc.docx

Doc Creator: tcuhawenimana11

Doc Date: 3/20/2023

Codes Applied: Obstacles and risks experienced by boys during the sexual and reproductive health changes occuring during puberty and adolescence

Excerpt Creator: tcuhawenimana11

Excerpt Created On: 3/21/2023

Excerpt Range: 20141-20242

Indi nzitizi ku bahungu nuko bagendera mu kigare akumva ko nimba runaka yarateye inda nawe yayitera.

Title: Focus group discussion-Kiziguro boys Transc.docx

Doc Creator: tcuhawenimana11

Doc Date: 3/20/2023

Codes Applied: Obstacles and risks experienced by boys during the sexual and reproductive health changes occuring during puberty and adolescence

Excerpt Creator: tcuhawenimana11

Excerpt Created On: 3/21/2023

Excerpt Range: 20300-20447

Indi nzitizi ishobora kubaho nuko nk’ubungubu iyo umuhungu ari kuganira n’abandi basore bagenzi be bamubwira ko atarabikora ari imbwa bakaba nyine…

Title: Focus group discussion-Kiziguro boys Transc.docx

Doc Creator: tcuhawenimana11

Doc Date: 3/20/2023

Codes Applied: Received information is misleading and risky

Excerpt Creator: tcuhawenimana11

Excerpt Created On: 3/21/2023

Excerpt Range: 20300-20447

Indi nzitizi ishobora kubaho nuko nk’ubungubu iyo umuhungu ari kuganira n’abandi basore bagenzi be bamubwira ko atarabikora ari imbwa bakaba nyine…

Title: Focus group discussion-Kiziguro boys Transc.docx

Doc Creator: tcuhawenimana11

Doc Date: 3/20/2023

Codes Applied: Received information is misleading and risky Obstacles and risks experienced by boys during the sexual and reproductive health changes occuring during puberty and adolescence

Excerpt Creator: tcuhawenimana11

Excerpt Created On: 3/21/2023

Excerpt Range: 20541-20684

No kugendera mu kigare urumva bagatangira bamutuka ngo wowe uri imbwa nyine bamushora muri ibyo ngibyo bamushuka ngo kuba atarabikora ni imbwa.

Title: Focus group discussion-Kiziguro boys Transc.docx

Doc Creator: tcuhawenimana11

Doc Date: 3/20/2023

Codes Applied: Suggestions to improve SRH during puberty and adolescence by boys

Excerpt Creator: tcuhawenimana11

Excerpt Created On: 3/21/2023

Excerpt Range: 20907-21065

Njyewe ndumva nk’abayobozi bagira icyo bakora wenda bagatangiza nk’ubukangurambaga mu mudugudu runaka urubyiruko rukarushaho kumenya ibijyanye n’imyororokere.

Title: Focus group discussion-Kiziguro boys Transc.docx

Doc Creator: tcuhawenimana11

Doc Date: 3/20/2023

Codes Applied: Suggestions to improve SRH during puberty and adolescence for girls

Excerpt Creator: tcuhawenimana11

Excerpt Created On: 3/21/2023

Excerpt Range: 21107-21357

Murakoze indi ni ukubashishikariza gutega amatwi radiyo mu makinamico akenshi na kenshi bakunda bakunda kwereka bimwe mu bibazo urubyiruko ruhura nabyo cyangwa se abakobwa bahura nabyo n’uburyo n’inama ku buryo bashobora kubyitwaramo mu gihe byabaye.

Title: Focus group discussion-Kiziguro boys Transc.docx

Doc Creator: tcuhawenimana11

Doc Date: 3/20/2023

Codes Applied: Suggestions to improve SRH during puberty and adolescence by boys

Excerpt Creator: tcuhawenimana11

Excerpt Created On: 3/21/2023

Excerpt Range: 21415-21678

Njyewe ikifuzo mfite nuko aya makuru yose tugenda dukura ahantu hatandukanye ku bijyanye n’ubuzima bw’imyororokere byibura yakusanyirizwa ahantu hamwe akaba yajya mu masomo nk’ayandi tukajiya tuyiga byimbitse byimbitse ndetse n’abazadukomokaho bakazaza babisanga.

Title: Focus group discussion-Kiziguro boys Transc.docx

Doc Creator: tcuhawenimana11

Doc Date: 3/20/2023

Codes Applied: Suggestions to improve SRH during puberty and adolescence by boys

Excerpt Creator: tcuhawenimana11

Excerpt Created On: 3/21/2023

Excerpt Range: 21700-21880

Icyo njyewe nakongeraho nuko amakuru batwigisha kubijyanye n’ubuzima bw’imyororokere bitazajya biba amasigaracyicaro ahubwo bikaba byiza ko tuyajyana ahantu hose tukazaza tuyibuka.

Title: Focus group discussion-Kiziguro boys Transc.docx

Doc Creator: tcuhawenimana11

Doc Date: 3/20/2023

Codes Applied: Suggestions to improve SRH during puberty and adolescence by boys

Excerpt Creator: tcuhawenimana11

Excerpt Created On: 3/21/2023

Excerpt Range: 21982-22215

Njyewe icyo nakongeraho cyangwa nasaba nukugirango ibiganiro nk’abantu bakuru bageze muri icyo gihe cyangwa se nk’urubyiruko nkatwe twebwe twasaba ubugizi bikajya bica ku maradiyo inshuro nyinshi cyane noneho tukajya duhora tubyumva.

Title: Focus group discussion-Kiziguro boys Transc.docx

Doc Creator: tcuhawenimana11

Doc Date: 3/20/2023

Codes Applied: Suggestions to improve SRH during puberty and adolescence by boys

Excerpt Creator: tcuhawenimana11

Excerpt Created On: 3/21/2023

Excerpt Range: 22237-22495

Njyewe icyo nasaba nuko wenda babicisha nko mu mashuri hakabaho nk’umunsi wo kubwira abanyeshuri bakabibabwiraho kubera ko hari benshi badakurikira izo radiyo bakabibabwira ku kigo cy’amashuri cyangwa se bakagira nk’umunsi mu midugudu urubyiruko rwigishaho.

Title: Focus group discussion-Kiziguro boys Transc.docx

Doc Creator: tcuhawenimana11

Doc Date: 3/20/2023

Codes Applied: Suggestions to improve SRH during puberty and adolescence by boys

Excerpt Creator: tcuhawenimana11

Excerpt Created On: 3/21/2023

Excerpt Range: 22535-22655

Icyo njye nasaba nukongera ibigo by’urubyiruko kuko hariya niho rubasha gukura amakuru menshi ku buzima bw’imyororokere.
