# Supplementary material for: Adolescent awareness and experience of the pubertal changes: A qualitative study from Rwanda
Source: PLoS One. 2025 Jun 24;20(6):e0325502. doi: 10.1371/journal.pone.0325502 (PMC12186878; doi:10.1371/journal.pone.0325502)
Supplement: Attachment — Submitted filename: Rebutal letter-Kellen et al. 2024-Kellen.pdf [file pone.0325502.s004.pdf]

| Comments | Responses | Page within the manuscript |
|---|---|---|
| Please ensure that your manuscript meets PLOS ONE's style requirements, including those for file naming. The PLOS ONE style templates can be found at https://journals.plos.org/plosone/s/file?id=wjVg/PLOSOne_formatting_sample_main_body.pdf and https://journals.plos.org/plosone/s/file?id=ba62/PLOSOne_formatting_sample_title_authors_affiliations.pdf | Thank you for the comment. We have checked the requirements and have adhered to them. | |
| The introduction part in abstract contains grammatical errors and awkward phrasing, making it difficult to understand. For example, "Adolescence experienced puberty changes both biological and psychological changes that takes place during adolescent age and affects them differently" is confusing and should be rephrased for clarity. | Thank you for the comment. We have revised the introductory part of the abstract and addressed the grammatical errors and awkward phrasing that were appearing. | See Page 1 |
| Its not highlight what is novel or unique about this study compared to previous research on the topic. Emphasizing any new contributions or insights would strengthen the abstract. | We really appreciate this comment. First, we revised the conclusion of the manuscript to indicate what our study is contributing to the evidence and practice. In addition, in the main manuscript we have revised the discussion and conclusion sections to highlight the uniqueness of this study. | |
| Improved sentence structure and grammar for better readability and understanding. | We have crosschecked the manuscript to address some grammatical errors and structure related issues that affected the readability of the manuscript. | Changes have been made throughout the manuscript. |
| Ensured consistent use of terms such as "puberty," "adolescents," and "children." | Thank you for these important observations. We resolved to use puberty and children to ensure consistency | See pages 1,2,3,5,6,7, 10,12,16,19,20,21 and 22 |
| The methodology mentions a descriptive qualitative design but does not provide details on the specific qualitative methods used | The comment is appreciated. Details were given on the specific qualitative methods used. | 7 |
| The sampling approach is not clearly justified. The criteria for selecting participants are not fully explained. | The sampling approach were clearly justified and selecting participants criteria were explained | 7-8 |
| The discussion reiterates the findings but does not delve deeply into their implications | Thank you for this important observation. We improved the discussion section to also comment on the implications of some of the findings. | 19-22 |

| | | |
|---|---|---|
| While the discussion compares the study's findings with those from Kenya and Nigeria, it does not provide a detailed analysis of why these differences might exist. Exploring cultural, social, or educational factors that could explain these differences would add depth | Thanks for the comment. A detailed analysis on comparison study findings were addressed in the manuscript. | 20-21 |
| The conclusion does not emphasize the significance or potential impact of the study's findings on the field of adolescent health or education | Thank you very much. We revised and elaborated the conclusion section of our manuscript to indicate the significance or potential impact of our study's findings on the field of adolescent children's health or education. | 22-23 |
