# Supplementary material for: Adolescent awareness and experience of the pubertal changes: A qualitative study from Rwanda
Source: PLoS One. 2025 Jun 24;20(6):e0325502. doi: 10.1371/journal.pone.0325502 (PMC12186878; doi:10.1371/journal.pone.0325502)
Supplement: Attachment — Submitted filename: Rebutal letter-Muganwa Kellen Study-Revised 2.pdf [file pone.0325502.s006.pdf]

Point-by-point response to reviewers' comments

Comments	How the comments have been addressed
Academic Editor	
Please review your reference list to ensure that it is complete and correct. If you have cited papers that have been retracted, please include the rationale for doing so in the manuscript text, or remove these references and replace them with relevant current references. Any changes to the reference list should be mentioned in the rebuttal letter that accompanies your revised manuscript. If you need to cite a retracted article, indicate the article's retracted status in the References list and also include a citation and full reference for the retraction notice.	*We appreciate your guidance. Upon reviewing the references, we found that none of the studies we cited have been retracted.*
Reviewer 2	
The authors have responded to a number of comments.<br>But I still think it is poor in terms of writing and English.	*We appreciate your feedback. We have thoroughly reviewed the manuscript and made corrections to several issues, specifically where words were omitted, and we have also improved the sentence structure in various sections to enhance the overall readability of the paper.*
The interview questions are not clearly stated.	*Thank you very much. Since we have included the interview guide in the supplementary documents, we deemed it unnecessary to incorporate all the questions from the main manuscript.*
There are inaccuracies in the text, for example, the themes changed after revision. But the names of the themes in the text and the abstract are not the same.	*We have examined the text and corrected the inaccuracies. The content in the results section of the abstract has been aligned to correspond with the findings obtained.*
Innovation and creativity in a study have nothing to do with intervention. Every study should have creativity. You didn't mention innovation and the gap knowledge.	*Thank you very much for this observation. We have carefully considered this issue and highlighted the gap in knowledge that led to the conduct of this study. For details, see pages 5-6 of the revised document with track changes.*
The inclusion and exclusion criteria are not modified. The inclusion criteria should mention all the characteristics of the participants. You should know that the readers should fully understand your study. For example, gender is not mentioned in the inclusion criteria.	*Thank you very much. We have revised the inclusion and exclusion criteria. Clarifications were made to show who was included and excluded from the study. For detail, see page 7 of the revised document with track changes.*
