# Supplementary material for: Adolescent awareness and experience of the pubertal changes: A qualitative study from Rwanda
Source: PLoS One. 2025 Jun 24;20(6):e0325502. doi: 10.1371/journal.pone.0325502 (PMC12186878; doi:10.1371/journal.pone.0325502)
Supplement: Attachment — Submitted filename: Rebutal letter-Muganwa Kellen Study.docx [file pone.0325502.s005.docx]

**Point-by-point response to reviewers’ comments**

| **Comments** | **How the comments have been addressed** |
| --- | --- |
| ***Reviewer 1*** | |
| Please provide a table or image illustrating the process of extracting themes from the code. Considering that the immersion of a qualitative researcher is necessary for extracting data, how was this done by a team, please mention the evidence of this method. | *Thank you for this insight. We have provided a table illustrating how themes and subthemes emerged.* |
| How is the trustworthiness of the study measured? | *Thank you very much. Since this paper is qualitative, we have indicated how we followed rigorous methods to reach transferable findings.* |
| ***Reviewer 2*** | |
| Your article is valuable, but it has fundamental flaws, which are listed below. | *Thank you for the feedback. We have tried as much as we could to address some of the flaws you have highlighted as you will find in the revised version of this manuscript.* |
| 1-Rwandan may be unknown to many readers from other continents. Maybe it would be better to write about African adolescents. | *We greatly appreciate your valuable insight. After careful consideration of your suggestion, we concluded that it is essential to retain 'Rwanda' in the title. This decision is based on the fact that we have not conducted a similar study in any other African country, which would provide a broader comparative context beyond Rwanda.* |
| 2-In title: adolescent or children??? The title should indicate the target group. | *We maintained adolescent as the target group.* |
| 3- Rwandan adolescent children’s awareness and experience of the pubertal changes: a qualitative study: suggest a change to : Adolescent awareness and experience of the pubertal changes: a qualitative study from Africa | *We have formulated the study title as follows: ‘****Adolescent awareness and experience of the pubertal changes: a qualitative study from Rwanda’*** |
| Abstract: 1-Modify the abstract structure according to the journal guidelines. It is too long and vague. | *We have revised the abstract section to make it clear and made it succinct from 417 words to 276 words.* |
| 2-Mention the purpose of the study in the abstract. | *Addressed* |
| 3-Mention the method of your analysis in the abstract. | *Thank you. We have addressed this.* |
| 4-You wrote that 4 themes were identified. Write the names of the themes and categories in the findings. | *Thank you. We have addressed the comment.* |
| 5-Please, rewrite the conclusion based on the results. | *The conclusion was rewritten to reflect the results.* |
| 6- Choose our keywords based on mesh | *Addressed.* |
| Introduction: 1-The manuscript needs editing as it lacks proficiency in English. | *We appreciate the comment, and we have particularly paid attention to reducing the issue of long paragraph highlighted. Though English is not our first language, we have done our best to make the section more comprehensible.* |
| 2-Long paragraphs with only one reference?? | *Thank you for highlighting this issue. We have revised the introduction section to reduce the length of paragraphs and further improve the references.* |
| 3-The introduction is too long and vague, does not indicate the purpose of the study, does not indicate the GAP knowledge | *We appreciate this observation. Based on this, we have revised the introduction section to first reduce its length, demonstrate the research gap, and further highlighted why this study was needed in the context of Rwanda.* |
| 4- You must demonstrate the innovation of your work in the introduction. | *Thank you for the comment. Our study did not intend to develop any intervention as a innovation but instead we sought to first highlight gaps in adolescents’ knowledge of pubertal changes and from their experiences of puberty, we sought to highlight areas that would need strengthening in the current sexual education programs in Rwanda.* |
| Methods: 1-There is no need to define adolescence based on WHO in the method. | *Thank you. We have addressed this issue.* |
| 2-What are your inclusion and exclusion criteria? | *Thank you. We have added the exclusion criteria to complement the inclusion criteria for our study.* |
| 3-Did you use any special software to analyze qualitative data? | *Thank you. We used Dedoose software to analyse the data.* |
| 4-Explain more about the analysis method. | *Thank you very much. We have revised the sub-section of data analysis to explain the process of analyzing our data.* |
| 5-Mention some of the interview questions. | *We have provided the key questions that comprised the topic guide used during the moderation of the focus group discussions.* |
| 6-On what basis were the interview questions developed? | *Thank you. We have indicated that the questions were developed based on the study’s objectives and literature about adolescents’ experiences of pubertal changes.* |
| 7-Was there a specific order in which the questions were asked in the interview? | *We appreciate your feedback. In the manuscript, we have provided a list of questions posed to participants, and the sequence in which they are presented mirrors the order utilized by researchers to facilitate the focus group discussion.* |
| Results and discussion: 1-You wrote 4 themes in the abstract, and 5 themes in the findings. Which one is correct?? | *Thank you for your observation. With the current revised version, two major themes and their subthemes are presented.* |
| 2-Theme names should be short, descriptive, and comprehensive | *Thank you. Within the current version, we took into consideration this comment. Theme names have been shortened to ensure they reflect the study objectives. In addition, we also subdivided the themes into sub-themes reflecting themes to ensure more comprehension.* |
| 3-Please provide one sentence from the contributors for each sub-category. | *Thank you. We have considered this suggestion.* |
| 4-The findings are too long. It is suggested that you present the findings in a table and briefly summarize them in the text. | *We really appreciate this observation. We have summarized tried to make the results section succinct and more comprehensive as suggested.* |
| 5-You just had a theme? How about sub-themes or codes? | *Thank you. We have taken the comment into consideration.* |
| 6-In the first paragraph, briefly present the findings and in the following paragraphs, discuss the findings. | *Thank you for your observation. We have addressed this in the revised manuscript. See page…* |
| 7-what are your limitations, strengths points, and suggestions? | *Thank you for this suggestion. Throughout the discussion section we have highlighted some limitations and suggestions for further work to expand on our findings. We have also provided a section of methodology limitation of our study and suggested further research.* |
